# Sialome of a Generalist Lepidopteran Herbivore: Identification of Transcripts and Proteins from *Helicoverpa armigera* Labial Salivary Glands

**DOI:** 10.1371/journal.pone.0026676

**Published:** 2011-10-27

**Authors:** Maria de la Paz Celorio-Mancera, Juliette Courtiade, Alexander Muck, David G. Heckel, Richard O. Musser, Heiko Vogel

**Affiliations:** 1 Department of Entomology, Max Planck Institute for Chemical Ecology, Jena, Germany; 2 Department of Biological Sciences, Western Illinois University, Macomb, Illinois, United States of America; University of South Florida College of Medicine, United States of America

## Abstract

Although the importance of insect saliva in insect-host plant interactions has been acknowledged, there is very limited information on the nature and complexity of the salivary proteome in lepidopteran herbivores. We inspected the labial salivary transcriptome and proteome of *Helicoverpa armigera*, an important polyphagous pest species. To identify the majority of the salivary proteins we have randomly sequenced 19,389 expressed sequence tags (ESTs) from a normalized cDNA library of salivary glands. In parallel, a non-cytosolic enriched protein fraction was obtained from labial salivary glands and subjected to two-dimensional gel electrophoresis (2-DE) and de novo peptide sequencing. This procedure allowed comparison of peptides and EST sequences and enabled us to identify 65 protein spots from the secreted labial saliva 2DE proteome. The mass spectrometry analysis revealed ecdysone, glucose oxidase, fructosidase, carboxyl/cholinesterase and an uncharacterized protein previously detected in *H. armigera* midgut proteome. Consistently, their corresponding transcripts are among the most abundant in our cDNA library. We did find redundancy of sequence identification of saliva-secreted proteins suggesting multiple isoforms. As expected, we found several enzymes responsible for digestion and plant offense. In addition, we identified non-digestive proteins such as an arginine kinase and abundant proteins of unknown function. This identification of secreted salivary gland proteins allows a more comprehensive understanding of insect feeding and poses new challenges for the elucidation of protein function.

## Introduction

Many triploblastic metazoans benefit from a functional gland apparatus dedicated to produce saliva, a substance that in most cases lubricates their mouthparts and aids in predigestion. In addition, saliva may contain components crucial for a particular adaptation, from building a nest [1] to disarming a host's antibleeding defense [2]. In humans, salivary constituents and their function have been extensively studied to the point of using saliva as a diagnostic medium for various biochemical tests. The human salivary proteome is composed of more than 1300 proteins and ongoing proteomic studies are performed to understand its quantitative and qualitative plasticity and find disease-related biomarkers [3]. The saliva produced by blood-feeding arthropods has also been well characterized. High-throughput approaches, including Proteomics, have been utilized to identify the secreted salivary constituents of vectors such as ticks, triatomines, fleas, flies and mosquitoes [2], [4], [5] aiming to find good targets to control the diseases they transmit. It has been observed that blood-feeding animals share salivary constituents which function is antihemostatic such as vasodilators, inhibitors of blood coagulation and platelet aggregation [4].

More recently, salivary proteins or secreted proteomes of three different insect herbivore species have been elucidated [6], [7], [8]. The protein profiles corresponding to these three aphid species reflect more differences than similarities among each other. However, this discrepancy may represent the different interaction between each aphid species and its host(s) [7]. The salivary constituents may be also very different depending on the particular feeding strategy used by an insect herbivore. Aphids, piercing the plant tissue intercellularly until reaching phloem cells, trigger a totally different plant defense response than the mostly jasmonic acid-regulated one triggered by a chewing caterpillar [9], [10]. The complexity and identity of caterpillar saliva constituents has not been studied in detail. However, there is evidence that a glucose oxidase produced by *Helicoverpa zea* is the primary salivary factor to suppress the induction of nicotine in tobacco plants and that saliva of this same lepidopteran species has antibacterial properties [11], [12], [13]. In turn, elicitors of plant defense responses have been found in caterpillar regurgitate [14] which may include salivary components. The Old World cotton bollworm, *H. armigera* (Har) belongs to a “major-pest lineage” of the cosmopolitan subfamily Heliothinae (Lepidoptera:Noctuidae) [15]. Efforts to understand the digestive system of this generalist herbivore include the identification of its larval midgut lumen proteome [16]. In turn, the insect gut has an intricate relationship with the salivary glands. It has been stated that during larval feeding, the plant tissue is sheared with the mandibles and passes through the foregut where it is mixed with digestive secretions from the salivary glands [16]. The salivary apparatus is represented by the long and tubular labial glands and the relative smaller mandibular glands. One of the characteristics of most Endopterygota is the ability of their larvae to produce protein threads (silk) from their labial glands. Therefore, silk production may be an ancestral function of the labial salivary glands in Lepidoptera [17], [18]. In the domesticated mulberry silkworm, *Bombyx mori*, the labial glands are referred as “silk glands” since they produce massive amounts of silk proteins during the final stages of larval development. Due to its economic relevance, these silk proteins are the best characterized components of lepidopteran labial saliva [17], [19]. Here, we use an unbiased high-throughput approach, to expand the current knowledge on labial saliva produced by a generalist phytophagous insect and in particular, to aid understanding the role of saliva in Har digestion and elicitation of host plant responses. For this purpose, we generated a salivary-gland transcriptome dataset and examined the non-cytosolic enriched protein fraction from labial salivary glands using two-dimensional gel electrophoresis, identifying the proteins using de novo peptide sequencing and public database searches including sequence information from diverse Har cDNA libraries.

## Results and Discussion

### Har salivary gland cDNA library

Normalization of the Har salivary gland cDNA resulted in reduction of any over-abundant transcripts and production of a more even distribution of transcripts ranging from 0.2 to >4.0 kb in size. The average size of the cDNAs of the Har salivary gland cDNA library that were cloned and sequenced was 1,040 bp. The total number of high quality reads subsequently used for the assembly was 19,389 with an average length of read (bases) of 548 after vector clipping and quality trimming. Expressed sequenced tag (EST) clustering resulted in a total of 2,826 contiguous sequences (contigs; with 2 to 485 ESTs) and 5,463 singletons represented by a single EST, yielding a total of 8,289 putative gene objects. We found that 50% of the contigs are above 980 bases with the largest contig having 3,172 bases. The deduced sequences from 5,056 clusters (61% of total clusters) shared significant similarities with protein sequences deposited in non-redundant databases (EMBL/Genbank), a proportion comparable to that found in other studies of insect sialotranscriptomes [5], [20], [21]. One has to note that the transcripts of the unknown class could represent novel proteins or derive from the less conserved 3′ or 5′ untranslated regions of genes, as was indicated for the transcriptomes in other insects [5], [20], [21].

### Functional analyses using Gene Ontologies

For functional comparisons, all sequences were subjected to Gene Ontology (GO) analysis in Blast2GO, where we classified all gene objects in Biological Function, Molecular Process and Cellular Component. Of the 5,056 contigs in the Har salivary gland cDNA library with high-score matches in the Genbank non-redundant (nr) protein database, 4,173 (82.5%) shared significant similarity with proteins with assigned molecular functions in the GO database and thus could be classified into a GO category, with each class containing at least 21 sequences (0.5% of 4,173). Blast-positive clusters were classified into 10 molecular functional categories at level 2 of the gene ontology system (Figure S1), among which “binding” (GO:0005488) and “catalytic activity” (GO:0003824) categories were over-represented (43% and 35%, respectively), followed by “structural molecule activity” function (GO:0005198) and “transporter activity” (GO:0005215). Most of these dominant GO categories are also the most common functional categories identified in the venom gland transcriptome of a parasitic wasp [22]. Transcript abundance is another indication of how important the proteins they code for can be to the specific organ or tissue, such as the case of digestive proteases in gut tissue. The most highly expressed genes in the moderately normalized Har salivary gland library encoded proteins involved in mitochondrial respiratory chain and ATP synthase proteins (cytochrome C, vacuolar ATP synthase), general cellular homeostasis, ribosomal proteins but also glucose oxidase and glucose dehydrogenase (belonging to the GMC oxidoreductase superfamily), coagulin, fibroin, lipases and protease inhibitors (e.g. brasiliensin) (Table S1).

When comparing the GO terms obtained from the salivary gland tissue library sequences with those obtained from other Har tissue-specific cDNA libraries (gut and hemocytes), there are clear differences in the relative representations of certain functional categories. Examples for such an over-representation of the GO categories are hydrolase and oxidoreductase activity, which are more prominent in the gut tissue versus both hemocytes and salivary gland tissue, while the category structural molecule activity is more prominent in the salivary gland tissue as compared to both other tissues (Figure S2). Overall, the assembly into 8,289 contigs from the salivary gland of Har and subsequent sequence annotation and functional categorization has revealed that this tissue is more complex than we envisioned beforehand. The encountered complexity can be at least partially addressed by identification of candidate gene groups.

### Pre-digestion gene candidates

A very important aspect of any dietary constraints in Lepidoptera is the availability of proteins and nitrogen in the respective diets and the abundance of functional larval digestive enzymes to access these resources. Plant tissues are not only characterized by high levels of non-digestible materials such as cellulose and lignin, but leaves usually also contain low levels of both protein and lipids (i.e. triglycerides, phospholipids and galactolipids). The insect midgut has classically been viewed as a tissue primarily involved in digestion and detoxification and endopeptidases such as serine proteases (trypsin and chymotrypsin-like) are thought to play the dominant role in protein hydrolysis as well as exopeptidases of varying terminal amino acid specificity (aminopeptidases and carboxypeptidases). So far the lepidopteran salivary gland has not explicitly been seen as an additional, and potentially important, source of enzymes involved in pre-digestion of plant materials. However, the pre-digestion of food may occur already on the damaged plant tissue, provided that enough saliva is secreted, or takes place outside the midgut, for example, in the crop or foregut or even in some extent inside the oral cavity. Digestive enzymes in this case either come from the salivary gland alone or can be passed forward from the midgut and are then mixed with salivary gland enzymes. It is noteworthy to mention that, given the feeding of a chewing herbivore, salivary gland-derived enzymes would likely also end up in the midgut, thus adding to the midgut tissue-derived enzymatic composition of the gut lumen. In support of a role of the lepidopteran salivary gland in plant predigestion, our Har salivary gland cDNA library contains a range of contigs coding for proteases, lipases and amylases. Among the contigs with similarity to proteases, seven code for trypsin-like serine proteases, most of which display highest similarity to silk-gland derived serine proteases and trypsins form *Ostrinia nubilalis* with unknown functions. Among the proteases known to be present in the digestive enzyme repertoire of gut tissues, we could also identify 3 different carboxypeptidases but were unable to identify any sequence with homology to aminopeptidases. These proteases could act in concert, thus contributing to the efficient use of the low nitrogen-content of the ingested plant material which will be completed by the gut enzymes.

In addition to nitrogen acquisition through the concerted action of proteases, insect herbivores need to have a range of lipases in order to overcome their host plant limitations in lipid content. For example, deficiency of cholesterol, normally synthesized from phytosterols, leads to increased larval mortality and reduced egg hatch [23]. Several studies have characterized lipid metabolic activities from insects. These include lipase [24], [25] and phospholipase A2 [26]. Digestive lipases and phospholipases are key enzymes in processing dietary lipids and enzyme activities have been identified in lepidopteran larval midgut, fat body and salivary gland [27]. Our Har salivary gland cDNA library codes for 8 lipases and 2 phospholipases and two contigs coding for lipases are among the most highly expressed genes in the salivary gland (Table S1), pointing at an important role of these in plant tissue pre-digestion.

**Figure 1:**
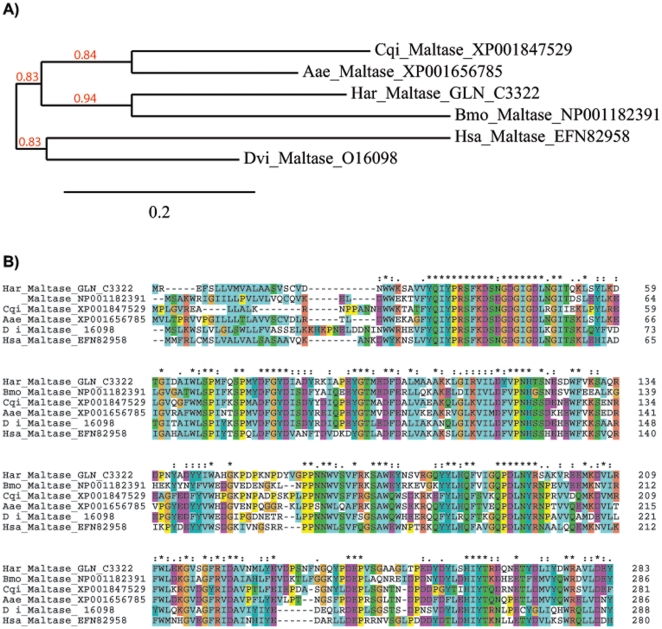
Phylogenetic tree and amino acid alignment of insect maltases. (A) An unrooted Bayesian inference tree constructed from the alignment of amino acid sequences presented in (B). The *Helicoverpa* (Har) sequence clusters with the predicted maltase from *Bombyx mori* (Bmo) with good bootstrap support. (B) The complete predicted polypeptide sequences of 5 insect maltases and the identified *Helicoverpa* maltase are aligned. Amino acid sequence alignments were performed using MAFFT multiple alignment program. Identical residues are color-coded and residues highly conserved in all arthropod CLPs are marked with asterisks. Species abbreviations: *Drosophila virilis* (Dvi), *Harpegnathos saltator* (Hsa), *Aedes aegypti* (Aae), *Culex quincefasciens* (Cqu). GenBank accession numbers are given at the end of sequence names.

Though low in proteins and lipids, plant tissue is often a rich source of starch and sugars. In humans starch degradation starts in the oral cavity, where an amylase enzyme in saliva begins to break down starch into disaccharides such as maltose but also into dextrin. We have identified several putative alpha-amylases and a maltase with high similarity to Dipteran salivary maltases. Alpha-amylase genes often form multigene families in living organisms and this multigene family has been extensively studied in Diptera [28]. It is therefore interesting to note that there is a single predicted alpha-amylase sequence of a lepidopteran insect in the NCBI nr database, as all other hits of the Har salivary amylases are against Diptera or Hymenoptera. However, Blast searches against the NCBI dbEST database lead to multiple hits against insect ESTs mostly derived from midgut cDNA libraries, pointing at a lack of annotated amylase genes in public databases containing Lepidoptera sequences. Among the three different alpha-amylases form Har salivary glands, one predicted protein sequence shows a much higher similarity to Dipteran alpha-amylases as compared to the existing lepidopteran enzymes present in the NCBI nr database. One of the identified alpha-amylases, Har_Contig 3039, coding for only a partial protein sequence, is identical to an alpha-amylase previously identified in the gut lumen of Har (ABU98614) [16]. To examine the relationships among maltase proteins identified in Har salivary glands and those found in other insects, sequences from six insect species were aligned and used to construct a gene phylogeny (Figure 1A). The phylogenetic analysis revealed that these sequences clustered in distinct clades according to species phylogeny, with both lepidopteran maltase sequences clearly separated with a high bootstrap support. Overall, the sequence alignment of Har and all other insect maltases display multiple highly conserved amino acids (Figure 1B). As the salivary glands in Har express both, alpha-amylases and a maltase, pre-digestion of complex carbohydrates could process from cleavage into disaccharides by the action of the alpha-amylases and the release of the sugar glucose through the action of maltase. We also have identified a salivary gland beta-glucanase (Contig_1557; Contig_4824) and a fructosidase (EF600050) previously identified as digestive enzymes in the gut of Har [16], [29].

In addition to merely aid in the digestion of plant nutrients, salivary gland enzymes could aid in host plant penetration, detoxify plant defensive phytochemicals, but could also both induce and degrade plant wound messengers. Several ribonucleases (RNases) are prominent in the Har salivary gland transcriptome, among which we have identified a contig with high sequence similarity to salivary secreted ribonucleases found in e.g. *Glossina morsitans* [2]. Besides being active in nutrient acquisition through the degradation of ribonucleic acid, RNase has been shown to, when applied to wounded plant tissue, induce pathogen defense response in the attacked plant [30].

For the closely related species *H. zea* it was shown that a glucose oxidase enzyme can manipulate inducible plant defenses to benefit the herbivore when this enzyme gets into contact with wounded plant tissues [11]. In our salivary gland transcriptome dataset we have identified several gene objects with homology to glucose oxidase/glucose dehydrogenases, all of which belong to the superfamily of glucose-methanol-choline oxidoreductases (GMCs). The GMC oxidoreductase gene family is known for a variety of substrates and catalytic activities [31], [32] and has been characterized at the molecular and functional level in a beetle-host plant interaction system, where a specific GMC oxidoreductase is involved in beetle chemical defense [33]. However, with the exception of a few GMC proteins, very little is known about the specific roles of members of this gene family.

### Immunity-related transcripts

Lepidoptera, as other insects, protect themselves against microbial infections through several defensive molecules, including the diverse group of antimicrobial peptides (AMPs). Many AMPs can lyse microbes, although this has only been directly shown with individual AMPs in few cases, while others can also act as eukaryotic cytolysins [34], [35]. An emerging pattern and seemingly common feature of blood-sucking or plant sap-feeding insect sialotranscriptomes analyzed (which mostly excludes Lepidoptera) is the presence of AMPs such as defensins, cecropins, and lysozyme, as well as pattern recognition molecules (e.g. Gram-negative binding proteins (GNBPs), beta-1,3 glucan recognition protein (BGRP) and C-type lectins) and serine proteases that may act as proximal activators of the prophenoloxidase or proteolytic cascades [20], [36]. Our transcriptomic analysis resulted in identification of a large number of AMPs among which are gloverin, attacin, cecropin, defensin, heliomicin, several lysozymes, pattern recognition proteins such as BGRP and ESTs (Contig_1415+Contig_719) with homology to an inducible metalloproteinase inhibitor identified and described in *G. mellonella* [37]. The extent of antimicrobial defense molecule complexity expressed in Har salivary glands was somewhat surprising, but is in line with what was found in another tissue of Lepidoptera exposed to the outside, i.e. pheromone glands of *Heliothis virescens* female moths [63].

#### Lysozymes

**Figure 2:**
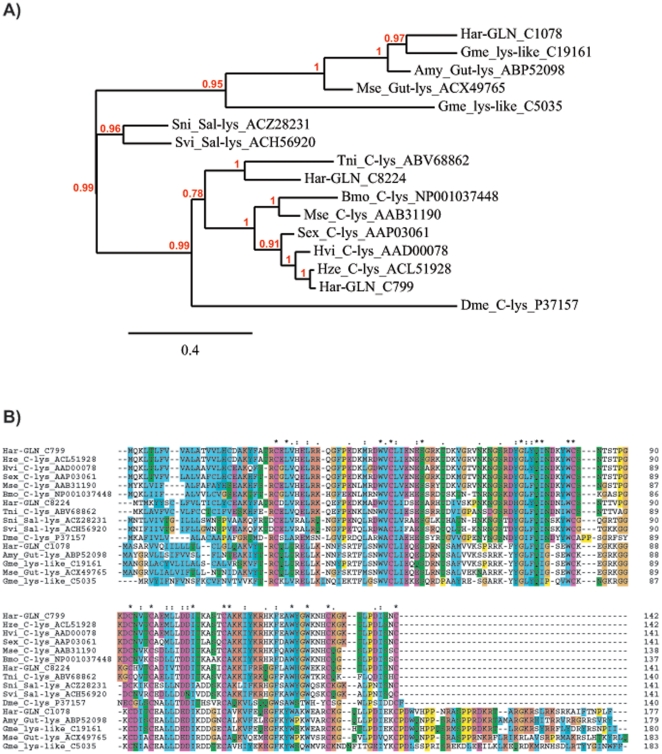
Gene phylogeny and amino acid alignment of C-type lysozyme protein sequences. (A) An unrooted Bayesian inference tree constructed from the alignment of lysozyme amino acid sequences presented in (B). Bayesian posterior probabilities are shown for all major nodes supported with probability higher than 60%. (B) Amino acid alignment of the three predicted proteins from *Helicoverpa* (Har) together with predicted protein sequences deduced from publicly available insect sequence datasets. Amino acid sequence alignments were performed using MAFFT multiple alignment program. Identical residues are color-coded and marked with asterisks above the alignment. Species abbreviations: *Galleria mellonella* (Gme), *Antheraea mylitta* (Amy), *Manduca sexta* (Mse), *Simulium nigrimanum* (Sni), *Simulium vittatum* (Svi), *Trichoplusia ni* (Tni), *Bombyx mori* (Bmo), *Spodoptera exigua* (Sex), *Heliothis virescens* (Hvi), *Helicoverpa zea* (Hze), *Drosophila melanogaster* (Dme). GenBank accession numbers are given at the end of sequence names.

We identified four different lysozymes expressed in the salivary glands of Har. Lysozymes are a very interesting group of immune-related proteins, as they have frequently been shown to have a dual function, being both involved in immune defense and digestion [38], [39]. One of the first lysozymes was identified in *Galleria mellonella* more than 40 years ago, representing the first antimicrobial protein reported from insects [40]. In addition to antibacterial activity, *G. mellonella* lysozyme was also shown to exhibit antifungal activity *in vitro*, similar to that of human lysozyme against the pathogenic yeast *Candida albicans* [41], [42]. A phylogenetic analysis of the Har salivary gland predicted lysozyme protein sequences revealed that they cluster in two distinct clades. One of these clades contains the C (chicken) type lysozymes [43], which includes three of the four Har lysozymes identified here (Figure 2). These findings are consistent with a typical number of C-type lysozymes found in other Lepidoptera (e.g. three lysozymes identified in the genome of *Bombyx*). To further examine the relationships among lysozyme proteins identified in Har salivary glands and those found in other insects, C-type lysozyme sequences from several insect species were aligned and used to construct a gene phylogeny (Figure 2A). The phylogenetic analysis revealed that the three Har and other lepidopteran C-type lysozyme sequences clustered in two distinct clades. One of these clades clearly separated with a high bootstrap support contains two of the three Har sequences and lepidopteran lysozymes generally associated with immune system functions, while another Har gland lysozyme clusters together with lysozymes identified in the gut of several Lepidoptera. This specific Har C-type lysozyme is 76% and 75% identical to the *Antherea mylitta* and *Manduca sexta* homologues, 42% identical to the salivary lysozyme homolog from a Diptera (*Simulium nigrimanum*), but displays only 35–39% identity to the immune-related C-type lysozymes from other Lepidoptera. The lysozyme sequence clustering displayed in the phylogeny can also be seen in the protein alignment, clearly separating two distinct groups of proteins (Figure 2B). In addition to the C-type, we identified one i-type-like lysozyme whose function remains to be elucidated. I-type lysozymes are vertebrate-specific and, although somewhat diverged in their activities, differ from other lysozymes in having 10–12 cysteine residues in the primary sequence. These cysteine residues are predicted to form five disulfide bonds which have been attributed to cause stability against heat denaturation or proteolytic degradation as i-type lysozymes can be intact even after prolonged heating [44]. The i-type lysozymes are typically coded for by single copy genes in Lepidoptera [45].

#### Proteinase inhibitors

**Figure 3:**
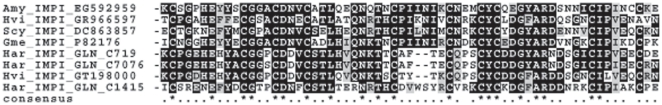
Alignment of IMPI proteins. Multiple sequence alignment of the conserved N-terminal parts coding for the complete mature metalloprotease inhibitor peptides (IMPIs) of *Helicoverpa armigera* (Har), *Galleria mellonella* (Gme), *Heliothis virescens* (Hvi), *Antheraea mylitta* (Amy), and *Samia cynthia* (Scy). Identical residues are boxed with dark shading, and conserved residues are boxed with light shading. Conserved residues are marked with dots and identical residues in all IMPIs are marked with asterisks below the alignment.

Har salivary gland transcriptome is also very rich in genes coding for proteinase inhibitors, such as immune-related proteases involved in immune defense regulation, and several Kazal-type proteinase inhibitors (KPIs) such as dipetalogastin/brasiliensin-like inhibitors [46], several of which are amongst the most highly expressed genes in our library (Table1). Proteinases and proteinase inhibitors are involved in several biological and physiological processes in all multicellular organisms and can act as modulators for controlling the extent of deleterious proteinase activity. The invertebrate KPIs which function as anticoagulants in blood-sucking animals such as leech, mosquitoes and ticks, are likely involved in protecting host from microbial proteinases and have been shown to protect silk moth cocoons from predators and microbial destruction [46]. The salivary gland transcriptome of Har comprises a number of serine proteinase inhibitors among which we identified three genes encoding potential metalloprotease inhibitors (Har_GLN-C719; C7076; C1415). All three insect metalloproteinase inhibitors (IMPIs) share sequence similarity only with an IMPI isolated from immune-induced *G. mellonella* larvae. This IMPI represents the first and to date only peptide known from animals which is capable of inhibiting thermolysin-like microbial metalloproteinases [37], including a number of prominent members such as bacillolysin and vibriolysin which are produced by pathogenic bacteria to invade the tissues of their hosts [47]. IMPI proteins have recently been found to encode two distinct inhibitors where the N-terminal part contributes to innate immune responses by inhibiting microbial metalloproteases, whereas the C-terminal part has been implicated to mediate regulation of endogenous immunity and development-related matrix metalloproteinases [48]. Two of the three Har IMPI cDNAs are truncated at the C-terminus but all three code for the complete N-terminal microbial metalloprotease inhibitor peptide, differing in several amino acid positions and thus pointing at the existence of a small IMPI gene family (Figure 3).

**Table 1:** Results of MS BLAST searches using de novo peptide sequences against the ButterflyBase Database and BLASTP searches using ButterflyBase protein sequences against UniRef100.

spot	ButterflyBase identification^a^	Length predicted polypeptide (aa)^b^	MS BLAST Score^c^	Peptides^d^	BLASTp vs Uniref100 (top hit)^e^			
					Description	species	Accession	E-value
2	STP00012_1	636	206	8	Ecdysone oxidase	*Spodoptera littoralis*	Q95NZ0	0
5	ni	-	-	-	-	*-*	-	-
6	BMP000597_1	331	280	7	oxidase/peroxidase	*Nasonia vitripennis*	UPI00015B588D	4,00E-48
7	PXP00618_1	188	207	4	Antennal esterase CXE3	*S. littoralis*	D3GDL4	5,00E-82
8	PXP00109_1	629	821	11	Heat shock cognate 70	*Plutella xylostella*	Q2WG65	0
15	ni	-	-	-	-	*-*	-	-
16	ni	-	-	-	-	*-*	-	-
20	SFP04769_1	244	362	7	Juvenile hormone binding protein	*Heliothis virescens*	Q25175	2,00E-88
22	AYP00014_1	164	193	3	Elongation factor 1 beta (Fragment)	*Antheraea yamamai*	Q6L609	1,00E-43
23	HMP00286_1	243	308	4	Proteasome subunit alpha type	*Bombyx mori*	Q2F661	e-128
24	ni	-	-	-	-	*-*	-	-
25	ni	-	-	-	-	*-*	-	-
26	SFP00311_3	175	389	4	Translationally-controlled tumor protein homolog	*B.mori*	Q75VN3	4,00E-91
27	MSP00420_1	193	63	1	Brasiliensin	*Triatoma brasiliensis*	A7YIH7	2,00E-32
28	SEP00040_1	160	115	2	Eukaryotic translation initiation factor 5A	*Spodoptera exigua*	P62924	1,00E-89
29	ni	-	-	-	-	*-*	-	-
31	TBP00299_1	111	160	2	Group 15 allergen protein	*Dermatophagoides pteronyssinus*	Q4JK70	2,00E-07
34	SFP11092_1	301	462	9	Hemolymph proteinase	*Manduca sexta*	Q5MPB6	8,00E-61
40	SFP04769_1	244	273	5	Juvenile hormone binding protein	*H. virescens*	Q25175	2,00E-88
43	LOP00661_1	177	328	5	Putative uncharacterized protein	*Branchiostoma floridae*	C3YWF0	7,00E-56
44	SFP01905_2	255	114	2	Phosphoglycerate mutase	*B. mori*	Q3S2I9	e-132
45	BMP020939_1	262	317	6	PREDICTED: similar to AGAP001957-PA	*Tribolium castaneum*	UPI0000D56B2F	e-122
47	ni	-	-	-	-	*-*	-	-
49	PIP01950_1	161	251	4	Actin-depolymerizing factor 1	*B. mori*	Q1HPP5	8,00E-79
50	HMP01674_3	151	403	6	Actin-depolymerizing factor 1	*B. mori*	Q1HPP5	1,00E-79
51	HEP01070_2	218	158	3	-	*-*	-	-
55	PIP04233_1	189	78	1	GF16309	*Drosophila ananassae*	B3LX08	8,00E-67
60	HAP00799_1	297	511	9	Glyceraldehyde-3-phosphate dehydrogenase	*B. mori*	Q1EPM0	e-152
61	HAP00799_1	297	461	6	Glyceraldehyde-3-phosphate dehydrogenase	*B. mori*	Q1EPM0	e-152
62	BMP006112_1	176	375	5	GTP-binding nuclear protein	*B. mori*	Q2F5M1	2,00E-97
64	TNP00255_1	167	287	4	Peptidyl-prolyl cis-trans isomerase	*B. mori*	Q5CCL3	2,00E-79
65	LOP00067_1	165	150	3	Peptidyl-prolyl cis-trans isomerase	*B. mori*	Q5CCL3	2,00E-79

a Cluster ID of best hit in ButterflyBase by MS BLAST (ni = not identified).

b Number of amino acids of predicted ButterflyBase protein.

c MS BLAST scoring.

d Number of peptides matching best hit in ButterflyBase in MS BLAST search.

e Results of blastp search using ButterflyBase predicted protein against UniRef100.

### Unknown transcripts with overlap to insect sialomes

A wide range of sequences identified in the Har salivary gland transcriptome display homology to predicted proteins that have been identified in salivary glands of aphids and mosquitoes but also in the venom glands of wasps. Among the overlapping cDNAs are sequences with homology to a 17 kDa salivary protein described in *Phlebotomus*, a putative 6.3 kDa salivary peptide *Anopheles funestus*, a putative secreted salivary protein from a flea *Xenopsylla cheopis*, several cDNAs with homology to an unknown salivary protein from a mosquito (*Culicoides sonorensis*), salivary cysteine-rich peptides of *B. mori*, a salivary/fat body serine carboxypeptidase identified in wheat midge *Sitodiplosis mosellana*, several cDNAs with similarity to venom acid phosphatases and a gamma-glutamyl cyclotransferase-like venom protein isoform 2 of *Nasonia vitripennis*, and several cDNAs with homology to secreted salivary ribonucleases. In general we can find a range of salivary-gland expressed genes which, based on their GO associations and predicted function overlap with the venom gland transcriptome of wasps [22]. These findings support the hypothesis that most insect sialomes share a core fraction of expressed genes related to potentially important functional categories such as oxidative stress response, immune defense, pre-digestion and/or tissue penetration, and proteins determining the viscosity of the saliva. A complete list of the contigs and singletons with their GO annotations and BLAST results can be found in Table S2.

### Characterization of secreted labial salivary proteins

**Figure 4:**
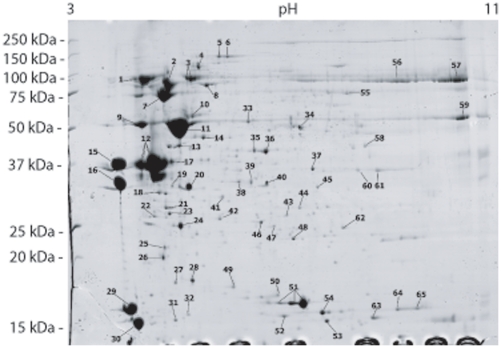
Two-dimensional SDS-Page map of *H. armigera* larval salivary gland proteins. Secretion-enriched proteins from *H. armigera* larval salivary (labial) were loaded on 24-cm pH 3 to 11 NL isoelectric focusing strips, separated in the second dimension by Tris–Tricine–SDS-polyacrylamide gel electrophoresis on a 15% gel and stained with colloidal Coomassie. Numbers designate the protein spots which were analyzed by mass spectrometry. Molecular mass standards are indicated in kDa (left) and the pI range at the top of the gel.

Obtaining enough labial saliva in order to undergo a proteomic analysis is a challenging task. Since collection of labial saliva through the spinneret (tube-like structure on the larval labium from where the silk is drawn) is a time-consuming impractical possibility, we decided to extract the labial gland pairs and subject them to a centrifugal force such that by compressing the organs towards the bottom of the tube, the supernatant obtained would be enriched on proteins from the gland lumen. Previously, secreted salivary proteins have been recovered in the supernatant using this approach [49]. The protein complexity of the non-cytosolic enriched soluble fraction from Har labial salivary glands is mostly represented by at least 20 proteins in the acidic pI range with an apparent molecular mass ranging from 25 to 150 kDa (Figure 4). A total of 65 gel spots were subjected to peptide de novo sequencing since they were considered of sufficient abundance (intensity) for subsequent MS analysis. The sequenced peptides from these spots yielded best protein hits from NCBI Insecta using MS-BLAST (Table S3) and only 24 hits when searched against ButterflyBase (http://butterflybase.ice.mpg.de/) (Table 1). Signal peptide probability was obtained for Har ESTs (positive hits) obtained after performing a MS-BLAST search of the peptides against Har salivary gland cDNA library translated into amino acid sequences. Indeed, the majority of more intensively stained and larger protein spots detected by 2-DE where predicted to have a high probability of being secreted enzymes (Table S4). Fewer abundant proteins between 15 to 25 kDa across the pI range were also detected. The majority of the remaining inconspicuous spots correspond to non-secreted proteins such as spots 23, 32 and 50, predicted to be involved in ubiquitin mediated proteolysis or glycolysis-related proteins (e.g. spots 47, 60) which indicates a degree of cytosolic protein contamination in the sample preparation. Similarly, the presence of the infection-inducible, hemolymph-clotting scolexin (spot 39), and arylphorin storage protein (spot 56, 57) may indicate a certain degree of contamination of our sample with hemolymph. However, salivary agglutinins may play a vital biological role by protecting the insect oral cavity from pathogens as observed in the case of humans [50]. The complete list of peptide sequences detected per protein spot and interpreted de novo from MS/MS spectra are available in Table S5.

### Identified secreted proteins

*Pre-digestion.* The extent of food digestion in the oral cavity of caterpillars previous to further processing and absorption in the gut is unknown. However, the feeding strategy of a phytophagous insect may indicate the importance of digestive enzymes as salivary components. Phytophagous piercing-sucking insects digesting the plant tissue in an extra-oral fashion may depend on a more complex battery of digestive enzymes including those required for the digestion of the plant cell wall. Indeed, peptides corresponding to predicted pectinases, cellulases or amylases in the labial salivary proteome of Har caterpillars were not detected, despite the identification of amylase sequences in the salivary gland transcriptome. However, the proteomic analysis did predict the presence of digestive enzymes such as β-fructofuranosidase (spots 9, 11), fructose-bisphosphate aldolase (spot 58), glucose dehydrogenase (spot 4) and proteases (spots 37, and 41).

It is necessary to contemplate these results taking in consideration the insect diet used. The commercial artificial diet (Bio-Serv) fed for the experimental group of insects is a sucrose, soy-wheat germ based diet without antibiotics. Therefore, whether the quality and quantity of Har labial gland proteome varies when a given host-plant is offered as food is still an open question. For now, the prediction of β-fructofuranosidase as a salivary secreted protein, specifically as predicted product of *GH32FruA-1* (EF600050), is consistent with the finding of this enzymatic activity in the labial glands of the related heliothine species *H. zea* [51] and β-fructofuranosidase *BmSuc1* expression and product localization in the labial glands of *B. mori* [52]. This protein has also been detected at relatively lower levels in the Har larval gut lumen [16]. Since the sucrose-digesting activity of recombinant BmSUC1 is not inhibited by the alkaloidal sugar mimic glycosidase inhibitors found in mulberry leaves, it has been suggested that this enzyme is an adaptation which allows the silkworm to bypass the mulberry's defense system. In addition, transcript *GH32FruA-1* is up-regulated in a tissue other than the gut upon detrimental gossypol concentrations to Har larval growth [53]. All this information opens intriguing questions about the role of β-fructofuranosidases in insect host-plant adaptation and the importance of defining whether there is a main organ of production of this type of enzymes. Fructose-bisphosphate aldolase is an important enzyme in fructose metabolism found, although not exclusively, in human salivary glands [3], [54] and glucose dehydrogenase, also a relevant enzyme in carbohydrate metabolism, is a protein that has been reported to be a component of the green peach aphid secreted saliva [8].

#### Herbivore offense

The identification of glucose oxidase (GOX) as a component of Har salivary proteome (spot 3) was consistent with previous reports revealing the ubiquity of this enzyme not only within the family Noctuidae but across Lepidoptera [55], [56], [57]. GOX occurrence in *Helicoverpa spp* labial glands has been correlated with the inhibition of plant defences (e. g. nicotine production in tobacco) [11], [56] and bacterial protection [12]. Moreover, the production of this enzymatic activity seems to be correlated with herbivore diet breadth. Thus, it has been suggested that GOX activity represents a potential mechanism contributing to host-range expansion in insect species [57]. The apparent multifunctional nature of GOX claims additional research, especially considering other components in caterpillar saliva, such as secreted antioxidant enzymes. We detected oxidase/peroxidase (spots 5, 6), superoxide dismutase (spot 53) and a putative secreted peroxiredoxin (spot 48) which potentially play a role in the removal of reactive oxygen species (ROS). Indeed, peroxidase activity has been found in the labial gland homogenate of a heliothine caterpillar species [58]. It has been claimed that GOX, along with enzymes able to eliminate hydrogen peroxide (a product of GOX) constitute an antioxidant system in insect physiology [55]. Another relatively abundant oxidase type, ecdysone oxidase (spots 1, 2), was also detected in the Har salivary gland secreted sialome. Ecdysteroids are involved in controlling different aspects of insect physiology such as moulting, development and reproduction, and in turn, one important player in their metabolism is ecdysone oxidase [59]. Further analysis on the function of this enzyme in labial saliva may indicate whether this protein is indeed involved in steroidal hormone metabolism.

Preventing plant defences to be triggered upon feeding may not be the only offense strategy of an insect herbivore, but detoxification of constitutive chemical defences in the host. A highly abundant protein corresponding to a carboxyl/cholinesterase (CCEO16d) was found represented by spot 7 on the 2D-gel. CCEO16d has been classified as an extracellular non-catalytic esterase and through comparative genomics, it has been grouped among dipteran CCEs involved in insecticide resistance [60]. Since there is evidence suggesting that esterases may not always hydrolyse their substrates [60], we speculate that labial salivary Har CCEO16d is an esterase involved in the modification and transport of host plant metabolites as a mechanism of insect defense.

Chymotrypsin inhibitor protein (spot 13) and brasiliensin (spot 27) were detected as elements of the secreted sialome in *H. armigera*. Brasiliensin is a multi domain serine protease inhibitor similar to other blood anticoagulants of blood-sucking insects. Termed after the hematophagous invertebrate *Triatoma brasiliensis*, its role in blood intake has recently been addressed confirming its anticoagulant activity [61]. Although anticoagulants have been mostly identified from blood-sucking invertebrates, other protease inhibitor-like proteins have been found in a seed-feeding hemipteran [20]. *H. armigera* brasiliensin-like protein raises the possibility of an additional insect response to a plant defense mechanism based on the increase of viscosity of the diet, interfering with insect digestion.

#### Arginine kinase and HaPUF-1

These two proteins are particularly interesting since both have previously also been detected in the *H. armigera* larval midgut lumen proteome [16]. The role of each of these enzymes in the insect midgut lumen is still unknown. Consistent with the midgut lumen 2D-gel protein separation results, arginine kinase, a human-allergenic enzyme, was detected also as two neighbor spots (35, 36) in our analysis. Recently, arginine kinase has been identified as a cytoplasmic protein which transcript is relatively abundant in different tissues of the silkworm, including the labial glands [62]. In addition, both arginine kinase transcript and protein are elevated in a silkworm strain resistant to nucleopolyhedrovirus in comparison to the susceptible one [62]. Comparing the intensity and magnitude of arginine kinase spots and HaPUF-1 (*H. armigera* protein of unknown function 1 or B1NLD7) in the midgut and the labial gland proteomes, HaPUF-1 (spot 12) represents a very abundant protein in the secreted labial salivary preparation while arginine kinase appears to occur at the same intensity in both the gut and labial gland. Further studies are required to determine whether there is a major organ of production of each of these proteins.

The main objective of this study was to evaluate the labial sialome (transcriptome and proteome) complexity of a lepidopteran herbivore and to identify a list of candidate genes and proteins likely to be involved in Har digestion and plant defense response manipulation. Indeed, the results herein represent additional evidence that Har labial glands are not simply a silk-producing organ but an overlooked important organ involved in insect immunity and digestion. In fact, a substantial number of the proteins found previously in Har gut have been identified as soluble luminal salivary proteins in this study, posing interesting questions regarding the mechanisms of insect digestive physiology. Therefore, the recurrence of such proteins claims a better understanding of their function. The insect mouth parts and oral cavity, as the first point of contact with the host, not only need to be protected from pathogens on the plant tissue but require offense molecules to counteract the plant chemical defense and enzymes that allow the acquisition of energy. The products of some Har labial salivary transcripts were not found in our proteomic analysis, such as amylases, lipases and some immune-related proteins. Reasons to explain this incongruence might be that such proteins were not detected in the soluble luminal protein preparation subjected to the proteomic analysis. AMPs, for example, are notoriously hard to detect in standard MS/MS analyses, mainly due to their small size which makes difficult their fragmentation and separation, and other proteins such as lipases may need even more stringent conditions to become denatured and solubilized. Other possible explanations are that these proteins are of low abundance, or that feeding induction studies may be necessary to allow such proteins to be detectable. We have generated a comprehensive tissue-specific database as a resource for more in-depth analyses of the salivary gland reprogramming of Har upon stress, most notably by toxic plant secondary metabolites. Furthermore, this data can be used for comparative genomics studies to identify overlap and differences among phytophagous and hematophagous insects and more specifically among generalist and specialist lepidopteran herbivores.

## Materials and Methods

### Insects and diet

Har eggs were acquired in 2008 from Bayer CropScience AG (Monheim, Germany) and reared under laboratory conditions (26°C, 55% RH, 16∶8 hr = L:D) in Jena, Germany since 2009, for about 10 generations prior to the start of this study. The artificial diet for larval rearing was purchased from BioServ (Cat. No. F9772, Frenchtown, NJ, USA).

### Sample preparation

Batches of second-day fifth-instar larvae were dissected longitudinally under ice-cold phosphate buffered saline (PBS) in order to retrieve with fine forceps the labial salivary gland apparatus (LG) which were collected in 1.5 ml tube containing 100 µl PBS. After centrifugation (16 000 g, 20 min. 4°C), the supernatant enriched with LG lumen soluble proteins was collected in a new tube and stored at −20°C until sample preparation for 2-D electrophoresis. Samples were pooled into two independent biological replicates each representing approximately 90 LG pairs and protein concentration was determined using the Protein Dye reagent (BioRad) and bovine serum albumin (BSA) as standard.

### Normalization and cDNA library construction

Har salivary (labial and mandibular) glands were isolated from 3rd to 5th instar larvae by microsurgery. Isolated glands were placed in pre-cooled 1.5 ml tubes with 1 ml TriZol, homogenized with a TissueLyser (Qiagen) and shock frozen in liquid nitrogen before RNA isolation. After RNA purification with TriZol, an additional DNAse (Turbo DNAse, Ambion) treatment was included prior to the second purification step to eliminate any contaminating DNA. The DNAse enzyme was removed and the RNA was further purified by using the RNeasy MinElute Clean up Kit (Qiagen) following the manufacturer's protocol and eluted in 20 µl of RNA Storage Solution (Ambion). RNA integrity and quantity was verified on an Agilent 2100 Bioanalyzer using the RNA Nano chips (Agilent Technologies, Palo Alto, CA). RNA quantity was determined on a Nanodrop ND-1000 spectrophotometer. RNA extractions were generated from different pooled glands and four RNA extracts were subsequently pooled for cDNA generation.

For Har salivary gland tissue material a full-length enriched, normalized cDNA library was generated using a combination of the SMART cDNA library construction kit (Clontech) and the Trimmer Direct cDNA normalization kit (Evrogen) generally following the manufacturer's protocol but with several important modifications, essentially as previously described [63]. Each step of the normalization procedure was carefully monitored to avoid the generation of artefacts and overcycling. The resulting ds-cDNA pool was purified and concentrated using the DNA Clean and Concentrator kit (Zymogen) and size fractionated with SizeSep 400 spun columns (GE Healthcare) that resulted in a cut-off at ∼200 bp. The full-length-enriched cDNAs were cut with *Sfi*I and ligated to pDNR-Lib plasmid (Clontech). Ligations were transformed into *E. coli* ELECTROMAX DH5α-E electro-competent cells (Invitrogen). Hemocyte and midgut Har cDNA libraries [16] were used along with Har salivary cDNA library to inspect GO enrichment among tissue-specific cDNA libraries.

### Sequencing, Generation of EST Databases and Sequence Analysis

Plasmid minipreparation from bacterial colonies grown in 96 deep-well plates was performed using the 96well robot plasmid isolation kit (NextTec) on a Tecan Evo Freedom 150 robotic platform (Tecan). Single-pass sequencing of the 5′ termini of cDNA libraries was carried out on an ABI 3730 xl automatic DNA sequencer (PE Applied Biosystems). Vector clipping, quality trimming and sequence assembly using stringent conditions (e.g. high quality sequence trimming parameters, 95% sequence identity cutoff, 25bp overlap) was done with the Lasergene software package (DNAStar Inc.). To identify similarities with known proteins, the sequences of contigs and singletons were searched using the BLASTX algorithm [64] against a local non-redundant protein database (NR, NCBI) with a E-value cut-off of 10^−04^. To define the function of the contigs and singletons, we used the Gene Ontology (GO) [65] controlled vocabulary, which provides annotations and allows a more global view of the dataset using the Blast2GO software with a stringency cut-off of 10^−3^. To minimize the number of classes with only few gene objects, we set the minimum number of gene objects (cut-off level) in a class to 0.5% of the total number of sequences that could be classified. The signalP algorithm was accessed online to predict the presence of signal peptides (SignalP 3.0 Server. [http://www.cbs.dtu.dk/services/SignalP]). The EST sequences were deposited into the NCBI dbEST database under accessions JK126269-JK145657.

### Phylogenetic reconstruction

Nucleotide sequences were analyzed in more detail using the commercial Lasergene Software package and the freeware BioEdit program. Genes were aligned by their amino acid sequences using the ClustalX2 function [66] or the MAFFT (http://mafft.cbrc.jp/alignment/server/index.html) program. If necessary, alignments were then corrected by eye and reverted back to the nucleotide sequences for the phylogenetic analyses and in order to remove redundant contigs. Conserved residues in the alignments were highlighted with BOXSHADE 3.21 (http://www.ch.embnet.org/software/BOX_form.html) or in ClustalX2. The phylogenetic reconstruction implemented for the analysis of several proteins was performed using two different methods, Maximum-Likelihood analyses using PhyML and by Bayesian inference using Mr. Bayes, both implemented in the Phylogeny.fr webserver (http://www.phylogeny.fr/version2_cgi/alacarte.cgi). The Maximum-Likelihood and the Bayesian tree topologies including their general subfamily relationships and node supports were in agreement. The gene trees were visualized and optimized with the TreeDyn tool also implemented on the Phylogeny.fr webserver.

### Separation of Proteins by Two-Dimensional Gel Electrophoresis

The protocol used in order to separate the enriched LG lumen protein samples by 2-D PAGE has been described previously [16] with the only modification of staining the gels with colloidal Coomassie working solution prepared following the protocol described elsewhere (http://www1.em.mpg.de/proteomics/) [67].

### Protein Spot Picking and Processing

The protein spots were manually picked and processed as described earlier [16] with the following modifications: trypsin digestion was carried out overnight with 70 ng of porcine trypsin (Promega) in 10* µ*L of 50 mM ammonium bicarbonate at 37°C. The digest was centrifuged down in MTPs and 50* µ*L of extraction solution (50% acetonitrile, 0.1% TFA) were added twice for 20 min extraction, and the solution was transferred to the plate. The extracted peptide mixtures were then vacuum-dried for approx. 45 min at 45°C.

#### Mass spectrometry (MS)

The tryptic peptides were reconstituted in 6 µL aqueous 0.1% formic acid (FA). The selected volume of samples (ca 4.5 µL) was injected on a nanoAcquity nanoUPLC system (Waters, Milford, MA, USA). Mobile phase A (0.1% aqueous formic acid, 15 µL/min for 1 min) was used to concentrate and desalt the samples on a 20×0.180 mm Symmetry C18, 5 µm particle precolumn. The samples were then eluted on a 100 mm×75 µm ID, 1.7 µm BEH nanoAcquity C18 column (Waters). Phases A and B (100% MeCN in 0.1% FA) were linearly mixed in a gradient to 5% phase B in 0.33 min, increased to 40% B in 10 min, and finally increased to 85% B in 10.5 min, holding 85%B to 11 min and decreasing to 1% B in 11.1 min of the run. The eluted peptides were transferred to the nano electrospray source of a Synapt HDMS tandem mass spectrometer (Waters) equipped with metal coated nanoelectrospray tips (Picotip, 50×0.36 mm, 10 µm I.D, New Objective, Woburn, MA, USA). The source temperature was set to 80°C, cone gas flow 20 L/h, and the nanoelectrospray voltage was 3.2 kV. The TOF analyzer was used in reflectron mode. The MS/MS spectra were collected at 1 s intervals (50–1700 m/z). A 650 fmol/µL human Glu-Fibrinopeptide B in 0.1% formic acid/acetonitrile (1∶1 v/v) was infused at a flow rate of 0.5 µL/min through the reference NanoLockSpray source every 30th scan compensating for mass shifts in the MS and MS/MS fragmentation mode.

#### Bioinformatics

The data were collected by MassLynx v4.1 software. ProteinLynx Global Server Browser v.2.3 software (both Waters) was used for baseline subtraction and smoothing, deisotoping, *de novo* peptide sequence identification. The *de novo* sequence characterization from collisionally induced (CID) MS/MS fragment spectra used peptide mass tolerance 0.03 Da mass deviation of precursor peptide masses, 1 possible missed cleavage, carbamidomethylation of cysteins, possible oxidation of methionines, and possible deamidation of asparagines and glutamines, respectively. Signal peptide prediction probabilities were obtained using SignalP 3.0 [68].

### MS blast

The procedure and its merits have been described by others [69]. In brief, sequences with ladder scores (percentage of expected y- and b-ions) exceeding 40% were used in a homology-based search strategy using the MS BLAST program. The MS-BLAST utilizes possibly redundant short peptide sequences for similarity searches in protein databases from organisms phylogenetically distant from the study species. All candidate sequences from a given spot exceeding the threshold, even different sequences from the same peptide, are concatenated into a single query separated by dashes in an arbitrary order. The WU-BLAST2 BLASTP search engine (http://blast.wustl.edu) scores only the most significant match in the case of several peptide candidates covering the same region in the target sequence. In addition, the PAM30MS matrix, which accounts for the inability to distinguish I and L residues and allows for unknown residues X, is used in the blastp similarity search. This enables identification of homologous proteins in other species with many amino acid substitutions, under conditions where spectral searches are not possible due to lack of sequences for the given organism. Scoring of the significance of such matches is on precomputed threshold scores conditional on the number of query peptides and their *E*- values of the individual HSPs (high-scoring segment pairs) hits. Computational studies [70] have estimated a false positive rate of <3%. The searches were performed on MS BLAST server installed in-house for searching the EBI_100-nr database and on a locally generated EST database from Har salivary gland cDNA library or on the ButterflyBase web page (http://butterflybase.org/) for searching the ButterflyBase EST database from Lepidoptera, exclusive of *B. mori* (34 882 protein sequences).

## Supporting Information

Figure S1 Gene ontology (GO) assignments for the *Helicoverpa* sialotranscriptome. GO assignments as predicted for their involvement in (A) biological processes and (B) molecular functions. Data for biological processes are presented at level 2 GO categorization while data for molecular functions are presented at level 3 GO categorization. Classified gene objects are depicted as percentages of the total number of gene objects with GO assignments.
(TIF)

Figure S2 Comparison of GO category representations between *Helicoverpa armigera* salivary gland, gut and hemocyte transcriptome data. Each transcript was assigned applicable high-level generic GO terms. Data are presented for Molecular Function GO-level 3. Obtained GO data for gut (GN) and hemocyte (HCN) tissues were multiplied by the factor depicted next to the abbreviations in order to correct for different numbers of total contigs obtained. Note that one gene object can be classified into more than 1 class, therefore the total number of gene objects classified for both species is not identical to the number of contigs with GO associations.
(TIF)

Table S1 Top highest expressed ESTs in salivary gland library.
(PDF)

Table S2 Complete annotation file of the assembled *Helicoverpa* salivary gland ESTs. Contig IDs, sequence length, Helicoverpa contig sequences, top BLAST hits (if any) in the NCBI nr database for each unique contig, including accession number, E-value and percentage similarity, EC numbers, GO annotations and InterPro scans are listed.
(XLS)

Table S3 Results of MS BLAST searches using de novo peptide sequences against the NCBI_insecta Database. ^a^GenBank Accession number and description of best hit protein in NCBI_insecta by MS BLAST. ^b^Species of best hit in NCBI-insecta. ^c^Predicted molecular weight of best hit (kDa). ^d^Number of peptides matching best hit in the MS BLAST search. ^e^MS BLAST scoring (see Material and Methods).
(PDF)

Table S4 Results of MS BLAST searches using de novo peptide sequences against *H. armigera* ESTs and salivary gland cDNA library sequences and BLASTP searches using *H. armigera* protein predicted from cDNA against UniRef100. ^a^Number of peptides matching the target in the MS BLAST search. ^b^Number of amino acids of predicted *H. armigera* protein.^ c^Predicted molecular weight of *H. armigera* protein (kDa). ^d^Predicted pI of *H. armigera* protein. ^e^Result of blastp search using *H. armigera* predicted protein against UniRef100. ^f^UniRef100 Accession Number. ^g^Species of best hit in UniRef100. ^h^E-value of best hit in blastp search again UniRef100. ^i^Signal peptide probability of *H. armigera* predicted protein.
(PDF)

Table S5 Identification obtained with the de novo sequenced peptides using MS BLAST. Peptide sequences interpreted de novo from MS/MS spectra were used to query NCBI_insecta, ButterflyBase, or *the H. armigera* EST database using the MS BLAST search engine.
(PDF)
