# Supplementary figures and images for: Sialome of a Generalist Lepidopteran Herbivore: Identification of Transcripts and Proteins from Helicoverpa armigera Labial Salivary Glands
Source: PLoS One. 2011 Oct 27;6(10):e26676. doi: 10.1371/journal.pone.0026676 (PMC3203145; doi:10.1371/journal.pone.0026676)

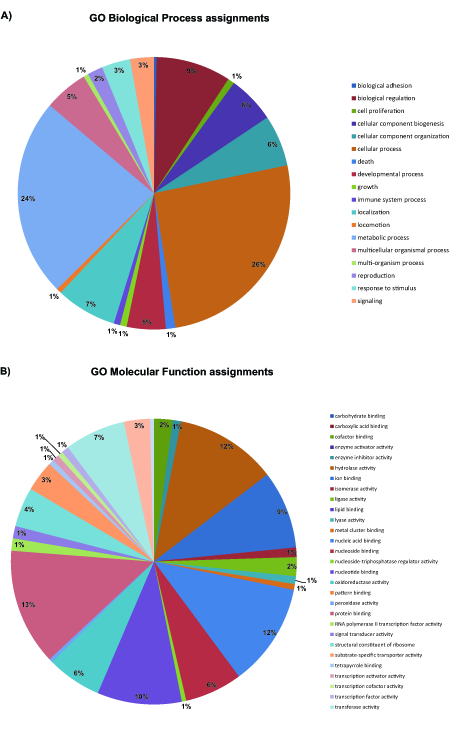

Supplement: Figure S1 — Gene ontology (GO) assignments for the Helicoverpa sialotranscriptome. GO assignments as predicted for their involvement in (A) biological processes and (B) molecular functions. Data for biological processes are presented at level 2 GO categorization while data for molecular functions are presented at level 3 GO categorization. Classified gene objects are depicted as percentages of the total number of gene objects with GO assignments. (TIF) [file pone.0026676.s001.tif]

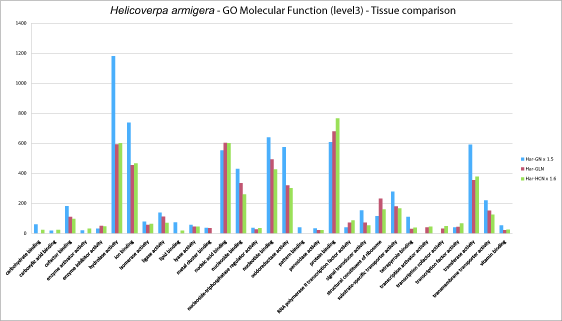

Supplement: Figure S2 — Comparison of GO category representations between Helicoverpa armigera salivary gland, gut and hemocyte transcriptome data. Each transcript was assigned applicable high-level generic GO terms. Data are presented for Molecular Function GO-level 3. Obtained GO data for gut (GN) and hemocyte (HCN) tissues were multiplied by the factor depicted next to the abbreviations in order to correct for different numbers of total contigs obtained. Note that one gene object can be classified into more than 1 class, therefore the total number of gene objects classified for both species is not identical to the number of contigs with GO associations. (TIF) [file pone.0026676.s002.tif]
