# Supplementary material for: Sialome of a Generalist Lepidopteran Herbivore: Identification of Transcripts and Proteins from Helicoverpa armigera Labial Salivary Glands
Source: PLoS One. 2011 Oct 27;6(10):e26676. doi: 10.1371/journal.pone.0026676 (PMC3203145; doi:10.1371/journal.pone.0026676)
Supplement: Table S1 — Top highest expressed ESTs in salivary gland library. (PDF) [file pone.0026676.s003.pdf]

Table 1. The 100 most abundant transcripts in the *Helicoverpa armigera* salivary gland transcriptome

Contig ID	Total no. of ESTs	Length (bp)	Description	Top BLAST hit (NR)		
				Accession	Organism	E-value
Har-GLN_Contig_166	485	1059	unknown protein [Helicoverpa armigera]	ABU98617	Helicoverpa armigera	4.56E-155
Har-GLN_Contig_721	228	1652	juvenile hormone epoxide hydrolase	ACM78602	Helicoverpa armigera	1.41E-133
Har-GLN_Contig_1578	141	1868	glucose dehydrogenase	ACL36977	Helicoverpa zea	0
Har-GLN_Contig_1229	68	779	elongation factor 1 alpha	BAG30769	Papilio xuthus	4.48E-134
Har-GLN_Contig_1740	50	3085	minor ampullate spidroin-like protein	ABR37276	Nephilengys cruentata	7.81E-126
Har-GLN_Contig_1068	48	807	hypothetical protein	XP_843140	Leishmania major	4.49E-07
Har-GLN_Contig_1935	46	1397	hepatic triacylglycerol lipase	ADA67928	Bombyx mori	3.34E-61
Har-GLN_Contig_1642	45	681	cytochrome c oxidase subunit ii	AAY54543	Helicoverpa armigera	4.37E-113
Har-GLN_Contig_639	44	770	ribosomal protein l32	Q962T1	Spodoptera frugiperda	4.03E-71
Har-GLN_Contig_949	43	1354	serine protease inhibitor dipetalogastin (brasiliensin)	ABI96910	Triatoma brasiliensis	1.00E-54
Har-GLN_Contig_858	41	1691	sucrose-6-phosphate hydrolase (fructosidase)	ABU98615	Helicoverpa armigera	0
Har-GLN_Contig_1974	40	755	autophagy related protein atg8	NP_001040244	Bombyx mori	3.63E-61
Har-GLN_Contig_339	38	874	signal peptidase 18 kda subunit	NP_001040280	Bombyx mori	1.47E-94
Har-GLN_Contig_248	36	2455	glucose oxidase	ACC94296	Helicoverpa armigera	0
Har-GLN_Contig_1343	34	844	ribosomal protein s6	Q95V32	Spodoptera frugiperda	3.86E-137
Har-GLN_Contig_362	33	541	ribosomal protein l24	Q962T5	Spodoptera frugiperda	1.71E-82
Har-GLN_Contig_676	32	547	ribosomal protein l22	AAK92160	Spodoptera frugiperda	1.83E-79
Har-GLN_Contig_1577	32	941	fibroin p25 (silk protein)	ACX50393	Corcyra cephalonica	3.89E-67
Har-GLN_Contig_2598	32	2504	pe-pgrs family protein	EFA85227	Polysphondylium pallidum	1.82E-61
Har-GLN_Contig_774	31	929	ribosomal protein l7	AAL62469	Spodoptera frugiperda	2.49E-143
Har-GLN_Contig_939	29	575	ribosomal protein s18	Q962R1	Spodoptera frugiperda	4.51E-82
Har-GLN_Contig_16	29	844	serf-like protein	XP_002428430	Pediculus humanus corporis	7.05E-14
Har-GLN_Contig_1995	29	505	ribosomal protein s17	NP_001037267	Spodoptera frugiperda	3.55E-70
Har-GLN_Contig_279	28	1355	lipase 3	XP_973187	Tribolium castaneum	3.67E-73
Har-GLN_Contig_733	28	525	ribosomal protein s24	Q962Q6	Spodoptera frugiperda	6.89E-70
Har-GLN_Contig_39	28	685	atp synthase delta mitochondrial	NP_001093091	Bombyx mori	1.03E-69
Har-GLN_Contig_1449	28	639	ribosomal protein l11	ACY95309	Manduca sexta	3.11E-107
Har-GLN_Contig_98	28	884	40s ribosomal protein s3a	Q95V35	Spodoptera frugiperda	2.77E-149
Har-GLN_Contig_1074	27	2677	copia-type polyprotein	BAA74713	Bombyx mori	1.94E-125
Har-GLN_Contig_238	27	1227	glucosamine-6-phosphate n-acetyltransferase	NP_001040128	Bombyx mori	8.93E-100
Har-GLN_Contig_2104	27	452	ribosomal protein l35	NP_001037241	Bombyx mori	6.27E-61
Har-GLN_Contig_2594	26	1359	ribosomal protein l3	AAL62468	Spodoptera frugiperda	0
Har-GLN_Contig_758	26	1140	bax inhibitor-1-like protein	NP_001091820	Bombyx mori	5.88E-111
Har-GLN_Contig_750	26	589	ribosomal protein l28	Q962T2	Spodoptera frugiperda	9.34E-70
Har-GLN_Contig_534	26	750	signal peptidase complex subunit 2	NP_001040214	Bombyx mori	1.83E-89
Har-GLN_Contig_2746	26	1089	serine protease inhibitor dipetalogastin (brasiliensin)	ABI96910	Triatoma brasiliensis	1.39E-29
Har-GLN_Contig_1644	26	669	ribosomal protein l18a	Q8WQI7	Spodoptera frugiperda	2.91E-98
Har-GLN_Contig_1291	26	1292	nuclear excison repair protein rad23 homolog a	NP_001164652	Bombyx mori	4.38E-137
Har-GLN_Contig_409	26	1530	cytochrome oxidase subunit i	ABH07885	Feltia jaculifera	0
Har-GLN_Contig_550	25	2001	oxidase peroxidase	XP_311448	Anopheles gambiae	2.46E-66
Har-GLN_Contig_640	25	837	translationally controlled tumor protein	NP_001037572	Bombyx mori	1.33E-89
Har-GLN_Contig_214	25	982	selenoprotein m	XP_001600851	Nasonia vitripennis	1.60E-18
Har-GLN_Contig_857	25	1641	sucrose-6-phosphate hydrolase (fructosidase)	ABU98615	Helicoverpa armigera	0
Har-GLN_Contig_628	25	1409	mitochondrial porin	XP_967480	Tribolium castaneum	2.92E-105
Har-GLN_Contig_1765	24	1389	zinc transporter (catsup protein)	XP_001849361	Culex quinquefasciatus	2.59E-106
Har-GLN_Contig_1168	24	657	ribosomal protein l12	AAL26576	Spodoptera frugiperda	4.17E-86
Har-GLN_Contig_1427	24	716	ribosomal protein l19	ACY95336	Manduca sexta	7.95E-108
Har-GLN_Contig_234	22	590	ribosomal protein l26e	AAK92162	Spodoptera frugiperda	4.63E-77
Har-GLN_Contig_1245	22	537	ribosomal protein l27	ABS57452	Heliconius melpomene	3.16E-73
Har-GLN_Contig_1	22	810	cytochrome oxidase subunit iii	ABN04114	Spodoptera exigua	5.17E-120
Har-GLN_Contig_847	22	2333	protein disulfide isomerase	NP_001037171	Bombyx mori	0
Har-GLN_Contig_2788	22	500	ribosomal protein l23	NP_001037227	Bombyx mori	1.92E-73
Har-GLN_Contig_918	22	861	coagulin	BAH70732	Acyrthosiphon pisum	4.45E-11
Har-GLN_Contig_622	22	865	ribosomal protein l8	NP_001037141	Bombyx mori	8.68E-148
Har-GLN_Contig_1407	21	597	promoting protein	NP_001037199	Bombyx mori	4.16E-20
Har-GLN_Contig_1414	21	912	proteasome beta subunit	ACJ13433	Helicoverpa armigera	3.07E-130
Har-GLN_Contig_222	21	804	ribosomal protein l13	AAV91770	Helicoverpa zea	4.99E-123
Har-GLN_Contig_883	21	1307	glyceraldehyde-3-phosphate dehydrogenase	BAE96011	Bombyx mori	1.62E-179
Har-GLN_Contig_742	21	2104	glucosidase 2 subunit beta	XP_974655	Tribolium castaneum	1.60E-148
Har-GLN_Contig_863	21	927	eukaryotic translation initiation factor 1a	NP_001093083	Bombyx mori	1.07E-77
Har-GLN_Contig_2804	21	1855	protein disulfide-isomerase like protein erp57	BAD93613	Bombyx mori	0
Har-GLN_Contig_2651	21	770	nadh:ubiquinone dehydrogenase	XP_001663929	Aedes aegypti	7.89E-59
Har-GLN_Contig_511	21	844	no hit	na	na	na
Har-GLN_Contig_123	20	936	ribosomal protein l7a	ABX55885	Spodoptera exigua	4.93E-147
Har-GLN_Contig_2631	20	618	ribosomal protein s19	AAK92188	Spodoptera frugiperda	2.96E-80
Har-GLN_Contig_932	20	2025	ac1147-like protein	NP_001020173	Rattus norvegicus	1.03E-36
Har-GLN_Contig_508	20	645	immune reactive putative protease inhibitor (cysteine-rich venom)	ABK29470	Helicoverpa armigera	2.84E-39
Har-GLN_Contig_966	20	747	transmembrane emp24 protein (glycoprotein 25l)	NP_001040538	Bombyx mori	6.08E-109
Har-GLN_Contig_790	20	741	ribosomal protein l10a	Q963B6	Spodoptera frugiperda	6.37E-119
Har-GLN_Contig_1039	20	614	ribosomal protein s27	AAK92195	Spodoptera frugiperda	2.60E-44
Har-GLN_Contig_630	20	741	ribosomal protein l24	ACY95333	Manduca sexta	4.15E-102
Har-GLN_Contig_994	20	621	ribosomal protein s23	Q6EV23	Spodoptera frugiperda	3.67E-78
Har-GLN_Contig_2670	20	974	nascent polypeptide associated complex protein alpha subunit	NP_001040365	Bombyx mori	1.59E-103
Har-GLN_Contig_948	20	1304	serine protease inhibitor dipetalogastin (brasiliensin)	ABI96910	Triatoma brasiliensis	1.46E-55
Har-GLN_Contig_390	20	790	cyclophilin a	BAD90848	Bombyx mori	1.46E-79
Har-GLN_Contig_2044	20	1265	putative secreted protein	ABI52743	Argas monolakensis	1.41E-23
Har-GLN_Contig_625	20	808	succinate dehydrogenase	XP_971814	Tribolium castaneum	3.53E-28
Har-GLN_Contig_2312	20	1331	farnesoic acid o-methyltransferase-like	XP_974395	Tribolium castaneum	6.38E-30
Har-GLN_Contig_2611	19	648	eukaryotic translation initiation factor 6	ABB92838	Spodoptera frugiperda	3.57E-98
Har-GLN_Contig_18	19	1112	metallothionein b	XP_002013344	Drosophila persiminis	5.49E-05
Har-GLN_Contig_813	19	1032	ribosomal protein l5	ACY95338	Manduca sexta	8.86E-164
Har-GLN_Contig_355	19	1012	tgf beta-inducible nuclear protein 1	ABD36171	Bombyx mori	2.19E-143
Har-GLN_Contig_1337	19	914	endopeptidase inhibitor-like	CBH09269	Heliconius melpomene	4.06E-82
Har-GLN_Contig_1863	19	1756	calreticulin	BAC57964	Bombyx mori	0
Har-GLN_Contig_404	19	757	Yipf6 protein	NP_001040374	Bombyx mori	2.26E-111
Har-GLN_Contig_787	19	2031	dentin sialophosphoprotein	ACN91278	Canis lupus	6.01E-44
Har-GLN_Contig_1774	19	2051	putative ecdysone oxidase (glucose dehydrogenase)	CBH09301	Heliconius melpomene	1.86E-93
Har-GLN_Contig_1782	19	617	ribosomal protein s27a	AAL62473	Spodoptera frugiperda	1.38E-85
Har-GLN_Contig_1443	19	1949	antennal esterase CXE3	ACV60230	Spodoptera littoralis	3.98E-130
Har-GLN_Contig_343	18	828	chemosensory protein	ACX53817	Heliothis virescens	1.61E-47
Har-GLN_Contig_2741	18	2124	argonaute 2	NP_001036995	Bombyx mori	7.46E-69
Har-GLN_Contig_1527	18	662	sec61 beta subunit	NP_001037632	Bombyx mori	8.91E-44
Har-GLN_Contig_1148	18	896	histone deacetylase	XP_002427086	Pediculus humanis corporis	6.56E-29
Har-GLN_Contig_1613	18	873	mki67 (fha domain) interacting nucleolar phosphoprotein	NP_001037642	Bombyx mori	8.66E-71
Har-GLN_Contig_1741	18	577	ribosomal protein s13	Q962R6	Spodoptera frugiperda	3.28E-80
Har-GLN_Contig_1235	18	574	ribosomal protein l36	AAK92170	Spodoptera frugiperda	9.77E-61
Har-GLN_Contig_1683	18	747	cuticular protein analogous to peritrophins 3-e	XP_001603894	Nasonia vitripennis	3.49E-64
Har-GLN_Contig_1705	17	839	proteasome subunit beta type-6 precursor	XP_973571	Tribolium castaneum	5.74E-101
Har-GLN_Contig_953	17	877	ribosomal protein s2	AAN86048	Spodoptera frugiperda	2.74E-149
Har-GLN_Contig_1744	17	1287	oxidase/peroxidase	XP_001657372	Aedes aegypti	4.95E-48
