# Supplementary material for: Sialome of a Generalist Lepidopteran Herbivore: Identification of Transcripts and Proteins from Helicoverpa armigera Labial Salivary Glands
Source: PLoS One. 2011 Oct 27;6(10):e26676. doi: 10.1371/journal.pone.0026676 (PMC3203145; doi:10.1371/journal.pone.0026676)
Supplement: Table S3 — Results of MS BLAST searches using de novo peptide sequences against the NCBI_insecta Database. aGenBank Accession number and description of best hit protein in NCBI_insecta by MS BLAST. bSpecies of best hit in NCBI-insecta. cPredicted molecular weight of best hit (kDa). dNumber of peptides matching best hit in the MS BLAST search. eMS BLAST scoring (see Material and Methods). (PDF) [file pone.0026676.s005.pdf]

spot	GenBank accession[a]	Description[a]	organism[b]	predicted MW[c]	peptide hits MS blast[d]	MS BLAST score[e]
1	ACL36977.1	putative ecdysone oxidase	*Helicoverpa zea*	63,8	3	181
2	EDV38464.1	GF19436	*Drosophila ananassae*	132,8	8	431
3	ACC94296.1	glucose oxidase-like enzyme	*Helicoverpa armigera*	66,9	19	1152
4	BAI49425.1	neuroglian	*Mythimna separata*	140	7	375
5	XP_001603338.1	PREDICTED: hypothetical protein	*Nasonia vitripennis*	76,1	1	67
6	XP_001946039.1	PREDICTED: similar to AGAP008350-PA	*Acyrthosiphon pisum*	154,1	7	297
7	XP_311557.4	AGAP010390-PA	*Anopheles gambiae str. PEST*	81,9	3	276
8	XP_002031106.1	GM24208	*Drosophila sechellia*	71	13	884
9	ABU98615.1	fructosidase	*H. armigera*	54	10	712
10	ACI32835.1	serine proteinase-like protein 1	*H. armigera*	46,5	5	388
11	ABU98615.1	Fructosidase	*H. armigera*	54	11	693
12	ABU98617.1	unknown	*H. armigera*	29,9	6	391
13	ABV74209.1	serpin-6	*Bombyx mori*	46,4	5	323
14	ABW03226.1	beta actin	*Xestia c-nigrum*	41,8	7	442
15	EAL40833.3	AGAP002826-PA	*A. gambiae str. PEST*	23,2	1	67
16	ACT80221.1	ATP synthase F0 subunit 6	*Mayetiola destructor*	25,8	3	126
17	AAK52495.1	AF361483_1 chymotrypsin inhibitor CI-8A	*B. mori*	43,8	5	292
18	BAG38534.1	14-3-3 epsilon	*B. mori*	29,6	6	398
19	AAA68242.1	juvenile hormone binding protein	*Heliothis virescens*	26,8	4	224
20	EDS42418.1	conserved hypothetical protein	*D. erecta*	16,8	9	301
21	ACR07788.1	14-3-3 zeta	*H. virescens*	28	12	796
22	XP_973769.1	PREDICTED: similar to Elongation factor 1 beta CG6341-PA	*Tribolium castaneum*	29,2	4	221
23	XP_973117.1	PREDICTED: similar to proteasome zeta subunit	*T. castaneum*	26,4	2	198
24	XP_002429704.1	glucose dehydrogenase, putative	*Pediculus humanus corporis*	73,7	2	121
25	XP_001943976.1	PREDICTED: similar to viral A-type	*A. pisum*	199,8	4	179
26	XP_395299.2	PREDICTED: similar to Translationally controlled tumor protein CG4800-PA isoform 1	*Apis mellifera*	19,7	4	339
27	AAB32409.1	18 kda serine proteinase inhibitor	*H. zea*	3	1	63
28	EAT44854.1	initiation factor 5a	*Aedes aegypti*	17,5	2	115
29	EDW67351.1	GJ23129	*Drosophila virilis*	110,4	1	68
30	ABU98615.2	Fructosidase	*H. armigera*	54	1	68
31	P82171.1	CU095_LOCMI RecName: Full=Cuticle protein 9.5;	*Locusta migratoria*	9,5	5	187
32	ABB04499.1	SUMO	*B. mori*	10,3	2	112
33	ABC96694.1	yellow-d	*B. mori*	50,3	4	503
34	XP_001649322.1	proacrosin, putative	*A. aegypti*	42	4	212
35	ABX57148.1	arginine kinase	*Archiearis parthenias*	22,6	16	822
36	ABX57154.1	arginine kinase	*Macrosoma tipulata*	22,6	7	445
37	ACI32835.1	serine proteinase-like protein 1	*H. armigera*	46,5	9	725
38	CAC88582.1	unnamed protein product	*H. virescens*	32	8	562
39	C1JE15.1	SCLXB_HELVI RecName: Full=Scolexin B; Flags:	*H. virescens*	30,5	6	306
40	EDW90053.1	GE13058	*Drosophila yakuba*	62	2	113
41	NP_001155191.1	silk gland derived serine protease	*B. mori*	42	6	276
42	ABM90638.1	6-phosphogluconolactonase	*B. mori*	25,9	3	182
43	XP_002007655.1	GI12260	*Drosophila mojavensis*	29	3	148
44	XP_002093710.1	GE20611	*D. yakuba*	43,8	6	265
45	XP_968982.1	PREDICTED: similar to AGAP001957-PA	*T. castaneum*	31,3	6	311
46	CAA72953.1	diverged serine protease	*H. armigera*	27,3	2	171
47	ACV89326.1	triosephosphate isomerase	*Nigronia sp.*	17,5	2	121
48	ABW96360.1	thioredoxin peroxidase	*H. armigera*	21,9	6	521
49	XP_001866830.1	conserved hypothetical protein	*Culex pipiens*	19	5	279
50	AAR09835.1	similar to Drosophila melanogaster tsr	*D. yakuba*	17,1	7	443
51	XP_001985637.1	GH17180	*Drosophila grimshawi*	207,9	2	160
52	NP_001093087.1	mobility group protein 1B	*B. mori*	13,3	1	95
53	XP_394370.1	PREDICTED: similar to CG5255-PA	*Apis mellifera*	28	2	113
54	AAM53644.1	abnormal wing disc-like protein	*Choristoneura parallela*	17	8	574
55	EDV53781.1	GG11332	*Drosophila erecta*	63,2	1	86
56	CAB55605.1	arylphorin subunit	*Spodoptera litura*	84,1	6	355
57	CAB55605.1	arylphorin subunit	*S. litura*	84,1	7	416
58	BAD12426.1	fructose 1,6-bisphosphate aldolase	*Antheraea yamamai*	39,7	10	660
59	ABU98615.1	fructosidase	*H. armigera*	54	4	270
60	AAU95199.1	putative glyceraldehyde-3-phosphate dehydrogenase	*Oncometopia nigricans*	35,6	7	399
61	ABV60323.1	putative glyceraldehyde-3-phosphate dehydrogenase	*Lutzomyia longipalpis*	35,2	7	485
62	XP_002423913.1	GTP-binding nuclear protein RAN1, putative	*Pediculus humanus corporis*	49,7	5	351
63	ABK29488.1	promoting protein	*H. armigera*	8,4	2	146
64	XP_001119897.1	PREDICTED: similar to Cyclophilin 1	*A. mellifera*	17,8	6	305
65	ACD69576.1	peptidyl-prolyl isomerase-1	*Gryllus pennsylvanicus*	17,9	3	149
