# Supplementary material for: Sialome of a Generalist Lepidopteran Herbivore: Identification of Transcripts and Proteins from Helicoverpa armigera Labial Salivary Glands
Source: PLoS One. 2011 Oct 27;6(10):e26676. doi: 10.1371/journal.pone.0026676 (PMC3203145; doi:10.1371/journal.pone.0026676)
Supplement: Table S4 — Results of MS BLAST searches using de novo peptide sequences against H. armigera ESTs and salivary gland cDNA library sequences and BLASTP searches using H. armigera protein predicted from cDNA against UniRef100. aNumber of peptides matching the target in the MS BLAST search. bNumber of amino acids of predicted H. armigera protein. cPredicted molecular weight of H. armigera protein (kDa). dPredicted pI of H. armigera protein. eResult of blastp search using H. armigera predicted protein against UniRef100. fUniRef100 Accession Number. gSpecies of best hit in UniRef100. hE-value of best hit in blastp search again UniRef100. iSignal peptide probability of H. armigera predicted protein. (PDF) [file pone.0026676.s006.pdf]

spot	hits[a]	aa[b]	MW[c]	pI[d]	blastp vs UniRef100[e]	UniRef100[f]	species[g]	E-value[h]	Signal peptide probability[i]
1	4	581	63283	4,87	Putative ecdysone oxidase	B8Y866	*Heliothis zea*	0.0	0,525
2	11	213	23353,09	4,92	Putative ecdysone oxidase	B8Y866	*Heliothis zea*	3 e-26	0,002
3	10	278	31006,38	5,26	Glucose oxidase-like enzyme	B2MW81	*Helicoverpa armigera*	1 e-161	0,001
4	3	77	8838,99	5,81	Glucose dehydrogenase	Q17DW2	*Aedes aegypti*	1 e-5	0
5	4	244	27755,13	4,78	Oxidase/peroxidase	Q177U0	*Aedes aegypti*	1 e-34	0
6	6	244	27755,13	4,78	Oxidase/peroxidase	Q177U0	*Aedes aegypti*	1 e-34	0
7	7	372	42093,05	4,87	Antennal esterase CXE3	D3GDL4	Spodoptera littoralis	7 e-76	0
8	6	170	18693,57	7,68	Heat shock cognate 70 protein	Q0MUU7	Trichoplusia ni	7 e-89	0,983
9	11	366	41117,33	4,91	Fructosidase	B1NLD5	*Helicoverpa armigera*	0.0	0,98
10	9	458	51656,67	5	Juvenile hormone epoxide hydrolase	C0KH33	*Helicoverpa armigera*	1 e-132	0,98
11	12	458	51656,67	5	Fructosidase	B1NLD5	*Helicoverpa armigera*	0.0	0,98
12	5	300	33463,69	4,5	Putative uncharacterized protein	B1NLD7	*Helicoverpa armigera*	1 e-155	0,999
13	5	154	17156,75	5,34	Chymotrypsin inhibitor CI-8A	Q967V9	*Bombyx mori*	2 e-56	0,001
14	5	359	39854,99	5,29	Actin-A3b, cytoplasmic	P84184	*Helicoverpa armigera*	0.0	0
15	4	275	30755,66	7,81	Ubiquitin MGC53081 protein	Q7ZXG5	*Xenopus laevis*	1 e-146	0,003
16	2	158	18399,78	10,81	Predicted protein	A7SI41	Nematostella vectensis	0,004	0,527
17	3	300	33463,69	4,5	Putative uncharacterized protein	B1NLD7	*Helicoverpa armigera*	1 e-155	0,999
18	3	197	21414,04	4,72	Elongation factor 1 delta	Q9BPS1	*Bombyx mori*	8 e-76	0
19	4	458	51656,67	5	Fructosidase	B1NLD5	*Helicoverpa armigera*	0.0	0,98
20	9	242	26991,42	5,28	Juvenile hormone binding protein	Q25175	*Heliothis virescens*	5 e-80	0,997
21	12	247	28071,68	4,84	14-3-3 zeta protein domain specific binding	C4NFK6	*Heliothis virescens*	1 e-135	0
22	4	223	24574,59	4,52	Elongation factor 1 beta'	A9LSA6	*Spodoptera exigua*	1 e-116	0
23	2	160	17624,24	4,76	Proteasome subunit alpha type	Q2F661	*Bombyx mori*	1 e-84	0
24	1	213	23353,09	4,92	Putative ecdysone oxidase	B8Y866	*Heliothis zea*	3 e-26	0,002
25	5	54	6160,88	4,9	ni	–	–	–	0
26	4	172	19761,68	4,71	Translationally-controlled tumor protein homolog	Q75VN3	*Bombyx mori*	4 e-90	0
27	1	178	19137,96	5,16	Brasiliensin protease inhibitor	A7YIH7	*Triatoma brasiliensis*	3 e-30	1,00
28	2	160	17524,84	5,16	Eukaryotic translation initiation factor 5A	P62925	*Spodoptera frugiperda*	1 e-89	0
29	2	–	–	–	ni	–	–	–	–
30	1	458	45626,95	4,17	Dentin sialophosphoprotein	C9W8D1	*Odocoileus virginianus*	8 e-30	1,00
31	3	–	–	–	ni	–	–	–	–
32	2	91	10295,64	5,29	Small ubiquitin-like modifier proteinUbiquitin-like protein SMT3	Q2VEW6	*Bombyx mori*	1 e-43	0
33	8	233	26374,86	6,59	Yellow-d storage protein	Q2HZG6	*Bombyx mori*	3 e-98	0,001
34	1	–	–	–	ni	–	–	–	–
35	11	226	25793,39	6,09	Arginine kinase	D3Y4D1	*Helicoverpa armigera*	1 e-129	0
36	7	226	25793,39	6,09	Arginine kinase	D3Y4D1	*Helicoverpa armigera*	1 e-129	0
37	2	183	19606,31	4,79	Serine proteinase-like protein 1	B7SVM2	*Helicoverpa armigera*	1 e-105	0,995
38	4	242	26580,18	5,7	Juvenile hormone binding protein	Q25175	*Heliothis virescens*	1 e-106	0,998
39	7	257	27377,98	5,61	Scolexin B serine endopeptidase	C1JE15	*Heliothis virescens*	1 e-134	1,00
40	8	242	26580,18	5,7	Juvenile hormone binding protein	Q25175	*Heliothis virescens*	1 e-106	0,998
41	5	394	42816,81	7,35	Trypsin-like proteinase T2a	Q6R559	Ostrinia nubilalis	1 e-145	1,00
42	4	214	23732,44	4,92	6-phosphogluconolactonase	A2TF14	*Bombyx mori*	3 e-75	0
43	4	156	17435,3	8,4	ACYPI005456 protein	C4WU27	Acyrthosiphon pisum	2 e-45	0,117
44	2	255	28758,93	5,93	Phosphoglycerate mutase	Q3S2I9	*Bombyx mori*	1 e-136	0
45	3	455	49557,89	9,42	Hypothetical conserved protein	D3TQ61	Glossina morsitans morsita	5 e-40	0,951
46	1	173	19702,18	5,32	Heat shock protein hsp21.4	Q5R1P5	*Bombyx mori*	2 e-91	0
47	3	234	24849,46	5,98	Triosephosphate isomerase	Q5XUN5	*Helicoverpa armigera*	1 e-130	0
48	4	207	23125,65	6,2	Putative peroxiredoxin	A8CWH8	*Lutzomyia longipalpis*	5 e-84	0,97
49	4	148	17023,36	6,17	Actin-depolymerizing factor 1Actin-depolymerizing factor 4	Q1HPP5	*Bombyx mori*	1 e-78	0
50	6	145	16619,02	6,75	Ubiquitin carrier protein	Q2Q469	Bombyx mori	8 e-79	0
51	2	458	51656,67	5	Fructosidase	B1NLD5	*Helicoverpa armigera*	0.0	0,98
52	1	119	13292,25	8,58	Mobility group protein 1B	Q1HQ86	*Bombyx mori*	2 e-57	0,003
53	1	153	15605,48	6,17	Superoxide dismutase [Cu-Zn]	C3RSN1	Spodoptera exigua	2 e-69	0,001
54	8	176	19790,12	9,01	Nucleoside diphosphate kinase	Q8MUR5	*Choristoneura parallela*	4 e-80	0,849
55	1	65	7185,33	7,71	GL27078 Putative oxidoreductase activity	B4HAK7	Drosophila persimilis	8 e-5	0
56	6	354	42077,92	8,93	Arylphorin subunit	Q9U5K4	Spodoptera litura	1 e-147	1,00
57	?	335	39874,08	5,7	Arylphorin subunit	Q9U5K4	Spodoptera litura	1 e-165	0
58	10	364	39427,81	7	Fructose-bisphosphate aldolase	Q75PQ3	*Antheraea yamamai*	0.0	0
59	5	366	41117,33	4,91	Fructosidase	B1NLD5	*Helicoverpa armigera*	0.0	0
60	9	332	35554,64	7,71	Glyceraldehyde-3-phosphate dehydrogenase	Q1EPM0	*Bombyx mori*	1 e-179	0,001
61	7	332	35554,64	7,71	Glyceraldehyde-3-phosphate dehydrogenase	Q1EPM0	*Bombyx mori*	1 e-179	0,001
62	4	213	24406,48	6,96	GTP-binding nuclear protein Ran	D2CVL0	*Helicoverpa armigera*	1 e-122	0,002
63	2	134	14570,82	8,48	Promoting protein	B6A8I6	*Helicoverpa armigera*	2 e-36	0,999
64	5	165	17716,09	8,45	Peptidyl-prolyl cis-trans isomerase	Q5CCL3	*Bombyx mori*	4 e-80	0,002
65	2	165	17716,09	8,45	Peptidyl-prolyl cis-trans isomerase	Q5CCL4	*Bombyx mori*	5 e-80	1
