# Supplementary material for: Sialome of a Generalist Lepidopteran Herbivore: Identification of Transcripts and Proteins from Helicoverpa armigera Labial Salivary Glands
Source: PLoS One. 2011 Oct 27;6(10):e26676. doi: 10.1371/journal.pone.0026676 (PMC3203145; doi:10.1371/journal.pone.0026676)
Supplement: Table S5 — Identification obtained with the de novo sequenced peptides using MS BLAST. Peptide sequences interpreted de novo from MS/MS spectra were used to query NCBI_insecta, ButterflyBase, or the H. armigera EST database using the MS BLAST search engine. (PDF) [file pone.0026676.s007.pdf]

Spot 1

m/z	Charge	Peak mW	Peptide mW	Delta (Da)	Delta (ppm)	Ladder Score	Log Likelihood	Start	End	Sequence	Modifications	Subs	Query Tool
846,389	2	1690,7624	1690,7872	-0,0248	-14,6819	48,3871	182,7959	1	16	SYGFNAMGSVVNSTLK	Oxidation M / Deamidation	7 / 12	De-novo
846,389	2	1690,7624	1690,7872	-0,0248	-14,6819	48,3871	182,0285	1	16	SYGFNAMGSVVDSTLK	Oxidation M	7	De-novo
846,389	2	1690,7624	1690,7543	0,0081	4,7766	50,5376	179,4009	1	16	SYGTCAMGSVVDSTLK	Carbamidomethyl C /	5 / 7	De-novo
706,3473	2	1410,6790	1410,6848	-0,0058	-4,1285	73,3333	337,1761	1	13	NTLCMALSTTATK	Carbamidomethyl C	4	De-novo
706,3473	2	1410,6790	1410,6848	-0,0058	-4,1285	73,3333	330,2192	1	13	QSLCMALSTTATK	Carbamidomethyl C	4	De-novo
706,3473	2	1410,6790	1410,6848	-0,0058	-4,1285	70,6667	332,7321	1	13	TNLCMALSTTATK	Carbamidomethyl C	4	De-novo
838,3864	2	1674,7572	1674,7594	-0,0022	-1,3280	51,6129	176,1439	1	16	SASYCAMGSVVDSTLK	Carbamidomethyl C	5	De-novo
838,3864	2	1674,7572	1674,7594	-0,0022	-1,3280	45,1613	169,1143	1	16	SASYCAMGVSVDSTLK	Carbamidomethyl C	5	De-novo
838,3864	2	1674,7572	1674,7594	-0,0022	-1,3280	44,8276	165,537	1	15	SASYCAMKDVDSTLK	Carbamidomethyl C	5	De-novo
900,9174	2	1799,8192	1799,8955	-0,0763	-42,4047	41,9048	107,79	1	18	SNLGYYALGGFAAANVGR			De-novo
900,9174	2	1799,8192	1799,8914	-0,0722	-40,1269	48,5714	108,8603	1	18	SDGLNPNANLFAAANVGR			De-novo
900,9174	2	1799,8192	1799,8954	-0,0762	-42,3491	46,4646	105,0974	1	17	SNLGYYANLFAAANVGR			De-novo
892,9011	2	1783,7866	1783,8311	-0,0445	-24,9598	68,6869	366,633	1	17	SYGGNLCNLFAAANVGR	Deamidation N /	5 / 7	De-novo
892,9011	2	1783,7866	1783,8311	-0,0445	-24,9598	68,6869	365,9174	1	17	YSGGNLCNLFAAANVGR	Deamidation N /	5 / 7	De-novo
892,9011	2	1783,7866	1783,8311	-0,0445	-24,9598	64,5161	362,6038	1	16	NSNYLCNLFAAANVGR	Deamidation N /	3 / 6	De-novo
1093,1667	3	3276,4767	3276,5571	-0,0804	-24,5489	38,9831	346,5897	1	30	WNTNFAAPASSETLPHYNLQDSVVSLTQGK	Deamidation N	2 / 4	De-novo
1093,1667	3	3276,4767	3276,5571	-0,0804	-24,5489	38,9831	346,5896	1	30	WNTNFAAPASSELTPHYNLQDSVVSLTQGK	Deamidation N	2 / 4	De-novo
1093,1667	3	3276,4767	3276,5574	-0,0807	-24,6405	38,9831	346,5903	1	30	WNTNFAAPASTDLTPHYNLQDSVVSLTQGK	Deamidation N	2 / 4	De-novo
764,8524	2	1527,6892	1527,6909	-0,0017	-1,1285	68,8172	276,4763	1	16	GSTCGAMGSVVDSTLK	Oxidation M	7	De-novo
764,8524	2	1527,6892	1527,691	-0,0018	-1,1940	75,8621	272,2689	1	15	GSTCAMGSVVDSTLK	Carbamidomethyl C /	4 / 6	De-novo
764,8524	2	1527,6892	1527,6909	-0,0017	-1,1285	70,9677	275,8312	1	16	SGTCGAMGSVVDSTLK	Oxidation M	7	De-novo
756,8558	2	1511,6960	1511,6962	-0,0002	-0,1482	65,5172	245,3675	1	15	GSTCAMGSVVDSTLK	Carbamidomethyl C	4	De-novo
756,8558	2	1511,6960	1511,6962	-0,0002	-0,1482	65,5172	246,2836	1	15	GSTCAMGSVVNSTLK	Carbamidomethyl C /	4 / 11	De-novo
756,8558	2	1511,6960	1511,6962	-0,0002	-0,1482	70,1149	244,5504	1	15	SGTCAMGSVVDSTLK	Carbamidomethyl C	4	De-novo
788,9149	2	1575,8142	1575,8145	-0,0003	-0,2056	45,679	105,0546	1	14	TVQNPVVAQSLYQK	Deamidation N /	4 / 13	De-novo
595,6175	3	1783,8291	1783,8311	-0,0020	-1,1414	50,5051	151,7241	1	17	NSDYLCGNLFAAANVGR			De-novo
595,6175	3	1783,8291	1783,8311	-0,0020	-1,1414	52,5253	151,0644	1	17	SNDYLCGNLFAAANVGR			De-novo
595,6175	3	1783,8291	1783,8311	-0,0020	-1,1414	50,5051	151,7628	1	17	NSNYLCGNLFAAANVGR	Deamidation N	3	De-novo
661,3778	2	1320,7400	1320,7401	-0,0001	-0,0939	66,6667	281,6069	1	13	QVLVSAGALHTPK	Deamidation Q	1	De-novo
661,3778	2	1320,7400	1320,7401	-0,0001	-0,0939	66,6667	279,145	1	13	VQLVSAGALHTPK	Deamidation Q	2	De-novo
661,3778	2	1320,7400	1320,7401	-0,0001	-0,0939	66,6667	283,1187	1	13	EVLVSAGALHTPK			De-novo

NCBI gi|219815604|gb|ACL36977.1| putative ecdysone oxidase [Helicoverpa zea] Total Score: 181

```
                    0      120     240     360     480
                    |       |       |       |       |      |583
gi|219815604|gb|ACL36  | ___________________________________
Local hits (HSPs)      |               _ _             _
```

Score = 63 (33.8 bits)
Identities = 9/14 (64%), Positives = 10/14 (71%)

```
Query:   510 KEVLVSAGALHTPK 523
             KEV+VSAG    PK
Sbjct:   296 KEVIVSAGTFNSPK 309   MatchSeq
```

Score = 63 (33.8 bits)
Identities = 10/14 (71%), Positives = 10/14 (71%)

```
Query:    35 GTCAMGSVVDSTLK 48
             GTCAMG V DS  K
Sbjct:   525 GTCAMGLVLDSKMK 538   MatchSeq
```

Score = 55 (29.7 bits)
Identities = 9/12 (75%), Positives = 9/12 (75%)

```
Query:    76 NLCMALSTTATK 87
             NLC  L TTATK
Sbjct:   258 NLCVSLFTTATK 269   MatchSeq
```

Ha SG EST hargln|HA-GLN Contig 1427 FWD frame 1 Total Score: 214

```
                    0      130     260     390     520
                    |       |       |       |       |      |620
hargln|HA-GLN_Contig_  | ___________________________________
Local hits (HSPs)      |       _       _ _             _
```

Score = 63 (33.8 bits)
Identities = 9/14 (64%), Positives = 10/14 (71%)

```
Query:   510 KEVLVSAGALHTPK 523
             KEV+VSAG    PK
Sbjct:   308 KEVIVSAGTFNSPK 321
```

Score = 63 (33.8 bits)
Identities = 10/14 (71%), Positives = 10/14 (71%)

```
Query:    35 GTCAMGSVVDSTLK 48
             GTCAMG V DS  K
Sbjct:   537 GTCAMGLVLDSKMK 550
```

Score = 55 (29.7 bits)
Identities = 9/13 (69%), Positives = 9/13 (69%)

```
Query:    62 QSLCMALSTTATK 74
             Q LC  L TTATK
Sbjct:   269 QNLCVSLFTTATK 281
```

Score = 33 (18.6 bits)
Identities = 5/5 (100%), Positives = 5/5 (100%)

```
Query:   263 LTQGK 267
             LTQGK
Sbjct:   133 LTQGK 137
```

Spot 2

m/z	Charge	Peak mW	Peptide mW	Delta (Da)	Delta (ppm)	Ladder Score	Log Likelihood	Start	End	Sequence	Modifications	Subs	Query Tool
1409,5693	2	2817,1230	2817,3682	-0,2452	-87,0401	31,9149	226,7472	1	24	YNSLNYVPFTTSLVSMYSNLYVSR			De-novo
1409,5693	2	2817,1230	2817,3682	-0,2452	-87,0401	31,9149	226,7097	1	24	NYSLNYVPFTTSLVSMYSNLYVSR			De-novo
1409,5693	2	2817,1230	2817,3682	-0,2452	-87,0401	31,2057	226,4848	1	24	YNSLNYVPFTSTLVSMYSNLYVSR			De-novo
599,7802	2	1197,5448	1197,5779	-0,0331	-27,6592	71,9298	323,2399	1	10	QSPAYSYLNR			De-novo
599,7802	2	1197,5448	1197,5779	-0,0331	-27,6592	65,0794	319,8956	1	11	GASPAYSYLNR			De-novo
599,7802	2	1197,5448	1197,5779	-0,0331	-27,6592	65,0794	316,4651	1	11	AGSPAYSYLNR			De-novo
524,7878	2	1047,5600	1047,5601	-0,0001	-0,1184	70,1754	183,6059	1	10	LGETPTFVGK			De-novo
524,7878	2	1047,5600	1047,5601	-0,0001	-0,1184	70,1754	179,419	1	10	LGQTPTFVGK	Deamidation Q	3	De-novo
524,7878	2	1047,5600	1047,5601	-0,0001	-0,1184	63,1579	172,9925	1	10	GLETPTFVGK			De-novo
1224,847	3	3671,5176	3671,6714	-0,1538	-41,8981	36,3184	292,785	1	34	TCLNVASPEVTGPMPWYPATDTSAEPVLETGMYK	Deamidation N / Oxidation	4 / 14	De-novo
1224,847	3	3671,5176	3671,6714	-0,1538	-41,8981	36,3184	292,3424	1	34	TCLNVASEPVTGPMPWYPATDTSAEPVLETGMYK	Deamidation N / Oxidation	4 / 14	De-novo
1224,847	3	3671,5176	3671,6714	-0,1538	-41,8981	36,3184	292,4767	1	34	TCLNVGTPEVTGPMPWYPATDTSAEPVLETGMYK	Deamidation N / Oxidation	4 / 14	De-novo
739,6656	3	2215,9734	2215,9514	0,0220	9,9118	41,8803	307,8396	1	20	NAVASCPMFDPAANKEYGTR	Deamidation N /	/ 6 / 8 /	De-novo
739,6656	3	2215,9734	2215,9514	0,0220	9,9118	41,4634	309,0901	1	21	NAVASCGPMFDPAANKEYGTR	Deamidation N / Oxidation	1 / 9 / 15	De-novo
739,6656	3	2215,9734	2215,9329	0,0405	18,2605	39,3162	304,0828	1	20	NAVASCEDFDPAANKEYGTR	Deamidation N /	1 / 6 / 14	De-novo
513,3192	2	1024,6228	1024,6281	-0,0053	-5,1960	64,9123	169,5295	1	10	LLQLSGLGPK			De-novo
687,872	2	1373,7284	1373,7554	-0,0270	-19,6716	65,3333	325,3346	1	13	QLLLSAGVFNSPK	Deamidation Q	1	De-novo
687,872	2	1373,7284	1373,7554	-0,0270	-19,6716	65,3333	322,5948	1	13	LQLLSAGVFNSPK	Deamidation Q	2	De-novo
687,872	2	1373,7284	1373,7554	-0,0270	-19,6716	65,3333	325,6618	1	13	ELLLSAGVFNSPK			De-novo
645,3699	2	1288,7242	1288,7279	-0,0037	-2,8897	63,4921	148,0589	1	11	ELFTPSELLLK			De-novo
645,3699	2	1288,7242	1288,7279	-0,0037	-2,8897	63,4921	147,5606	1	11	LEFTPSELLLK			De-novo
645,3699	2	1288,7242	1288,7279	-0,0037	-2,8897	63,4921	146,964	1	11	QLFTPSELLLK	Deamidation Q	1	De-novo
700,8865	2	1399,7574	1399,7571	0,0003	0,1972	64	115,2947	1	13	GGSSLLNHLLAYR			De-novo
700,8865	2	1399,7574	1399,757	0,0004	0,2686	56	114,5273	1	13	GGSSLLNHLLSFR			De-novo
700,8865	2	1399,7574	1399,7571	0,0003	0,1972	66,6667	113,2564	1	13	SGGSLLNHLLAYR			De-novo
785,9375	2	1569,8594	1569,8627	-0,0033	-2,1174	83,908	245,0403	1	15	GLGGSSLLNHLLYAR			De-novo
785,9375	2	1569,8594	1569,8628	-0,0034	-2,1811	82,716	243,3343	1	14	GLNSSLLNHLLYAR			De-novo
785,9375	2	1569,8594	1569,8628	-0,0034	-2,1811	80,2469	242,412	1	14	LGNSSLLNHLLYAR			De-novo
732,89	2	1463,7644	1463,7733	-0,0089	-6,0966	53,0864	142,381	1	14	ELNNAAVAGQVLHK	Deamidation Q	10	De-novo
732,89	2	1463,7644	1463,7733	-0,0089	-6,0966	53,0864	141,9465	1	14	ELNNAAVAGEVLHK			De-novo
732,89	2	1463,7644	1463,7733	-0,0089	-6,0966	55,5556	141,2554	1	14	ELNNAAVAGQLVHK	Deamidation Q	10	De-novo
591,2839	2	1180,5522	1180,5876	-0,0354	-30,0054	70,1754	163,675	1	10	TPPAYSYLNR			De-novo
591,2839	2	1180,5522	1180,5876	-0,0354	-30,0054	70,1754	166,3424	1	10	PTPAYSYLNR			De-novo
591,2839	2	1180,5522	1180,5625	-0,0103	-8,7450	61,4035	153,0947	1	10	GHTAYSYLNR			De-novo
803,4716	2	1604,9276	1604,9249	0,0027	1,6674	86,0215	430,7687	1	16	VLHGATNAAVLVLAEK			De-novo
803,4716	2	1604,9276	1604,9249	0,0027	1,6674	86,0215	428,5753	1	16	VLHGATNAAVLVLAQK	Deamidation Q	15	De-novo
803,4716	2	1604,9276	1604,9249	0,0027	1,6674	83,871	419,4205	1	16	LVHGATNAAVLVLAEK			De-novo
677,298	3	2028,8706	2028,9919	-0,1213	-59,8011	31,4286	259,9899	1	18	NSFLNGGKSWHAAYALHR	Deamidation N	5	De-novo
677,298	3	2028,8706	2028,9919	-0,1213	-59,8011	32,381	259,9766	1	18	NSFLNGGKSWAAHYALHR	Deamidation N	5	De-novo
677,298	3	2028,8706	2028,9919	-0,1213	-59,8011	36,3636	258,9478	1	17	NSFLNNKSWAHAYALHR	Deamidation N	5	De-novo
959,4473	2	1916,8790	1916,9667	-0,0877	-45,7619	54,0541	279,0533	1	19	SAMPPKGGPYGTVVNGQLK	Oxidation M / Deamidation	3 / 15	De-novo
845,9825	2	1689,9494	1689,9414	0,0080	4,7197	53,5354	210,9198	1	17	LLENAVGAAVAVEAVHK			De-novo
845,9825	2	1689,9494	1689,9414	0,0080	4,7197	53,5354	210,073	1	17	LLQNAVGAAVAVEAVHK	Deamidation Q	3	De-novo
845,9825	2	1689,9494	1689,9414	0,0080	4,7197	53,5354	210,761	1	17	LLENAVGAAVAVQAVHK	Deamidation Q	13	De-novo
1108,9935	2	2215,9714	2215,9329	0,0385	17,3633	47,9675	232,7496	1	21	NAAVGPSEFDECGADKEYGTR	Deamidation N	1	De-novo
1108,9935	2	2215,9714	2215,9329	0,0385	17,3633	47,9675	232,6098	1	21	ANAVGPSEFDECGADKEYGTR	Deamidation N	2	De-novo
1108,9935	2	2215,9714	2215,9658	0,0056	2,5163	39,8374	231,8875	1	21	NAAVGNGFDFPDAADKEYGTR	Deamidation N	1 / 6	De-novo
535,9819	3	1604,9223	1604,925	-0,0027	-1,7048	56,9892	308,0542	1	16	HGATNLVAAVLVLAEK			De-novo
535,9819	3	1604,9223	1604,925	-0,0027	-1,7048	56,9892	306,0926	1	16	HGATNLVAAVLVLAQK	Deamidation Q	15	De-novo

NCBI gi|190622940|gb|EDV38464.1| GF19436 [Drosophila ananassae] Total Score: 431

```
                         0       240      480      720      960
                         |        |        |        |        |        |1197
gi|190622940|gb|EDV38    | ______________________________________________
Local hits (HSPs)        |           _         _ ___    _         _
```

```
 Score = 83 (43.5 bits)
 Identities = 12/18 (66%), Positives = 13/18 (72%)

Query:   328 VFNSPKELLLSAGVFNSP 345
             VF   KE +LSAG FNSP
Sbjct:   302 VFKARKEVILSAGAFNSP 319   MatchSeq

 Score = 71 (37.4 bits)
 Identities = 11/19 (57%), Positives = 14/19 (73%)

Query:   307 KQLLLSAGVFNSPKLQLLS 325
             K+ +LSAG FNSP +  LS
Sbjct:   307 KEVILSAGAFNSPQILMLS 325   MatchSeq

 Score = 63 (33.4 bits)
 Identities = 11/16 (68%), Positives = 12/16 (75%)

Query:   323 LLSAGVFNSPKELLLS 338
             +LSAGVF SP  L LS
Sbjct:   880 ILSAGVFGSPQILMLS 895   MatchSeq

 Score = 52 (27.9 bits)
 Identities = 9/15 (60%), Positives = 11/15 (73%)

Query:   420 LGGSSLLNHLLYARG 434
             LGGSS +N  +Y RG
Sbjct:   710 LGGSSSINTMIYNRG 724   MatchSeq

 Score = 42 (22.9 bits)
 Identities = 6/9 (66%), Positives = 8/9 (88%)

Query:   810 LLQLSGLGP 818
             +L LSG+GP
Sbjct:   891 ILMLSGIGP 899   MatchSeq

 Score = 42 (22.9 bits)
 Identities = 6/9 (66%), Positives = 8/9 (88%)

Query:   298 LLQLSGLGP 306
             +L LSG+GP
Sbjct:   321 ILMLSGIGP 329   MatchSeq

 Score = 39 (21.4 bits)
 Identities = 6/11 (54%), Positives = 7/11 (63%)

Query:   539 TNAAVLVLAEK 549
             TNA    +AEK
Sbjct:  1174 TNAVAFMIAEK 1184   MatchSeq

 Score = 39 (21.4 bits)
 Identities = 6/11 (54%), Positives = 7/11 (63%)

Query:   884 TNAAVLVLAEK 894
             TNAA   + EK
Sbjct:   604 TNAAAFMIGEK 614   MatchSeq
```

ButterflyBase STP00012_1 Total Score: 206

```
                         0       130      260      390      520
                         |        |        |        |        |        |636
butterfly|STP00012_1     | ______________________________________________
Local hits (HSPs)        |           _         _         _        _
```

```
 Score = 67 (35.4 bits)
 Identities = 11/18 (61%), Positives = 13/18 (72%)

Query:   308 QLLLSAGVFNSPKLQLLS 325
             + + SAG FN PKL LLS
Sbjct:   336 EVIVSAGTFNTPKLLLLS 353

 Score = 52 (27.9 bits)
 Identities = 10/15 (66%), Positives = 10/15 (66%)

Query:   420 LGGSSLLNHLLYARG 434
             LGGS  LN   YARG
Sbjct:   167 LGGSGSLNDMVYARG 181

 Score = 51 (27.4 bits)
 Identities = 8/14 (57%), Positives = 9/14 (64%)

Query:   567 VHGATNAAVLVLAE 580
             V G TNA V  +AE
Sbjct:   610 VRGNTNAPVVMIAE 623

 Score = 36 (19.8 bits)
 Identities = 5/8 (62%), Positives = 6/8 (75%)

Query:   773 LVAAVLVL 780
             +VAAV  L
Sbjct:   480 IVAAVVLL 487
```

Ha SG EST hargln|HA-GLN Contig 236 FWD frame 1 Total Score: 814

```
                         0        80      160      240      320
                         |        |        |        |        |        |394
hargln|HA-GLN_Contig_    | ______________________________________________
Local hits (HSPs)        | _ __                   _  ______ _  __
```

```
 Score = 108 (56.0 bits)
 Identities = 14/16 (87%), Positives = 15/16 (93%)

Query:   222 TDTSAEPVLETGMYKN 237
             TDTSAEPV+ETG YKN
Sbjct:   183 TDTSAEPVIETGXYKN 198

 Score = 105 (54.5 bits)
 Identities = 15/16 (93%), Positives = 16/16 (100%)

Query:   879 VLHGATNAAVLVLAEK 894
             VLHGATNAAV+VLAEK
Sbjct:   294 VLHGATNAAVIVLAEK 309

 Score = 93 (48.5 bits)
 Identities = 12/14 (85%), Positives = 14/14 (100%)

Query:   333 KELLLSAGVFNSPK 346
             KE++LSAGVFNSPK
Sbjct:    24 KEIILSAGVFNSPK 37

 Score = 77 (40.4 bits)
 Identities = 10/11 (90%), Positives = 11/11 (100%)

Query:   799 KLGETPTFVGK 809
             K+GETPTFVGK
Sbjct:   214 KIGETPTFVGK 224

 Score = 77 (40.4 bits)
 Identities = 13/16 (81%), Positives = 14/16 (87%)

Query:   323 LLSAGVFNSPKELLLS 338
             +LSAGVFNSPK L LS
Sbjct:    27 ILSAGVFNSPKILQLS 42

 Score = 76 (39.9 bits)
 Identities = 10/12 (83%), Positives = 12/12 (100%)

Query:   809 KLLQLSGLGPKE 820
             K+LQLSG+GPKE
Sbjct:    37 KILQLSGIGPKE 48

 Score = 69 (36.4 bits)
 Identities = 10/10 (100%), Positives = 10/10 (100%)

Query:   661 AAVAVEAVHK 670
             AAVAVEAVHK
Sbjct:     5 AAVAVEAVHK 14

 Score = 68 (35.9 bits)
 Identities = 10/13 (76%), Positives = 11/13 (84%)

Query:   285 DFDPAANKEYGTR 297
             DFD  A+KEYGTR
Sbjct:   232 DFDECADKEYGTR 244

 Score = 55 (29.4 bits)
 Identities = 8/9 (88%), Positives = 9/9 (100%)

Query:   645 GTVVNGQLK 653
             GTVV+GQLK
Sbjct:   268 GTVVDGQLK 276

 Score = 48 (25.9 bits)
 Identities = 8/13 (61%), Positives = 10/13 (76%)

Query:   704 KNAAVGPSEFDEC 716
             K+AAV   +FDEC
Sbjct:   224 KDAAVVGVDFDEC 236

 Score = 38 (20.9 bits)
 Identities = 5/5 (100%), Positives = 5/5 (100%)

Query:   595 YALHR 599
             YALHR
Sbjct:   250 YALHR 254
```

Spot 3

m/z	Charge	Peak mW	Peptide mW	Delta (Da)	Delta (ppm)	Ladder Score	Log Likelihood	Start	End	Sequence	Modifications	Subs	Query Tool
692,855	2	1383,6944	1383,6921	0,0023	1,6449	65,0794	103,2208	1	11	KYVELDPEYTK			De-novo
649,3754	2	1296,7352	1296,7288	0,0064	4,9170	52	159,1883	1	13	QVLVSAGPLTSPK	Deamidation Q	1	De-novo
649,3754	2	1296,7352	1296,7289	0,0063	4,8399	52	160,7492	1	13	EVLVSAGPLTSPK			De-novo
649,3754	2	1296,7352	1296,7289	0,0063	4,8399	54,6667	160,0107	1	13	VELVSAGPLTSPK			De-novo
562,8422	2	1123,6688	1123,6713	-0,0025	-2,2462	68,254	96,6295	1	11	LSANAVALLPR			De-novo
562,8422	2	1123,6688	1123,6713	-0,0025	-2,2462	58,7302	95,7108	1	11	LSNAAVALLPR			De-novo
562,8422	2	1123,6688	1123,6713	-0,0025	-2,2462	68,254	91,9035	1	11	SLANAVALLPR			De-novo
866,9453	2	1731,8750	1731,913	-0,0380	-21,9549	51,8519	118,2844	1	14	PLMKTFNYQPPQLR			De-novo
866,9453	2	1731,8750	1731,9131	-0,0381	-22,0127	45,977	117,6408	1	15	PLMKTFNYAGPPQLR			De-novo
866,9453	2	1731,8750	1731,9131	-0,0381	-22,0127	49,3827	116,0464	1	14	LPMKTFNYQPPQLR			De-novo
746,4293	2	1490,8430	1490,8457	-0,0027	-1,8272	43,2099	85,1654	1	14	LVLLEAGGPEPLQR			De-novo
746,4293	2	1490,8430	1490,8456	-0,0026	-1,7601	41,9753	87,257	1	14	LVLLEAGGPEQPLR			De-novo
746,4293	2	1490,8430	1490,8457	-0,0027	-1,8272	43,2099	84,9667	1	14	VLLLEAGGPEPLQR			De-novo
849,3695	2	1696,7234	1696,725	-0,0016	-0,9571	62,3656	261,2333	1	16	TGVLGEPAEDCSNGYK	Carbamidomethyl C /	11 / 13	De-novo
849,3695	2	1696,7234	1696,725	-0,0016	-0,9571	60,2151	261,0511	1	16	GTVLGEPAEDCSNGYK	Carbamidomethyl C /	11 / 13	De-novo
665,7542	2	1329,4928	1329,5295	-0,0367	-27,6218	63,4921	75,9816	1	11	TMGNDYEAWSR	Deamidation N	4	De-novo
665,7542	2	1329,4928	1329,5295	-0,0367	-27,6218	63,4921	74,8521	1	11	TMGDDYEAWSR			De-novo
665,7542	2	1329,4928	1329,5472	-0,0544	-40,9342	58,7302	71,6408	1	11	TSTDNYEAWSR	Deamidation N	5	De-novo
675,3526	2	1348,6896	1348,6875	0,0021	1,5393	59,4203	131,8401	1	12	QLFESELVAGEK			De-novo
453,211	2	904,4064	904,4688	-0,0624	-69,0173	50,9804	112,2055	1	9	SLGVMVDGK			De-novo
593,3453	2	1184,6750	1184,6764	-0,0014	-1,2020	55,0725	144,1363	1	12	VAGLGLVTNNVK	Deamidation N	9	De-novo
593,3453	2	1184,6750	1184,6764	-0,0014	-1,2020	52,1739	142,8988	1	12	GLGLGLVTNNVK	Deamidation N	9	De-novo
593,3453	2	1184,6750	1184,6764	-0,0014	-1,2020	52,1739	143,3589	1	12	AVGLGLVTNNVK	Deamidation N	9	De-novo

NCBI gi|186909546|gb|ACC94296.1| glucose oxidase-like enzyme [Helicoverpa armigera] Total Score: 1152

```
                       0      130     260     390     520
                       |       |       |       |       |      |606
gi|186909546|gb|ACC94  | ___________________________________________
Local hits (HSPs)      |  _    ____ _ _ _   _ ____     _   __  _
```

```
 Score = 113 (58.0 bits)
 Identities = 16/20 (80%), Positives = 18/20 (90%)

Query:   247 DLLEPLGLPVVADVPVGKQL 266
             + LEPLG+PVVADVPVGK L
Sbjct:   337 EVLEPLGIPVVADVPVGKRL 356   MatchSeq

 Score = 111 (57.0 bits)
 Identities = 15/19 (78%), Positives = 17/19 (89%)

Query:   264 KQLVEPLGLPVVADVPVGK 282
             K+  EPLG+PVVADVPVGK
Sbjct:   336 KEVLEPLGIPVVADVPVGK 354   MatchSeq

 Score = 95 (49.0 bits)
 Identities = 13/15 (86%), Positives = 15/15 (100%)

Query:    98 KEVLVSAGPLTSPKV 112
             KEV+VSAGPLTSPK+
Sbjct:   313 KEVIVSAGPLTSPKI 327   MatchSeq

 Score = 92 (47.5 bits)
 Identities = 12/12 (100%), Positives = 12/12 (100%)

Query:   327 KYKVELDPEYTK 338
             KYKVELDPEYTK
Sbjct:   510 KYKVELDPEYTK 521   MatchSeq

 Score = 88 (45.6 bits)
 Identities = 10/12 (83%), Positives = 12/12 (100%)

Query:   556 KECNDYEAWSRE 567
             KEC+DYEAWSR+
Sbjct:   521 KECDDYEAWSRD 532   MatchSeq

 Score = 75 (39.1 bits)
 Identities = 10/14 (71%), Positives = 11/14 (78%)

Query:   525 TAARLLMHSGVGPK 538
             T   +LMHSGVGPK
Sbjct:   323 TSPKILMHSGVGPK 336   MatchSeq

 Score = 70 (36.6 bits)
 Identities = 10/11 (90%), Positives = 11/11 (100%)

Query:   372 LSANAVALLPR 382
             +SANAVALLPR
Sbjct:   449 ISANAVALLPR 459   MatchSeq

 Score = 58 (30.6 bits)
 Identities = 8/10 (80%), Positives = 8/10 (80%)

Query:    37 KGLGVEYVDK 46
             K  GVEYVDK
Sbjct:   293 KAVGVEYVDK 302   MatchSeq

 Score = 58 (30.6 bits)
 Identities = 8/8 (100%), Positives = 8/8 (100%)

Query:   439 VVDPQLRV 446
             VVDPQLRV
Sbjct:   557 VVDPQLRV 564   MatchSeq

 Score = 45 (24.2 bits)
 Identities = 6/7 (85%), Positives = 7/7 (100%)

Query:   125 NQGELGC 131
             +QGELGC
Sbjct:   131 DQGELGC 137   MatchSeq

 Score = 45 (24.2 bits)
 Identities = 7/7 (100%), Positives = 7/7 (100%)

Query:   303 GSLVDGK 309
             GSLVDGK
Sbjct:   194 GSLVDGK 200   MatchSeq

 Score = 43 (23.2 bits)
 Identities = 5/5 (100%), Positives = 5/5 (100%)

Query:   457 PNFCR 461
             PNFCR
Sbjct:   126 PNFCR 130   MatchSeq

 Score = 39 (21.2 bits)
 Identities = 5/5 (100%), Positives = 5/5 (100%)

Query:   135 WPLGK 139
             WPLGK
Sbjct:   139 WPLGK 143   MatchSeq

 Score = 38 (20.7 bits)
 Identities = 4/5 (80%), Positives = 4/5 (80%)

Query:    79 DYEAW 83
             DYE W
Sbjct:   163 DYETW 167   MatchSeq

 Score = 37 (20.2 bits)
 Identities = 4/5 (80%), Positives = 4/5 (80%)

Query:    63 WSRCE 67
             W RCE
Sbjct:    40 WGRCE 44   MatchSeq

 Score = 37 (20.2 bits)
 Identities = 4/7 (57%), Positives = 6/7 (85%)

Query:   246 RDLLEPL 252
             RDL+E +
Sbjct:   221 RDLIEAI 227   MatchSeq

 Score = 37 (20.2 bits)
 Identities = 5/5 (100%), Positives = 5/5 (100%)

Query:   449 PQLRD 453
             PQLRD
Sbjct:   218 PQLRD 222   MatchSeq

 Score = 36 (19.7 bits)
 Identities = 5/6 (83%), Positives = 5/6 (83%)

Query:   211 TAARAY 216
             T ARAY
Sbjct:   261 TTARAY 266   MatchSeq

 Score = 35 (19.2 bits)
 Identities = 5/5 (100%), Positives = 5/5 (100%)

Query:    97 PKEVL 101
             PKEVL
Sbjct:   335 PKEVL 339   MatchSeq
```

Ha SG EST hargln|HA-GLN Contig 1966 FWD frame 2 Total Score: 767

```
                       0       80      160     240     320
                       |        |        |       |        |    |355
hargln|HA-GLN_Contig_  | ___________________________________________
Local hits (HSPs)      | _ _____           ___      ___    _
```

```
 Score = 113 (58.0 bits)
 Identities = 16/20 (80%), Positives = 18/20 (90%)

Query:   247 DLLEPLGLPVVADVPVGKQL 266
             + LEPLG+PVVADVPVGK L
Sbjct:    41 EVLEPLGIPVVADVPVGKRL 60

 Score = 111 (57.0 bits)
 Identities = 15/19 (78%), Positives = 17/19 (89%)

Query:   264 KQLVEPLGLPVVADVPVGK 282
             K+  EPLG+PVVADVPVGK
Sbjct:    40 KEVLEPLGIPVVADVPVGK 58

 Score = 92 (47.5 bits)
 Identities = 12/12 (100%), Positives = 12/12 (100%)

Query:   327 KYKVELDPEYTK 338
             KYKVELDPEYTK
Sbjct:   214 KYKVELDPEYTK 225

 Score = 86 (44.6 bits)
 Identities = 12/15 (80%), Positives = 14/15 (93%)

Query:    98 KEVLVSAGPLTSPKV 112
             KEV+ SAGPLTSPK+
Sbjct:    17 KEVIASAGPLTSPKI 31

 Score = 80 (41.6 bits)
 Identities = 9/12 (75%), Positives = 12/12 (100%)

Query:   556 KECNDYEAWSRE 567
             KEC+D+EAWSR+
Sbjct:   225 KECDDFEAWSRD 236

 Score = 75 (39.1 bits)
 Identities = 10/14 (71%), Positives = 11/14 (78%)

Query:   525 TAARLLMHSGVGPK 538
             T   +LMHSGVGPK
Sbjct:    27 TSPKILMHSGVGPK 40

 Score = 70 (36.6 bits)
 Identities = 10/11 (90%), Positives = 11/11 (100%)

Query:   372 LSANAVALLPR 382
             +SANAVALLPR
Sbjct:   153 ISANAVALLPR 163

 Score = 58 (30.6 bits)
 Identities = 8/8 (100%), Positives = 8/8 (100%)

Query:   439 VVDPQLRV 446
             VVDPQLRV
Sbjct:   261 VVDPQLRV 268

 Score = 47 (25.2 bits)
 Identities = 6/6 (100%), Positives = 6/6 (100%)

Query:    32 VEYVDK 37
             VEYVDK
Sbjct:     1 VEYVDK 6

 Score = 35 (19.2 bits)
 Identities = 5/5 (100%), Positives = 5/5 (100%)

Query:    97 PKEVL 101
             PKEVL
Sbjct:    39 PKEVL 43
```

Spot 4

A-mod.dn

m/z	Charge	Peak mW	Peptide mW	Delta (Da)	Delta (ppm)	Ladder Score	Log Likelihood	Start	End	Sequence	Modifications	Subs	
													A-mod.dn
708,3136	2	1414,6116	1414,6622	-0,0506	-35,7852	53,6232	91,366	1	12	HMSDLAQKQNSR	Deamidation N	10	A-mod.dn
708,3136	2	1414,6116	1414,6622	-0,0506	-35,7852	47,8261	91,1546	1	12	HMSDLAQQKNSR	Deamidation N	10	A-mod.dn
752,8398	2	1503,6640	1503,6589	0,0051	3,3758	60,8696	125,4765	1	12	DNQQLDFENQPR	Deamidation N	2	
752,8398	2	1503,6640	1503,6589	0,0051	3,3758	60,8696	125,9688	1	12	DDQQLDFENQPR			
760,3606	2	1518,7056	1518,7202	-0,0146	-9,6292	61,3333	215,9395	1	13	LQTWVNGEEDSLK	Deamidation N	6	
760,3606	2	1518,7056	1518,7202	-0,0146	-9,6292	61,3333	216,2038	1	13	LQTWVNGEENSLK	Deamidation N	6 / 10	
760,3606	2	1518,7056	1518,7202	-0,0146	-9,6292	61,3333	216,0004	1	13	LQTWVNGQEDSLK	Deamidation N /	6 / 8	
612,8124	2	1223,6092	1223,6034	0,0058	4,7205	66,6667	142,4736	1	12	NAASSALVSEFK	Deamidation N	1	
612,8124	2	1223,6092	1223,6034	0,0058	4,7205	66,6667	140,8267	1	12	NAASSALVSQFK	Deamidation N /	1 / 10	
729,9086	2	1457,8016	1457,7977	0,0039	2,6588	59,2593	216,6501	1	14	VDDTTLGKPSLLSL			
729,9086	2	1457,8016	1457,7977	0,0039	2,6588	56,7901	216,4141	1	14	DVDTTLGKPSLLSL			
729,9086	2	1457,8016	1457,7979	0,0037	2,5216	59,2593	214,5895	1	14	VDESTLGKPSLLSL			
734,8723	2	1467,7290	1467,7245	0,0045	3,0496	60,4938	226,0315	1	14	NASASALVSEFFPK	Deamidation N	1	
734,8723	2	1467,7290	1467,7245	0,0045	3,0496	58,0247	223,6001	1	14	NTGASALVSEFFPK	Deamidation N	1	
734,8723	2	1467,7290	1467,7245	0,0045	3,0496	67,9012	223,3689	1	14	NAASSALVSEFFPK	Deamidation N	1	
616,8054	2	1231,5952	1231,5905	0,0047	3,7967	51,8519	146,7002	1	14	GSGNGGGLGKNGTR	Deamidation N	11	
439,2492	2	876,4828	876,4818	0,0010	1,1135	62,2222	70,1001	1	8	LSNAAVFR			
544,1378	2	1086,2600	1086,6185	-0,3585	-329,9447	52,6316	91,166	1	10	AAPAFLDKVR			
842,4092	2	1682,8028	1682,7939	0,0089	5,2746	61,7284	167,5787	1	14	DAPLLYPNYFEDAR			
842,4092	2	1682,8028	1682,7939	0,0089	5,2746	61,7284	167,1009	1	14	ADPLLYPNYFEDAR			
842,4092	2	1682,8028	1682,7939	0,0089	5,2746	54,321	166,799	1	14	DAPLLYPNYEFDAR			
623,3263	2	1244,6370	1244,6401	-0,0031	-2,5100	68,1159	95,0784	1	12	VTGTSLAAHEFL			
623,3263	2	1244,6370	1244,6401	-0,0031	-2,5100	68,1159	94,3	1	12	VTGTSLAAHQFL	Deamidation Q	10	
623,3263	2	1244,6370	1244,6401	-0,0031	-2,5100	63,7681	93,8443	1	12	VTGTSLAAQHFL	Deamidation Q	9	

NCBI gi|266634534|dbj|BAI49425.1| neuroglian [Mythimna separata] Total Score: 375

```
                      0       260      520     780    1040
                      |         |        |       |       |       |1254
gi|266634534|dbj|BAI4 | ____________________________________________
Local hits (HSPs)     |   _            __         __ _     _
```

```
 Score = 83 (43.1 bits)
 Identities = 10/12 (83%), Positives = 11/12 (91%)

Query:    25 DNQQLDFENQPR 36
             D QQ+DFENQPR
Sbjct:   561 DGQQIDFENQPR 572   MatchSeq
```

```
 Score = 82 (42.6 bits)
 Identities = 10/14 (71%), Positives = 13/14 (92%)

Query:    61 KLQTWVNGEENSLK 74
             K+QTWV+GEE+ LK
Sbjct:   855 KIQTWVDGEEDRLK 868   MatchSeq
```

```
 Score = 55 (29.2 bits)
 Identities = 9/10 (90%), Positives = 9/10 (90%)

Query:   102 ASSALVSQFK 111
             ASSALVS FK
Sbjct:   876 ASSALVSKFK 885   MatchSeq
```

```
 Score = 42 (22.7 bits)
 Identities = 5/6 (83%), Positives = 6/6 (100%)

Query:   126 DVDTTL 131
             DVDTT+
Sbjct:  1110 DVDTTI 1115   MatchSeq
```

```
 Score = 41 (22.2 bits)
 Identities = 6/7 (85%), Positives = 7/7 (100%)

Query:    57 EDSLKLQ 63
             +DSLKLQ
Sbjct:   549 DDSLKLQ 555   MatchSeq
```

```
 Score = 38 (20.7 bits)
 Identities = 6/6 (100%), Positives = 6/6 (100%)

Query:   270 VTGTSL 275
             VTGTSL
Sbjct:   958 VTGTSL 963   MatchSeq
```

```
 Score = 34 (18.7 bits)
 Identities = 4/5 (80%), Positives = 5/5 (100%)

Query:    34 QPRDD 38
             QPRD+
Sbjct:    94 QPRDE 98   MatchSeq
```

Ha SG EST hargln|HA-GLN Contig 3744 FWD frame 0 Total Score: 282

```
                      0         30        60       90       120
                      |          |         |        |         |   |133
hargln|HA-GLN_Contig_ | ____________________________________________
Local hits (HSPs)     |                                       _______
```

```
 Score = 107 (55.0 bits)
 Identities = 13/14 (92%), Positives = 14/14 (100%)

Query:   228 DAPLLYPNYFEDAR 241
             DAPL+YPNYFEDAR
Sbjct:   119 DAPLIYPNYFEDAR 132
```

```
 Score = 101 (52.0 bits)
 Identities = 12/13 (92%), Positives = 13/13 (100%)

Query:   244 PLLYPNYFEDARD 256
             PL+YPNYFEDARD
Sbjct:   121 PLIYPNYFEDARD 133
```

```
 Score = 74 (38.6 bits)
 Identities = 9/12 (75%), Positives = 10/12 (83%)

Query:   253 DARDAPLLYPNY 264
             D  DAPL+YPNY
Sbjct:   116 DPKDAPLIYPNY 127
```

Spot 5

m/z	Charge	Peak mW	Peptide mW	Delta (Da)	Delta (ppm)	Ladder Score	Log Likelihood	Start	End	Sequence	Modifications	Subs	Query Tool
463,7313	2	925,4470	925,4506	-0,0036	-3,9159	66,6667	140,2429	1	8	TTLYSEGR			De-novo
463,7313	2	925,4470	925,4506	-0,0036	-3,9159	66,6667	141,4196	1	8	TTLYSQGR	Deamidation Q	6	De-novo
505,2643	2	1008,5130	1008,5128	0,0002	0,1745	76,4706	174,7936	1	9	TFPNTSTLK	Deamidation N	4	De-novo
505,2643	2	1008,5130	1008,5128	0,0002	0,1745	72,549	174,6526	1	9	FTPNTSTLK	Deamidation N	4	De-novo
505,2643	2	1008,5130	1008,5128	0,0002	0,1745	76,4706	172,7477	1	9	TFPDTSTLK			De-novo
652,8293	2	1303,6430	1303,6406	0,0024	1,8226	66,6667	256,0997	1	11	YNLADPDLVQR	Deamidation N	2	De-novo
652,8293	2	1303,6430	1303,6406	0,0024	1,8226	66,6667	256,8397	1	11	YNLADPNLVQR	Deamidation N	2 / 7	De-novo
652,8293	2	1303,6430	1303,6406	0,0024	1,8226	66,6667	255,8709	1	11	YNLANPDLVQR	Deamidation N	2 / 5	De-novo
838,4107	2	1674,8058	1674,807	-0,0012	-0,7308	67,7419	256,4279	1	16	MLCDLGAGLTLGANPR	Oxidation M /	1 / 3 / 14	De-novo
838,4107	2	1674,8058	1674,807	-0,0012	-0,7308	67,7419	260,1039	1	16	MLCNLGAGLTLGANPR	Oxidation M /	/ 3 / 4 /	De-novo
838,4107	2	1674,8058	1674,8069	-0,0011	-0,6711	73,1183	255,5809	1	16	MLCDLGAGLTVAANPR	Oxidation M /	1 / 3 / 14	De-novo
792,9056	2	1583,7956	1583,8341	-0,0385	-24,3233	59,7701	98,5515	1	15	MLSPLGAGLTELNPR	Oxidation M	1	De-novo
792,9056	2	1583,7956	1583,834	-0,0384	-24,2601	54,8387	101,2466	1	16	MLSPLGAGLTGVAQPR	Oxidation M / Deamidation	1 / 14	De-novo
586,8555	2	1171,6954	1171,6964	-0,0010	-0,8739	52,381	39,5572	1	11	LALELVPAHLP			De-novo
586,8555	2	1171,6954	1171,6964	-0,0010	-0,8739	41,2698	38,3144	1	11	LALEHLPLLGP			De-novo
586,8555	2	1171,6954	1171,6964	-0,0010	-0,8739	49,2063	39,127	1	11	LALEVLPAHLP			De-novo
743,7122	3	2228,1132	2228,1299	-0,0167	-7,5112	43,5897	201,2866	1	20	TPPLNHAAGVFYCLVAEQLK	Deamidation N /	5 / 13	De-novo
1177,6318	2	2353,2480	2353,2529	-0,0049	-2,0924	56,5891	368,8972	1	22	VLNATPLLDLSPLYGNTVEPAR	Deamidation N	3	De-novo
1177,6318	2	2353,2480	2353,2529	-0,0049	-2,0924	56,5891	368,4336	1	22	VLNATPLLDLSPLYGNTVQPAR	Deamidation N /	3 / 19	De-novo
1177,6318	2	2353,2480	2353,2529	-0,0049	-2,0924	56,5891	369,422	1	22	VLNATPLLNLSPLYGNTVEPAR	Deamidation N	3 / 9	De-novo
899,4656	2	1796,9156	1796,9197	-0,0041	-2,2950	68,8172	305,1041	1	16	TSFPLVNLTSNTDFLK	Deamidation N	7	De-novo
899,4656	2	1796,9156	1796,9197	-0,0041	-2,2950	68,8172	305,5064	1	16	TSFPLVNLTSNTNFLK	Deamidation N	7 / 13	De-novo
899,4656	2	1796,9156	1796,9197	-0,0041	-2,2950	68,8172	304,0675	1	16	STFPLVNLTSNTDFLK	Deamidation N	7	De-novo
951,4271	2	1900,8386	1900,8439	-0,0053	-2,8009	45,045	89,5126	1	19	SATDGGTANLNYYGASNKP	Deamidation N	17	De-novo
951,4271	2	1900,8386	1900,8439	-0,0053	-2,8009	43,2432	89,404	1	19	ASTDGGTANLNYYGASNKP	Deamidation N	17	De-novo
951,4271	2	1900,8386	1900,855	-0,0164	-8,6403	45,045	91,7769	1	19	SATDGGTANLNYGYASRSP			De-novo
785,4233	3	2353,2465	2353,2529	-0,0064	-2,7349	58,1395	403,0205	1	22	TANVLPLLDLSPLYGNTVEPAR	Deamidation N	3	De-novo
785,4233	3	2353,2465	2353,2529	-0,0064	-2,7349	56,5891	403,5029	1	22	VLTNAPLLDLSPLYGNTVEPAR	Deamidation N	4	De-novo
785,4233	3	2353,2465	2353,2529	-0,0064	-2,7349	58,1395	403,216	1	22	TANLVPLLDLSPLYGNTVEPAR	Deamidation N	3	De-novo

NCBI	gi|156555634|ref|XP 001603338.1| PREDICTED: hypothetical protein [Nasonia	Total Score: 67

```
                         0      140     280     420     560
                         |       |       |       |       |       |677
gi|156555634|ref|XP_0  | ____________________________________________
Local hits (HSPs)      |                ____
```

Score = 67 (35.3 bits)
Identities = 14/21 (66%), Positives = 14/21 (66%)

```
Query:   212 ATPLLDLSPLYGNTVEPARVL 232
             AT  LDLSPLYG   E A VL
Sbjct:   261 ATSYLDLSPLYGSSEEIAHVL 281   MatchSeq
```

ButterflyBase	None

Ha SG EST	hargln|HA-GLN Contig 1948 FWD frame 2	Total Score: 328

```
                         0       80      160     240     320
                         |       |       |       |       |      |366
hargln|HA-GLN_Contig_  | ____________________________________________
Local hits (HSPs)      |     __   _           _   __
```

Score = 113 (58.3 bits)
Identities = 15/17 (88%), Positives = 17/17 (100%)

```
Query:   306 KSTFPLVNLTSNTDFLK 322
             KSTFP+V+LTSNTDFLK
Sbjct:    48 KSTFPIVDLTSNTDFLK 64
```

Score = 77 (40.3 bits)
Identities = 9/11 (81%), Positives = 11/11 (100%)

```
Query:    76 RMLCDLGAGLT 86
             RMLCD+GAG+T
Sbjct:   227 RMLCDIGAGIT 237
```

Score = 75 (39.3 bits)
Identities = 9/10 (90%), Positives = 10/10 (100%)

```
Query:   199 FYCLVAEQLK 208
             FYC+VAEQLK
Sbjct:   184 FYCIVAEQLK 193
```

Score = 63 (33.3 bits)
Identities = 7/9 (77%), Positives = 9/9 (100%)

```
Query:    46 LADPDLVQR 54
             +ADPD+VQR
Sbjct:    88 IADPDIVQR 96
```

Spot 6

m/z	Charge	Peak mW	Peptide mW	Delta (Da)	Delta (ppm)	Ladder Score	Log Likelihood	Start	End	Sequence	Modifications	Subs	Query Tool
535,2351	2	1068,4546	1068,4546	0,0000	-0,0225	62,7451	131,7692	1	9	TNYACTPNK	Deamidation N /	2 / 5	De-novo
535,2351	2	1068,4546	1068,4546	0,0000	-0,0225	52,6316	133,9455	1	10	TNYAGCTPNK	Deamidation N	2	De-novo
535,2351	2	1068,4546	1068,4546	0,0000	-0,0225	62,7451	131,3399	1	9	TDYACTPNK	Carbamidomethyl C	5	De-novo
463,7299	2	925,4442	925,4506	-0,0064	-6,9415	62,2222	132,3524	1	8	TTLYSQGR	Deamidation Q	6	De-novo
463,7299	2	925,4442	925,4506	-0,0064	-6,9415	62,2222	131,8654	1	8	TTLYSEGR			De-novo
463,7299	2	925,4442	925,4505	-0,0063	-6,8334	62,2222	133,6209	1	8	TTLYTNGR	Deamidation N	6	De-novo
628,8542	2	1255,6928	1255,6813	0,0115	9,1393	60,3175	156,546	1	11	LLSASYYKPSK			De-novo
628,8542	2	1255,6928	1255,6813	0,0115	9,1393	50,7937	149,3535	1	11	LLSASYSYKPSK			De-novo
628,8542	2	1255,6928	1255,6812	0,0116	9,2189	50,7937	147,7648	1	11	LLSASYYPKSK			De-novo
611,287	2	1220,5584	1220,5708	-0,0124	-10,1788	57,971	273,5566	1	12	NSNLMTGGVEAK	Deamidation N	3	De-novo
652,8288	2	1303,6420	1303,6406	0,0014	1,0555	76,1905	299,5799	1	11	YNLADPDLVQR	Deamidation N	2	De-novo
652,8288	2	1303,6420	1303,6406	0,0014	1,0555	76,1905	300,2887	1	11	YNLADPNLVQR	Deamidation N	2 / 7	De-novo
652,8288	2	1303,6420	1303,6406	0,0014	1,0555	76,1905	299,3194	1	11	YNLANPDLVQR	Deamidation N	2 / 5	De-novo
846,4141	2	1690,8126	1690,9001	-0,0875	-51,7618	54,5455	217,3093	1	17	VPSNPGLGAGLTEVQPR			De-novo
846,4141	2	1690,8126	1690,9001	-0,0875	-51,7618	52,5253	216,646	1	17	VPSPNGLGAGLTEVQPR			De-novo
846,4141	2	1690,8126	1690,9001	-0,0875	-51,7618	54,5455	215,7256	1	17	VPSNPGLGAGLTQVQPR	Deamidation Q	13	De-novo
838,411	2	1674,8064	1674,8069	-0,0005	-0,3129	77,0115	340,3134	1	15	MLCDLGAGLTQVQPR	Oxidation M /	1 / 3 / 11	De-novo
838,411	2	1674,8064	1674,8069	-0,0005	-0,3129	77,0115	340,016	1	15	MLCDLGAGLTEVQPR	Oxidation M /	1 / 3	De-novo
838,411	2	1674,8064	1674,807	-0,0006	-0,3726	77,0115	340,275	1	15	MLCDLGAGLTLNQPR	Oxidation M /	1 / 3 / 12	De-novo
582,6472	3	1744,9182	1744,9221	-0,0039	-2,2557	64,5161	321,029	1	16	TSRPNAFTAAQLAELR			De-novo
873,4706	2	1744,9256	1744,9221	0,0035	1,9921	89,2473	238,6789	1	16	TSRPNAFTAAQLAELR			De-novo
873,4706	2	1744,9256	1744,9221	0,0035	1,9921	89,2473	238,6789	1	16	TSRPNAFTAAQLAELR			De-novo
582,6472	3	1744,9182	1744,9221	-0,0039	-2,2557	64,5161	320,5294	1	16	TSRPNAFTAAQLAQLR	Deamidation Q	14	De-novo
582,6472	3	1744,9182	1744,9221	-0,0039	-2,2557	64,5161	320,3168	1	16	TRSPNAFTAAQLAELR			De-novo
830,396	2	1658,7764	1658,8119	-0,0355	-21,4153	70,1149	331,3502	1	15	CNLMLGAGLTEVQPR	Carbamidomethyl C /	1 / 2	De-novo
830,396	2	1658,7764	1658,8119	-0,0355	-21,4153	65,5914	334,9125	1	16	CGNLMLGAGLTEVQPR	Deamidation N	3	De-novo
830,396	2	1658,7764	1658,8119	-0,0355	-21,4153	65,5914	334,525	1	16	GCNLMLGAGLTEVQPR	Deamidation N	3	De-novo
708,3357	2	1414,6558	1414,6517	0,0041	2,8813	71,4286	146,6922	1	11	TNTNYVTWRYP	Deamidation N	2	De-novo
708,3357	2	1414,6558	1414,6517	0,0041	2,8813	71,4286	145,9788	1	11	TDTNYVTWRYP			De-novo
708,3357	2	1414,6558	1414,6517	0,0041	2,8813	68,254	144,6951	1	11	NTTNYVTWRYP	Deamidation N	1	De-novo
926,4222	2	1850,8288	1850,8257	0,0031	1,6620	63,2184	198,7618	1	15	VNPCWTDNQLFETAR	Carbamidomethyl C /	4 / 8	De-novo
926,4222	2	1850,8288	1850,8257	0,0031	1,6620	60,9195	198,7173	1	15	NVPCWTDNQLFETAR	Carbamidomethyl C /	4 / 8	De-novo
926,4222	2	1850,8288	1850,8257	0,0031	1,6620	63,2184	198,3119	1	15	VNPCWTNDQLFETAR	Carbamidomethyl C /	4 / 7	De-novo
586,8573	2	1171,6990	1171,6964	0,0026	2,1985	66,6667	145,2262	1	10	LALEYPVVLR			De-novo
586,8573	2	1171,6990	1171,6964	0,0026	2,1985	66,6667	144,6117	1	10	LALQYPVVLR	Deamidation Q	4	De-novo
586,8573	2	1171,6990	1171,6964	0,0026	2,1985	56,1404	139,7255	1	10	ALLEYPVVLR			De-novo
785,4244	3	2353,2498	2353,2529	-0,0031	-1,3326	53,4884	318,7944	1	22	NATVLPLLDLSPLYGNTVEPAR	Deamidation N	1	De-novo
785,4244	3	2353,2498	2353,2529	-0,0031	-1,3326	53,4884	318,4062	1	22	ANTVLPLLDLSPLYGNTVEPAR	Deamidation N	2	De-novo
785,4244	3	2353,2498	2353,2529	-0,0031	-1,3326	55,0388	317,8618	1	22	EGTVLPLLNLSPLYGNTVEPAR	Deamidation N	9	De-novo
899,4584	2	1796,9012	1796,902	-0,0008	-0,4586	65,5914	203,3172	1	16	LPFMGVNLTSNTNFLK	Deamidation N	7 / 13	De-novo
899,4584	2	1796,9012	1796,902	-0,0008	-0,4586	65,5914	202,4177	1	16	LPFMGVNLTSNTDFLK	Deamidation N	7	De-novo
505,2654	2	1008,5152	1008,5128	0,0024	2,3559	60,7843	194,4584	1	9	TFPNTSTLK	Deamidation N	4	De-novo
505,2654	2	1008,5152	1008,5128	0,0024	2,3559	60,7843	194,3503	1	9	FTPNTSTLK	Deamidation N	4	De-novo
505,2654	2	1008,5152	1008,5128	0,0024	2,3559	60,7843	193,2397	1	9	TFPDTSTLK			De-novo
873,4706	2	1744,9256	1744,9221	0,0035	1,9921	89,2473	237,2743	1	16	STRPNAFTAAQLAELR			De-novo
873,4706	2	1744,9256	1744,9221	0,0035	1,9921	83,871	234,7729	1	16	RSTPNAFTAAQLAELR			De-novo
1177,6248	2	2353,2340	2353,2529	-0,0189	-8,0416	53,4884	254,4883	1	22	QGTVLPLLDLSPLYGNTVEPAR	Deamidation Q	1	De-novo
1177,6248	2	2353,2340	2353,2529	-0,0189	-8,0416	54,0741	253,295	1	23	QGTVLPLLDLSPLYGGGTVEPAR	Deamidation Q	1	De-novo
1177,6248	2	2353,2340	2353,2529	-0,0189	-8,0416	53,4884	249,7046	1	22	QGTVLPLLDLSPLYNGTVEPAR	Deamidation Q	1	De-novo
830,3958	2	1658,7760	1658,812	-0,0360	-21,7167	70,1149	189,1931	1	15	MLCNLGAGLTEVQPR	Carbamidomethyl C /	3 / 4	De-novo
830,3958	2	1658,7760	1658,812	-0,0360	-21,7167	70,1149	187,1695	1	15	MLCDLGAGLTEVQPR	Carbamidomethyl C	3	De-novo
830,3958	2	1658,7760	1658,812	-0,0360	-21,7167	72,4138	188,7534	1	15	MLCNLGAGLTVEQPR	Carbamidomethyl C /	3 / 4	De-novo

NCBI gi|193624794|ref|XP_001946039.1| PREDICTED: similar to AGAP008350-PA — Total Score: 297

```
                     0        280       560       840      1120
                     |         |         |         |         |        |1363
gi|193624794|ref|XP_0  |___________________________________________
Local hits (HSPs)      | _   _  _               _  _    _   _
```

```
Score = 60 (31.7 bits)
Identities = 8/13 (61%), Positives = 9/13 (69%)

Query:   593 FTAAQLAELRRST 605
             F  AQLAE+R  T
Sbjct:  1050 FSEAQLAEIRKAT 1062   MatchSeq
```

```
Score = 54 (28.7 bits)
Identities = 8/12 (66%), Positives = 9/12 (75%)

Query:   387 VNPCWTDNQLFE 398
             VNP W +N LFE
Sbjct:   194 VNPGWSNNTLFE 205   MatchSeq
```

```
Score = 38 (20.7 bits)
Identities = 6/7 (85%), Positives = 6/7 (85%)

Query:   463 ATVLPLL 469
             ATVLP L
Sbjct:   263 ATVLPML 269   MatchSeq
```

```
Score = 38 (20.7 bits)
Identities = 6/8 (75%), Positives = 6/8 (75%)

Query:   469 LDLSPLYG 476
             LDLS  YG
Sbjct:   701 LDLSSVYG 708   MatchSeq
```

```
Score = 37 (20.2 bits)
Identities = 5/16 (31%), Positives = 10/16 (62%)

Query:   416 RVNPCWTNDQLFETAR 431
             R NP W ++ +++  R
Sbjct:   789 RLNPHWNDEIIYQNTR 804   MatchSeq
```

```
Score = 36 (19.7 bits)
Identities = 5/7 (71%), Positives = 6/7 (85%)

Query:   309 LMLGAGL 315
             LMLGA +
Sbjct:    13 LMLGASI 19   MatchSeq
```

```
Score = 34 (18.7 bits)
Identities = 5/9 (55%), Positives = 6/9 (66%)

Query:   409 NQLFETARV 417
             N+LFE   V
Sbjct:   930 NHLFENVKV 938   MatchSeq
```

ButterflyBase BMP000597_1 — Total Score: 280

```
                     0         70       140       210       280
                     |         |         |         |         |        |331
db|BMP000597_1|BMP000  |___________________________________________
Local hits (HSPs)      |  __                                 ___
```

```
Score = 77 (40.1 bits)
Identities = 12/19 (63%), Positives = 13/19 (68%)

Query:   587 STRPNAFTAAQLAELRRST 605
             S RP AF  AQLAE+R  T
Sbjct:   266 SDRPYAFNVAQLAEIRKAT 284
```

```
Score = 73 (38.2 bits)
Identities = 11/17 (64%), Positives = 12/17 (70%)

Query:   226 RTSRPNAFTAAQLAELR 242
             R  RP AF  AQLAE+R
Sbjct:   265 RSDRPYAFNVAQLAEIR 281
```

```
Score = 70 (36.7 bits)
Identities = 11/17 (64%), Positives = 12/17 (70%)

Query:   606 PNAFTAAQLAELRQGTV 622
             P AF  AQLAE+R  TV
Sbjct:   269 PYAFNVAQLAEIRKATV 285
```

```
Score = 60 (31.7 bits)
Identities = 9/17 (52%), Positives = 13/17 (76%)

Query:   387 VNPCWTDNQLFETARNV 403
             +NP W D  LF+TAR++
Sbjct:    18 INPHWDDETLFQTARDI 34
```

Ha SG EST hargln|HA-GLN_Contig_1948_FWD_frame_2 — Total Score: 543

```
                     0         80       160       240       320
                     |         |         |         |         |      |366
hargln|HA-GLN_Contig_  |___________________________________________
Local hits (HSPs)      |  _   _   _             ______
```

```
Score = 118 (60.6 bits)
Identities = 16/19 (84%), Positives = 17/19 (89%)

Query:   587 STRPNAFTAAQLAELRRST 605
             STRPNAFTAAQLAE+R  T
Sbjct:   206 STRPNAFTAAQLAEIRKAT 224
```

```
Score = 116 (59.6 bits)
Identities = 14/16 (87%), Positives = 16/16 (100%)

Query:   196 RMLCDLGAGLTEVQPR 211
             RMLCD+GAG+TEVQPR
Sbjct:   227 RMLCDIGAGITEVQPR 242
```

```
Score = 106 (54.6 bits)
Identities = 14/17 (82%), Positives = 15/17 (88%)

Query:   226 RTSRPNAFTAAQLAELR 242
             R  RPNAFTAAQLAE+R
Sbjct:   205 RSTRPNAFTAAQLAEIR 221
```

```
Score = 71 (37.2 bits)
Identities = 10/11 (90%), Positives = 11/11 (100%)

Query:   549 VNLTSNTDFLK 559
             V+LTSNTDFLK
Sbjct:    54 VDLTSNTDFLK 64
```

```
Score = 69 (36.2 bits)
Identities = 9/10 (90%), Positives = 10/10 (100%)

Query:   432 LALEYPVVLR 441
             +ALEYPVVLR
Sbjct:    19 IALEYPVVLR 28
```

```
Score = 63 (33.2 bits)
Identities = 7/9 (77%), Positives = 9/9 (100%)

Query:   100 LADPDLVQR 108
             +ADPD+VQR
Sbjct:    88 IADPDIVQR 96
```

Spot 7

m/z	Charge	Peak mW	Peptide mW	Delta (Da)	Delta (ppm)	Ladder Score	Log Likelihood	Start	End	Sequence	Modifications	Subs	Query Tool
1052,4159	3	3154,2243	3154,4873	-0,2630	-83,3847	32,2751	250,1882	1	32	NADGALSAGAAAEHTRRDTSSNASLGLDLENA			De-novo
1052,4159	3	3154,2243	3154,4873	-0,2630	-83,3847	32,2751	250,6904	1	32	NADGALSAGAAAQHTRRDTSSNASLGLDLENA	Deamidation Q	13	De-novo
1052,4159	3	3154,2243	3154,4873	-0,2630	-83,3847	32,2751	250,2504	1	32	NADGALSAGAAQAHTRRDTSSNASLGLDLENA	Deamidation Q	12	De-novo
512,2988	2	1022,5820	1022,5873	-0,0053	-5,2064	62,7451	261,883	1	9	KAPPVNELR			De-novo
512,2988	2	1022,5820	1022,5873	-0,0053	-5,2064	62,7451	260,1013	1	9	KAPPVNQLR	Deamidation Q	7	De-novo
512,2988	2	1022,5820	1022,5873	-0,0053	-5,2064	58,8235	259,7677	1	9	AKPPVNELR			De-novo
532,8187	2	1063,6218	1063,6025	0,0193	18,1233	72,549	194,4346	1	9	VVTVTLNYR			De-novo
532,8187	2	1063,6218	1063,6025	0,0193	18,1233	72,549	182,5257	1	9	VVTVTLYNR			De-novo
532,8187	2	1063,6218	1063,6027	0,0191	17,9353	63,1579	182,0141	1	10	VVTVTLGGYR			De-novo
525,2918	2	1048,5680	1048,5376	0,0304	28,9699	73,0159	255,0516	1	11	SPMALAGGFAK			De-novo
525,2918	2	1048,5680	1048,5376	0,0304	28,9699	73,0159	254,4906	1	11	MPSALAGGFAK			De-novo
525,2918	2	1048,5680	1048,5375	0,0305	29,0652	70,1754	247,4837	1	10	MPSALANFAK			De-novo
646,2897	2	1290,5638	1290,5802	-0,0164	-12,7261	68,254	288,0199	1	11	EVDFMLGYSSK	Oxidation M	5	De-novo
646,2897	2	1290,5638	1290,5802	-0,0164	-12,7261	68,254	286,8313	1	11	QVDFMLGYSSK	Deamidation Q / Oxidation	1 / 5	De-novo
646,2897	2	1290,5638	1290,5802	-0,0164	-12,7261	71,4286	287,2682	1	11	VEDFMLGYSSK	Oxidation M	5	De-novo
1050,9669	2	2099,9182	2099,9534	-0,0352	-16,7737	55,8559	315,928	1	19	ESTSPNTTLTWPAYTSSEK	Deamidation N	6	De-novo
1050,9669	2	2099,9182	2099,9534	-0,0352	-16,7737	57,6577	315,9241	1	19	SETSPNTTLTWPAYTSSEK	Deamidation N	6	De-novo
1050,9669	2	2099,9182	2099,9534	-0,0352	-16,7737	55,8559	315,2746	1	19	ESTPNSTTLTWPAYTSSEK	Deamidation N	5	De-novo
707,9977	3	2120,9697	2120,9917	-0,0220	-10,3895	45,9459	361,3066	1	19	RNFPQDPEPWDGVLAATAH	Deamidation N	2	De-novo
707,9977	3	2120,9697	2120,9917	-0,0220	-10,3895	45,9459	362,2822	1	19	RNFPQNPEPWDGVLAATAH	Deamidation N	2 / 6	De-novo
707,9977	3	2120,9697	2120,9917	-0,0220	-10,3895	45,2991	363,3391	1	20	RNFPGANPEPWDGVLAATAH	Deamidation N	2 / 7	De-novo
645,3414	2	1288,6672	1288,7279	-0,0607	-47,1193	71,4286	293,1084	1	11	ELFTPSLELLK			De-novo
645,3414	2	1288,6672	1288,7279	-0,0607	-47,1193	71,4286	292,6947	1	11	LEFTPSLELLK			De-novo
645,3414	2	1288,6672	1288,7279	-0,0607	-47,1193	80,9524	291,4024	1	11	ELFTPSELLLK			De-novo
965,9356	2	1929,8556	1929,918	-0,0624	-32,3454	50,4065	419,4642	1	21	NGGGTGGFLTKGPANPVNGSQ	Deamidation N	18	De-novo
965,9356	2	1929,8556	1929,918	-0,0624	-32,3454	50,4065	418,7703	1	21	NGGGTGGFLTKGPANPVDGSQ			De-novo
965,9356	2	1929,8556	1929,918	-0,0624	-32,3454	52,8455	423,2765	1	21	NGGGTGGFLTKGPANVPDGSQ			De-novo
976,4753	2	1950,9350	1950,9586	-0,0236	-12,1089	44,1441	236,724	1	19	FNAGGPNTPSFVFGQQGVK			De-novo
976,4753	2	1950,9350	1950,9587	-0,0237	-12,1602	49,5495	235,9092	1	19	FNAGPNSAPSFVFGQQGVK			De-novo
778,6762	3	2333,0052	2333,0696	-0,0644	-27,6186	42,2222	421,459	1	23	SPGGNEGPTPPDYGLTPPPPEEK	Deamidation N	5	De-novo
778,6762	3	2333,0052	2333,0696	-0,0644	-27,6186	42,2222	421,5404	1	23	SPGGNEGPTPPNYGLTPPPPEEK	Deamidation N	5 / 12	De-novo
778,6762	3	2333,0052	2333,0696	-0,0644	-27,6186	42,2222	419,9108	1	23	SPGGNEGPTPPDYGLTPPPPQEK	Deamidation N /	5 / 21	De-novo
428,5699	3	1282,6863	1282,7032	-0,0169	-13,2034	68,254	368,6924	1	11	YPAKPPVNELR			De-novo
428,5699	3	1282,6863	1282,7032	-0,0169	-13,2034	68,254	366,8804	1	11	YPAKPPVNQLR	Deamidation Q	9	De-novo
428,5699	3	1282,6863	1282,7032	-0,0169	-13,2034	68,254	360,6268	1	11	PYAKPPVNELR			De-novo
727,4131	2	1452,8106	1452,8088	0,0018	1,2225	73,3333	242,7588	1	13	GLPYAKPPVNELR			De-novo
727,4157	2	1452,8158	1452,8088	0,0070	4,8017	70,6667	270,6261	1	13	GLPYAKPPVNELR			De-novo
727,4157	2	1452,8158	1452,8088	0,0070	4,8017	70,6667	270,6261	1	13	GLPYAKPPVNELR			De-novo
727,4131	2	1452,8106	1452,8088	0,0018	1,2225	70,6667	241,8486	1	13	AVPYAKPPVNELR			De-novo
727,4157	2	1452,8158	1452,8088	0,0070	4,8017	70,6667	269,8319	1	13	AVPYAKPPVNELR			De-novo
727,4157	2	1452,8158	1452,8088	0,0070	4,8017	70,6667	269,8319	1	13	AVPYAKPPVNELR			De-novo
727,4131	2	1452,8106	1452,8088	0,0018	1,2225	70,6667	241,6523	1	13	VAPYAKPPVNELR			De-novo
892,4242	2	1782,8328	1782,8312	0,0016	0,8840	70,9677	284,2549	1	16	SPNTTLTWPAYTSSEK	Deamidation N	3	De-novo
892,4242	2	1782,8328	1782,8312	0,0016	0,8840	66,6667	282,9992	1	16	TNTPSLTWPAYTSSEK	Deamidation N	2	De-novo
892,4242	2	1782,8328	1782,8312	0,0016	0,8840	70,9677	281,0778	1	16	PSNTTLTWPAYTSSEK	Deamidation N	3	De-novo
727,4157	2	1452,8158	1452,8088	0,0070	4,8017	70,6667	270,0972	1	13	LGPYAKPPVNELR			De-novo

NCBI gi|158289948|ref|XP 311557.4| AGAP010390-PA [Anopheles gambiae str. PEST] Total Score: 276

```
                       0     150    300    450    600
                       |      |      |      |      |     |717
gi|158289948|ref|XP_3  | ________________________________________
Local hits (HSPs)      |   _
```

```
 Score = 94 (48.6 bits)
 Identities = 12/13 (92%), Positives = 13/13 (100%)

Query:   568 GLPYAKPPVNELR 580
             G+PYAKPPVNELR
Sbjct:    57 GIPYAKPPVNELR 69   MatchSeq
```

```
 Score = 91 (47.1 bits)
 Identities = 13/15 (86%), Positives = 14/15 (93%)

Query:   634 VPYAKPPVNELRVAP 648
              +PYAKPPVNELR AP
Sbjct:    58 IPYAKPPVNELRFAP 72   MatchSeq
```

```
 Score = 91 (47.1 bits)
 Identities = 14/16 (87%), Positives = 14/16 (87%)

Query:   709 PYAKPPVNELRAKPPV 724
             PYAKPPVNELR  PPV
Sbjct:    59 PYAKPPVNELRFAPPV 74   MatchSeq
```

ButterflyBase PXP00618 1 Total Score: 207

```
                       0     40     80     120    160
                       |      |      |      |      |     |188
butterfly|PXP00618_1   | ________________________________________
Local hits (HSPs)      |    _______           __
```

```
 Score = 59 (31.2 bits)
 Identities = 11/16 (68%), Positives = 11/16 (68%)

Query:   296 PQNPEPWDGVLAATAH 311
             PQ P PWDGV  AT H
Sbjct:    46 PQPPLPWDGVRKATEH 61
```

```
 Score = 54 (28.7 bits)
 Identities = 7/9 (77%), Positives = 8/9 (88%)

Query:   124 VVTVTLNYR 132
             VV VT+NYR
Sbjct:   129 VVLVTINYR 137
```

```
 Score = 53 (28.2 bits)
 Identities = 9/12 (75%), Positives = 9/12 (75%)

Query:   608 VPYAKPPVNELR 619
             VPYA PPV  LR
Sbjct:    31 VPYAAPPVGKLR 42
```

```
 Score = 41 (22.2 bits)
 Identities = 8/12 (66%), Positives = 8/12 (66%)

Query:   107 APPVNQLRAKPP 118
             APPV  LR K P
Sbjct:    35 APPVGKLRFKAP 46
```

Ha SG EST **= hargln|HA-GLN Contig 197 FWD frame 1** Total Score: 449

```
          Length = 470
                       0     100    200    300    400
                       |      |      |      |      |     |470
hargln|HA-GLN_Contig_  | ________________________________________
Local hits (HSPs)      | _        _  _  _  _   ___
```

```
 Score = 99 (51.1 bits)
 Identities = 14/20 (70%), Positives = 15/20 (75%)

Query:   254 KESTPNSTTLTWPAYTSSEK 273
             K S P   T+TWPAYTSSEK
Sbjct:   337 KNSDPSTDTITWPAYTSSEK 356
```

```
 Score = 91 (47.1 bits)
 Identities = 11/13 (84%), Positives = 13/13 (100%)

Query:   183 KEVDFMLGYSSKQ 195
             KEVDFM+GYSSK+
Sbjct:   169 KEVDFMIGYSSKE 181
```

```
 Score = 73 (38.1 bits)
 Identities = 9/11 (81%), Positives = 11/11 (100%)

Query:   354 ELFTPSELLLK 364
             ELFTPSE+L+K
Sbjct:   202 ELFTPSEILIK 212
```

```
 Score = 60 (31.7 bits)
 Identities = 8/9 (88%), Positives = 9/9 (100%)

Query:   439 VFGQQGVKF 447
             VFGQQGVK+
Sbjct:   283 VFGQQGVKY 291
```

```
 Score = 49 (26.2 bits)
 Identities = 7/11 (63%), Positives = 8/11 (72%)

Query:   173 KMPSALANFAK 183
             K   A+ANFAK
Sbjct:   327 KITTAIANFAK 337
```

```
 Score = 43 (23.2 bits)
 Identities = 6/7 (85%), Positives = 7/7 (100%)

Query:    20 SSNASLG 26
             SSNAS+G
Sbjct:   247 SSNASIG 253
```

```
 Score = 34 (18.7 bits)
 Identities = 7/10 (70%), Positives = 7/10 (70%)

Query:   414 FLTKGPANVP 423
             FL  G ANVP
Sbjct:     1 FLSLGDANVP 10
```

Spot 8

m/z	Charge	Peak mW	Peptide mW	Delta (Da)	Delta (ppm)	Ladder Score	Log Likelihood	Start	End	Sequence	Modifications	Subs	Query Tool
731,8465	2	1461,6774	1461,6847	-0,0073	-5,0107	44,086	71,7052	1	16	SGPGEPLGVNGHSASP			De-novo
731,8465	2	1461,6774	1461,6847	-0,0073	-5,0107	44,086	71,0793	1	16	GSPGEPLGVNGHSASP			De-novo
635,7872	2	1269,5588	1269,5547	0,0041	3,2106	66,6667	145,8985	1	11	FENATVQADMK	Deamidation N / Oxidation	3 / 10	De-novo
635,7872	2	1269,5588	1269,5547	0,0041	3,2106	66,6667	144,9691	1	11	EFNATVQADMK	Deamidation N / Oxidation	3 / 10	De-novo
635,7872	2	1269,5588	1269,5547	0,0041	3,2106	66,6667	143,5474	1	11	FQNATVQADMK	Deamidation Q /	2 / 3 / 10	De-novo
491,2486	2	980,4816	980,4815	0,0001	0,0775	50,9804	126,6806	1	9	QATEAYLGK	Deamidation Q	1	De-novo
491,2486	2	980,4816	980,4816	0,0000	-0,0245	54,902	126,2527	1	9	TAEEAYLGK			De-novo
491,2486	2	980,4816	980,4815	0,0001	0,0775	50,9804	125,4217	1	9	EATEAYLGK			De-novo
741,4108	2	1480,8060	1480,7198	0,0862	58,1987	58,6667	272,2916	1	13	PVPSYVAFTDTER			De-novo
741,4108	2	1480,8060	1480,7198	0,0862	58,1987	58,6667	273,4007	1	13	PVPSYVAFTNTER	Deamidation N	10	De-novo
741,4108	2	1480,8060	1480,7198	0,0862	58,1987	58,6667	272,5263	1	13	PVPSYVAFTDTQR	Deamidation Q	12	De-novo
744,3583	2	1486,7010	1486,694	0,0070	4,6923	54,6667	111,7841	1	13	TTPSYVATNFTER	Deamidation N	9	De-novo
744,3583	2	1486,7010	1486,694	0,0070	4,6923	46,6667	112,0237	1	13	TTPSYVATNFQTR	Deamidation N /	9 / 11	De-novo
744,3583	2	1486,7010	1486,694	0,0070	4,6923	46,6667	111,1516	1	13	TTPSYVATNFETR	Deamidation N	9	De-novo
596,3197	2	1190,6238	1190,677	-0,0532	-44,7006	48	205,3418	1	13	TLGVGGGLPAPPR			De-novo
596,3197	2	1190,6238	1190,6406	-0,0168	-14,1302	50,7246	210,5145	1	12	QLQGGGLPAPPR	Deamidation Q	1	De-novo
596,3197	2	1190,6238	1190,6406	-0,0168	-14,1302	50,7246	210,9506	1	12	ELQGGGLPAPPR			De-novo
599,3387	2	1196,6618	1196,6553	0,0065	5,4118	63,4921	243,1802	1	11	EFLTGLPAPPR			De-novo
599,3387	2	1196,6618	1196,6553	0,0065	5,4118	69,8413	243,2838	1	11	FELTGLPAPPR			De-novo
599,3387	2	1196,6618	1196,6552	0,0066	5,4953	63,4921	240,9895	1	11	QFLTGLPAPPR	Deamidation Q	1	De-novo
608,3412	2	1214,6668	1214,6619	0,0049	4,0143	49,2754	122,6462	1	12	NAGTLSGLNVLR	Deamidation N	1	De-novo
608,3412	2	1214,6668	1214,6619	0,0049	4,0143	49,2754	122,4893	1	12	ANGTLSGLNVLR	Deamidation N	2	De-novo
608,3412	2	1214,6668	1214,6619	0,0049	4,0143	49,2754	121,102	1	12	DAGTLSGLNVLR			De-novo
1012,0245	2	2022,0334	2022,017	0,0164	8,0988	56,7568	240,6772	1	19	GANAVTVLTVPAYFNDSQR			De-novo
1012,0245	2	2022,0334	2022,017	0,0164	8,0988	56,7568	241,5211	1	19	GANAVTVLTVPAYFNNSQR	Deamidation N	16	De-novo
1012,0245	2	2022,0334	2022,017	0,0164	8,0988	56,7568	239,7207	1	19	AGNAVTVLTVPAYFNDSQR			De-novo
815,9006	2	1629,7856	1629,7927	-0,0071	-4,3711	59,2593	211,5756	1	14	TFFPEEVPTATYTK			De-novo
627,3126	2	1252,6096	1252,6086	0,0010	0,7792	63,1579	88,3662	1	10	FEELNNALFR	Deamidation N	6	De-novo
627,3126	2	1252,6096	1252,6086	0,0010	0,7792	63,1579	87,569	1	10	FQELNNALFR	Deamidation Q /	2 / 6	De-novo
627,3126	2	1252,6096	1252,6086	0,0010	0,7792	73,6842	87,7704	1	10	FEELNANLFR	Deamidation N	7	De-novo
693,868	2	1385,7204	1385,7225	-0,0021	-1,5328	60,8696	275,8874	1	12	QLEGLCNPLLTK	Deamidation Q /	1 / 6	De-novo
693,868	2	1385,7204	1385,7225	-0,0021	-1,5328	63,7681	278,073	1	12	LEEGLCNPLLTK	Carbamidomethyl C	6	De-novo
693,868	2	1385,7204	1385,7225	-0,0021	-1,5328	60,8696	277,4305	1	12	ELEGLCNPLLTK	Carbamidomethyl C	6	De-novo
541,784	2	1081,5524	1081,4386	0,1138	105,2080	45,614	166,7081	1	10	GANFPMEDGK	Deamidation N / Oxidation	3 / 6	De-novo

NCBI gi|195328813|ref|XP_002031106.1| GM24208 [Drosophila sechellia] Total Score: 884

```
                     0       140      280      420      560
                     |        |        |        |        |    |651
gi|195328813|ref|XP_0  | ________________________________________
Local hits (HSPs)      |  _  _ _____        _        _       _
```

```
Score = 97 (50.2 bits)
Identities = 12/13 (92%), Positives = 13/13 (100%)

Query:   283 VLTVPAYFNDSQR 295
             V+TVPAYFNDSQR
Sbjct:   143 VITVPAYFNDSQR 155   MatchSeq

Score = 84 (43.7 bits)
Identities = 10/13 (76%), Positives = 12/13 (92%)

Query:   401 KELEGLCNPLLTK 413
             KELEG CNP++TK
Sbjct:   597 KELEGVCNPIITK 609   MatchSeq

Score = 84 (43.7 bits)
Identities = 11/11 (100%), Positives = 11/11 (100%)

Query:    95 PSYVAFTDTER 105
             PSYVAFTDTER
Sbjct:    39 PSYVAFTDTER 49   MatchSeq

Score = 76 (39.7 bits)
Identities = 10/11 (90%), Positives = 11/11 (100%)

Query:   367 RFEELNANLFR 377
             RFEELNA+LFR
Sbjct:   301 RFEELNADLFR 311   MatchSeq

Score = 75 (39.2 bits)
Identities = 9/13 (69%), Positives = 12/13 (92%)

Query:   378 QLEGLCNPLLTKL 390
             +LEG CNP++TKL
Sbjct:   598 ELEGVCNPIITKL 610   MatchSeq

Score = 71 (37.2 bits)
Identities = 10/12 (83%), Positives = 11/12 (91%)

Query:   265 DAGTLSGLNVLR 276
             DAGT+ GLNVLR
Sbjct:   160 DAGTIAGLNVLR 171   MatchSeq

Score = 66 (34.7 bits)
Identities = 9/12 (75%), Positives = 10/12 (83%)

Query:    64 MKQATEAYLGKT 75
             MK+  EAYLGKT
Sbjct:   127 MKETAEAYLGKT 138   MatchSeq

Score = 63 (33.2 bits)
Identities = 8/13 (61%), Positives = 10/13 (76%)

Query:    54 KFQNATVQADMKQ 66
             KF +A VQ DMK+
Sbjct:    77 KFDDAAVQSDMKH 89   MatchSeq

Score = 59 (31.2 bits)
Identities = 8/8 (100%), Positives = 8/8 (100%)

Query:   131 RTTPSYVA 138
             RTTPSYVA
Sbjct:    36 RTTPSYVA 43   MatchSeq

Score = 58 (30.7 bits)
Identities = 9/12 (75%), Positives = 9/12 (75%)

Query:    83 KEATEAYLGKPV 94
             KE  EAYLGK V
Sbjct:   128 KETAEAYLGKTV 139   MatchSeq

Score = 53 (28.2 bits)
Identities = 7/13 (53%), Positives = 9/13 (69%)

Query:   328 FNDSQRTFFPEEV 340
             + D   TFFPEE+
Sbjct:   107 YKDEKKTFFPEEI 119   MatchSeq

Score = 53 (28.2 bits)
Identities = 7/11 (63%), Positives = 8/11 (72%)

Query:   219 FELTGLPAPPR 229
             FEL G+P  PR
Sbjct:   459 FELSGIPPAPR 469   MatchSeq

Score = 45 (24.2 bits)
Identities = 8/11 (72%), Positives = 8/11 (72%)

Query:    78 EAYLGKEATEA 88
             EAYLGK  T A
Sbjct:   132 EAYLGKTVTNA 142   MatchSeq
```

ButterflyBase PXP00109_1 Total Score: 821

```
                     0       130      260      390      520
                     |        |        |        |        |    |629
db|PXP00109_1|PXP0010  | ________________________________________
Local hits (HSPs)      |   _  _  _ __ _        _         _       _
```

```
Score = 97 (50.2 bits)
Identities = 12/13 (92%), Positives = 13/13 (100%)

Query:   283 VLTVPAYFNDSQR 295
             V+TVPAYFNDSQR
Sbjct:   147 VITVPAYFNDSQR 159

Score = 89 (46.2 bits)
Identities = 10/13 (76%), Positives = 13/13 (100%)

Query:   401 KELEGLCNPLLTK 413
             KELEG+CNP++TK
Sbjct:   601 KELEGICNPIITK 613

Score = 84 (43.7 bits)
Identities = 11/11 (100%), Positives = 11/11 (100%)

Query:    95 PSYVAFTDTER 105
             PSYVAFTDTER
Sbjct:    43 PSYVAFTDTER 53

Score = 80 (41.7 bits)
Identities = 10/13 (76%), Positives = 13/13 (100%)

Query:    54 KFQNATVQADMKQ 66
             KF++ATVQADMK+
Sbjct:    81 KFEDATVQADMKH 93

Score = 80 (41.7 bits)
Identities = 9/13 (69%), Positives = 13/13 (100%)

Query:   378 QLEGLCNPLLTKL 390
             +LEG+CNP++TKL
Sbjct:   602 ELEGICNPIITKL 614

Score = 76 (39.7 bits)
Identities = 10/11 (90%), Positives = 11/11 (100%)

Query:   367 RFEELNANLFR 377
             RFEELNA+LFR
Sbjct:   305 RFEELNADLFR 315

Score = 71 (37.2 bits)
Identities = 10/12 (83%), Positives = 11/12 (91%)

Query:   265 DAGTLSGLNVLR 276
             D GT+SGLNVLR
Sbjct:   164 DSGTISGLNVLR 175

Score = 69 (36.2 bits)
Identities = 10/16 (62%), Positives = 12/16 (75%)

Query:    64 MKQATEAYLGKTAEEA 79
             MK+  EAYLGKT + A
Sbjct:   131 MKETAEAYLGKTVQNA 146

Score = 60 (31.7 bits)
Identities = 8/11 (72%), Positives = 9/11 (81%)

Query:   219 FELTGLPAPPR 229
             FELTG+P  PR
Sbjct:   463 FELTGIPPAPR 473

Score = 59 (31.2 bits)
Identities = 8/8 (100%), Positives = 8/8 (100%)

Query:   131 RTTPSYVA 138
             RTTPSYVA
Sbjct:    40 RTTPSYVA 47

Score = 56 (29.7 bits)
Identities = 7/7 (100%), Positives = 7/7 (100%)

Query:   334 TFFPEEV 340
             TFFPEEV
Sbjct:   117 TFFPEEV 123
```

Ha SG EST _hargln|HA-GLN_Contig_4456_FWD_frame_0 Total Score: 308

```
                     0         50        100       150       200
                     |          |          |          |          |  |221
hargln|HA-GLN_Contig_  | ________________________________________
Local hits (HSPs)      |                        __       __       ___
```

```
Score = 59 (31.2 bits)
Identities = 8/11 (72%), Positives = 9/11 (81%)

Query:    64 MKQATEAYLGK 74
             MK+  EAYLGK
Sbjct:   206 MKETAEAYLGK 216

Score = 56 (29.7 bits)
Identities = 9/12 (75%), Positives = 9/12 (75%)

Query:    83 KEATEAYLGKPV 94
             KE  EAYLGK V
Sbjct:   207 KETAEAYLGKKV 218

Score = 53 (28.2 bits)
Identities = 7/7 (100%), Positives = 7/7 (100%)

Query:    95 PSYVAFT 101
             PSYVAFT
Sbjct:   117 PSYVAFT 123

Score = 50 (26.7 bits)
Identities = 7/8 (87%), Positives = 7/8 (87%)

Query:   131 RTTPSYVA 138
             R TPSYVA
Sbjct:   114 RITPSYVA 121

Score = 46 (24.7 bits)
Identities = 7/10 (70%), Positives = 8/10 (80%)

Query:    46 NATVQADMKF 55
             +ATVQ D KF
Sbjct:   159 DATVQHDVKF 168

Score = 44 (23.7 bits)
Identities = 8/11 (72%), Positives = 8/11 (72%)

Query:    78 EAYLGKEATEA 88
             EAYLGK  T A
Sbjct:   211 EAYLGKKVTHA 221
```

Spot 9

m/z	Charge	Peak mW	Peptide mW	Delta (Da)	Delta (ppm)	Ladder Score	Log Likelihood	Start	End	Sequence	Modifications	Subs	Query Tool
534,7558	2	1067,4960	1067,5248	-0,0288	-27,0008	36,8421	71,2045	1	10	VFTGSLNSSR	Deamidation N	7	De-novo
534,7558	2	1067,4960	1067,5248	-0,0288	-27,0008	36,8421	70,7677	1	10	FVTGSLNSSR	Deamidation N	7	De-novo
534,7558	2	1067,4960	1067,4917	0,0043	4,0057	33,3333	72,2513	1	10	VMTGSSLSNR	Oxidation M / Deamidation	2 / 9	De-novo
413,2773	2	824,5390	824,5484	-0,0094	-11,4293	58,9744	83,718	1	7	LLQKPVK			De-novo
413,2773	2	824,5390	824,512	0,0270	32,7175	51,1111	79,0785	1	8	VPGTKPVK			De-novo
413,2773	2	824,5390	824,5233	0,0157	19,0122	51,2821	74,347	1	7	RTPKPVK			De-novo
552,7765	2	1103,5374	1103,5359	0,0015	1,3375	66,6667	164,7018	1	10	TGNVNNPFNK			De-novo
552,7765	2	1103,5374	1103,536	0,0014	1,2469	58,7302	165,7012	1	11	TGNVNNPFGGK			De-novo
552,7765	2	1103,5374	1103,5359	0,0015	1,3375	69,8413	165,2185	1	11	TGGGVNNPFNK			De-novo
586,2885	2	1170,5614	1170,5558	0,0056	4,7635	68,4211	122,3922	1	10	VVGNFNYETK	Deamidation N	6	De-novo
586,2885	2	1170,5614	1170,5557	0,0057	4,8490	69,8413	122,003	1	11	VVGGGFNYETK	Deamidation N	7	De-novo
734,3516	2	1466,6876	1466,6888	-0,0012	-0,8345	87,6543	418,1512	1	14	LGNGSNADDLTVYK	Deamidation N	3	De-novo
734,3516	2	1466,6876	1466,6888	-0,0012	-0,8345	87,6543	418,4734	1	14	LGNGSNADNLTVYK	Deamidation N	3 / 9	De-novo
734,3516	2	1466,6876	1466,6888	-0,0012	-0,8345	87,6543	418,0529	1	14	LGNGSNANDLTVYK	Deamidation N	3 / 8	De-novo
679,8259	2	1357,6362	1357,6586	-0,0224	-16,5167	58,0247	218,0679	1	14	NNGSSGGGQLTLPR	Deamidation N	2	De-novo
679,8259	2	1357,6362	1357,6586	-0,0224	-16,5167	59,2593	216,7616	1	14	NNGSSGGSGQLTLPR	Deamidation N	2	De-novo
679,8259	2	1357,6362	1357,6586	-0,0224	-16,5167	58,0247	221,0046	1	14	NNGSSGGGQLTLRP	Deamidation N	2	De-novo
447,2435	2	892,4714	892,4291	0,0423	47,3718	58,8235	115,529	1	9	GDAFDGALK			De-novo
530,2822	2	1058,5488	1058,5608	-0,0120	-11,3588	64,7059	190,5319	1	9	ELELTQGLR	Deamidation Q	6	De-novo
530,2822	2	1058,5488	1058,5608	-0,0120	-11,3588	64,7059	189,6111	1	9	ELELTEGLR			De-novo
530,2822	2	1058,5488	1058,5608	-0,0120	-11,3588	64,7059	187,6096	1	9	QLELTQGLR	Deamidation Q	1 / 6	De-novo
672,3345	2	1342,6534	1342,663	-0,0096	-7,1678	76,8116	284,7845	1	12	NNGWSGQLTLPR	Deamidation N	2	De-novo
672,3345	2	1342,6534	1342,663	-0,0096	-7,1678	76,8116	281,7652	1	12	NDGWSGQLTLPR			De-novo
672,3345	2	1342,6534	1342,663	-0,0096	-7,1678	71,0145	275,2503	1	12	NNGWSGQLTLPR	Deamidation N	1	De-novo
921,9801	2	1841,9446	1841,9424	0,0022	1,1814	63,6364	248,1757	1	17	YPGNPVLEGAAFQPNLR			De-novo
921,9801	2	1841,9446	1841,9424	0,0022	1,1814	63,6364	246,9745	1	17	YPGNPVLQGAAFQPNLR	Deamidation Q	8	De-novo
921,9801	2	1841,9446	1841,9424	0,0022	1,1814	58,0952	246,547	1	18	YPGNPVLEGAAFGAPNLR			De-novo
746,3925	2	1490,7694	1490,7729	-0,0035	-2,3639	67,8161	368,8884	1	15	GAYTLGLEAAAANLR	Deamidation N	13	De-novo
746,3925	2	1490,7694	1490,7729	-0,0035	-2,3639	67,8161	369,3532	1	15	GYATLGLEAAAANLR	Deamidation N	13	De-novo
746,3925	2	1490,7694	1490,7729	-0,0035	-2,3639	63,2184	369,5716	1	15	TYGALGLEAAAANLR	Deamidation N	13	De-novo
513,7744	2	1025,5332	1025,5336	-0,0004	-0,4134	62,2222	87,7776	1	8	LFFPYRSP			De-novo
513,7744	2	1025,5332	1025,5334	-0,0002	-0,2184	51,1111	87,0126	1	8	FLFPYRSP			De-novo
562,3099	2	1122,6042	1122,6073	-0,0031	-2,7828	78,4314	250,1488	1	9	LFTPLTEFR			De-novo
562,3099	2	1122,6042	1122,6073	-0,0031	-2,7828	78,4314	248,8828	1	9	LFTPLTQFR	Deamidation Q	7	De-novo
562,3099	2	1122,6042	1122,6073	-0,0031	-2,7828	74,5098	238,6454	1	9	FLTPLTEFR			De-novo
521,2831	2	1040,5506	1040,5615	-0,0109	-10,4982	56,8627	61,3896	1	9	QQPLTEGLR			De-novo
521,2831	2	1040,5506	1040,5615	-0,0109	-10,4982	54,902	61,2704	1	9	QQPLTEVAR			De-novo
521,2831	2	1040,5506	1040,5437	0,0069	6,6081	54,902	59,9841	1	9	HLCLTEVAR			De-novo
553,305	2	1104,5944	1104,5815	0,0129	11,6569	50,9804	96,3929	1	9	LTEPLTEFR			De-novo
553,305	2	1104,5944	1104,5815	0,0129	11,6569	50,9804	96,9096	1	9	LTQPLTEFR	Deamidation Q	3	De-novo
553,305	2	1104,5944	1104,5815	0,0129	11,6569	49,0196	96,202	1	9	LETPLTEFR			De-novo
692,8077	2	1383,5998	1383,6089	-0,0091	-6,5943	42,6667	92,3952	1	13	VMLGNSENSFGGR	Oxidation M / Deamidation	2 / 8	De-novo
692,8077	2	1383,5998	1383,6089	-0,0091	-6,5943	42,6667	91,1679	1	13	VMLGNSESNFGGR	Oxidation M / Deamidation	2 / 9	De-novo
692,8077	2	1383,5998	1383,6089	-0,0091	-6,5943	37,3333	91,4274	1	13	MVLGNSENSFGGR	Oxidation M / Deamidation	1 / 8	De-novo

NCBI gi|156968287|gb|ABU98615.1| fructosidase [Helicoverpa armigera] Total Score: 712

```
                    0      100      200      300      400
                    |       |        |        |        |       |479
gi|156968287|gb|ABU98  | ________________________________________
Local hits (HSPs)      |        _ __ _      _     ___ _     _
```

Score = 115 (59.4 bits)
Identities = 14/17 (82%), Positives = 16/17 (94%)

```
Query:   262 YPGNPVLEGAAFQPNLR 278
             YPGNPV+EGA FQPN+R
Sbjct:   144 YPGNPVIEGADFQPNIR 160   MatchSeq
```

Score = 96 (49.9 bits)
Identities = 11/13 (84%), Positives = 13/13 (100%)

```
Query:   237 RNDGWSGQLTLPR 249
             RNDGWSGQ+T+PR
Sbjct:   308 RNDGWSGQITIPR 320   MatchSeq
```

Score = 83 (43.4 bits)
Identities = 12/14 (85%), Positives = 13/14 (92%)

```
Query:   106 LGNGSNADDLTVYK 119
             LG+ SNADDLTVYK
Sbjct:   455 LGDSSNADDLTVYK 468   MatchSeq
```

Score = 80 (41.9 bits)
Identities = 11/13 (84%), Positives = 13/13 (100%)

```
Query:   316 YTLGLEAAAANLR 328
             YTLGLEAAAA++R
Sbjct:   355 YTLGLEAAAADIR 367   MatchSeq
```

Score = 68 (35.9 bits)
Identities = 10/10 (100%), Positives = 10/10 (100%)

```
Query:   207 RELELTEGLR 216
             RELELTEGLR
Sbjct:   320 RELELTEGLR 329   MatchSeq
```

Score = 65 (34.4 bits)
Identities = 9/10 (90%), Positives = 9/10 (90%)

```
Query:    53 TGNVNNPFNK 62
             TGNVNNP NK
Sbjct:   118 TGNVNNPINK 127   MatchSeq
```

Score = 60 (31.9 bits)
Identities = 8/12 (66%), Positives = 10/12 (83%)

```
Query:    85 VVGNFNYETKVV 96
             VVGNF+YE  +V
Sbjct:   252 VVGNFDYENEIV 263   MatchSeq
```

Score = 55 (29.3 bits)
Identities = 8/8 (100%), Positives = 8/8 (100%)

```
Query:    30 RLLQKPVK 37
             RLLQKPVK
Sbjct:   329 RLLQKPVK 336   MatchSeq
```

Score = 47 (25.3 bits)
Identities = 8/9 (88%), Positives = 8/9 (88%)

```
Query:   590 LTEGLRLTQ 598
             LTEGLRL Q
Sbjct:   324 LTEGLRLLQ 332   MatchSeq
```

Score = 43 (23.3 bits)
Identities = 6/6 (100%), Positives = 6/6 (100%)

```
Query:   482 MVLGNS 487
             MVLGNS
Sbjct:   173 MVLGNS 178   MatchSeq
```

Ha SG EST hargln|HA-GLN Contig 477 FWD frame 1 Total Score: 797

```
                    0      90      180      270      360
                    |       |       |        |        |       |442
hargln|HA-GLN_Contig_  | ________________________________________
Local hits (HSPs)      |  _ __ _        __     __ ___       _
```

Score = 106 (54.9 bits)
Identities = 13/22 (59%), Positives = 16/22 (72%)

```
Query:   257 LTLPRYPGNPVLEGAAFQPNLR 278
             +    Y GNPV+EGA FQPN+R
Sbjct:    47 ISVTKYAGNPVIEGADFQPNIR 68
```

Score = 96 (49.9 bits)
Identities = 11/13 (84%), Positives = 13/13 (100%)

```
Query:   237 RNDGWSGQLTLPR 249
             RNDGWSGQ+T+PR
Sbjct:   215 RNDGWSGQITIPR 227
```

Score = 83 (43.4 bits)
Identities = 12/14 (85%), Positives = 13/14 (92%)

```
Query:   106 LGNGSNADDLTVYK 119
             LG+ SNADDLTVYK
Sbjct:   362 LGDSSNADDLTVYK 375
```

Score = 80 (41.9 bits)
Identities = 11/13 (84%), Positives = 13/13 (100%)

```
Query:   316 YTLGLEAAAANLR 328
             YTLGLEAAAA++R
Sbjct:   262 YTLGLEAAAADIR 274
```

Score = 76 (39.9 bits)
Identities = 10/10 (100%), Positives = 10/10 (100%)

```
Query:    53 TGNVNNPFNK 62
             TGNVNNPFNK
Sbjct:    26 TGNVNNPFNK 35
```

Score = 76 (39.9 bits)
Identities = 10/11 (90%), Positives = 11/11 (100%)

```
Query:   513 VVGNFNYETKL 523
             VVGNF+YETKL
Sbjct:   160 VVGNFDYETKL 170
```

Score = 68 (35.9 bits)
Identities = 10/10 (100%), Positives = 10/10 (100%)

```
Query:   207 RELELTEGLR 216
             RELELTEGLR
Sbjct:   227 RELELTEGLR 236
```

Score = 67 (35.4 bits)
Identities = 8/10 (80%), Positives = 10/10 (100%)

```
Query:   578 LFTPLTEFRQ 587
             LFTP+TEFR+
Sbjct:   170 LFTPITEFRE 179
```

Score = 55 (29.3 bits)
Identities = 8/8 (100%), Positives = 8/8 (100%)

```
Query:    30 RLLQKPVK 37
             RLLQKPVK
Sbjct:   236 RLLQKPVK 243
```

Score = 47 (25.3 bits)
Identities = 8/9 (88%), Positives = 8/9 (88%)

```
Query:   590 LTEGLRLTQ 598
             LTEGLRL Q
Sbjct:   231 LTEGLRLLQ 239
```

Score = 43 (23.3 bits)
Identities = 6/6 (100%), Positives = 6/6 (100%)

```
Query:   482 MVLGNS 487
             MVLGNS
Sbjct:    81 MVLGNS 86
```

Spot 10

m/z	Charge	Peak mW	Peptide mW	Delta (Da)	Delta (ppm)	Ladder Score	Log Likelihood	Start	End	Sequence	Modifications	Subs	Query Tool
727,8301	2	1453,6446	1453,6686	-0,0240	-16,5265	57,3333	250,9399	1	13	ELPVNENDPNGQK	Deamidation N	10	De-novo
727,8301	2	1453,6446	1453,6686	-0,0240	-16,5265	57,3333	251,8559	1	13	ELPVNENNPNGQK	Deamidation N	8 / 10	De-novo
727,8301	2	1453,6446	1453,6686	-0,0240	-16,5265	60	250,4426	1	13	LEPVNENDPNGQK	Deamidation N	10	De-novo
742,3584	2	1482,7012	1482,7025	-0,0013	-0,8930	57,3333	108,5451	1	13	GALFTDEMVTDLR	Oxidation M	8	De-novo
742,3584	2	1482,7012	1482,7025	-0,0013	-0,8930	57,3333	107,1145	1	13	AGLFTDEMVTDLR	Oxidation M	8	De-novo
742,3584	2	1482,7012	1482,7025	-0,0013	-0,8930	57,3333	108,0551	1	13	GALFTDEMVTNLR	Oxidation M / Deamidation	8 / 11	De-novo
883,8961	2	1765,7766	1765,8094	-0,0328	-18,5886	39,7849	100,4171	1	16	WNSSALMEVSGWGNTK			De-novo
883,8961	2	1765,7766	1765,7756	0,0010	0,5527	51,4286	98,986	1	18	GSTNVEGSDGLSGWGNTK	Deamidation N	4	De-novo
883,8961	2	1765,7766	1765,8094	-0,0328	-18,5886	36,5591	99,9613	1	16	WNSLTGMEVSGWGNTK			De-novo
579,2919	2	1156,5682	1156,5724	-0,0042	-3,6522	50,6667	203,8454	1	13	ANSVGPLGAGANK	Deamidation N	2 / 12	De-novo
579,2919	2	1156,5682	1156,5724	-0,0042	-3,6522	50,6667	203,7883	1	13	NASVGPLGAGANK	Deamidation N	1 / 12	De-novo
579,2919	2	1156,5682	1156,5724	-0,0042	-3,6522	54,6667	204,3127	1	13	ANVSGPLGAGANK	Deamidation N	2 / 12	De-novo
725,8787	2	1449,7418	1449,7426	-0,0008	-0,5684	60,8696	148,6432	1	12	NELLYESPLMLK	Deamidation N	1	De-novo
725,8787	2	1449,7418	1449,7426	-0,0008	-0,5684	60,8696	148,9933	1	12	ENLLYESPLMLK	Deamidation N	2	De-novo
901,9374	2	1801,8592	1801,8809	-0,0217	-12,0563	58,6207	330,2084	1	15	FTMTSLPLLTEYNEK	Oxidation M	3	De-novo
901,9374	2	1801,8592	1801,8776	-0,0184	-10,2249	63,2184	327,0476	1	15	FYEGSLPLLTEYNEK			De-novo
901,9374	2	1801,8592	1801,8776	-0,0184	-10,2249	58,6207	326,8913	1	15	FYGESLPLLTEYNEK			De-novo
662,8903	2	1323,7650	1323,7649	0,0001	0,0574	71,0145	271,481	1	12	LPTLPLVDSLEK			De-novo
662,8903	2	1323,7650	1323,7649	0,0001	0,0574	71,0145	269,0027	1	12	LPTLPLVDSLQK	Deamidation Q	11	De-novo
662,8903	2	1323,7650	1323,7649	0,0001	0,0574	71,0145	267,5939	1	12	LPTLPLVNSLEK	Deamidation N	8	De-novo
647,906	2	1293,7964	1293,7906	0,0058	4,4644	69,5652	245,1665	1	12	TPLLPLVDSLVK			De-novo
647,906	2	1293,7964	1293,7906	0,0058	4,4644	78,2609	244,1436	1	12	LPTLPLVDSLVK			De-novo
647,906	2	1293,7964	1293,7906	0,0058	4,4644	69,5652	245,3542	1	12	TPLLPLVNSLVK	Deamidation N	8	De-novo
692,8709	2	1383,7262	1383,7227	0,0035	2,5121	69,5652	114,7184	1	12	FFVAPFPEVFGK			De-novo
692,8709	2	1383,7262	1383,7227	0,0035	2,5121	69,5652	113,9394	1	12	FFVAPFPQVFGK	Deamidation Q	8	De-novo
692,8709	2	1383,7262	1383,7227	0,0035	2,5121	69,5652	108,767	1	12	FFVAPFPEVGFK			De-novo
727,3839	2	1452,7522	1452,7476	0,0046	3,1499	72,4638	164,3122	1	12	FGEFPWMVALLK	Oxidation M	7	De-novo
727,3839	2	1452,7522	1452,7476	0,0046	3,1499	72,4638	159,9415	1	12	FGQFPWMVALLK	Deamidation Q / Oxidation	3 / 7	De-novo
727,3839	2	1452,7522	1452,7476	0,0046	3,1499	66,6667	157,1915	1	12	GFEFPWMVALLK	Oxidation M	7	De-novo
804,8656	2	1607,7156	1607,7139	0,0017	1,0425	63,4409	292,8736	1	16	NGGGSPLSCGPLEYEK	Deamidation N	1	De-novo
804,8656	2	1607,7156	1607,7139	0,0017	1,0425	63,4409	292,4385	1	16	NGGGSPLSCGPLQYEK	Deamidation N /	1 / 13	De-novo
804,8656	2	1607,7156	1607,7139	0,0017	1,0425	63,4409	291,3106	1	16	NGGGSPLSCGPLEYQK	Deamidation N /	1 / 15	De-novo
621,3541	2	1240,6926	1240,6187	0,0739	59,5477	66,6667	189,4917	1	11	DPETLPENVVK	Deamidation N	8	De-novo
621,3541	2	1240,6926	1240,6187	0,0739	59,5477	66,6667	187,682	1	11	DPETLPEDVVK			De-novo
621,3541	2	1240,6926	1240,6187	0,0739	59,5477	66,6667	186,6722	1	11	DPQTLPEDVVK	Deamidation Q	3	De-novo
694,8059	2	1387,5962	1387,5939	0,0023	1,6402	70,6667	230,7708	1	13	CGGWANPDGANLR	Carbamidomethyl C /	1 / 11	De-novo
694,8059	2	1387,5962	1387,5939	0,0023	1,6402	65,3333	230,2629	1	13	GCGWANPDGANLR	Carbamidomethyl C /	2 / 11	De-novo
694,8059	2	1387,5962	1387,5939	0,0023	1,6402	71,0145	228,3546	1	12	CGGWANPDQNLR	Carbamidomethyl C /	1 / 10	De-novo
718,851	2	1435,6864	1435,6885	-0,0021	-1,4794	50,7246	222,0076	1	12	NEFLNGAFPHYK			De-novo
718,851	2	1435,6864	1435,6885	-0,0021	-1,4794	50,7246	219,3486	1	12	NQFLNGAFPHYK	Deamidation Q	2	De-novo
718,851	2	1435,6864	1435,6885	-0,0021	-1,4794	58,7302	217,7175	1	11	NEFLNQFPHYK			De-novo

NCBI gi|208972549|gb|ACI32835.1| serine proteinase-like protein 1 [Helicoverpa armigera] Total Score: 388

```
                    0        90       180       270      360
                    |        |         |         |        |      |428
gi|208972549|gb|ACI32  |___________________________________________
Local hits (HSPs)      |            ______                _
```

```
 Score = 101 (51.7 bits)
 Identities = 12/13 (92%), Positives = 13/13 (100%)

Query:   407 KFGEFPWMVALLK 419
             KFGEFPWMVA+LK
Sbjct:   170 KFGEFPWMVAILK 182   MatchSeq
```

```
 Score = 99 (50.7 bits)
 Identities = 13/16 (81%), Positives = 15/16 (93%)

Query:    26 KLEPVNENDPNGQKYN 41
             K+EPVNENDP+GQK N
Sbjct:   182 KIEPVNENDPDGQKLN 197   MatchSeq
```

```
 Score = 92 (47.3 bits)
 Identities = 12/13 (92%), Positives = 13/13 (100%)

Query:   538 GCGWANPDGANLR 550
             GCGWANPDGA+LR
Sbjct:   148 GCGWANPDGADLR 160   MatchSeq
```

```
 Score = 48 (25.5 bits)
 Identities = 5/7 (71%), Positives = 7/7 (100%)

Query:   454 PLEYEKN 460
             P+EYEK+
Sbjct:   373 PIEYEKD 379   MatchSeq
```

```
 Score = 48 (25.5 bits)
 Identities = 8/10 (80%), Positives = 8/10 (80%)

Query:   443 KNGGGSPLSC 452
             K  GGSPLSC
Sbjct:   363 KGDGGSPLSC 372   MatchSeq
```

Ha SG EST hargln|HA-GLN Contig 405 FWD frame 1 Total Score: 521

```
                    0       100       200       300       400
                    |        |         |         |         |      |463
hargln|HA-GLN_Contig_  |___________________________________________
Local hits (HSPs)      |    _    __    _     __          _    _    _
```

```
 Score = 87 (44.8 bits)      Score = 83 (42.8 bits)      Score = 73 (37.9 bits)      Score = 73 (37.9 bits)
 Identities = 9/13 (69%), P  Identities = 10/12 (83%), I Identities = 9/10 (90%), P  Identities = 9/9 (100%), Positives = 9/9 (100%)
```

```
Query:   230 KNELLYESPLMLK 242
             K+E++YESP+MLK
Sbjct:   408 KDEIIYESPIMLK 420
```

```
Query:   273 GSLPLLTEYNEK 284
             GS+PL+TEYNEK
Sbjct:   167 GSIPLITEYNEK 178
```

```
Query:   106 FTDEMVTDLR 115
             FTDEM+TDLR
Sbjct:    63 FTDEMITDLR 72
```

```
Query:   589 FLNQFPHYK 597
             FLNQFPHYK
Sbjct:   118 FLNQFPHYK 126
```

```
 Score = 57 (30.0 bits)
 Identities = 7/7 (100%), Positives = 7/7 (100%)

Query:    39 KYNFTER 45
             KYNFTER
Sbjct:   109 KYNFTER 115
```

```
 Score = 43 (23.1 bits)
 Identities = 6/10 (60%), Positives = 8/10 (80%)

Query:   103 GALFTDEMVT 112
             GAL  ++MVT
Sbjct:   233 GALIANDMVT 242
```

```
 Score = 37 (20.1 bits)
 Identities = 6/8 (75%), Positives = 7/8 (87%)

Query:   504 PETLPEDV 511
             PETL +DV
Sbjct:   357 PETLIDDV 364
```

```
 Score = 34 (18.6 bits)
 Identities = 4/4 (100%), Positives = 4/4 (100%)

Query:   265 EYNE 268
             EYNE
Sbjct:   460 EYNE 463
```

```
 Score = 34 (18.6 bits)
 Identities = 4/4 (100%), Positives = 4/4 (100%)

Query:   268 EKFY 271
             EKFY
Sbjct:   223 EKFY 226
```

Spot 11

m/z	Charge	Peak mW	Peptide mW	Delta (Da)	Delta (ppm)	Ladder Score	Log Likelihood	Start	End	Sequence	Modifications	Subs	Query Tool
413,2773	2	824,5390	824,5484	-0,0094	-11,4293	61,5385	125,6758	1	7	LLQKPVK			De-novo
413,2773	2	824,5390	824,5484	-0,0094	-11,4293	48,8889	124,7891	1	8	LLGAKPVK			De-novo
413,2773	2	824,5390	824,5484	-0,0094	-11,4293	48,8889	124,6195	1	8	LLAGKPVK			De-novo
459,2618	2	916,5080	916,509	-0,0010	-1,1173	73,3333	151,8725	1	8	RGTVTLNR	Deamidation N	7	De-novo
734,3088	2	1466,6020	1466,6888	-0,0868	-59,1973	87,6543	463,3674	1	14	LGNGSNADDLTVYK	Deamidation N	3	De-novo
734,3088	2	1466,6020	1466,6888	-0,0868	-59,1973	87,6543	464,6747	1	14	LGNGSNADNLTVYK	Deamidation N	3 / 9	De-novo
734,3088	2	1466,6020	1466,6888	-0,0868	-59,1973	87,6543	464,6894	1	14	LGNGSNANDLTVYK	Deamidation N	3 / 8	De-novo
744,691	3	2231,0496	2231,0244	0,0252	11,2791	50,4065	378,0546	1	21	NPGAPDHDFYATQTLLNGSGR	Deamidation N	17	De-novo
744,691	3	2231,0496	2231,0244	0,0252	11,2791	50,4065	377,8979	1	21	NPGAPDHNFYATQTLLNGSGR	Deamidation N	8 / 17	De-novo
744,691	3	2231,0496	2231,0244	0,0252	11,2791	50,4065	377,5807	1	21	NPGAPNHDFYATQTLLNGSGR	Deamidation N	6 / 17	De-novo
672,3352	2	1342,6548	1342,663	-0,0082	-6,1251	71,0145	320,296	1	12	NNGWSGQLTLPR	Deamidation N	2	De-novo
672,3352	2	1342,6548	1342,6628	-0,0080	-5,9762	68	318,107	1	13	GNGGWSGQLTLPR	Deamidation N	2	De-novo
672,3352	2	1342,6548	1342,6628	-0,0080	-5,9762	68	317,4001	1	13	NGGGWSGQLTLPR	Deamidation N	1	De-novo
921,9758	2	1841,9360	1841,9424	-0,0064	-3,4876	75,7576	505,8132	1	17	YPGNPVLEGAAFQPNLR			De-novo
921,9758	2	1841,9360	1841,9424	-0,0064	-3,4876	70,4762	506,1693	1	18	YPGNPVLEGAAFAGPNLR			De-novo
921,9758	2	1841,9360	1841,9424	-0,0064	-3,4876	69,5238	504,363	1	18	YPGNPVLEGAAFGAPNLR			De-novo
746,3843	2	1490,7530	1490,7729	-0,0199	-13,3649	65,5172	311,0792	1	15	TYGALGLEAVGGNLR	Deamidation N	13	De-novo
746,3846	2	1490,7536	1490,7729	-0,0193	-12,9624	50,5747	176,7906	1	15	TYGALGLEAVGGNLR	Deamidation N	13	De-novo
746,3846	2	1490,7536	1490,7729	-0,0193	-12,9624	50,5747	176,7906	1	15	TYGALGLEAVGGNLR			De-novo
746,3843	2	1490,7530	1490,7729	-0,0199	-13,3649	65,5172	310,7379	1	15	TYGALGLEAAAANLR	Deamidation N	13	De-novo
746,3843	2	1490,7530	1490,7729	-0,0199	-13,3649	65,5172	310,3934	1	15	TYGALGLQAVGGNLR	Deamidation Q /	8 / 13	De-novo
562,2957	2	1122,5758	1122,6073	-0,0315	-28,0811	74,5098	250,9722	1	9	LFTPLTEFR			De-novo
562,2957	2	1122,5758	1122,6073	-0,0315	-28,0811	74,5098	249,6911	1	9	LFTPLTQFR	Deamidation Q	7	De-novo
562,2957	2	1122,5758	1122,6073	-0,0315	-28,0811	74,5098	237,4095	1	9	FLTPLTEFR			De-novo
740,3602	2	1478,7048	1478,7001	0,0047	3,1622	55,5556	211,0549	1	14	NNDSLASLFGTSPR	Deamidation N	2	De-novo
740,3602	2	1478,7048	1478,7001	0,0047	3,1622	55,5556	210,5603	1	14	DNNSLASLFGTSPR	Deamidation N	2	De-novo
740,3602	2	1478,7048	1478,7001	0,0047	3,1622	55,5556	210,1076	1	14	NDNSLASLFGTSPR	Deamidation N	1	De-novo
569,3195	2	1136,6234	1136,623	0,0004	0,3308	49,0196	67,4748	1	9	LFTPLQVYR	Deamidation Q	6	De-novo
569,3195	2	1136,6234	1136,623	0,0004	0,3308	52,9412	66,561	1	9	LFTPLEYVR			De-novo
569,3195	2	1136,6234	1136,623	0,0004	0,3308	49,0196	66,6797	1	9	LFTPLEVYR			De-novo
559,3071	2	1116,5986	1116,5564	0,0422	37,7733	60,3175	76,7976	1	11	GPGSGPLTEFR			De-novo
618,3361	2	1234,6566	1234,6345	0,0221	17,8806	45,614	71,2709	1	10	PQFTPLYNVR	Deamidation N	8	De-novo
618,3361	2	1234,6566	1234,6346	0,0220	17,7996	47,3684	71,4123	1	10	PQFTPLYVNR	Deamidation N	9	De-novo
618,3361	2	1234,6566	1234,6346	0,0220	17,7996	47,3684	69,6588	1	10	PQFTPLYVDR			De-novo
774,3472	2	1546,6788	1546,6796	-0,0008	-0,5328	64,3678	108,2514	1	15	YMVLGGGYNGSSVMP	Oxidation M	2	De-novo
774,3472	2	1546,6788	1546,6797	-0,0009	-0,5974	64,1975	105,5939	1	14	YMVLGGGYNKSDMP	Oxidation M	2	De-novo
774,3472	2	1546,6788	1546,6797	-0,0009	-0,5974	64,1975	103,5136	1	14	MYVLGGGYNKSDMP	Oxidation M	1	De-novo
1116,5281	2	2231,0406	2231,0244	0,0162	7,2505	62,7907	350,6322	1	22	HNGAPGGPDFYATQTLLNGSGR	Deamidation N	2 / 18	De-novo
1116,5281	2	2231,0406	2231,0244	0,0162	7,2505	60,9756	348,1643	1	21	HNGAPNPDFYATQTLLNGSGR	Deamidation N	2 / 17	De-novo
1116,5281	2	2231,0406	2231,0244	0,0162	7,2505	62,7907	349,9387	1	22	HNGAPGGPNFYATQTLLNGSGR	Deamidation N	2 / 9 / 18	De-novo
530,283	2	1058,5504	1058,5608	-0,0104	-9,8473	68,6274	192,2579	1	9	QLELTQGLR	Deamidation Q	1 / 6	De-novo
530,283	2	1058,5504	1058,5608	-0,0104	-9,8473	68,6274	191,4945	1	9	QLELTEGLR	Deamidation Q	1	De-novo
530,283	2	1058,5504	1058,5608	-0,0104	-9,8473	68,6274	193,7414	1	9	ELELTQGLR	Deamidation Q	6	De-novo
600,8381	2	1199,6606	1199,651	0,0096	7,9823	68,254	135,491	1	11	TLGLEAAQELR			De-novo
600,8381	2	1199,6606	1199,651	0,0096	7,9823	68,254	135,2499	1	11	TLGLEAAQQLR	Deamidation Q	9	De-novo
553,2988	2	1104,5820	1104,5564	0,0256	23,1550	66,6667	97,4088	1	10	TGGQPLTQFR	Deamidation Q	8	De-novo
553,2988	2	1104,5820	1104,5564	0,0256	23,1550	70,1754	96,3887	1	10	TGANPLTQFR	Deamidation Q	8	De-novo
553,2988	2	1104,5820	1104,5564	0,0256	23,1550	66,6667	96,0703	1	10	TGGQPLTEFR			De-novo
688,3417	2	1374,6678	1374,6561	0,0117	8,4938	68	293,5308	1	13	NNGSMSGQLTLPR	Deamidation N	2	De-novo
688,3417	2	1374,6678	1374,6561	0,0117	8,4938	68	290,1418	1	13	NDGSMSGQLTLPR			De-novo
688,3417	2	1374,6678	1374,6561	0,0117	8,4938	72	287,4428	1	13	NNGAMSGQLTLPR	Deamidation N / Oxidation	2 / 5	De-novo
746,3846	2	1490,7536	1490,7729	-0,0193	-12,9624	49,4253	176,518	1	15	TYGLAGLEAVGGNLR	Deamidation N	13	De-novo
746,3846	2	1490,7536	1490,7729	-0,0193	-12,9624	51,8519	173,1966	1	14	TYQLGLEAVGGNLR	Deamidation N	12	De-novo

NCBI gi|156968287|gb|ABU98615.1| fructosidase [Helicoverpa armigera] Total Score: 693

```
                      0        100        200       300       400
                      |         |          |         |         |        |479
gi|156968287|gb|ABU98 | ______________________________________________
Local hits (HSPs)     |         __ _          _  ___ _   __    _
```

```
 Score = 115 (59.7 bits)
 Identities = 14/17 (82%), Positives = 16/17 (94%)

Query:   175 YPGNPVLEGAAFQPNLR 191
             YPGNPV+EGA FQPN+R
Sbjct:   144 YPGNPVIEGADFQPNIR 160   MatchSeq

 Score = 91 (47.6 bits)
 Identities = 13/15 (86%), Positives = 14/15 (93%)

Query:    31 RLGNGSNADDLTVYK 45
             RLG+ SNADDLTVYK
Sbjct:   454 RLGDSSNADDLTVYK 468   MatchSeq

 Score = 90 (47.1 bits)
 Identities = 10/13 (76%), Positives = 13/13 (100%)

Query:   136 RNNGWSGQLTLPR 148
             RN+GWSGQ+T+PR
Sbjct:   308 RNDGWSGQITIPR 320   MatchSeq

 Score = 63 (33.5 bits)
 Identities = 9/11 (81%), Positives = 11/11 (100%)

Query:   277 LGLEAAAANLR 287
             LGLEAAAA++R
Sbjct:   357 LGLEAAAADIR 367   MatchSeq

 Score = 61 (32.5 bits)
 Identities = 9/10 (90%), Positives = 10/10 (100%)

Query:   565 RELELTQGLR 574
             RELELT+GLR
Sbjct:   320 RELELTEGLR 329   MatchSeq

 Score = 60 (32.0 bits)
 Identities = 9/9 (100%), Positives = 9/9 (100%)

Query:   693 LRLLQKPVK 701
             LRLLQKPVK
Sbjct:   328 LRLLQKPVK 336   MatchSeq

 Score = 50 (27.0 bits)
 Identities = 7/8 (87%), Positives = 8/8 (100%)

Query:    24 RGTVTLNR 31
             RGTVTL+R
Sbjct:   395 RGTVTLDR 402   MatchSeq

 Score = 48 (26.0 bits)
 Identities = 9/11 (81%), Positives = 9/11 (81%)

Query:     1 LLQKPVKLLGA 11
             LLQKPVK L A
Sbjct:   330 LLQKPVKELDA 340   MatchSeq

 Score = 43 (23.4 bits)
 Identities = 7/7 (100%), Positives = 7/7 (100%)

Query:   575 TLGLEAA 581
             TLGLEAA
Sbjct:   356 TLGLEAA 362   MatchSeq

 Score = 39 (21.4 bits)
 Identities = 5/5 (100%), Positives = 5/5 (100%)

Query:   440 YMVLG 444
             YMVLG
Sbjct:   172 YMVLG 176   MatchSeq

 Score = 33 (18.4 bits)
 Identities = 5/6 (83%), Positives = 6/6 (100%)

Query:    89 LNGSGR 94
             L+GSGR
Sbjct:   286 LDGSGR 291   MatchSeq
```

Ha SG EST hargln|HA-GLN_Contig_477_FWD_frame_1 Total Score: 819

```
                      0        90        180       270       360
                      |         |         |         |         |        |442
hargln|HA-GLN_Contig_ | ______________________________________________
Local hits (HSPs)     |    __ _          _  _  ___ ___  _       _
```

```
 Score = 106 (55.1 bits)
 Identities = 13/22 (59%), Positives = 16/22 (72%)

Query:   170 LTLPRYPGNPVLEGAAFQPNLR 191
             +    Y GNPV+EGA FQPN+R
Sbjct:    47 ISVTKYAGNPVIEGADFQPNIR 68

 Score = 102 (53.1 bits)
 Identities = 13/15 (86%), Positives = 15/15 (100%)

Query:    80 HDFYATQTLLNGSGR 94
             HDFYATQT+L+GSGR
Sbjct:   184 HDFYATQTILDGSGR 198

 Score = 91 (47.6 bits)
 Identities = 13/15 (86%), Positives = 14/15 (93%)

Query:    31 RLGNGSNADDLTVYK 45
             RLG+ SNADDLTVYK
Sbjct:   361 RLGDSSNADDLTVYK 375

 Score = 90 (47.1 bits)
 Identities = 10/13 (76%), Positives = 13/13 (100%)

Query:   136 RNNGWSGQLTLPR 148
             RN+GWSGQ+T+PR
Sbjct:   215 RNDGWSGQITIPR 227

 Score = 66 (35.0 bits)
 Identities = 8/9 (88%), Positives = 9/9 (100%)

Query:   303 LFTPLTEFR 311
             LFTP+TEFR
Sbjct:   170 LFTPITEFR 178

 Score = 63 (33.5 bits)
 Identities = 9/11 (81%), Positives = 11/11 (100%)

Query:   277 LGLEAAAANLR 287
             LGLEAAAA++R
Sbjct:   264 LGLEAAAADIR 274

 Score = 61 (32.5 bits)
 Identities = 9/10 (90%), Positives = 10/10 (100%)

Query:   565 RELELTQGLR 574
             RELELT+GLR
Sbjct:   227 RELELTEGLR 236

 Score = 60 (32.0 bits)
 Identities = 9/9 (100%), Positives = 9/9 (100%)

Query:   693 LRLLQKPVK 701
             LRLLQKPVK
Sbjct:   235 LRLLQKPVK 243

 Score = 50 (27.0 bits)
 Identities = 7/8 (87%), Positives = 8/8 (100%)

Query:    24 RGTVTLNR 31
             RGTVTL+R
Sbjct:   302 RGTVTLDR 309

 Score = 48 (26.0 bits)
 Identities = 9/11 (81%), Positives = 9/11 (81%)

Query:     1 LLQKPVKLLGA 11
             LLQKPVK L A
Sbjct:   237 LLQKPVKELDA 247

 Score = 43 (23.4 bits)
 Identities = 7/7 (100%), Positives = 7/7 (100%)

Query:   575 TLGLEAA 581
             TLGLEAA
Sbjct:   263 TLGLEAA 269

 Score = 39 (21.4 bits)
 Identities = 5/5 (100%), Positives = 5/5 (100%)

Query:   440 YMVLG 444
             YMVLG
Sbjct:    80 YMVLG 84
```

Spot 12

m/z	Charge	Peak mW	Peptide mW	Delta (Da)	Delta (ppm)	Ladder Score	Log Likelihood	Start	End	Sequence	Modifications	Subs	Query Tool
562,2563	2	1122,4970	1122,4974	-0,0004	-0,3777	59,6491	148,2285	1	10	TTSLENNMGR	Deamidation N	6	De-novo
562,2563	2	1122,4970	1122,4974	-0,0004	-0,3777	59,6491	146,6063	1	10	TTSLEDNMGR			De-novo
678,3237	2	1354,6318	1354,6365	-0,0047	-3,4873	76	324,4923	1	13	TSADYLATNATAR	Deamidation N	9	De-novo
678,3237	2	1354,6318	1354,6365	-0,0047	-3,4873	76	324,4956	1	13	TSANYLATNATAR	Deamidation N	4 / 9	De-novo
678,3237	2	1354,6318	1354,6365	-0,0047	-3,4873	76	322,8604	1	13	STADYLATNATAR	Deamidation N	9	De-novo
584,3063	2	1166,5970	1166,5972	-0,0002	-0,1920	66,6667	134,3783	1	11	YFESPGVAAVK			De-novo
641,3296	2	1280,6436	1280,6401	0,0035	2,7143	44,4444	93,4567	1	11	NYFESPGVQLK			De-novo
512,765	2	1023,5144	1023,5389	-0,0245	-23,9600	82,2222	186,2935	1	8	VYFLETPR			De-novo
512,765	2	1023,5144	1023,5389	-0,0245	-23,9600	82,2222	185,3369	1	8	VYFLQTPR	Deamidation Q	5	De-novo
512,765	2	1023,5144	1023,5389	-0,0245	-23,9600	68,8889	179,6837	1	8	YVFLETPR			De-novo
427,2115	2	852,4074	852,413	-0,0056	-6,5977	48,4848	126,8077	1	6	YNWTLR	Deamidation N	2	De-novo
427,2115	2	852,4074	852,413	-0,0056	-6,5977	48,4848	125,4949	1	6	YDWTLR			De-novo
427,2115	2	852,4074	852,413	-0,0056	-6,5977	45,4545	119,9806	1	6	NYWTLR	Deamidation N	1	De-novo
763,3504	3	2287,0278	2287,083	-0,0552	-24,1513	58,1395	506,8588	1	22	NPKAAMGGVYDYFESPGVAAVK	Deamidation N / Oxidation	1 / 6	De-novo
763,3504	3	2287,0278	2287,083	-0,0552	-24,1513	59,3496	502,8683	1	21	NPKAAVMNYDYFESPGVAAVK	Deamidation N / Oxidation	1 / 7	De-novo
763,3504	3	2287,0278	2287,083	-0,0552	-24,1513	59,3496	505,5772	1	21	NPKAAMNVYDYFESPGVAAVK	Deamidation N / Oxidation	1 / 6	De-novo
579,609	3	1735,8036	1735,8167	-0,0131	-7,5676	52,6882	262,3871	1	16	TADSFPHYLATNATAR	Deamidation N	12	De-novo
579,609	3	1735,8036	1735,8167	-0,0131	-7,5676	52,6882	261,0193	1	16	TADSFPHYLATDATAR			De-novo
579,609	3	1735,8036	1735,8167	-0,0131	-7,5676	50,5376	258,6974	1	16	ATDSFPHYLATDATAR			De-novo

NCBI gi|156968291|gb|ABU98617.1| unknown [Helicoverpa armigera] Total Score: 391

```
                      0        60        120       180       240
                      |         |         |         |         |      |267
gi|156968291|gb|ABU98 | ______________________________________________
Local hits (HSPs)     |              ____              ___   __
```

```
 Score = 92 (47.1 bits)
 Identities = 12/14 (85%), Positives = 14/14 (100%)

Query:   153 NYDYFESPGVAAVK 166
             N++YFESPGVAAVK
Sbjct:   215 NFNYFESPGVAAVK 228   MatchSeq

 Score = 77 (39.7 bits)
 Identities = 11/13 (84%), Positives = 12/13 (92%)

Query:    47 STADYLATNATAR 59
             STADYLAT+AT R
Sbjct:   104 STADYLATDATTR 116   MatchSeq

 Score = 68 (35.3 bits)
 Identities = 10/13 (76%), Positives = 11/13 (84%)

Query:    23 ADYLATNATARTS 35
             ADYLAT+AT R S
Sbjct:   106 ADYLATDATTRNS 118   MatchSeq

 Score = 62 (32.4 bits)
 Identities = 7/8 (87%), Positives = 8/8 (100%)

Query:    82 VYFLETPR 89
             VYF+ETPR
Sbjct:   252 VYFIETPR 259   MatchSeq

 Score = 53 (27.9 bits)
 Identities = 5/7 (71%), Positives = 7/7 (100%)

Query:   117 RNYWTLR 123
             R+YWT+R
Sbjct:    94 RDYWTIR 100   MatchSeq

 Score = 39 (21.0 bits)
 Identities = 4/6 (66%), Positives = 5/6 (83%)

Query:   114 WTLRNY 119
             WT+R Y
Sbjct:    97 WTIRAY 102   MatchSeq
```

Ha SG EST hargln|HA-GLN_Contig_333_FWD_frame_2 Total Score: 391

```
                      0        70        140       210       280
                      |         |         |         |         |        |340
hargln|HA-GLN_Contig_ | ______________________________________________
Local hits (HSPs)     |               _____             __   _
```

```
 Score = 92 (47.1 bits)
 Identities = 12/14 (85%), Positives = 14/14 (100%)

Query:   153 NYDYFESPGVAAVK 166
             N++YFESPGVAAVK
Sbjct:   255 NFNYFESPGVAAVK 268

 Score = 77 (39.7 bits)
 Score = 53 (27.9 bits)
 Identities = 5/7 (71%), Positives = 7/7 (100%)

Query:   117 RNYWTLR 123
             R+YWT+R
Sbjct:   134 RDYWTIR 140

 Score = 39 (21.0 bits)
 Identities = 4/6 (66%), Positives = 5/6 (83%)

Query:   114 WTLRNY 119
             WT+R Y
Sbjct:   137 WTIRAY 142
```

Spot 12

m/z	Charge	Peak mW	Peptide mW	Delta (Da)	Delta (ppm)	Ladder Score	Log Likelihood	Start	End	Sequence	Modifications	Subs	Query Tool
562,2548	2	1122,4940	1122,4976	-0,0036	-3,2285	54,386	188,1013	1	10	TTSLECAEGR	Carbamidomethyl C	6	De-novo
562,2548	2	1122,4940	1122,4976	-0,0036	-3,2285	54,386	188,4716	1	10	TTSLECAQGR	Carbamidomethyl C /	6 / 8	De-novo
581,3073	2	1160,5990	1160,5978	0,0012	1,0133	76,4706	232,0078	1	9	HVYFLETPR			De-novo
581,3073	2	1160,5990	1160,5978	0,0012	1,0133	76,4706	231,5512	1	9	HVYFLQTPR	Deamidation Q	6	De-novo
581,3073	2	1160,5990	1160,5978	0,0012	1,0133	80,3922	219,6832	1	9	HVYFLTEPR			De-novo
641,3295	2	1280,6434	1280,6401	0,0033	2,5581	57,971	112,7431	1	12	NYFESPGVAAVK			De-novo
641,3295	2	1280,6434	1280,6401	0,0033	2,5581	66,6667	107,632	1	12	NYFESPVGAAVK			De-novo
427,2097	2	852,4038	852,413	-0,0092	-10,8210	48,4848	132,1386	1	6	YNWTLR	Deamidation N	2	De-novo
427,2097	2	852,4038	852,413	-0,0092	-10,8210	48,4848	130,8027	1	6	YDWTLR			De-novo
427,2097	2	852,4038	852,413	-0,0092	-10,8210	45,4545	125,3561	1	6	NYWTLR	Deamidation N	1	De-novo
931,8572	2	1861,6988	1861,8807	-0,1819	-97,7098	51,3514	321,5669	1	19	TADATGGPNPYLATNATAR	Deamidation N	15	De-novo
931,8572	2	1861,6988	1861,8807	-0,1819	-97,7098	47,8632	320,4338	1	20	TADATGGPGGPYLATNATAR	Deamidation N	16	De-novo
931,8572	2	1861,6988	1861,8806	-0,1818	-97,6561	52,381	316,3779	1	18	TADATNPNPYLATNATAR	Deamidation N	14	De-novo
768,3698	4	3069,4480	3069,5916	-0,1436	-46,7971	38,0117	426,8172	1	29	QANAPVPQGAVLPEPGVVPSHVYFLETPR	Deamidation Q	1	De-novo
768,3698	4	3069,4480	3069,5916	-0,1436	-46,7971	38,0117	427,0393	1	29	QANAPVPQGAVLPEPGVVPSHVYFLQTPR	Deamidation Q	1 / 26	De-novo
768,3698	4	3069,4480	3069,5916	-0,1436	-46,7971	40,3509	428,6647	1	29	QANAPQGVPVALPEPGVVPSHVYFLETPR	Deamidation Q	1	De-novo
512,7792	2	1023,5428	1023,5389	0,0039	3,7869	75,5556	122,1822	1	8	VYFLETPR			De-novo
512,7792	2	1023,5428	1023,5389	0,0039	3,7869	75,5556	122,1521	1	8	VYFLQTPR	Deamidation Q	5	De-novo
512,7792	2	1023,5428	1023,5389	0,0039	3,7869	80	117,9252	1	8	YVFLETPR			De-novo
737,7106	3	2210,1084	2210,0969	0,0115	5,1871	47,8632	263,0622	1	20	NHLNALDVLEGNEQFLNAAK	Deamidation N	4	De-novo
737,7106	3	2210,1084	2210,0969	0,0115	5,1871	47,8632	263,9728	1	20	NHLNALNVLEGNEQFLNAAK	Deamidation N	4 / 7	De-novo
737,7106	3	2210,1084	2210,0969	0,0115	5,1871	47,8632	263,1006	1	20	NHLDALNVLEGNEQFLNAAK	Deamidation N	7	De-novo
868,913	2	1735,8104	1735,8054	0,0050	2,8667	70,9677	284,8119	1	16	FSFSTADYLATNATAR	Deamidation N	12	De-novo
868,913	2	1735,8104	1735,8054	0,0050	2,8667	70,9677	285,4303	1	16	FSFSTANYLATNATAR	Deamidation N	7 / 12	De-novo
868,913	2	1735,8104	1735,8054	0,0050	2,8667	73,1183	282,0193	1	16	SFFSTADYLATNATAR	Deamidation N	12	De-novo
771,0265	3	2310,0561	2310,0737	-0,0176	-7,6344	49,6296	287,8331	1	23	TYNAGGPQGMALHGYAPGVVTSY			De-novo
771,0265	3	2310,0561	2310,1101	-0,0540	-23,3911	43,7037	282,6597	1	23	TYNAGGPQGMALPPPHPGVVTSY			De-novo
771,0265	3	2310,0561	2310,0989	-0,0428	-18,5429	48,8889	283,8217	1	23	TYNAGGPQGMALPPYAPGVVTSY			De-novo
579,6085	3	1735,8021	1735,8167	-0,0146	-8,4318	49,4624	202,3096	1	16	FSTNAPHYLATNATAR	Deamidation N	4 / 12	De-novo
579,6085	3	1735,8021	1735,8167	-0,0146	-8,4318	51,6129	201,5506	1	16	SFTNAPHYLATNATAR	Deamidation N	4 / 12	De-novo
579,6085	3	1735,8021	1735,8167	-0,0146	-8,4318	49,4624	200,2209	1	16	FSTNAPHYLATDATAR	Deamidation N	4	De-novo
419,7621	2	837,5086	837,5073	0,0013	1,5236	48,8889	39,1089	1	8	LPGVSLPR			De-novo
490,1638	2	978,3120	978,5287	-0,2167	-221,4795	42,2222	103,1254	1	8	GALYWTLR			De-novo
490,1638	2	978,3120	978,5287	-0,2167	-221,4795	35,5556	102,0922	1	8	GAYLWTLR			De-novo
490,1638	2	978,3120	978,5287	-0,2167	-221,4795	42,2222	102,063	1	8	AGLYWTLR			De-novo

NCBI gi|156968291|gb|ABU98617.1| unknown [Helicoverpa armigera] — Total Score: 265

```
                       0        60       120      180      240
                       |         |        |        |        |    |267
gi|156968291|gb|ABU98  | ____________________________________________
Local hits (HSPs)      |           _____                        __
```

```
 Score = 81 (42.1 bits)
 Identities = 12/15 (80%), Positives = 13/15 (86%)

Query:    27 STADYLATNATARTS 41
             STADYLAT+AT R S
Sbjct:   104 STADYLATDATTRNS 118   MatchSeq
```

```
 Score = 63 (33.2 bits)
 Identities = 12/23 (52%), Positives = 15/23 (65%)

Query:   115 RNYWTLRTANHPFSYLATNATAR 137
             R+YWT+R       YLAT+AT R
Sbjct:    94 RDYWTIRAYFSTADYLATDATTR 116   MatchSeq
```

```
 Score = 62 (32.7 bits)
 Identities = 7/8 (87%), Positives = 8/8 (100%)

Query:    80 VYFLETPR 87
             VYF+ETPR
Sbjct:   252 VYFIETPR 259   MatchSeq
```

```
 Score = 59 (31.2 bits)
 Identities = 8/8 (100%), Positives = 8/8 (100%)

Query:    54 SWTTTLSN 61
             SWTTTLSN
Sbjct:    83 SWTTTLSN 90   MatchSeq
```

Ha SG EST hargln|HA-GLN Contig 333 FWD frame 2 — Total Score: 272

```
                       0        70       140      210      280
                       |         |        |        |        |      |340
hargln|HA-GLN_Contig_  | ____________________________________________
Local hits (HSPs)      |             ______                    _
```

```
 Score = 81 (42.1 bits)
 Identities = 12/15 (80%), Positives = 13/15 (86%)

Query:    27 STADYLATNATARTS 41
             STADYLAT+AT R S
Sbjct:   144 STADYLATDATTRNS 158
```

```
 Score = 66 (34.7 bits)
 Identities = 9/9 (100%), Positives = 9/9 (100%)

Query:    53 TSWTTTLSN 61
             TSWTTTLSN
Sbjct:   122 TSWTTTLSN 130
```

```
 Score = 63 (33.2 bits)
 Identities = 12/23 (52%), Positives = 15/23 (65%)

Query:   115 RNYWTLRTANHPFSYLATNATAR 137
             R+YWT+R       YLAT+AT R
Sbjct:   134 RDYWTIRAYFSTADYLATDATTR 156
```

```
 Score = 62 (32.7 bits)
 Identities = 7/8 (87%), Positives = 8/8 (100%)

Query:    80 VYFLETPR 87
             VYF+ETPR
Sbjct:   292 VYFIETPR 299
```

Spot 12

m/z	Charge	Peak mW	Peptide mW	Delta (Da)	Delta (ppm)	Ladder Score	Log Likelihood	Start	End	Sequence	Modifications	Subs	Query Tool
678,3266	2	1354,6376	1354,6365	0,0011	0,7943	62,6667	145,8327	1	13	TSADYLATNATAR	Deamidation N	9	De-novo
678,3266	2	1354,6376	1354,6365	0,0011	0,7943	62,6667	146,9296	1	13	TSANYLATNATAR	Deamidation N	4 / 9	De-novo
678,3266	2	1354,6376	1354,6365	0,0011	0,7943	62,6667	145,2535	1	13	STADYLATNATAR	Deamidation N	9	De-novo
706,3428	2	1410,6700	1410,6627	0,0073	5,1579	45,3333	160,2344	1	13	TSANTTTLSSKNW	Deamidation N	4	De-novo
706,3428	2	1410,6700	1410,6738	-0,0038	-2,7108	60	152,3264	1	13	TSWTTTLSNTSGR			De-novo
706,3428	2	1410,6700	1410,6586	0,0114	8,0643	51,8519	152,7346	1	14	TSANTTTLSNTSGR	Deamidation N	4	De-novo
512,7769	2	1023,5382	1023,5389	-0,0007	-0,7073	64,4444	165,2759	1	8	VYFLETPR			De-novo
512,7769	2	1023,5382	1023,5389	-0,0007	-0,7073	64,4444	164,2957	1	8	VYFLQTPR	Deamidation Q	5	De-novo
512,7769	2	1023,5382	1023,5389	-0,0007	-0,7073	60	160,37	1	8	YVFLETPR			De-novo
427,2116	2	852,4076	852,413	-0,0054	-6,3631	75,7576	114,715	1	6	YNWTLR	Deamidation N	2	De-novo
427,2116	2	852,4076	852,413	-0,0054	-6,3631	75,7576	113,6283	1	6	YDWTLR			De-novo
427,2116	2	852,4076	852,413	-0,0054	-6,3631	57,5758	107,5613	1	6	NYWTLR	Deamidation N	1	De-novo
579,6078	3	1735,8000	1735,8167	-0,0167	-9,6416	49,4624	164,7618	1	16	TANHPFSYLATNATAR	Deamidation N	3 / 12	De-novo
579,6078	3	1735,8000	1735,8167	-0,0167	-9,6416	48,2759	160,1375	1	15	TANHPFSYLATDNKR	Deamidation N	3 / 13	De-novo
579,6078	3	1735,8000	1735,8167	-0,0167	-9,6416	48,2759	160,9144	1	15	TANHPFSYLATNNKR	Deamidation N	3 / 12 / 1[illegible]	De-novo
617,3355	2	1232,6554	1232,6553	0,0001	0,0617	58,7302	101,7558	1	11	FLPTFANLGPR	Deamidation N	7	De-novo
617,3355	2	1232,6554	1232,6553	0,0001	0,0617	58,7302	100,5639	1	11	FLPTFADLGPR			De-novo
617,3355	2	1232,6554	1232,6553	0,0001	0,0617	58,7302	101,8218	1	11	LFPTFANLGPR	Deamidation N	7	De-novo

NCBI gi|156968291|gb|ABU98617.1| unknown [Helicoverpa armigera] — Total Score: 414

```
                       0        60       120      180      240
                       |         |        |        |        |    |267
gi|156968291|gb|ABU98  | ____________________________________________
Local hits (HSPs)      |               ____          __     __   ___
```

```
 Score = 104 (53.6 bits)
 Identities = 14/17 (82%), Positives = 15/17 (88%)

Query:   161 PGVVPSHVYFLETPRQA 177
             PGVV  HVYF+ETPRQA
Sbjct:   245 PGVVTMHVYFIETPRQA 261   MatchSeq
```

```
 Score = 98 (50.6 bits)
 Identities = 14/19 (73%), Positives = 16/19 (84%)

Query:   347 TARSFFSTADYLATNATAR 365
             T R +FSTADYLAT+AT R
Sbjct:    98 TIRAYFSTADYLATDATTR 116   MatchSeq
```

```
 Score = 88 (45.7 bits)
 Identities = 12/12 (100%), Positives = 12/12 (100%)

Query:    48 NYFESPGVAAVK 59
             NYFESPGVAAVK
Sbjct:   217 NYFESPGVAAVK 228   MatchSeq
```

```
 Score = 71 (37.2 bits)
 Identities = 10/10 (100%), Positives = 10/10 (100%)

Query:     1 TTSLECAEGR 10
             TTSLECAEGR
Sbjct:   176 TTSLECAEGR 185   MatchSeq
```

```
 Score = 53 (28.2 bits)
 Identities = 5/7 (71%), Positives = 7/7 (100%)

Query:    83 RNYWTLR 89
             R+YWT+R
Sbjct:    94 RDYWTIR 100   MatchSeq
```

Ha SG EST hargln|HA-GLN Contig 333 FWD frame 2 — Total Score: 414

```
                       0        70       140      210      280
                       |         |        |        |        |      |340
hargln|HA-GLN_Contig_  | ____________________________________________
Local hits (HSPs)      |                _____        _    __   __
```

```
 Score = 104 (53.6 bits)
 Identities = 14/17 (82%), Positives = 15/17 (88%)

Query:   161 PGVVPSHVYFLETPRQA 177
             PGVV  HVYF+ETPRQA
Sbjct:   285 PGVVTMHVYFIETPRQA 301
```

```
 Score = 98 (50.6 bits)
 Identities = 14/19 (73%), Positives = 16/19 (84%)

Query:   347 TARSFFSTADYLATNATAR 365
             T R +FSTADYLAT+AT R
Sbjct:   138 TIRAYFSTADYLATDATTR 156
```

```
 Score = 88 (45.7 bits)
 Identities = 12/12 (100%), Positives = 12/12 (100%)

Query:    48 NYFESPGVAAVK 59
             NYFESPGVAAVK
Sbjct:   257 NYFESPGVAAVK 268
```

```
 Score = 71 (37.2 bits)
 Identities = 10/10 (100%), Positives = 10/10 (100%)

Query:     1 TTSLECAEGR 10
             TTSLECAEGR
Sbjct:   216 TTSLECAEGR 225
```

```
 Score = 53 (28.2 bits)
 Identities = 5/7 (71%), Positives = 7/7 (100%)

Query:    83 RNYWTLR 89
             R+YWT+R
Sbjct:   134 RDYWTIR 140
```

Spot 12

m/z	Charge	Peak mW	Peptide mW	Delta (Da)	Delta (ppm)	Ladder Score	Log Likelihood	Start	End	Sequence	Modifications	Substitu	Query Tool
511,7333	2	1021,4510	1021,4716	-0,0206	-20,1905	31,3725	56,5252	1	9	TSLEQANYP			De-novo
706,8411	2	1411,6666	1411,6578	0,0088	6,2168	66,6667	196,984	1	13	TSWTTTLSNASSR	Deamidation N	9	De-novo
706,8411	2	1411,6666	1411,6578	0,0088	6,2168	66,6667	195,3977	1	13	TSWTTTLSDASSR			De-novo
706,8411	2	1411,6666	1411,6578	0,0088	6,2168	66,6667	195,6335	1	13	TSWTTTLSNTGSR	Deamidation N	9	De-novo
581,3063	2	1160,5970	1160,5978	-0,0008	-0,7100	72,549	123,4912	1	9	HVYFLETPR			De-novo
581,3063	2	1160,5970	1160,5978	-0,0008	-0,7100	72,549	121,7668	1	9	HVYFLQTPR	Deamidation Q	6	De-novo
581,3063	2	1160,5970	1160,5978	-0,0008	-0,7100	78,4314	121,2196	1	9	HVYFLTEPR			De-novo
427,211	2	852,4064	852,413	-0,0066	-7,7709	60,6061	120,0373	1	6	YNWTLR	Deamidation N	2	De-novo
427,211	2	852,4064	852,413	-0,0066	-7,7709	60,6061	118,7419	1	6	YDWTLR			De-novo
868,8758	2	1735,7360	1735,8013	-0,0653	-37,6333	52,381	373,7416	1	18	TANGPAGADYLATGVSNR	Deamidation N	3 / 17	De-novo
868,8758	2	1735,7360	1735,8013	-0,0653	-37,6333	52,381	367,0206	1	18	TANGPAGADYLATGVSDR	Deamidation N	3	De-novo
868,8758	2	1735,7360	1735,8013	-0,0653	-37,6333	46,6667	367,2816	1	18	TANGPAGADYLATGVNSR	Deamidation N	3 / 16	De-novo
763,3491	1	762,3413	762,3912	-0,0499	-65,4677	84,6154	177,8707	1	7	YNPAAVK	Deamidation N	2	De-novo
516,9179	3	1547,7303	1547,7037	0,0266	17,1635	45,679	128,6995	1	14	SGGVMYELHPQASR	Oxidation M / Deamidation	5 / 11	De-novo
516,9179	3	1547,7303	1547,7037	0,0266	17,1635	48,1481	128,3542	1	14	GSGVMYELHPQASR	Oxidation M / Deamidation	5 / 11	De-novo
516,9179	3	1547,7303	1547,7368	-0,0065	-4,2229	43,2099	126,4374	1	14	SGGVFYELHPQASR	Deamidation Q	11	De-novo
579,6091	3	1735,8039	1735,8167	-0,0128	-7,3948	52,6882	246,4822	1	16	FSNTAPHYLATNATAR	Deamidation N	3 / 12	De-novo
579,6091	3	1735,8039	1735,8167	-0,0128	-7,3948	52,6882	243,4831	1	16	FSNTAPHYLATDATAR	Deamidation N	3	De-novo
579,6091	3	1735,8039	1735,8167	-0,0128	-7,3948	50,5376	243,4613	1	16	SFNTAPHYLATNATAR	Deamidation N	3 / 12	De-novo
727,4163	2	1452,8170	1452,7765	0,0405	27,8611	60	63,2017	1	13	GLPYAQPPVLYHV			De-novo
727,4163	2	1452,8170	1452,7765	0,0405	27,8611	60	62,2374	1	13	GLPYAQPPVLYVH			De-novo
727,4163	2	1452,8170	1452,7765	0,0405	27,8611	60,4938	63,5271	1	14	GLPYAGAPPVLYHV			De-novo
532,8083	2	1063,6010	1063,6025	-0,0015	-1,4329	62,7451	60,7606	1	9	VVTVTLNYR			De-novo
512,7787	2	1023,5418	1023,5389	0,0029	2,8099	71,1111	81,0035	1	8	VYFLQTPR	Deamidation Q	5	De-novo
512,7787	2	1023,5418	1023,5389	0,0029	2,8099	71,1111	79,3487	1	8	VYFLETPR			De-novo
512,7787	2	1023,5418	1023,5389	0,0029	2,8099	71,1111	77,8869	1	8	VYFLTEPR			De-novo

NCBI gi|156968291|gb|ABU98617.1| unknown [Helicoverpa armigera] — Total Score: 209

```
                      0        60       120       180       240
                      |         |         |         |         |    |267
gi|156968291|gb|ABU98 |________________________________________________
Local hits (HSPs)     |            ______                        __
```

```
 Score = 79 (41.2 bits)
 Identities = 12/16 (75%), Positives = 12/16 (75%)

Query:    11 SWTTTLSNASSRTSWT 26
             SWTTTLSN  SR  WT
Sbjct:    83 SWTTTLSNGTSRDYWT 98   MatchSeq
```

```
 Score = 72 (37.7 bits)
 Identities = 8/10 (80%), Positives = 10/10 (100%)

Query:    49 HVYFLETPRH 58
             HVYF+ETPR+
Sbjct:   251 HVYFIETPRQ 260   MatchSeq
```

```
 Score = 58 (30.8 bits)
 Identities = 11/20 (55%), Positives = 12/20 (60%)

Query:   336 WTLRFSNTAPHYLATDATAR 355
             WT+R       YLATDAT R
Sbjct:    97 WTIRAYFSTADYLATDATTR 116   MatchSeq
```

Ha SG EST hargln|HA-GLN Contig 333 FWD frame 2 — Total Score: 216

```
                      0        70       140       210       280
                      |         |         |         |         |    |340
hargln|HA-GLN_Contig_ |________________________________________________
Local hits (HSPs)     |            ______                      _
```

```
 Score = 86 (44.7 bits)
 Identities = 13/17 (76%), Positives = 13/17 (76%)

Query:    10 TSWTTTLSNASSRTSWT 26
             TSWTTTLSN  SR  WT
Sbjct:   122 TSWTTTLSNGTSRDYWT 138
```

```
 Score = 72 (37.7 bits)
 Identities = 8/10 (80%), Positives = 10/10 (100%)

Query:    49 HVYFLETPRH 58
             HVYF+ETPR+
Sbjct:   291 HVYFIETPRQ 300
```

```
 Score = 58 (30.8 bits)
 Identities = 11/20 (55%), Positives = 12/20 (60%)

Query:   336 WTLRFSNTAPHYLATDATAR 355
             WT+R       YLATDAT R
Sbjct:   137 WTIRAYFSTADYLATDATTR 156
```

Spot 13

m/z	Charge	Peak mW	Peptide mW	Delta (Da)	Delta (ppm)	Ladder Score	Log Likelihood	Start	End	Sequence	Modifications	Subs	Query Tool
504,7465	2	1007,4774	1007,4924	-0,0150	-14,9123	68,6274	164,0297	1	9	VAYDVAENK			De-novo
504,7465	2	1007,4774	1007,4924	-0,0150	-14,9123	68,6274	160,3728	1	9	GLYDVAENK			De-novo
561,7786	2	1121,5416	1121,5353	0,0063	5,5959	72,549	139,9192	1	9	YTQSPELQR	Deamidation Q	3	De-novo
561,7786	2	1121,5416	1121,5353	0,0063	5,5959	72,549	138,5856	1	9	YTESPELQR			De-novo
561,7786	2	1121,5416	1121,5353	0,0063	5,5959	75,4386	140,7	1	10	YTQSPELGAR	Deamidation Q	3	De-novo
643,31	2	1284,6044	1284,5985	0,0059	4,5742	60,3175	76,4313	1	11	YYTESPALNAR	Deamidation N	9	De-novo
643,31	2	1284,6044	1284,5986	0,0058	4,4963	57,8947	73,4409	1	10	YYTESPEQLR			De-novo
448,2328	2	894,4500	894,4521	-0,0021	-2,3746	76,9231	162,6399	1	7	MVELPYK	Oxidation M	1	De-novo
448,2328	2	894,4500	894,4521	-0,0021	-2,3746	76,9231	162,5586	1	7	MVQLPYK	Oxidation M / Deamidation	1 / 3	De-novo
448,2328	2	894,4500	894,4521	-0,0021	-2,3746	76,9231	160,2654	1	7	VMELPYK	Oxidation M	2	De-novo
477,2578	2	952,5000	952,5018	-0,0018	-1,9150	52,9412	122,4924	1	9	QAGAFAFLK	Deamidation Q	1	De-novo
477,2578	2	952,5000	952,5018	-0,0018	-1,9150	49,0196	121,6124	1	9	AQGAFAFLK	Deamidation Q	2	De-novo
700,8687	2	1399,7218	1399,7195	0,0023	1,6260	40,7407	99,1313	1	14	LNLPEDAATGLGTK	Deamidation N	2	De-novo
762,9024	2	1523,7892	1523,8195	-0,0303	-19,9000	58,6207	288,937	1	15	QSLGPVPEALTVANK	Deamidation Q	1	De-novo
762,9024	2	1523,7892	1523,8195	-0,0303	-19,9000	58,6207	289,6949	1	15	SLEGPVPEALTVANK			De-novo
762,9024	2	1523,7892	1523,8195	-0,0303	-19,9000	58,6207	287,5676	1	15	QLSGPVPEALTVANK	Deamidation Q	1	De-novo
647,3439	2	1292,6722	1292,6725	-0,0003	-0,2506	69,8413	136,2209	1	11	LSLQDTFTVNR			De-novo
647,3439	2	1292,6722	1292,6725	-0,0003	-0,2506	69,5652	136,0517	1	12	LSLQDTFTAAAR			De-novo
647,3439	2	1292,6722	1292,6725	-0,0003	-0,2506	69,8413	136,3653	1	11	LSLQNTFTVNR	Deamidation N	5	De-novo
920,4623	2	1838,9090	1838,9414	-0,0324	-17,6319	60,2151	281,5841	1	16	TVQLLYYTESPELGAR			De-novo
920,4623	2	1838,9090	1838,9414	-0,0324	-17,6319	60,2151	281,5405	1	16	TVQLLYYTESPQLGAR	Deamidation Q	12	De-novo
920,4623	2	1838,9090	1838,9414	-0,0324	-17,6319	60,2151	280,9197	1	16	TVQLLYYTQSPELGAR	Deamidation Q	9	De-novo
543,2689	2	1084,5222	1084,5189	0,0033	3,0207	68,6274	93,7644	1	9	EAFFVSETR			De-novo
543,2689	2	1084,5222	1084,5189	0,0033	3,0207	60,7843	93,3973	1	9	AEFFVSETR			De-novo
543,2689	2	1084,5222	1084,5665	-0,0443	-40,8679	52,9412	92,7444	1	9	PHLFVSETR			De-novo
645,3584	2	1288,7012	1288,7026	-0,0014	-1,1050	63,4921	103,9227	1	11	SKLDLNDFLPK			De-novo
645,3584	2	1288,7012	1288,7026	-0,0014	-1,1050	63,4921	103,8615	1	11	KSLDLNDFLPK			De-novo
645,3584	2	1288,7012	1288,7026	-0,0014	-1,1050	63,4921	103,4588	1	11	KSLDLNNFLPK	Deamidation N	7	De-novo
756,9053	2	1511,7950	1511,7943	0,0007	0,4472	69,1358	277,2619	1	14	DAVSNLLANLTPER			De-novo
756,9053	2	1511,7950	1511,7943	0,0007	0,4472	69,1358	276,8684	1	14	ADVSNLLANLTPER			De-novo
756,9053	2	1511,7950	1511,7943	0,0007	0,4472	69,1358	276,0806	1	14	DAVSNLLANLTPQR	Deamidation Q	13	De-novo
446,7466	2	891,4776	891,4814	-0,0038	-4,2895	64,4444	41,2753	1	8	LFDALTGR			De-novo
446,7466	2	891,4776	891,4814	-0,0038	-4,2895	71,1111	40,4625	1	8	LFDALGTR			De-novo
446,7466	2	891,4776	891,4814	-0,0038	-4,2895	40	41,4869	1	8	LFDANGKK			De-novo
766,905	2	1531,7944	1531,7882	0,0062	4,0319	83,9506	422,0765	1	14	FSQLNPEALTVANK	Deamidation Q	3	De-novo
766,905	2	1531,7944	1531,7882	0,0062	4,0319	83,9506	418,8322	1	14	FSELNPEALTVANK			De-novo
766,905	2	1531,7944	1531,7882	0,0062	4,0319	83,9506	419,6934	1	14	FSQLNPQALTVANK	Deamidation Q	3 / 7	De-novo

NCBI gi|157786102|gb|ABV74209.1| serpin-6 [Bombyx mori] — Total Score: 323

```
                      0        90       180       270       360
                      |         |         |         |         |    |413
gi|157786102|gb|ABV74 |________________________________________________
Local hits (HSPs)     | _                        _ __  _
```

```
 Score = 81 (41.8 bits)
 Identities = 10/15 (66%), Positives = 13/15 (86%)

Query:   307 KDAVSNLLANLTPER 321
             K+ VSN++ NLTPER
Sbjct:   272 KEGVSNIITNLTPER 286   MatchSeq
```

```
 Score = 79 (40.8 bits)
 Identities = 10/13 (76%), Positives = 12/13 (92%)

Query:   487 VSNLLANLTPERL 499
             VSN++ NLTPERL
Sbjct:   275 VSNIITNLTPERL 287   MatchSeq
```

```
 Score = 68 (35.4 bits)
 Identities = 9/9 (100%), Positives = 9/9 (100%)

Query:   248 EAFFVSETR 256
             EAFFVSETR
Sbjct:   217 EAFFVSETR 225   MatchSeq
```

```
 Score = 60 (31.4 bits)
 Identities = 9/12 (75%), Positives = 9/12 (75%)

Query:   430 ELGARMVELPYK 441
             ELGA   ELPYK
Sbjct:   245 ELGAQILELPYK 256   MatchSeq
```

```
 Score = 35 (19.1 bits)
 Identities = 5/5 (100%), Positives = 5/5 (100%)

Query:   377 LNPEA 381
             LNPEA
Sbjct:    29 LNPEA 33   MatchSeq
```

Ha SG EST None

Ha EST other harmi_fn|HA-FN-ALL Contig 2734 FWD frame 1 — Total Score: 368

```
           Length = 346
                      0        70       140       210       280
                      |         |         |         |         |    |346
harmi_fn|HA-FN-ALL_Co | __________________________________________________
Local hits (HSPs)     |              _              __         __
```

```
 Score = 122 (62.0 bits)     Score = 98 (50.2 bits)      Score = 57 (29.9 bits)      Score = 48 (25.5 bits)
 Identities = 16/18 (88%), I Identities = 15/18 (83%), I Identities = 9/11 (81%), Pc Identities = 7/10 (70%), Positives = 7/10 (70%)
```

```
Query:   425 YTQSPELGARMVELPYKQ 442
             YT+SPELGARMVELPYK+
Sbjct:   247 YTESPELGARMVELPYKE 264
```

```
Query:   204 LYYTESPELGARTVQLLY 221
             LYYTESPELGAR V+L Y
Sbjct:   245 LYYTESPELGARMVELPY 262
```

```
Query:   275 SKLDLNDFLPK 285
             SKLD ND LPK
Sbjct:   317 SKLDFNDILPK 327
```

```
Query:   179 LQDTFTAAAR 188
             L D F AAAR
Sbjct:   143 LXDXFXAAAR 152
```

```
 Score = 43 (23.0 bits)
 Identities = 9/15 (60%), Positives = 10/15 (66%)

Query:   299 LDLNNFLPKDAVSNL 313
             LD N+ LPK  VS L
Sbjct:   319 LDFNDILPKLGVSKL 333
```

Spot 14

m/z	Charge	Peak mW	Peptide mW	Delta (Da)	Delta (ppm)	Ladder Score	Log Likelihood	Start	End	Sequence	Modifications	Subs	Query Tool
599,7594	2	1197,5032	1197,515	-0,0118	-9,8738	68,254	202,0504	1	11	SDYVGDEAQSK			De-novo
488,7268	2	975,4380	975,4409	-0,0029	-2,9976	56,1404	97,4857	1	10	GAFAGDNAPR	Deamidation N	7	De-novo
488,7268	2	975,4380	975,4409	-0,0029	-2,9976	56,1404	97,0767	1	10	GAFAGNDAPR	Deamidation N	6	De-novo
750,3326	2	1498,6496	1498,6624	-0,0128	-8,5570	48	204,5506	1	13	NYEHCSGPSLVHR	Deamidation N	1	De-novo
750,3326	2	1498,6496	1498,6624	-0,0128	-8,5570	48	203,7996	1	13	NYECHSGPSLVHR	Deamidation N	1	De-novo
750,3326	2	1498,6496	1498,6687	-0,0191	-12,7607	40	200,1666	1	13	NYEDDPGPSLVHR	Deamidation N	1	De-novo
514,8021	2	1027,5886	1027,5927	-0,0041	-4,0133	78,4314	198,7033	1	9	FPSLVGRPR			De-novo
926,9047	2	1851,7938	1851,7986	-0,0048	-2,6050	52,8736	195,9725	1	15	EEEPTALVYDDHFCK	Carbamidomethyl C	14	De-novo
566,7676	2	1131,5196	1131,5197	-0,0001	-0,1096	64,9123	120,0555	1	10	GYSFTTATER			De-novo
566,7676	2	1131,5196	1131,5197	-0,0001	-0,1096	64,9123	119,5519	1	10	YGSFTTATER			De-novo
566,7676	2	1131,5196	1131,5197	-0,0001	-0,1096	64,9123	119,001	1	10	GYSFTTATQR	Deamidation Q	9	De-novo
758,8565	2	1515,6974	1515,6953	0,0021	1,3697	55,5556	86,7697	1	14	AGEYNESGPLSVHR	Deamidation N	5	De-novo
758,8565	2	1515,6974	1515,6953	0,0021	1,3697	60	85,1498	1	13	QEYNESGPLSVHR	Deamidation N	4	De-novo
758,8565	2	1515,6974	1515,6953	0,0021	1,3697	55,5556	85,865	1	14	GAEYNESGPLSVHR	Deamidation N	5	De-novo
473,2795	2	944,5434	944,5444	-0,0010	-1,0841	52,9412	89,2759	1	9	GLFPSLVGR			De-novo
473,2795	2	944,5434	944,5444	-0,0010	-1,0841	52,9412	89,5922	1	9	AVFPSLVGR			De-novo
473,2795	2	944,5434	944,5444	-0,0010	-1,0841	52,9412	88,7052	1	9	LGFPSLVGR			De-novo

NCBI gi|157927725|gb|ABW03226.1| beta actin [Xestia c-nigrum] Total Score: 442

```
                          0         80        160       240       320
                          |         |         |         |         |      |376
gi|157927725|gb|ABW03  |  ______________________________________________
Local hits (HSPs)      |  _ __  _             __                  __
```

```
 Score = 75 (39.4 bits)
 Identities = 10/13 (76%), Positives = 11/13 (84%)

Query:   112 TERGYSFTTATQR 124
             TERGYSFTT  +R
Sbjct:   195 TERGYSFTTTAER 207   MatchSeq

 Score = 70 (36.9 bits)
 Identities = 9/10 (90%), Positives = 10/10 (100%)

Query:   174 RAVFPSLVGR 183
             RAVFPS+VGR
Sbjct:    29 RAVFPSIVGR 38   MatchSeq

 Score = 66 (34.9 bits)
 Identities = 9/9 (100%), Positives = 9/9 (100%)

Query:     3 YVGDEAQSK 11
             YVGDEAQSK
Sbjct:    54 YVGDEAQSK 62   MatchSeq

 Score = 65 (34.4 bits)
 Identities = 8/9 (88%), Positives = 9/9 (100%)

Query:    71 FPSLVGRPR 79
             FPS+VGRPR
Sbjct:    32 FPSIVGRPR 40   MatchSeq

 Score = 65 (34.4 bits)
 Identities = 10/13 (76%), Positives = 11/13 (84%)

Query:   139 QEYNESGPLSVHR 151
             QEY+ESGP  VHR
Sbjct:   361 QEYDESGPSIVHR 373   MatchSeq

 Score = 61 (32.4 bits)
 Identities = 9/14 (64%), Positives = 10/14 (71%)

Query:    11 KGAFAGDNAPRGAF 24
             K  FAGD+APR  F
Sbjct:    19 KAGFAGDDAPRAVF 32   MatchSeq

 Score = 40 (21.9 bits)
 Identities = 7/12 (58%), Positives = 9/12 (75%)

Query:    79 REEEPTALVYDD 90
             R+EE  ALV D+
Sbjct:     2 RDEEVAALVVDN 13   MatchSeq
```

Ha SG EST hargln|HA-GLN Contig 357 FWD frame 0 Total Score: 333

```
                          0         80        160       240       320
                          |         |         |         |         |      |396
hargln|HA-GLN_Contig_  |  ______________________________________________
Local hits (HSPs)      |     __    _          ___
```

```
 Score = 71 (37.4 bits)
 Identities = 10/13 (76%), Positives = 11/13 (84%)

Query:   112 TERGYSFTTATQR 124
             TERGYSFTT  +R
Sbjct:   232 TERGYSFTTMAER 244

 Score = 70 (36.9 bits)
 Identities = 9/10 (90%), Positives = 10/10 (100%)

Query:   174 RAVFPSLVGR 183
             RAVFPS+VGR
Sbjct:    66 RAVFPSIVGR 75

 Score = 66 (34.9 bits)
 Identities = 9/9 (100%), Positives = 9/9 (100%)

Query:     3 YVGDEAQSK 11
             YVGDEAQSK
Sbjct:    91 YVGDEAQSK 99

 Score = 65 (34.4 bits)
 Identities = 8/9 (88%), Positives = 9/9 (100%)

Query:    71 FPSLVGRPR 79
             FPS+VGRPR
Sbjct:    69 FPSIVGRPR 77

 Score = 61 (32.4 bits)
 Identities = 9/14 (64%), Positives = 10/14 (71%)

Query:    11 KGAFAGDNAPRGAF 24
             K  FAGD+APR  F
Sbjct:    56 KAGFAGDDAPRAVF 69
```

Spot 15

m/z	Charge	Peak mW	Peptide mW	Delta (Da)	Delta (ppm)	Ladder Score	Log Likelihood	Start	End	Sequence	Modifications	Subs	Query Tool
457,7862	2	913,5568	913,567	-0,0102	-11,1913	60	144,6849	1	8	MSLLLPLK			De-novo
457,7862	2	913,5568	913,567	-0,0102	-11,1913	60	141,9754	1	8	SMLLLPLK			De-novo
457,7862	2	913,5568	913,567	-0,0102	-11,1913	60	125,5256	1	8	MALLLPLK	Oxidation M	1	De-novo
449,7909	2	897,5662	897,5535	0,0127	14,1228	64,4444	104,2041	1	8	NSLLLPLK	Deamidation N	1	De-novo
449,7909	2	897,5662	897,5534	0,0128	14,2343	64,4444	103,2778	1	8	SNLLLPLK	Deamidation N	2	De-novo
449,7909	2	897,5662	897,5535	0,0127	14,1228	64,4444	103,4	1	8	DSLLLPLK			De-novo
441,7846	2	881,5536	881,5738	-0,0202	-22,9408	69,2308	125,0691	1	7	WLLLPLK			De-novo
441,7846	2	881,5536	881,5585	-0,0049	-5,5856	64,4444	112,9425	1	8	NALLLPLK	Deamidation N	1	De-novo
441,7846	2	881,5536	881,5585	-0,0049	-5,5856	60	112,8847	1	8	ANLLLPLK	Deamidation N	2	De-novo
470,3078	2	938,6000	938,5952	0,0048	5,0885	51,1111	84,5395	1	8	GWLLLPLK			De-novo
470,3078	2	938,6000	938,5952	0,0048	5,0885	53,3333	84,245	1	8	WGLLLPLK			De-novo
511,8395	2	1021,6634	1021,6535	0,0099	9,6667	50,9804	120,741	1	9	TQPLLLPLK			De-novo
511,8395	2	1021,6634	1021,6535	0,0099	9,6667	50,9804	120,4459	1	9	QTPLLLPLK			De-novo
511,8395	2	1021,6634	1021,6535	0,0099	9,6667	41,1765	111,0313	1	9	QPTLLLPLK			De-novo
897,9133	2	1793,8110	1793,8256	-0,0146	-8,1524	53,3333	120,5619	1	18	AAEMSGGDGLLSGWGNKK	Oxidation M / Deamidation	4 / 16	De-novo
897,9133	2	1793,8110	1793,8254	-0,0144	-8,0409	47,619	121,2325	1	18	AAEMSGGDGLLSGWNSVK	Oxidation M	4	De-novo
897,9133	2	1793,8110	1793,8254	-0,0144	-8,0409	47,619	120,0049	1	18	AAEMSGGDGLLSGWSNVK	Oxidation M	4	De-novo

NCBI gi|157017918|gb|EAL40833.3| AGAP002826-PA [Anopheles gambiae str. PEST] Total Score: 67

```
                          0         50        100       150       200
                          |         |         |         |         |   |213
gi|157017918|gb|EAL40  |  ______________________________________________
Local hits (HSPs)      |                                         _____
```

```
 Score = 67 (35.7 bits)
 Identities = 10/21 (47%), Positives = 14/21 (66%)

Query:    64 ANLLLPLKGWLLLPLKWGLLL 84
             AN LLP   WL++P  W L++
Sbjct:   186 ANMLLPALAWLIIPQSWNLII 206   MatchSeq
```

ButterflyBase None

Ha SG EST **None**

Ha EST other harmi_fn|HA-FN-ALL Contig 1305 FWD frame 0 Total Score: 178

```
                          0         60        120       180       240
                          |         |         |         |         |      |293
harmi_fn|HA-FN-ALL_Co  |  ______________________________________________
Local hits (HSPs)      |     _          __          _          __
```

```
 Score = 60 (32.1 bits)
 Identities = 9/12 (75%), Positives = 10/12 (83%)

Query:    77 PLKWGLLLPLKT 88
             PLKW L +PLKT
Sbjct:    37 PLKWSLPIPLKT 48

 Score = 41 (22.5 bits)
 Identities = 7/10 (70%), Positives = 8/10 (80%)

Query:   168 KMSLLLPLKM 177
             K SLL+P KM
Sbjct:   267 KSSLLIPSKM 276

 Score = 41 (22.5 bits)
 Identities = 5/6 (83%), Positives = 6/6 (100%)

Query:    12 LLPLKM 17
             L+PLKM
Sbjct:   119 LIPLKM 124

 Score = 36 (20.0 bits)
 Identities = 7/9 (77%), Positives = 7/9 (77%)

Query:    46 PLKWLLLPL 54
             PLKW LL L
Sbjct:   189 PLKWSLLTL 197
```

Spot 16

m/z	Charge	Peak mW	Peptide mW	Delta (Da)	Delta (ppm)	Ladder Score	Log Likelihood	Start	End	Sequence	Modifications	Subs	Query Tool
515,2904	2	1028,5652	1028,5614	0,0038	3,6711	80,9524	117,7528	1	11	VGAVGQALTGR	Deamidation Q	6	De-novo
515,2904	2	1028,5652	1028,5614	0,0038	3,6711	76,1905	116,8004	1	11	VGAVGQALASR	Deamidation Q	6	De-novo
515,2904	2	1028,5652	1028,5614	0,0038	3,6711	80,9524	116,7728	1	11	VGAVGEALTGR			De-novo
679,3755	2	1356,7354	1356,7362	-0,0008	-0,6073	60,9195	136,2653	1	15	SAGLVGAVGQALTGR	Deamidation Q	10	De-novo
679,3755	2	1356,7354	1356,7362	-0,0008	-0,6073	58,6207	135,6414	1	15	GASLVGAVGQALTGR	Deamidation Q	10	De-novo
679,3755	2	1356,7354	1356,7362	-0,0008	-0,6073	60,9195	135,4418	1	15	SGALVGAVGQALTGR	Deamidation Q	10	De-novo
441,7918	2	881,5680	881,5585	0,0095	10,7491	57,7778	66,4382	1	8	NALLLPLK	Deamidation N	1	De-novo
441,7918	2	881,5680	881,5738	-0,0058	-6,6064	58,9744	71,8215	1	7	LWLLPLK			De-novo
441,7918	2	881,5680	881,5585	0,0095	10,7491	53,3333	65,7705	1	8	ANLLLPLK	Deamidation N	2	De-novo
568,8087	2	1135,6018	1135,6713	-0,0695	-61,2184	33,3333	92,718	1	11	AAPPTKVSLPR			De-novo
568,8087	2	1135,6018	1135,6349	-0,0331	-29,1678	44,9275	92,3864	1	12	AAPPTAGVSLPR			De-novo
568,8087	2	1135,6018	1135,6349	-0,0331	-29,1678	39,6825	91,3954	1	11	AAPPTQVSLPR			De-novo
401,2487	2	800,4818	800,483	-0,0012	-1,5291	43,5897	82,4275	1	7	MSLLPLK			De-novo
401,2487	2	800,4818	800,4796	0,0022	2,7184	35,8974	82,1111	1	7	PAYLPLK			De-novo
401,2487	2	800,4818	800,4796	0,0022	2,7184	46,1538	82,3939	1	7	YPALPLK			De-novo

NCBI gi|254763552|gb|ACT80221.1| ATP synthase F0 subunit 6 [Mayetiola destructor] Total Score: 146

```
                          0         50        100       150       200
                          |         |         |         |         |   |223
gi|254763552|gb|ACT80  |  ______________________________________________
Local hits (HSPs)      |     ___                     _
```

```
 Score = 58 (31.5 bits)
 Identities = 6/10 (60%), Positives = 9/10 (90%)

Query:    82 LLPLKLWLLP 91
             L+PLK W++P
Sbjct:    30 LIPLKFWIIP 39   MatchSeq

 Score = 57 (31.0 bits)
 Identities = 6/12 (50%), Positives = 9/12 (75%)

Query:   153 LPLKLWLLPLKM 164
             +PLK W++P  M
Sbjct:    31 IPLKFWIIPNNM 42   MatchSeq

 Score = 31 (17.7 bits)
 Identities = 3/5 (60%), Positives = 5/5 (100%)

Query:    95 NLLLP 99
             N+L+P
Sbjct:   136 NILIP 140   MatchSeq
```

ButterflyBase None

Ha SG EST hargln|HA-GLN_Contig_2225_FWD_frame_1 Total Score: 118

```
                          0         40        80        120       160
                          |         |         |         |         |      |182
hargln|HA-GLN_Contig_  |  ______________________________________________
Local hits (HSPs)      |  ____
```

```
 Score = 64 (34.6 bits)
 Identities = 10/15 (66%), Positives = 12/15 (80%)

Query:    18 ALASRVGAVGEALTG 32
             A+ S V AVGEA+TG
Sbjct:     1 AIGSNVNAVGEAITG 15

 Score = 54 (29.5 bits)
 Identities = 8/12 (66%), Positives = 10/12 (83%)

Query:    38 VGAVGQALTGRG 49
             V AVG+A+TG G
Sbjct:     6 VNAVGEAITGKG 17
```

Spot 17

m/z	Charge	Peak mW	Peptide mW	Delta (Da)	Delta (ppm)	Ladder Score	Log Likelihood	Start	End	Sequence	Modifications	Subs	Query Tool
504,7497	2	1007,4838	1007,4924	-0,0086	-8,5599	68,6274	181,4954	1	9	VAYDVAENK			De-novo
504,7497	2	1007,4838	1007,4923	-0,0085	-8,4606	68,6274	181,2792	1	9	VAYNVAENK	Deamidation N	4	De-novo
504,7497	2	1007,4838	1007,4923	-0,0085	-8,4606	56,8627	178,4553	1	9	VANYVAENK	Deamidation N	3	De-novo
495,7578	2	989,5000	989,5029	-0,0029	-2,9550	72,549	97,4351	1	9	TQLGESLNK	Deamidation N	8	De-novo
495,7578	2	989,5000	989,5029	-0,0029	-2,9550	64,9123	97,1761	1	10	TGALGESLNK	Deamidation N	9	De-novo
440,2345	2	878,4534	878,4572	-0,0038	-4,3531	41,0256	85,2565	1	7	EVMLPYK			De-novo
440,2345	2	878,4534	878,4572	-0,0038	-4,3531	46,1538	84,5344	1	7	VEMLPYK			De-novo
440,2345	2	878,4534	878,4572	-0,0038	-4,3531	41,0256	84,7568	1	7	QVMLPYK	Deamidation Q	1	De-novo
512,7764	2	1023,5372	1023,5389	-0,0017	-1,6844	51,1111	71,5883	1	8	VYFLETPR			De-novo
512,7764	2	1023,5372	1023,5389	-0,0017	-1,6844	51,1111	71,9879	1	8	VYFLQTPR	Deamidation Q	5	De-novo
868,9062	2	1735,7968	1735,8014	-0,0046	-2,6639	56,1905	322,2256	1	18	TADGPAGADYLATNATAR	Deamidation N	14	De-novo
868,9062	2	1735,7968	1735,8014	-0,0046	-2,6639	56,1905	318,9089	1	18	TADGPAGADYLATDATAR			De-novo
920,4808	2	1838,9460	1838,9414	0,0046	2,4884	59,1398	280,7038	1	16	TNLLLYYTESPELGAR			De-novo
920,4808	2	1838,9460	1838,9414	0,0046	2,4884	58,6207	279,1359	1	15	TNLLLYYTESPELQR			De-novo
920,4808	2	1838,9460	1838,9414	0,0046	2,4884	58,6207	277,8062	1	15	TNLLLYYTESPQLQR	Deamidation Q	12	De-novo
969,5344	2	1937,0532	1937,0543	-0,0011	-0,5803	49,5238	182,7192	1	18	MVVVLPNEVDGLPSVLEK			De-novo
969,5344	2	1937,0532	1937,0543	-0,0011	-0,5803	49,5238	181,6231	1	18	VMVVLPNEVDGLPSVLEK			De-novo
561,7767	2	1121,5378	1121,5353	0,0025	2,2077	62,7451	139,1263	1	9	YTESPELQR			De-novo
561,7767	2	1121,5378	1121,5353	0,0025	2,2077	62,7451	139,0616	1	9	YTQSPELQR	Deamidation Q	3	De-novo
561,7767	2	1121,5378	1121,5353	0,0025	2,2077	62,7451	137,4372	1	9	YTESPQLQR	Deamidation Q	6	De-novo
762,8991	2	1523,7826	1523,8195	-0,0369	-24,2312	62,069	248,8545	1	15	SLEGPVPEALTVANK			De-novo
762,8991	2	1523,7826	1523,8195	-0,0369	-24,2312	57,4713	248,1613	1	15	LSEGPVPEALTVANK			De-novo
762,8991	2	1523,7826	1523,8195	-0,0369	-24,2312	59,7701	246,2407	1	15	ESLGPVPEALTVANK			De-novo
766,9016	2	1531,7876	1531,7883	-0,0007	-0,4727	37,037	107,5385	1	14	ESFLNPEAVNATLK			De-novo
766,9016	2	1531,7876	1531,7883	-0,0007	-0,4727	37,037	107,457	1	14	SEFLNPEAVNATLK			De-novo
766,9016	2	1531,7876	1531,7883	-0,0007	-0,4727	34,5679	107,4299	1	14	DTFLNPEAVNATLK			De-novo

NCBI gi|14028769|gb|AAK52495.1|AF361483_1 chymotrypsin inhibitor CI-8A [Bombyx mori] Total Score: 292

```
                    0        80       160      240      320
                    |        |        |        |        |       |395
gi|14028769|gb|AAK524  |________________________________________
Local hits (HSPs)      |  _ _       _          __  __
```

```
Score = 107 (54.0 bits)
Identities = 15/19 (78%), Positives = 17/19 (89%)

Query:   165 RMVVVLPNEVDGLPSVLEK 183
             RMVVVLP+ +DGLP VLEK
Sbjct:   249 RMVVVLPDKIDGLPAVLEK 267   MatchSeq
```

```
Score = 65 (33.5 bits)
Identities = 9/14 (64%), Positives = 11/14 (78%)

Query:   336 LYYTESPELGARMV 349
               L+YTE  ELGA M+
Sbjct:   226 LFYTENEELGAKMI 239   MatchSeq
```

```
Score = 45 (23.8 bits)
Identities = 6/7 (85%), Positives = 7/7 (100%)

Query:   238 LTVANKL 244
             LTVANK+
Sbjct:   110 LTVANKI 116   MatchSeq
```

```
Score = 39 (20.8 bits)
Identities = 5/10 (50%), Positives = 8/10 (80%)

Query:    28 TQLGESLNKT 37
             T LG+ ++KT
Sbjct:    27 TRLGDAIDKT 36   MatchSeq
```

```
Score = 36 (19.4 bits)
Identities = 5/10 (50%), Positives = 7/10 (70%)

Query:     9 KVAYNVAENK 18
             K  YN A++K
Sbjct:    42 KESYNLADDK 51   MatchSeq
```

Ha SG EST hargln|HA-GLN_Contig_333_FWD_frame_2 Total Score: 166

```
                    0        70       140      210      280
                    |        |        |        |        |       |340
hargln|HA-GLN_Contig_  |________________________________________
Local hits (HSPs)      |                _                __ _
```

```
Score = 70 (36.0 bits)
Identities = 10/11 (90%), Positives = 10/11 (90%)

Query:   109 ADYLATDATAR 119
             ADYLATDAT R
Sbjct:   146 ADYLATDATTR 156
```

```
Score = 62 (32.1 bits)
Identities = 7/8 (87%), Positives = 8/8 (100%)

Query:    68 VYFLETPR 75
             VYF+ETPR
Sbjct:   292 VYFIETPR 299
```

```
Score = 34 (18.4 bits)
Identities = 4/6 (66%), Positives = 5/6 (83%)

Query:     3 YDVAEN 8
             Y+ AEN
Sbjct:   279 YELAEN 284
```

Spot 18

m/z	Charge	Peak mW	Peptide mW	Delta (Da)	Delta (ppm)	Ladder Score	Log Likelihood	Start	End	Sequence	Modifications	Subs	Query Tool
574,2322	2	1146,4488	1146,5227	-0,0739	-64,4767	45,614	195,3443	1	10	NLNPDVEAMK	Deamidation N / Oxidation	1 / 9	De-novo
574,2322	2	1146,4488	1146,5227	-0,0739	-64,4767	45,614	195,2988	1	10	LNNPDVEAMK	Deamidation N / Oxidation	2 / 9	De-novo
702,8122	2	1403,6088	1403,6527	-0,0439	-31,2926	74,6667	294,8022	1	13	NVSNGGELTVEQR	Deamidation N /	4 / 12	De-novo
702,8122	2	1403,6088	1403,6527	-0,0439	-31,2926	74,6667	294,4644	1	13	NVSNGGELTVEER	Deamidation N	4	De-novo
702,8122	2	1403,6088	1403,6527	-0,0439	-31,2926	74,6667	293,6093	1	13	NVSNGGELTVQER	Deamidation N /	4 / 11	De-novo
521,3066	2	1040,5976	1040,5978	-0,0002	-0,2153	63,1579	129,5565	1	10	VQATPLSVAR			De-novo
521,3066	2	1040,5976	1040,5978	-0,0002	-0,2153	71,9298	125,8058	1	10	VQATPLVSAR			De-novo
521,3066	2	1040,5976	1040,5978	-0,0002	-0,2153	63,1579	124,4689	1	10	VQATPLASVR			De-novo
628,7892	2	1255,5628	1255,5833	-0,0205	-16,3462	71,4286	257,5373	1	11	YLAEFATGNNR	Deamidation N	10	De-novo
628,7892	2	1255,5628	1255,5833	-0,0205	-16,3462	71,4286	256,1555	1	11	YLAEFATGNDR			De-novo
628,7892	2	1255,5628	1255,5833	-0,0205	-16,3462	71,4286	253,131	1	11	YLAQFATGNDR	Deamidation Q	4	De-novo
682,3422	2	1362,6688	1362,7184	-0,0496	-36,4154	52,1739	48,8421	1	12	WFASDLLVVDAK			De-novo
682,3422	2	1362,6688	1362,7184	-0,0496	-36,4154	49,2754	48,2006	1	12	FWASDLLVVDAK			De-novo
440,7489	2	879,4822	879,4814	0,0008	0,8823	50,8772	69,6824	1	10	PAAPSAPAAK			De-novo
440,7489	2	879,4822	879,4814	0,0008	0,8823	47,3684	69,1513	1	10	APAPSAPAAK			De-novo
440,7489	2	879,4822	879,4814	0,0008	0,8823	54,386	67,7325	1	10	APAPSAAPAK			De-novo
696,8156	2	1391,6156	1391,6204	-0,0048	-3,4665	60,4938	63,4117	1	14	LSEASQADAPGFEA			De-novo
696,8156	2	1391,6156	1391,6204	-0,0048	-3,4665	60,4938	64,0017	1	14	LSEASQANAPGFEA	Deamidation N	8	De-novo
696,8156	2	1391,6156	1391,6204	-0,0048	-3,4665	60,4938	63,7931	1	14	LSEASQADAPGFQA	Deamidation Q	13	De-novo
642,2968	2	1282,5780	1282,6517	-0,0737	-57,4778	60,8696	145,4049	1	12	HLLPSSQTGESK			De-novo
642,2968	2	1282,5780	1282,6517	-0,0737	-57,4778	60,8696	143,2524	1	12	HLLPSSQTEGSK			De-novo
642,2968	2	1282,5780	1282,6517	-0,0737	-57,4778	60,8696	143,5743	1	12	HLLPSSQTGQSK	Deamidation Q	10	De-novo
597,8052	2	1193,5948	1193,5928	0,0020	1,6555	50,7937	123,9941	1	11	QAAENSLVAYK	Deamidation Q	1	De-novo
597,8052	2	1193,5948	1193,5928	0,0020	1,6555	50,7937	124,3264	1	11	EAAENSLVAYK			De-novo
597,8052	2	1193,5948	1193,5928	0,0020	1,6555	50,7937	121,79	1	11	QAAQNSLVAYK	Deamidation Q	1 / 4	De-novo
566,238	2	1130,4604	1130,4624	-0,0020	-1,7904	66,6667	145,3766	1	9	NEYMVEAMK	Deamidation N / Oxidation	1 / 8	De-novo
566,238	2	1130,4604	1130,4624	-0,0020	-1,7904	66,6667	145,7312	1	9	ENYMVEAMK	Deamidation N / Oxidation	2 / 8	De-novo
566,238	2	1130,4604	1130,4624	-0,0020	-1,7904	66,6667	144,7299	1	9	QNYMVEAMK	Deamidation Q /	1 / 2 / 8	De-novo

NCBI gi|189086228|dbj|BAG38534.1| 14-3-3 epsilon [Bombyx mori] Total Score: 398

```
                    0        60       120      180      240
                    |        |        |        |        |   |262
gi|189086228|dbj|BAG3  |________________________________________
Local hits (HSPs)      |   _ ___        ____  ____
```

```
Score = 89 (45.8 bits)
Identities = 12/12 (100%), Positives = 12/12 (100%)

Query:   100 RYLAEFATGNDR 111
             RYLAEFATGNDR
Sbjct:   133 RYLAEFATGNDR 144   MatchSeq
```

```
Score = 86 (44.3 bits)
Identities = 13/14 (92%), Positives = 13/14 (92%)

Query:   265 KEAAENSLVAYKQA 278
             KEAAENSLVAYK A
Sbjct:   145 KEAAENSLVAYKAA 158   MatchSeq
```

```
Score = 72 (37.4 bits)
Identities = 10/11 (90%), Positives = 11/11 (100%)

Query:   220 LLPSSQTGESK 230
             L+PSSQTGESK
Sbjct:   111 LIPSSQTGESK 121   MatchSeq
```

```
Score = 59 (31.0 bits)
Identities = 8/8 (100%), Positives = 8/8 (100%)

Query:    40 ELTVEERN 47
             ELTVEERN
Sbjct:    39 ELTVEERN 46   MatchSeq
```

```
Score = 54 (28.5 bits)
Identities = 7/7 (100%), Positives = 7/7 (100%)

Query:    16 VEAMKNV 22
             VEAMKNV
Sbjct:    24 VEAMKNV 30   MatchSeq
```

```
Score = 38 (20.6 bits)
Identities = 5/5 (100%), Positives = 5/5 (100%)

Query:   284 VAYKN 288
             VAYKN
Sbjct:    50 VAYKN 54   MatchSeq
```

Ha SG EST hargln|HA-GLN_Contig_470_FWD_frame_1 Total Score: 210

```
                    0        50       100      150      200
                    |        |        |        |        |      |230
hargln|HA-GLN_Contig_  |________________________________________
Local hits (HSPs)      |          __               ___
```

```
Score = 80 (41.4 bits)
Identities = 12/12 (100%), Positives = 12/12 (100%)

Query:   156 KAPAPSAPAAKA 167
             KAPAPSAPAAKA
Sbjct:   155 KAPAPSAPAAKA 166
```

```
Score = 75 (38.9 bits)
Identities = 11/11 (100%), Positives = 11/11 (100%)

Query:   314 KVQATPLSVAR 324
             KVQATPLSVAR
Sbjct:    62 KVQATPLSVAR 72
```

```
Score = 55 (29.0 bits)
Identities = 9/13 (69%), Positives = 10/13 (76%)

Query:   327 APSAPAAKEAAEN 339
             APSAPAAK   E+
Sbjct:   158 APSAPAAKAQEED 170
```

Spot 19

m/z	Charge	Peak mW	Peptide mW	Delta (Da)	Delta (ppm)	Ladder Score	Log Likelihood	Start	End	Sequence	Modifications	Subs	Query Tool
622,3129	2	1242,6102	1242,6091	0,0011	0,8659	72,4638	213,6675	1	12	ALDSDPNAQALK	Deamidation N	7	De-novo
622,3129	2	1242,6102	1242,6091	0,0011	0,8659	72,4638	212,7249	1	12	ALNSDPDAQALK	Deamidation N	3	De-novo
622,3129	2	1242,6102	1242,6091	0,0011	0,8659	72,4638	213,5719	1	12	LADSDPNAQALK	Deamidation N	7	De-novo
530,2894	2	1058,5632	1058,5608	0,0024	2,2446	62,7451	171,9189	1	9	ELELTQGLR	Deamidation Q	6	De-novo
530,2894	2	1058,5632	1058,5608	0,0024	2,2446	62,7451	170,3768	1	9	ELELTEGLR			De-novo
530,2894	2	1058,5632	1058,5608	0,0024	2,2446	62,7451	168,8628	1	9	QLELTQGLR	Deamidation Q	1 / 6	De-novo
671,344	2	1340,6724	1340,6724	0,0000	-0,0179	69,8413	98,3329	1	11	DAYTVLLNNYR			De-novo
671,344	2	1340,6724	1340,6724	0,0000	-0,0179	69,8413	95,969	1	11	NAYTVLLNNYR	Deamidation N	1	De-novo
671,344	2	1340,6724	1340,6724	0,0000	-0,0179	69,8413	96,7364	1	11	ADYTVLLNNYR			De-novo
784,8812	2	1567,7468	1567,7438	0,0030	1,8983	79,0123	262,1681	1	14	VVMNEDLGLASEFK	Oxidation M / Deamidation	3 / 4	De-novo
784,8812	2	1567,7468	1567,7439	0,0029	1,8345	79,0123	260,9808	1	14	VVMDENLGLASEFK	Oxidation M / Deamidation	3 / 6	De-novo
784,8812	2	1567,7468	1567,7439	0,0029	1,8345	79,0123	257,8127	1	14	VVMDEDLGLASEFK	Oxidation M	3	De-novo
746,3963	2	1490,7770	1490,7728	0,0042	2,8012	48,1481	89,4414	1	14	GAYTLGLEANDVLR			De-novo
746,3963	2	1490,7770	1490,7728	0,0042	2,8012	48,1481	90,5229	1	14	GAYTLGLQANDVLR	Deamidation Q	8	De-novo
746,3963	2	1490,7770	1490,7728	0,0042	2,8012	45,679	88,8698	1	14	GATYLGLEANDVLR			De-novo
605,3593	2	1208,7030	1208,7017	0,0013	1,0557	57,1429	237,3193	1	11	TADPLLLPELK			De-novo
605,3593	2	1208,7030	1208,7015	0,0015	1,2211	55,5556	235,8631	1	11	ATDPLLLPELK			De-novo
605,3593	2	1208,7030	1208,7015	0,0015	1,2211	57,1429	234,2601	1	11	TANPLLLPELK	Deamidation N	3	De-novo
562,3125	2	1122,6094	1122,6073	0,0021	1,8493	66,6667	97,6094	1	9	LFTPLETFR			De-novo
562,3125	2	1122,6094	1122,6073	0,0021	1,8493	66,6667	97,7059	1	9	LFTPLQTFR	Deamidation Q	6	De-novo
562,3125	2	1122,6094	1122,6073	0,0021	1,8493	50,9804	93,0104	1	9	LFTPLTEFR			De-novo
412,7526	2	823,4896	823,4916	-0,0020	-2,4578	55,5556	93,0003	1	8	VPGVSLPR			De-novo
412,7526	2	823,4896	823,4916	-0,0020	-2,4578	55,5556	91,791	1	8	VGPVSLPR			De-novo
482,7578	2	963,5000	963,5026	-0,0026	-2,7234	64,4444	167,3272	1	8	NLQVTDFK			De-novo
672,3405	2	1342,6654	1342,6954	-0,0300	-22,3610	39,6825	74,2953	1	11	YTSTVLLMFPR	Oxidation M	8	De-novo
672,3405	2	1342,6654	1342,6954	-0,0300	-22,3610	42,8571	74,0257	1	11	TYSTVLLMFPR	Oxidation M	8	De-novo
672,3405	2	1342,6654	1342,6954	-0,0300	-22,3610	39,6825	73,2874	1	11	YTSTVLLMYPR			De-novo
744,3622	2	1486,7088	1486,7052	0,0036	2,4053	55,1724	171,6909	1	15	FANGAYSASTTALGR	Deamidation N	3	De-novo
744,3622	2	1486,7088	1486,7052	0,0036	2,4053	57,4713	170,2832	1	15	AFNGAYSASTTALGR	Deamidation N	3	De-novo
744,3622	2	1486,7088	1486,7053	0,0035	2,3381	55,1724	167,2216	1	15	FADGAYSASTTALGR			De-novo

NCBI gi|726332|gb|AAA68242.1| juvenile hormone binding protein [Heliothis virescens] Total Score: 224

```
                     0      50     100    150    200
                     |      |      |      |      |     |242
gi|726332|gb|AAA68242  | ______________________________________
Local hits (HSPs)      |        ____ __      __
```

```
Score = 78 (40.3 bits)
Identities = 10/14 (71%), Positives = 12/14 (85%)

Query:   189 ELKATDPLLLPELK 202
             E+KA DPL +PELK
Sbjct:    54 EIKAIDPLVIPELK 67   MatchSeq
```

```
Score = 62 (32.4 bits)
Identities = 10/17 (58%), Positives = 11/17 (64%)

Query:   108 EFKVVMDENLGLASEFK 124
             E KVV DE  GL  +FK
Sbjct:    65 ELKVVVDESMGLVFDFK 81   MatchSeq
```

```
Score = 49 (26.0 bits)
Identities = 9/13 (69%), Positives = 9/13 (69%)

Query:   314 FNGAYSASTTALG 326
             FNG Y A  TALG
Sbjct:   130 FNGPYIAKATALG 142   MatchSeq
```

```
Score = 35 (19.1 bits)
Identities = 5/8 (62%), Positives = 6/8 (75%)

Query:   257 NLQVTDFK 264
             N Q+ DFK
Sbjct:    90 NQQISDFK 97   MatchSeq
```

Ha SG EST hargln|HA-GLN Contig 477 FWD frame 1 Total Score: 223

```
                      0      90     180    270    360
                      |      |      |      |      |     |442
hargln|HA-GLN_Contig_  | ______________________________________
Local hits (HSPs)      |              _     __  _
```

```
Score = 68 (35.4 bits)
Identities = 10/10 (100%), Positives = 10/10 (100%)

Query:    45 RELELTEGLR 54
             RELELTEGLR
Sbjct:   227 RELELTEGLR 236
```

```
Score = 66 (34.4 bits)
Identities = 8/9 (88%), Positives = 9/9 (100%)

Query:   232 LFTPLTEFR 240
             LFTP+TEFR
Sbjct:   170 LFTPITEFR 178
```

```
Score = 50 (26.5 bits)
Identities = 8/10 (80%), Positives = 8/10 (80%)

Query:   138 KGAYTLGLEA 147
             K  YTLGLEA
Sbjct:   259 KAGYTLGLEA 268
```

```
Score = 39 (21.1 bits)
Identities = 5/13 (38%), Positives = 9/13 (69%)

Query:   250 GPVSLPRNLQVTD 262
             G + +PR L+ T+
Sbjct:   221 GQITIPRELELTE 233
```

Spot 20

m/z	Charge	Peak mW	Peptide mW	Delta (Da)	Delta (ppm)	Ladder Score	Log Likelihood	Start	End	Sequence	Modifications	Subs	Query Tool
622,2836	2	1242,5516	1242,6091	-0,0575	-46,2929	71,0145	333,9293	1	12	ALNSDPDAQALK	Deamidation N	3	De-novo
622,2836	2	1242,5516	1242,6091	-0,0575	-46,2929	71,0145	333,8863	1	12	LANSDPDAQALK	Deamidation N	3	De-novo
622,2836	2	1242,5516	1242,6091	-0,0575	-46,2929	71,0145	332,8017	1	12	ALDSDPDAQALK			De-novo
744,3436	2	1486,6716	1486,7053	-0,0337	-22,6837	67,8161	335,4686	1	15	FADGAYSASTTALGR			De-novo
744,3436	2	1486,6716	1486,7053	-0,0337	-22,6837	72,4138	251,0926	1	15	FADGAYSASTTALGR			De-novo
744,3436	2	1486,6716	1486,7053	-0,0337	-22,6837	72,4138	251,0926	1	15	FADGAYSASTTALGR			De-novo
744,3436	2	1486,6716	1486,7053	-0,0337	-22,6837	65,5172	334,0421	1	15	AFDGAYSASTTALGR			De-novo
744,3436	2	1486,6716	1486,7052	-0,0336	-22,6165	67,8161	331,6193	1	15	FANGAYSASTTALGR	Deamidation N	3	De-novo
744,3436	2	1486,6716	1486,7052	-0,0336	-22,6165	72,4138	257,1213	1	15	FANGAYSASTTALGR	Deamidation N	3	De-novo
744,3436	2	1486,6716	1486,7052	-0,0336	-22,6165	72,4138	257,1213	1	15	FANGAYSASTTALGR			De-novo
585,2795	2	1168,5434	1168,5547	-0,0113	-9,6906	74,6032	124,0495	1	11	TGNALFTACSK	Carbamidomethyl C	9	De-novo
585,2795	2	1168,5434	1168,5547	-0,0113	-9,6906	63,7681	121,6892	1	12	TGGGALFTACSK	Carbamidomethyl C	10	De-novo
585,2795	2	1168,5434	1168,5547	-0,0113	-9,6906	68,254	119,0281	1	11	TGNALFATCSK	Carbamidomethyl C	9	De-novo
597,1892	2	1192,3628	1192,5765	-0,2137	-179,2120	61,4035	149,7063	1	10	SFEKGYSFTK			De-novo
597,1892	2	1192,3628	1192,5402	-0,1774	-148,7782	47,619	149,5695	1	11	SFEGAGYSFTK			De-novo
597,1892	2	1192,3628	1192,5765	-0,2137	-179,2120	61,4035	147,7497	1	10	SFQKGYSFTK	Deamidation Q	3	De-novo
621,8596	2	1241,7036	1241,6979	0,0057	4,5712	69,8413	134,6626	1	11	QVLNLSNLNVK	Deamidation N	7	De-novo
621,8596	2	1241,7036	1241,6979	0,0057	4,5712	66,6667	132,8712	1	11	NLLNLSNLNVK	Deamidation N	7	De-novo
621,8596	2	1241,7036	1241,6979	0,0057	4,5712	63,7681	133,4712	1	12	GGLLNLSNLNVK	Deamidation N	8	De-novo
1028,5138	2	2055,0120	2055,0195	-0,0075	-3,6613	58,5586	382,5651	1	19	CEVLVEEPNVVLGDNLAGK	Carbamidomethyl C /	1 / 15	De-novo
1028,5138	2	2055,0120	2055,0195	-0,0075	-3,6613	58,5586	382,5209	1	19	CQVLVEEPNVVLGDNLAGK	Carbamidomethyl C /	1 / 2 / 15	De-novo
1028,5138	2	2055,0120	2055,0195	-0,0075	-3,6613	58,5586	384,1918	1	19	CELVVEEPNVVLGDNLAGK	Carbamidomethyl C /	1 / 15	De-novo
747,936	2	1493,8564	1493,8527	0,0037	2,4608	76	206,6625	1	13	KMPTDPLLLPELK			De-novo
747,936	2	1493,8564	1493,8527	0,0037	2,4608	74,6667	206,5798	1	13	MKPTDPLLLPELK			De-novo
747,936	2	1493,8564	1493,8527	0,0037	2,4608	76	208,4613	1	13	KMPTNPLLLPELK	Deamidation N	5	De-novo
461,8013	2	921,5870	921,5898	-0,0028	-3,0643	77,7778	144,7115	1	8	LPLLPELK			De-novo
461,8013	2	921,5870	921,5898	-0,0028	-3,0643	82,2222	140,398	1	8	PLLLPELK			De-novo
601,3159	2	1200,6162	1200,6179	-0,0017	-1,4359	61,4035	186,5367	1	10	FEFLLAPEVH			De-novo
601,3159	2	1200,6162	1200,6179	-0,0017	-1,4359	64,9123	187,2729	1	10	EFFLLAPEVH			De-novo
601,3159	2	1200,6162	1200,6179	-0,0017	-1,4359	64,9123	185,2064	1	10	QFFLLAPEVH	Deamidation Q	1	De-novo
534,2369	2	1066,4582	1066,4607	-0,0025	-2,3667	54,902	174,2813	1	9	SYDGYSFTK			De-novo
534,2369	2	1066,4582	1066,4794	-0,0212	-19,9010	62,7451	174,5816	1	9	SFMGYSFTK			De-novo
534,2369	2	1066,4582	1066,4607	-0,0025	-2,3667	54,902	173,2928	1	9	SYNGYSFTK	Deamidation N	3	De-novo
578,2799	2	1154,5442	1154,5277	0,0165	14,2708	61,4035	196,9833	1	10	QEALFTACSK	Deamidation Q /	1 / 8	De-novo
578,2799	2	1154,5442	1154,5278	0,0164	14,1842	61,4035	196,8183	1	10	EEALFTACSK	Carbamidomethyl C	8	De-novo
578,2799	2	1154,5442	1154,5277	0,0165	14,2708	61,4035	192,5853	1	10	QQALFTACSK	Deamidation Q /	1 / 2 / 8	De-novo
743,8396	2	1485,6636	1485,7213	-0,0577	-38,8525	65,5172	351,4272	1	15	FANGAYSASTTALGR			De-novo
743,8396	2	1485,6636	1485,7213	-0,0577	-38,8525	63,2184	350,0593	1	15	AFNGAYSASTTALGR			De-novo
743,8396	2	1485,6636	1485,7213	-0,0577	-38,8525	74,7126	343,2218	1	15	FANGAYSASTTLAGR			De-novo
874,9301	2	1747,8446	1747,947	-0,1024	-58,5967	50,5376	118,3252	1	16	LNNLTGYTNLNLTGLK			De-novo
744,3436	2	1486,6716	1486,7052	-0,0336	-22,6165	67,8161	255,6888	1	15	AFNGAYSASTTALGR	Deamidation N	3	De-novo

NCBI gi|167879035|gb|EDS42418.1| conserved hypothetical protein [Culex Total Score: 301

```
                        0      40     80     120
                        |      |      |      |      |154
gi|167879035|gb|EDS42  | ______________________________________
Local hits (HSPs)      |    ___ _____ ___ __ ______ __ ___ _____ ___
```

```
Score = 37 (20.4 bits)
Identities = 8/12 (66%), Positives = 8/12 (66%)

Query:   507 SASTTALGRMKP 518
             SAST AL R  P
Sbjct:   132 SASTPALQRQRP 143   MatchSeq
```

```
Score = 33 (18.4 bits)
Identities = 7/9 (77%), Positives = 7/9 (77%)

Query:    43 SASTTALGR 51
             SAST AL R
Sbjct:    20 SASTPALQR 28   MatchSeq
```

```
Score = 33 (18.4 bits)
Identities = 7/9 (77%), Positives = 7/9 (77%)

Query:    58 SASTTALGR 66
             SAST AL R
Sbjct:    34 SASTPALQR 42   MatchSeq
```

```
Score = 33 (18.4 bits)
Identities = 7/9 (77%), Positives = 7/9 (77%)

Query:    73 SASTTALGR 81
             SAST AL R
Sbjct:    48 SASTPALQR 56   MatchSeq
```

```
Score = 33 (18.4 bits)
Identities = 7/9 (77%), Positives = 7/9 (77%)

Query:    88 SASTTALGR 96
             SAST AL R
Sbjct:    62 SASTPALQR 70   MatchSeq
```

```
Score = 33 (18.4 bits)
Identities = 7/9 (77%), Positives = 7/9 (77%)

Query:   103 SASTTALGR 111
             SAST AL R
Sbjct:    76 SASTPALQR 84   MatchSeq
```

```
Score = 33 (18.4 bits)
Identities = 7/9 (77%), Positives = 7/9 (77%)

Query:   118 SASTTALGR 126
             SAST AL R
Sbjct:    90 SASTPALQR 98   MatchSeq
```

```
Score = 33 (18.4 bits)
Identities = 7/9 (77%), Positives = 7/9 (77%)

Query:   133 SASTTALGR 141
             SAST AL R
Sbjct:   104 SASTPALQR 112   MatchSeq
```

```
Score = 33 (18.4 bits)
Identities = 7/9 (77%), Positives = 7/9 (77%)

Query:   446 SASTTALGR 454
             SAST AL R
Sbjct:   118 SASTPALQR 126   MatchSeq
```

ButterflyBase SFP04769_1 Total Score: 362

```
                           0      50     100    150    200
                           |      |      |      |      |     |244
butterfly|SFP04769_1  | ______________________________________
Local hits (HSPs)     |       ______ _    _ ___    _  __
```

```
Score = 72 (38.1 bits)
Identities = 12/15 (80%), Positives = 12/15 (80%)

Query:   500 KAFNGAYSASTTALG 514
             K FNG YSAS TALG
Sbjct:   128 KVFNGVYSASYTALG 142
```

```
Score = 65 (34.6 bits)
Identities = 9/18 (50%), Positives = 13/18 (72%)

Query:   306 LPELKMKPTDPLLLPELK 323
             +P+    K  DPL++PELK
Sbjct:    50 IPDYDIKAIDPLIIPELK 67
```

```
Score = 62 (33.1 bits)
Identities = 7/12 (58%), Positives = 11/12 (91%)

Query:   343 LKPLLLPELKFE 354
             + PL++PELK+E
Sbjct:    58 IDPLIIPELKYE 69
```

```
Score = 49 (26.5 bits)
Identities = 8/12 (66%), Positives = 9/12 (75%)

Query:    25 ALDSDPDAQALK 36
             A+D D DA ALK
Sbjct:   185 AIDQDADAKALK 196
```

```
Score = 46 (25.0 bits)
Identities = 6/8 (75%), Positives = 7/8 (87%)

Query:   493 NLNLTGLK 500
             N+N TGLK
Sbjct:    82 NINVTGLK 89
```

```
Score = 35 (19.4 bits)
Identities = 5/7 (71%), Positives = 6/7 (85%)

Query:   205 TKQVLNL 211
             TK VLN+
Sbjct:   109 TKAVLNI 115
```

```
Score = 33 (18.4 bits)
Identities = 5/9 (55%), Positives = 6/9 (66%)

Query:   363 EFFLLAPEV 371
             E+F   PEV
Sbjct:   157 EYFEVGPEV 165
```

Ha SG EST **None**

Ha EST other harmi_rn|HA-RN-ALL_Contig 4696 FWD frame 0 Total Score: 684

```
                        0      60     120    180    240
                        |      |      |      |      |  |251
harmi_rn|HA-RN-ALL_Co  | ______________________________________
Local hits (HSPs)      |   __   ___  __      ______ _____
```

```
Score = 109 (56.6 bits)
Identities = 16/16 (100%), Positives = 16/16 (100%)

Query:   500 KAFNGAYSASTTALGR 515
             KAFNGAYSASTTALGR
Sbjct:   134 KAFNGAYSASTTALGR 149
```

```
Score = 99 (51.6 bits)
Identities = 13/18 (72%), Positives = 16/18 (88%)

Query:   306 LPELKMKPTDPLLLPELK 323
             LP+  MKPTDPL++PELK
Sbjct:    55 LPDYNMKPTDPLIIPELK 72
```

```
Score = 88 (46.1 bits)
Identities = 13/13 (100%), Positives = 13/13 (100%)

Query:    24 KALDSDPDAQALK 36
             KALDSDPDAQALK
Sbjct:   190 KALDSDPDAQALK 202
```

```
Score = 76 (40.0 bits)
Identities = 10/11 (90%), Positives = 10/11 (90%)

Query:   536 KEFFLLAPEVH 546
             KEFF LAPEVH
Sbjct:   162 KEFFXLAPEVH 172
```

```
Score = 72 (38.0 bits)
Identities = 9/10 (90%), Positives = 10/10 (100%)

Query:   420 EEALFTACSK 429
             EEA+FTACSK
Sbjct:    25 EEAIFTACSK 34
```

```
Score = 72 (38.0 bits)
Identities = 10/11 (90%), Positives = 11/11 (100%)

Query:   246 EEPNVVLGDNL 256
             EEPNVVLGD+L
Sbjct:   178 EEPNVVLGDDL 188
```

```
Score = 70 (37.0 bits)
Identities = 10/11 (90%), Positives = 11/11 (100%)

Query:    15 NSDPDAQALKA 25
             +SDPDAQALKA
Sbjct:   193 DSDPDAQALKA 203
```

```
Score = 51 (27.5 bits)
Identities = 6/8 (75%), Positives = 8/8 (100%)

Query:   493 NLNLTGLK 500
             N+N+TGLK
Sbjct:    87 NINITGLK 94
```

```
Score = 47 (25.4 bits)
Identities = 6/8 (75%), Positives = 7/8 (87%)

Query:   178 EKGYSFTK 185
             + GYSFTK
Sbjct:   151 DXGYSFTK 158
```

Spot 21

m/z	Charge	Peak mW	Peptide mW	Delta (Da)	Delta (ppm)	Ladder Score	Log Likelihood	Start	End	Sequence	Modifications	Subs	Query Tool
541,2445	2	1080,4734	1080,4733	0,0001	0,0703	78,4314	121,8762	1	9	CPEPCPPPK	Carbamidomethyl C	1 / 5	De-novo
541,2445	2	1080,4734	1080,4731	0,0003	0,2554	78,4314	119,0407	1	9	CPQPCPPPK	Carbamidomethyl C /	1 / 3 / 5	De-novo
541,2445	2	1080,4734	1080,4733	0,0001	0,0703	75,4386	120,781	1	10	CPEPGCPPPK	Carbamidomethyl C	1	De-novo
796,3377	2	1590,6598	1590,7373	-0,0775	-48,7346	77,7778	428,6362	1	14	QVTETGVELSNEER	Deamidation Q	1	De-novo
796,3377	2	1590,6598	1590,7373	-0,0775	-48,7346	77,7778	428,2169	1	14	QVTETGVELSNEQR	Deamidation Q	1 / 13	De-novo
796,3377	2	1590,6598	1590,7373	-0,0775	-48,7346	77,7778	428,4013	1	14	QVTETGVELSNQER	Deamidation Q	1 / 12	De-novo
605,3025	2	1208,5894	1208,6038	-0,0144	-11,9344	76,1905	258,2289	1	11	YLAEVATGQTR	Deamidation Q	9	De-novo
571,2664	2	1140,5172	1140,5121	0,0051	4,4506	74,5098	235,1625	1	9	YNQELMNTK	Deamidation N	2	De-novo
571,2664	2	1140,5172	1140,5121	0,0051	4,4506	74,5098	231,6433	1	9	YNQQLMNTK	Deamidation N /	2 / 4	De-novo
571,2664	2	1140,5172	1140,5121	0,0051	4,4506	74,5098	231,1478	1	9	YDQELMNTK			De-novo
590,3046	2	1178,5936	1178,5931	0,0005	0,4039	77,193	166,1081	1	10	YEELQLTAGR			De-novo
590,3046	2	1178,5936	1178,5931	0,0005	0,4039	78,4314	161,7865	1	9	YEELQLTQR			De-novo
590,3046	2	1178,5936	1178,5931	0,0005	0,4039	77,193	165,4067	1	10	YQELQLTAGR	Deamidation Q	2	De-novo
586,275	2	1170,5344	1170,5557	-0,0213	-18,2170	36,5079	175,0374	1	11	NAGAFAYELSK	Deamidation N	1	De-novo
586,275	2	1170,5344	1170,5557	-0,0213	-18,2170	36,5079	175,0503	1	11	ANGAFAYELSK	Deamidation N	2	De-novo
586,275	2	1170,5344	1170,5557	-0,0213	-18,2170	36,5079	174,2953	1	11	NAGAFYAELSK	Deamidation N	1	De-novo
454,2638	2	906,5120	906,5175	-0,0055	-6,0936	62,2222	173,3454	1	8	NLLSVAYK			De-novo
1041,9852	2	2081,9548	2081,9753	-0,0205	-9,8579	48,6486	247,7657	1	19	TEANGTGSNTEYQQLLDLK	Deamidation N	4	De-novo
1041,9852	2	2081,9548	2081,9756	-0,0208	-10,0020	48,6486	247,3279	1	19	TEANTGGSNTEYQQLLDLK	Deamidation N	4	De-novo
1041,9852	2	2081,9548	2081,9753	-0,0205	-9,8579	48,6486	247,6819	1	19	TEANGTGSNTEYQQLLNLK	Deamidation N	4 / 17	De-novo
692,3532	2	1382,6908	1382,6831	0,0077	5,5515	74,6667	247,8607	1	13	SLNNFGAASFLNK	Deamidation N	12	De-novo
692,3532	2	1382,6908	1382,6831	0,0077	5,5515	74,6667	246,3835	1	13	SLNNGAFASFLNK	Deamidation N	12	De-novo
1079,4974	2	2156,9792	2156,9751	0,0041	1,8897	63,4146	319,2986	1	21	FNNAAGALAELDTLGGENSYK	Deamidation N	2 / 3 / 18	De-novo
1079,4974	2	2156,9792	2156,9751	0,0041	1,8897	63,4146	319,9465	1	21	FDNAAGALAELDTLGGENSYK	Deamidation N	3 / 18	De-novo
727,3683	2	1452,7210	1452,7865	-0,0655	-45,1023	58,6667	112,129	1	13	TLFGYDVLGLLDK			De-novo
727,3683	2	1452,7210	1452,7865	-0,0655	-45,1023	53,3333	111,4519	1	13	TLGFYDVLGLLDK			De-novo
651,8635	2	1301,7114	1301,7078	0,0036	2,7472	73,913	139,5696	1	12	SLDLDSLLAEVK			De-novo
651,8635	2	1301,7114	1301,7078	0,0036	2,7472	65,2174	139,7015	1	12	SLDLDLSLAEVK			De-novo
651,8635	2	1301,7114	1301,7078	0,0036	2,7472	73,913	138,0241	1	12	SLDLDSLLAQVK	Deamidation Q	10	De-novo
719,3707	2	1436,7258	1436,7223	0,0035	2,4194	69,5652	145,2244	1	12	LECYDVLGLLDK	Carbamidomethyl C	3	De-novo
719,3707	2	1436,7258	1436,7223	0,0035	2,4194	63,7681	142,2034	1	12	QLCYDVLGLLDK	Deamidation Q /	1 / 3	De-novo
719,3707	2	1436,7258	1436,7223	0,0035	2,4194	69,5652	145,4406	1	12	LECYNVLGLLDK	Carbamidomethyl C /	3 / 5	De-novo
595,3368	2	1188,6580	1188,6536	0,0044	3,6815	54,386	112,4042	1	10	TSNLLMQLLR	Deamidation N	3	De-novo
595,3368	2	1188,6580	1188,6536	0,0044	3,6815	68,4211	112,668	1	10	SDTLLMQLLR			De-novo
595,3368	2	1188,6580	1188,6536	0,0044	3,6815	68,4211	112,0987	1	10	SNTLLMQLLR	Deamidation N	2	De-novo
622,8313	2	1243,6470	1243,6407	0,0063	5,0465	71,4286	216,6569	1	11	QVGVSEELVQR	Deamidation Q	1	De-novo
622,8313	2	1243,6470	1243,6407	0,0063	5,0465	73,6842	217,0964	1	10	EVDKEELVQR			De-novo
622,8313	2	1243,6470	1243,6407	0,0063	5,0465	68,254	219,0448	1	11	LNGVSEELVQR	Deamidation N	2	De-novo
669,3961	1	668,3883	668,3898	-0,0015	-2,2622	70,3704	117,6788	1	5	VFYLK			De-novo
883,371	2	1764,7264	1764,7928	-0,0664	-37,6384	42,8571	103,3402	1	18	GSHSLGGNGSFGAQNYGR			De-novo
719,9995	3	2156,9751	2156,9785	-0,0034	-1,5930	57,265	218,7405	1	20	TLCSNAAELLDTLGGENSYK	Carbamidomethyl C /	3 / 5 / 17	De-novo
719,9995	3	2156,9751	2156,9785	-0,0034	-1,5930	52,1368	219,2505	1	20	TLCSNAAELLDTLGANDSYK	Carbamidomethyl C /	3 / 5 / 16	De-novo
719,9995	3	2156,9751	2156,9785	-0,0034	-1,5930	52,1368	220,0663	1	20	TLCSNAAELLDTLGANNSYK	Carbamidomethyl C /	' 5 / 16 /	De-novo
487,2664	2	972,5172	972,524	-0,0068	-7,0168	48,8889	77,822	1	8	LQLSELNR	Deamidation Q	2	De-novo
633,3289	2	1264,6422	1264,63	0,0122	9,6281	77,7778	138,0534	1	11	NTAENEFVTLK			De-novo
633,3289	2	1264,6422	1264,63	0,0122	9,6281	77,7778	136,8717	1	11	NTAENQFVTLK	Deamidation Q	6	De-novo
854,3894	2	1706,7632	1706,7609	0,0023	1,3335	50,5051	114,7397	1	17	GSGLNFSSGNGPQQDSR			De-novo
854,3894	2	1706,7632	1706,7609	0,0023	1,3335	56,5657	113,8997	1	17	GSGLNFSSGNGPQQSDR			De-novo
854,3894	2	1706,7632	1706,7609	0,0023	1,3335	49,4949	113,0273	1	17	GSGLNFSSGNGPQENSR			De-novo

NCBI gi|237636932|gb|ACR07788.1| 14-3-3 zeta [Heliothis virescens] Total Score: 796

```
                         0         50        100       150       200
                         |         |         |         |         |         |247
gi|237636932|gb|ACR07  | ________________________________________________
Local hits (HSPs)      | _  _______       ____ _ __    _         _ __
```

```
 Score = 106 (54.3 bits)
 Identities = 15/17 (88%), Positives = 16/17 (94%)

Query:   673 KQVTETGVELSNEERSL 689
             K+VTETGVELSNEER L
Sbjct:    29 KEVTETGVELSNEERNL 45   MatchSeq

 Score = 91 (46.9 bits)
 Identities = 15/20 (75%), Positives = 17/20 (85%)

Query:    57 QVTETGVELSNQERYLAEVA 76
             +VTETGVELSN+ER L  VA
Sbjct:    30 EVTETGVELSNEERNLLSVA 49   MatchSeq

 Score = 79 (41.0 bits)
 Identities = 11/12 (91%), Positives = 12/12 (100%)

Query:    70 RYLAEVATGQTR 81
             RYLAEVATG+TR
Sbjct:   129 RYLAEVATGETR 140   MatchSeq

 Score = 77 (40.0 bits)
 Identities = 11/12 (91%), Positives = 12/12 (100%)

Query:   368 CYDVLGLLDKQL 379
             CYDVLGLLDK+L
Sbjct:    96 CYDVLGLLDKHL 107   MatchSeq

 Score = 77 (40.0 bits)
 Identities = 10/12 (83%), Positives = 12/12 (100%)

Query:   378 QLCYDVLGLLDK 389
             ++CYDVLGLLDK
Sbjct:    94 EICYDVLGLLDK 105   MatchSeq

 Score = 66 (34.5 bits)
 Identities = 9/9 (100%), Positives = 9/9 (100%)

Query:   444 VDKEELVQR 452
             VDKEELVQR
Sbjct:     3 VDKEELVQR 11   MatchSeq

 Score = 58 (30.6 bits)
 Identities = 8/10 (80%), Positives = 9/10 (90%)

Query:   414 TLLMQLLRSN 423
             TL+MQLLR N
Sbjct:   217 TLIMQLLRDN 226   MatchSeq

 Score = 54 (28.6 bits)
 Identities = 8/8 (100%), Positives = 8/8 (100%)

Query:   171 NLLSVAYK 178
             NLLSVAYK
Sbjct:    44 NLLSVAYK 51   MatchSeq

 Score = 53 (28.1 bits)
 Identities = 8/11 (72%), Positives = 9/11 (81%)

Query:   401 KTSNLLMQLLR 411
             K S L+MQLLR
Sbjct:   214 KDSTLIMQLLR 224   MatchSeq

 Score = 50 (26.6 bits)
 Identities = 7/8 (87%), Positives = 8/8 (100%)

Query:   268 ALAELDTL 275
             A+AELDTL
Sbjct:   201 AIAELDTL 208   MatchSeq

 Score = 45 (24.1 bits)
 Identities = 6/6 (100%), Positives = 6/6 (100%)

Query:   711 KVFYLK 716
             KVFYLK
Sbjct:   117 KVFYLK 122   MatchSeq

 Score = 40 (21.6 bits)
 Identities = 5/7 (71%), Positives = 7/7 (100%)

Query:   143 AYELSKA 149
             A+E+SKA
Sbjct:   154 AFEISKA 160   MatchSeq
```

Ha SG EST hargln|HA-GLN Contig 1084 FWD frame 1 Total Score: 791

```
                         0         80        160       240       320
                         |         |         |         |         |         |394
hargln|HA-GLN_Contig_  | ________________________________________________
Local hits (HSPs)      |     _  _____    _ _____ _      __
```

```
 Score = 106 (54.3 bits)
 Identities = 15/17 (88%), Positives = 16/17 (94%)

Query:   673 KQVTETGVELSNEERSL 689
             K+VTETGVELSNEER L
Sbjct:    69 KEVTETGVELSNEERNL 85

 Score = 91 (46.9 bits)
 Identities = 15/20 (75%), Positives = 17/20 (85%)

Query:    57 QVTETGVELSNQERYLAEVA 76
             +VTETGVELSN+ER L  VA
Sbjct:    70 EVTETGVELSNEERNLLSVA 89

 Score = 79 (41.0 bits)
 Identities = 11/12 (91%), Positives = 12/12 (100%)

Query:    70 RYLAEVATGQTR 81
             RYLAEVATG+TR
Sbjct:   169 RYLAEVATGETR 180

 Score = 77 (40.0 bits)
 Identities = 11/12 (91%), Positives = 12/12 (100%)

Query:   368 CYDVLGLLDKQL 379
             CYDVLGLLDK+L
Sbjct:   136 CYDVLGLLDKHL 147

 Score = 77 (40.0 bits)
 Identities = 10/12 (83%), Positives = 12/12 (100%)

Query:   378 QLCYDVLGLLDK 389
             ++CYDVLGLLDK
Sbjct:   134 EICYDVLGLLDK 145

 Score = 61 (32.0 bits)
 Identities = 8/9 (88%), Positives = 8/9 (88%)

Query:   444 VDKEELVQR 452
             VDKEE VQR
Sbjct:    43 VDKEEXVQR 51

 Score = 58 (30.6 bits)
 Identities = 8/10 (80%), Positives = 9/10 (90%)

Query:   414 TLLMQLLRSN 423
             TL+MQLLR N
Sbjct:   257 TLIMQLLRDN 266

 Score = 54 (28.6 bits)
 Identities = 8/8 (100%), Positives = 8/8 (100%)

Query:   171 NLLSVAYK 178
             NLLSVAYK
Sbjct:    84 NLLSVAYK 91

 Score = 53 (28.1 bits)
 Identities = 8/11 (72%), Positives = 9/11 (81%)

Query:   401 KTSNLLMQLLR 411
             K S L+MQLLR
Sbjct:   254 KDSTLIMQLLR 264

 Score = 50 (26.6 bits)
 Identities = 7/8 (87%), Positives = 8/8 (100%)

Query:   268 ALAELDTL 275
             A+AELDTL
Sbjct:   241 AIAELDTL 248

 Score = 45 (24.1 bits)
 Identities = 6/6 (100%), Positives = 6/6 (100%)

Query:   711 KVFYLK 716
             KVFYLK
Sbjct:   157 KVFYLK 162

 Score = 40 (21.6 bits)
 Identities = 5/7 (71%), Positives = 7/7 (100%)

Query:   143 AYELSKA 149
             A+E+SKA
Sbjct:   194 AFEISKA 200
```

Spot 22

m/z	Charge	Peak mW	Peptide mW	Delta (Da)	Delta (ppm)	Ladder Score	Log Likelihood	Start	End	Sequence	Modifications	Subs	Query Tool
691,3278	2	1380,6400	1380,6046	0,0354	25,6236	56,5217	74,8147	1	12	PSEESNEYLEGK			De-novo
691,3278	2	1380,6400	1380,6046	0,0354	25,6236	56,5217	74,4961	1	12	PSEESNEYLQGK	Deamidation Q	10	De-novo
691,3278	2	1380,6400	1380,6046	0,0354	25,6236	56,5217	74,6714	1	12	PSQESNEYLEGK	Deamidation Q	3	De-novo
825,9229	2	1649,8302	1649,7898	0,0404	24,4734	52,5253	153,4381	1	17	TNAAVSGGDPATYLANK	Deamidation N	16	De-novo
825,9229	2	1649,8302	1649,7898	0,0404	24,4734	52,5253	152,882	1	17	TNAAVSGGDPATYLADK			De-novo
825,9229	2	1649,8302	1649,7898	0,0404	24,4734	52,5253	153,0807	1	17	TANAVSGGDPATYLANK	Deamidation N	16	De-novo
718,8592	2	1435,7028	1435,7017	0,0011	0,7495	65,2174	138,8626	1	12	TLEMDAVLWQSK	Oxidation M	4	De-novo
423,2257	2	844,4358	844,429	0,0068	8,0244	49,0196	41,1933	1	9	SPATGVNLS			De-novo
423,2257	2	844,4358	844,4402	-0,0044	-5,2390	48,8889	41,348	1	8	SPATRNLS			De-novo
530,3121	2	1058,6086	1058,6124	-0,0038	-3,6123	59,6491	200,5563	1	10	VLPVGYGLNK			De-novo
530,3121	2	1058,6086	1058,6124	-0,0038	-3,6123	59,6491	199,2359	1	10	LVPVGYGLNK			De-novo
530,3121	2	1058,6086	1058,5397	0,0689	65,0670	63,1579	195,8825	1	10	NPPVGYGLNK	Deamidation N	1	De-novo

NCBI gi|91088233|ref|XP_973769.1| PREDICTED: similar to Elongation factor 1 beta CG6341-PA [Tribolium castaneum] Total Score: 221

```
                         0         50        100       150       200
                         |         |         |         |         |    |218
gi|91088233|ref|XP_97  | ________________________________________________
Local hits (HSPs)      |    __                         _____
```

```
 Score = 76 (39.7 bits)
 Identities = 10/11 (90%), Positives = 11/11 (100%)

Query:   126 KLVPVGYGLNK 136
             KLVPVGYG+NK
Sbjct:   169 KLVPVGYGINK 179   MatchSeq

 Score = 62 (32.7 bits)
 Identities = 8/9 (88%), Positives = 9/9 (100%)

Query:   119 PVGYGLNKL 127
             PVGYG+NKL
Sbjct:   172 PVGYGINKL 180   MatchSeq

 Score = 50 (26.7 bits)
 Identities = 5/9 (55%), Positives = 6/9 (66%)

Query:    88 TLEMDAVLW 96
             T+EMD   W
Sbjct:   157 TIEMDGLVW 165   MatchSeq

 Score = 33 (18.3 bits)
 Identities = 5/8 (62%), Positives = 6/8 (75%)

Query:     4 ESNEYLEG 11
             ES  Y+EG
Sbjct:    23 ESRSYIEG 30   MatchSeq
```

ButterflyBase AYP00014_1 Total Score: 193

```
                         0         40        80        120       160
                         |         |         |         |         | |164
butterfly|AYP00014_1   | ________________________________________________
Local hits (HSPs)      |                              ___ ____
```

```
 Score = 76 (39.6 bits)
 Identities = 10/11 (90%), Positives = 11/11 (100%)

Query:   126 KLVPVGYGLNK 136
             KLVPVGYG+NK
Sbjct:   113 KLVPVGYGINK 123

 Score = 62 (32.6 bits)
 Identities = 8/9 (88%), Positives = 9/9 (100%)

Query:   119 PVGYGLNKL 127
             PVGYG+NKL
Sbjct:   116 PVGYGINKL 124

 Score = 55 (29.1 bits)
 Identities = 6/9 (66%), Positives = 7/9 (77%)

Query:    88 TLEMDAVLW 96
             T+EMD  LW
Sbjct:   101 TIEMDGLLW 109
```

Ha SG EST hargln|HA-GLN Contig 21 FWD frame 1 Total Score: 222

```
                         0         60        120       180       240
                         |         |         |         |         |     |269
hargln|HA-GLN_Contig_  | ________________________________________________
Local hits (HSPs)      |     _                       _____
```

```
 Score = 76 (39.7 bits)
 Identities = 10/11 (90%), Positives = 11/11 (100%)

Query:   126 KLVPVGYGLNK 136
             KLVPVGYG+NK
Sbjct:   192 KLVPVGYGINK 202

 Score = 62 (32.7 bits)
 Identities = 8/9 (88%), Positives = 9/9 (100%)

Query:   119 PVGYGLNKL 127
             PVGYG+NKL
Sbjct:   195 PVGYGINKL 203

 Score = 47 (25.2 bits)
 Identities = 5/9 (55%), Positives = 6/9 (66%)

Query:    88 TLEMDAVLW 96
             T+EM   LW
Sbjct:   180 TIEMXGLLW 188

 Score = 37 (20.2 bits)
 Identities = 5/6 (83%), Positives = 5/6 (83%)

Query:    65 TYLADK 70
             TYL DK
Sbjct:    35 TYLXDK 40
```

Spot 23

m/z	Charge	Peak mW	Peptide mW	Delta (Da)	Delta (ppm)	Ladder Score	Log Likelihood	Start	End	Sequence	Modifications	Subs	Query Tool
601,2976	2	1200,5796	1200,5846	-0,0050	-4,1846	57,1429	77,1338	1	11	NTNAADVQNVR			De-novo
601,2976	2	1200,5796	1200,5847	-0,0051	-4,2679	57,1429	76,956	1	11	NTNAADVQVNR			De-novo
601,2976	2	1200,5796	1200,5846	-0,0050	-4,1846	57,1429	76,6495	1	11	NTNAADVNVQR			De-novo
666,8398	2	1331,6640	1331,6682	-0,0042	-3,1720	70,1149	200,0867	1	15	ALGSGSQGAQAGSLK	Deamidation Q	7	De-novo
666,8398	2	1331,6640	1331,6682	-0,0042	-3,1720	67,8161	200,6363	1	15	ALGSGSQGAQAGSLK	Deamidation Q	7	De-novo
666,8398	2	1331,6640	1331,6682	-0,0042	-3,1720	70,1149	198,9853	1	15	LAGSGSQGAQAGSLK	Deamidation Q	7	De-novo
626,8118	2	1251,6080	1251,6095	-0,0015	-1,2176	61,9048	204,2787	1	11	GNSLYELNKGR	Deamidation N	2 / 8	De-novo
626,8118	2	1251,6080	1251,6095	-0,0015	-1,2176	61,9048	203,6983	1	11	NGSLYELNKGR	Deamidation N	1 / 8	De-novo
626,8118	2	1251,6080	1251,6095	-0,0015	-1,2176	61,9048	200,6751	1	11	GNSLYELDKGR	Deamidation N	2	De-novo
725,8619	2	1449,7082	1449,7098	-0,0016	-1,1202	73,3333	246,7076	1	13	FLNEAKDLDASAR	Deamidation N	3	De-novo
725,8619	2	1449,7082	1449,7098	-0,0016	-1,1202	73,3333	247,1806	1	13	FLNEAKDLNASAR	Deamidation N	3 / 9	De-novo
598,3449	2	1194,6742	1194,672	0,0022	1,8214	49,2063	78,0997	1	11	NAVPLTTNRLP			De-novo
598,3449	2	1194,6742	1194,672	0,0022	1,8214	57,1429	77,5159	1	11	NAVPLTTRNLP			De-novo
598,3449	2	1194,6742	1194,672	0,0022	1,8214	42,8571	77,1842	1	11	NAVPLTLPSRQ			De-novo
738,3874	2	1474,7592	1474,7589	0,0003	0,1871	66,6667	269,2781	1	13	LTSPLMEPTTLEK	Oxidation M	6	De-novo
738,3874	2	1474,7592	1474,7589	0,0003	0,1871	64	266,1393	1	13	LTSPLMEPTLTEK	Oxidation M	6	De-novo
738,3874	2	1474,7592	1474,7589	0,0003	0,1871	66,6667	266,0718	1	13	TLSPLMEPTTLEK	Oxidation M	6	De-novo
562,7639	2	1123,5122	1123,5146	-0,0024	-2,1575	64,9123	119,3554	1	10	GNSLYEVEGR	Deamidation N	2	De-novo
562,7639	2	1123,5122	1123,5146	-0,0024	-2,1575	57,8947	118,7483	1	10	NGSLYEVEGR	Deamidation N	1	De-novo
562,7639	2	1123,5122	1123,5145	-0,0023	-2,0685	56,1404	121,4131	1	10	GSDLYELNGR	Deamidation N	8	De-novo
730,3897	2	1458,7638	1458,764	-0,0002	-0,1536	77,3333	237,2142	1	13	LTSPLMEPTTLEK			De-novo
730,3897	2	1458,7638	1458,764	-0,0002	-0,1536	74,6667	236,8215	1	13	TLSPLMEPTTLEK			De-novo
730,3897	2	1458,7638	1458,764	-0,0002	-0,1536	77,3333	234,7816	1	13	LTSPLMEPTTLQK	Deamidation Q	12	De-novo
701,8395	2	1401,6634	1401,6372	0,0262	18,6753	39,0805	142,7951	1	15	NAEVSSAAGGPEANK	Deamidation N	14	De-novo
701,8395	2	1401,6634	1401,6372	0,0262	18,6753	36,7816	141,705	1	15	ANEVSSAAGGPEANK	Deamidation N	14	De-novo
479,2151	2	956,4146	956,4927	-0,0781	-81,6776	50,9804	100,8789	1	9	ESATLTAHK			De-novo
479,2151	2	956,4146	956,4563	-0,0417	-43,6235	52,9412	103,4544	1	9	ESGDLTAHK			De-novo
733,8032	2	1465,5908	1465,7571	-0,1663	-113,4731	39,5062	67,66	1	14	GHGLGLSSPAVCRR	Carbamidomethyl C	12	De-novo
733,8032	2	1465,5908	1465,7096	-0,1188	-81,0693	46,6667	64,9221	1	13	NYSGLSSPAVRCR	Carbamidomethyl C	12	De-novo
733,8032	2	1465,5908	1465,7096	-0,1188	-81,0693	46,6667	64,9694	1	13	YNSGLSSPAVRCR	Carbamidomethyl C	12	De-novo
484,2417	3	1449,7017	1449,7098	-0,0081	-5,6122	54,6667	142,9943	1	13	ENLFAKDLNASAR	Deamidation N	2 / 9	De-novo
484,2417	3	1449,7017	1449,7098	-0,0081	-5,6122	54,6667	141,0067	1	13	EDLFAKDLNASAR	Deamidation N	9	De-novo
709,8358	2	1417,6560	1417,6508	0,0052	3,6511	65,5172	279,8339	1	15	NAEVSSAVGPGSLCQ			De-novo
709,8358	2	1417,6560	1417,6508	0,0052	3,6511	58,6207	278,5372	1	15	ANEVSSAVGPGSLCQ			De-novo
709,8358	2	1417,6560	1417,6509	0,0051	3,5806	70,1149	276,0895	1	15	NAEVSSAVGPGGLMN	Oxidation M	14	De-novo

NCBI gi|91087635|ref|XP_973117.1| PREDICTED: similar to proteasome zeta subunit [Tribolium castaneum] Total Score: 198

```
                     0       50      100     150     200
                     |       |       |       |       |      |242
gi|91087635|ref|XP_97  | ________________________________________
Local hits (HSPs)      |        __
```

Score = 105 (54.2 bits)
Identities = 12/15 (80%), Positives = 15/15 (100%)

```
Query:   448 RLTSPLMEPTTLEKL 462
             R+TSPLMEPTT+EK+
Sbjct:    53 RITSPLMEPTTIEKI 67   MatchSeq
```

Score = 93 (48.2 bits)
Identities = 12/17 (70%), Positives = 15/17 (88%)

```
Query:   266 LTSPLMEPTTLQKNAEV 282
             +TSPLMEPTT++K  EV
Sbjct:    54 ITSPLMEPTTIEKIVEV 70   MatchSeq
```

ButterflyBase HMP00286_1 Total Score: 308

```
                     0       50      100     150     200
                     |       |       |       |       |      |243
butterfly|HMP00286_1   | ________________________________________
Local hits (HSPs)      |       __                 __
```

Score = 100 (51.9 bits)
Identities = 12/14 (85%), Positives = 14/14 (100%)

```
Query:   239 RLTSPLMEPTTLEK 252
             R+TSPLMEPTT+EK
Sbjct:    53 RITSPLMEPTTIEK 66
```

Score = 97 (50.4 bits)
Identities = 11/14 (78%), Positives = 14/14 (100%)

```
Query:   171 LTSPLMEPTTLEKL 184
             +TSPLMEPTT+EK+
Sbjct:    54 ITSPLMEPTTIEKI 67
```

Score = 56 (29.8 bits)
Identities = 8/10 (80%), Positives = 10/10 (100%)

```
Query:    34 ALGSGSQGAQ 43
             A+GSGS+GAQ
Sbjct:   170 AIGSGSEGAQ 179
```

Score = 55 (29.3 bits)
Identities = 8/10 (80%), Positives = 10/10 (100%)

```
Query:    48 KALGSGSQGA 57
             KA+GSGS+GA
Sbjct:   169 KAIGSGSEGA 178
```

Ha SG EST hargln|HA-GLN_Contig_1190_FWD_frame_0 Total Score: 198

```
                     0       40      80      120     160
                     |       |       |       |       |      |199
hargln|HA-GLN_Contig_  | ________________________________________
Local hits (HSPs)      |                __
```

Score = 105 (54.2 bits)
Identities = 12/15 (80%), Positives = 15/15 (100%)

```
Query:   448 RLTSPLMEPTTLEKL 462
             R+TSPLMEPTT+EK+
Sbjct:    92 RITSPLMEPTTIEKI 106
```

Score = 93 (48.2 bits)
Identities = 12/17 (70%), Positives = 15/17 (88%)

```
Query:   266 LTSPLMEPTTLQKNAEV 282
             +TSPLMEPTT++K  EV
Sbjct:    93 ITSPLMEPTTIEKIVEV 109
```

Spot 24

m/z	Charge	Peak mW	Peptide mW	Delta (Da)	Delta (ppm)	Ladder Score	Log Likelihood	Start	End	Sequence	Modifications	Subs	Query Tool
649,3097	2	1296,6038	1296,6021	0,0017	1,2926	63,7681	136,1882	1	12	FNELSGVSCVNK	Deamidation N	2	De-novo
649,3097	2	1296,6038	1296,6021	0,0017	1,2926	63,4921	130,9635	1	11	FNELSSVCVNK	Deamidation N /	2 / 8	De-novo
649,3097	2	1296,6038	1296,6022	0,0016	1,2155	63,7681	134,3931	1	12	FDELSGVSCVNK			De-novo
598,3229	2	1194,6302	1194,5629	0,0673	56,3185	61,9048	85,7918	1	11	PNPAPQASQQR	Deamidation N /	2 / 6	De-novo
598,3229	2	1194,6302	1194,5629	0,0673	56,3185	65,0794	84,279	1	11	PNPAPAESQQR	Deamidation N	2	De-novo
598,3229	2	1194,6302	1194,5629	0,0673	56,3185	61,9048	84,6007	1	11	PNPAPEASQQR	Deamidation N	2	De-novo
687,8836	2	1373,7516	1373,7667	-0,0151	-11,0091	62,6667	161,7385	1	13	ELLLSAGVPHNPK			De-novo
687,8836	2	1373,7516	1373,7666	-0,0150	-10,9364	54,6667	160,8929	1	13	ELLLSAGHPVNPK			De-novo
687,8836	2	1373,7516	1373,7666	-0,0150	-10,9364	54,6667	160,4805	1	13	ELLLSAGVHNPPK			De-novo

NCBI gi|242018482|ref|XP_002429704.1| glucose dehydrogenase, putative [Pediculus Total Score: 121

```
                     0       140     280     420     560
                     |       |       |       |       |      |662
gi|242018482|ref|XP_0  | ________________________________________
Local hits (HSPs)      |                  __
```

Score = 61 (32.4 bits)
Identities = 12/21 (57%), Positives = 14/21 (66%)

```
Query:    94 KELLLSAGVHNPPKFNELSGV 114
             KE +LSAGV N P    LSG+
Sbjct:   342 KEVILSAGVVNSPRLLLLSGI 362   MatchSeq
```

Score = 60 (31.9 bits)
Identities = 11/20 (55%), Positives = 13/20 (65%)

```
Query:    67 QRELLLSAGVPHNPKELLLS 86
              + E +LSAGV   P  LLLS
Sbjct:   341 EKEVILSAGVVNSPRLLLLS 360   MatchSeq
```

ButterflyBase None

Ha SG EST hargln|HA-GLN_Contig_236_FWD_frame_1 Total Score: 82

```
                     0       80      160     240     320
                     |       |       |       |       |      |394
hargln|HA-GLN_Contig_  | ________________________________________
Local hits (HSPs)      | __
```

Score = 82 (43.0 bits)
Identities = 13/21 (61%), Positives = 17/21 (80%)

```
Query:    94 KELLLSAGVHNPPKFNELSGV 114
             KE++LSAGV N PK  +LSG+
Sbjct:    24 KEIILSAGVFNSPKILQLSGI 44
```

Spot 25

m/z	Charge	Peak mW	Peptide mW	Delta (Da)	Delta (ppm)	Ladder Score	Log Likelihood	Start	End	Sequence	Modifications	Subs	Query Tool
688,8311	2	1375,6466	1375,6466	0,0000	-0,0174	65,2174	151,6896	1	12	ELNETKDQDGVK	Deamidation N	3	De-novo
688,8311	2	1375,6466	1375,6466	0,0000	-0,0174	60,8696	151,3849	1	12	ELNETKDENGVK	Deamidation N	3	De-novo
688,8311	2	1375,6466	1375,6466	0,0000	-0,0174	57,3333	153,824	1	13	ELNETKDGANGVK	Deamidation N	3 / 10	De-novo
562,2449	2	1122,4742	1122,4799	-0,0057	-5,0994	60,3175	167,1523	1	11	LNTVCNCGGGK	Carbamidomethyl C /	5 / 6	De-novo
562,2449	2	1122,4742	1122,4799	-0,0057	-5,0994	68,4211	163,5957	1	10	LNTVCNCGGK	Carbamidomethyl C /	5 / 6 / 7	De-novo
562,2449	2	1122,4742	1122,4799	-0,0057	-5,0994	60,3175	164,4682	1	11	LNTVCDCGGGK	Carbamidomethyl C	5	De-novo
576,803	2	1151,5904	1151,5896	0,0008	0,6739	68,4211	186,3305	1	10	FSTPMVVLDK	Oxidation M	5	De-novo
576,803	2	1151,5904	1151,5896	0,0008	0,6739	68,4211	184,5839	1	10	FSTPMVVLNK	Oxidation M / Deamidation	5 / 9	De-novo
576,803	2	1151,5904	1151,5896	0,0008	0,6739	61,4035	181,6102	1	10	SFTPMVVLDK	Oxidation M	5	De-novo
568,806	2	1135,5964	1135,5947	0,0017	1,4759	68,4211	204,0296	1	10	FSTPMVVLDK			De-novo
568,806	2	1135,5964	1135,5947	0,0017	1,4759	64,9123	199,8821	1	10	SFTPMVVLDK			De-novo
568,806	2	1135,5964	1135,5947	0,0017	1,4759	68,4211	201,3422	1	10	FSTPMVVLNK	Deamidation N	9	De-novo
696,8758	2	1391,7360	1391,6456	0,0904	64,9418	47,8261	70,1292	1	12	PDDEVLNLNTYV	Deamidation N	9	De-novo
696,8758	2	1391,7360	1391,6456	0,0904	64,9418	49,2754	68,5596	1	12	PDDEVLNLNVYT	Deamidation N	9	De-novo
696,8758	2	1391,7360	1391,6456	0,0904	64,9418	43,4783	68,4013	1	12	DPDEVLNLNYVT	Deamidation N	9	De-novo

NCBI gi|193636585|ref|XP_001943976.1| PREDICTED: similar to viral A-type inclusion protein, putative, partial [Acyrthosiphon pisum] Total Score: 179

```
                     0       360     720     1080    1440
                     |       |       |       |       |      |1752
gi|193636585|ref|XP_0  | ________________________________________
Local hits (HSPs)      |          _  _ _           _
```

Score = 60 (31.5 bits)
Identities = 9/15 (60%), Positives = 12/15 (80%)

```
Query:    16 ETKDENGVKELNETK 30
             +T  E GVK+LN+TK
Sbjct:   382 QTRAEGGVKQLNDTK 396   MatchSeq
```

Score = 48 (25.5 bits)
Identities = 6/7 (85%), Positives = 6/7 (85%)

```
Query:    83 PMVVLNK 89
             PMVV NK
Sbjct:   568 PMVVVNK 574   MatchSeq
```

Score = 36 (19.6 bits)
Identities = 5/8 (62%), Positives = 7/8 (87%)

```
Query:   144 GKDKAAME 151
             GK+K AM+
Sbjct:  1135 GKNKIAMD 1142   MatchSeq
```

Score = 35 (19.1 bits)
Identities = 4/9 (44%), Positives = 7/9 (77%)

```
Query:   183 PDDEVLNLN 191
             P DEV+ ++
Sbjct:   625 PADEVIIID 633   MatchSeq
```

ButterflyBase none

Ha SG EST harmi_gn|HA-GN-ALL_Contig_927_FWD_frame_2 Total Score: 335

```
                     0       60      120     180     240
                     |       |       |       |       |  |250
harmi_gn|HA-GN-ALL_Co  | ________________________________________
Local hits (HSPs)      | __                 _____      __
```

Score = 78 (40.4 bits)
Identities = 10/13 (76%), Positives = 11/13 (84%)

```
Query:    69 KFSTPMVVLDKFS 81
             KFSTPM V+DK S
Sbjct:    14 KFSTPMLVIDKLS 26
```

Score = 78 (40.4 bits)
Identities = 10/13 (76%), Positives = 11/13 (84%)

```
Query:   228 KFSTPMVVLDKFS 240
             KFSTPM V+DK S
Sbjct:   132 KFSTPMLVIDKLS 144
```

Score = 61 (32.0 bits)
Identities = 9/13 (69%), Positives = 10/13 (76%)

```
Query:    34 NGVKLNTVCNCGG 46
             NG  L TVC+CGG
Sbjct:   146 NGPXLXTVCDCGG 158
```

Score = 60 (31.5 bits)
Identities = 8/10 (80%), Positives = 9/10 (90%)

```
Query:    49 LNTVCNCGGK 58
             L TVC+CGGK
Sbjct:   150 LXTVCDCGGK 159
```

Score = 58 (30.5 bits)
Identities = 8/11 (72%), Positives = 10/11 (90%)

```
Query:     2 LNETKDQDGVK 12
             ++ETKD DGVK
Sbjct:   208 IDETKDLDGVK 218
```

Spot 26

m/z	Charge	Peak mW	Peptide mW	Delta (Da)	Delta (ppm)	Ladder Score	Log Likelihood	Start	End	Sequence	Modifications	Subs	Query Tool
631,8011	2	1261,5866	1261,5826	0,0040	3,1516	57,1429	56,0506	1	11	TVLEFEANDQP			De-novo
509,7629	2	1017,5102	1017,5131	-0,0029	-2,8737	82,4561	259,6748	1	10	APNGAVDVFK	Deamidation N	3	De-novo
627,8392	2	1253,6628	1253,6656	-0,0028	-2,2526	49,2063	99,2688	1	11	LVQTFAQAFTK	Deamidation Q	3	De-novo
627,8392	2	1253,6628	1253,6655	-0,0027	-2,1728	65,0794	97,5579	1	11	LVQTFAFGDKK	Deamidation Q	3	De-novo
627,8392	2	1253,6628	1253,6656	-0,0028	-2,2526	56,5217	99,7102	1	12	LVQTFAFGVSGK	Deamidation Q	3	De-novo
825,8638	2	1649,7120	1649,6768	0,0352	21,3230	55,5556	136,7363	1	14	DLNYGDETLCNTYK	Deamidation N	3 / 11	De-novo
825,8638	2	1649,7120	1649,6768	0,0352	21,3230	53,0864	136,2134	1	14	LDNYGDETLCNTYK	Deamidation N	3 / 11	De-novo
825,8638	2	1649,7120	1649,6768	0,0352	21,3230	53,0864	136,8645	1	14	EVNYGDETLCNTYK	Deamidation N	3 / 11	De-novo
817,8663	2	1633,7170	1633,7181	-0,0011	-0,6880	67,9012	173,7109	1	14	DLLTGNEMFSDTYK	Deamidation N	6	De-novo
817,8663	2	1633,7170	1633,7181	-0,0011	-0,6880	67,9012	170,6809	1	14	DLLTGDEMFSDTYK			De-novo
817,8663	2	1633,7170	1633,7181	-0,0011	-0,6880	60,4938	164,8736	1	14	DLLTNGEMFSDTYK	Deamidation N	5	De-novo
696,8754	2	1391,7352	1391,7296	0,0056	4,0065	75,3623	258,2861	1	12	LVNEVLYEVTGR	Deamidation N	3	De-novo
696,8754	2	1391,7352	1391,7296	0,0056	4,0065	75,3623	257,5246	1	12	LVNEVLYQVTGR	Deamidation N /	3 / 8	De-novo
696,8754	2	1391,7352	1391,7296	0,0056	4,0065	72,4638	255,1007	1	12	VLNEVLYEVTGR	Deamidation N	3	De-novo
702,8767	2	1403,7378	1403,7449	-0,0071	-5,0750	36,5079	25,9536	1	11	LPEEVLVWYTR			De-novo
702,8767	2	1403,7378	1403,7449	-0,0071	-5,0750	36,5079	25,9781	1	11	LPQEVLVWYTR	Deamidation Q	3	De-novo
702,8767	2	1403,7378	1403,7296	0,0082	5,8245	36,5079	28,2683	1	11	LPEEVLYKEER			De-novo

NCBI gi|66515987|ref|XP_395299.2| PREDICTED: similar to Translationally controlled tumor protein CG4800-PA isoform 1 [Apis mellifera] Total Score: 339

```
                      0       40       80       120      160
                      |       |        |        |        |   |172
gi|66515987|ref|XP_39 | ________________________________________
Local hits (HSPs)     | _______         ___
```

Score = 114 (57.4 bits)
Identities = 14/16 (87%), Positives = 15/16 (93%)

```
Query:   110 YKDLLTGDEMFSDTYK 125
             YKD+ TGDEMFSDTYK
Sbjct:     4 YKDIFTGDEMFSDTYK 19   MatchSeq
```

Score = 103 (52.1 bits)
Identities = 12/16 (75%), Positives = 15/16 (93%)

```
Query:   230 KDLLTGNEMFSDTYKL 245
             KD+ TG+EMFSDTYK+
Sbjct:     5 KDIFTGDEMFSDTYKI 20   MatchSeq
```

Score = 62 (32.1 bits)
Identities = 9/15 (60%), Positives = 12/15 (80%)

```
Query:   139 KLVNEVLYEVTGRLV 153
             KLV++VLYEV G  +
Sbjct:    21 KLVDDVLYEVYGKVI 35   MatchSeq
```

Score = 60 (31.1 bits)
Identities = 8/9 (88%), Positives = 9/9 (100%)

```
Query:    35 QTFAFGDKK 43
             +TFAFGDKK
Sbjct:    76 ETFAFGDKK 84   MatchSeq
```

ButterflyBase SFP00311_3 Total Score: 389

```
                      0       40       80       120      160
                      |       |        |        |        |   |175
db|SFP00311_3|SFP0031 | ________________________________________
Local hits (HSPs)     |  _______        __
```

Score = 122 (61.3 bits)
Identities = 14/16 (87%), Positives = 16/16 (100%)

```
Query:   110 YKDLLTGDEMFSDTYK 125
             YKD++TGDEMFSDTYK
Sbjct:     7 YKDIITGDEMFSDTYK 22
```

Score = 111 (56.0 bits)
Identities = 12/16 (75%), Positives = 16/16 (100%)

```
Query:   230 KDLLTGNEMFSDTYKL 245
             KD++TG+EMFSDTYK+
Sbjct:     8 KDIITGDEMFSDTYKI 23
```

Score = 91 (46.2 bits)
Identities = 12/15 (80%), Positives = 14/15 (93%)

```
Query:   139 KLVNEVLYEVTGRLV 153
             KLV+EV+YEVTG LV
Sbjct:    24 KLVDEVIYEVTGKLV 38
```

Score = 65 (33.5 bits)
Identities = 9/11 (81%), Positives = 11/11 (100%)

```
Query:    33 LVQTFAFGDKK 43
             LV+T+AFGDKK
Sbjct:    77 LVETYAFGDKK 87
```

HaEST hargln|HA-GLN Contig 461 FWD frame 2 Total Score: 404

```
                      0       60       120      180      240
                      |       |        |        |        |   |271
hargln|HA-GLN_Contig_ | ________________________________________
Local hits (HSPs)     |    ______     __
```

Score = 122 (61.3 bits)
Identities = 14/16 (87%), Positives = 16/16 (100%)

```
Query:   110 YKDLLTGDEMFSDTYK 125
             YKD++TGDEMFSDTYK
Sbjct:    35 YKDIITGDEMFSDTYK 50
```

Score = 111 (56.0 bits)
Identities = 12/16 (75%), Positives = 16/16 (100%)

```
Query:   230 KDLLTGNEMFSDTYKL 245
             KD++TG+EMFSDTYK+
Sbjct:    36 KDIITGDEMFSDTYKI 51
```

Score = 99 (50.1 bits)
Identities = 13/15 (86%), Positives = 15/15 (100%)

```
Query:   139 KLVNEVLYEVTGRLV 153
             KLV+EV+YEVTGRLV
Sbjct:    52 KLVDEVIYEVTGRLV 66
```

Score = 72 (36.9 bits)
Identities = 10/11 (90%), Positives = 11/11 (100%)

```
Query:    33 LVQTFAFGDKK 43
             LV+TFAFGDKK
Sbjct:   105 LVETFAFGDKK 115
```

Spot 27

m/z	Charge	Peak mW	Peptide mW	Delta (Da)	Delta (ppm)	Ladder Score	Log Likelihood	Start	End	Sequence	Modifications	Subs	Query Tool
502,7378	2	1003,4600	1003,4581	0,0019	1,8695	57,7778	60,7792	1	8	LPPCTCTR	Carbamidomethyl C	4 / 6	De-novo
502,7378	2	1003,4600	1003,458	0,0020	1,9692	44,4444	58,9608	1	8	LPPCCTTR	Carbamidomethyl C	4 / 5	De-novo
611,7625	2	1221,5094	1221,5587	-0,0493	-40,3779	36,5079	61,0647	1	11	SLEPVEFMTGP	Oxidation M	8	De-novo
592,8159	2	1183,6162	1183,6196	-0,0034	-2,8928	36,5079	59,2692	1	11	LPNGDLQLGTR	Deamidation N	3	De-novo

NCBI gi|707031|gb|AAB32409.1| 18 kda serine proteinase inhibitor [Helicoverpa zea] Total Score: 63

```
                      0          10         20
                      |          |          |          |29
gi|707031|gb|AAB32409 | ________________________________________
Local hits (HSPs)     | _______
```

Score = 63 (33.7 bits)
Identities = 8/8 (100%), Positives = 8/8 (100%)

```
Query:     1 LPPCTCTR 8
             LPPCTCTR
Sbjct:     1 LPPCTCTR 8   MatchSeq
```

ButterflyBase MSP00420_1 Total Score: 63

```
                      0       40       80       120      160
                      |       |        |        |        |     |193
db|MSP00420_1|MSP0042 | ________________________________________
Local hits (HSPs)     |     _
```

Score = 63 (33.7 bits)
Identities = 8/8 (100%), Positives = 8/8 (100%)

```
Query:     1 LPPCTCTR 8
             LPPCTCTR
Sbjct:    20 LPPCTCTR 27
```

Ha SG EST hargln|HA-GLN Contig 831 FWD frame 2 Total Score: 63

```
                      0       80       160      240      320
                      |       |        |        |        |   |353
hargln|HA-GLN_Contig_ | ________________________________________
Local hits (HSPs)     |           _
```

Score = 63 (33.7 bits)
Identities = 8/8 (100%), Positives = 8/8 (100%)

```
Query:     1 LPPCTCTR 8
             LPPCTCTR
Sbjct:   102 LPPCTCTR 109
```

Spot 28

m/z	Charge	Peak mW	Peptide mW	Delta (Da)	Delta (ppm)	Ladder Score	Log Likelihood	Start	End	Sequence	Modifications	Subs	Query Tool
478,2376	2	954,4596	954,5651	-0,1055	-110,5467	44,4444	69,7244	1	8	KFTVPHVK			De-novo
478,2376	2	954,4596	954,5651	-0,1055	-110,5467	42,2222	69,4463	1	8	FKTVPHVK			De-novo
626,2733	2	1250,5310	1250,5748	-0,0438	-35,0431	49,3333	230,3754	1	13	SCGNGVGCSLAVK	Carbamidomethyl C	2	De-novo
626,2733	2	1250,5310	1250,5748	-0,0438	-35,0431	49,3333	228,6123	1	13	CSGNGVGCSLAVK	Carbamidomethyl C	1	De-novo
626,2733	2	1250,5310	1250,5747	-0,0437	-34,9631	53,0864	234,4568	1	14	SGCGNGVGCSLAVK			De-novo
592,8044	2	1183,5932	1183,6196	-0,0264	-22,3247	82,5397	331,0312	1	11	LPDGNLGTQLR	Deamidation N	5	De-novo
592,8044	2	1183,5932	1183,6196	-0,0264	-22,3247	82,5397	328,3769	1	11	LPNGNLGTQLR	Deamidation N	3 / 5	De-novo
592,8044	2	1183,5932	1183,6196	-0,0264	-22,3247	82,5397	327,0154	1	11	LPNGDLGTQLR	Deamidation N	3	De-novo
649,3719	2	1296,7282	1296,7401	-0,0119	-9,1954	42,029	56,9511	1	12	EVLVSALPRDAK			De-novo
649,3719	2	1296,7282	1296,7401	-0,0119	-9,1954	33,3333	56,2458	1	12	LEVVSALPRDAK			De-novo
649,3719	2	1296,7282	1296,7401	-0,0119	-9,1954	33,3333	56,2182	1	12	ELVVSALPRDAK			De-novo
1273,0137	2	2544,0118	2544,0349	-0,0231	-9,0895	40,1361	214,8135	1	25	FHQESGEGLNAPDTGGSTSSPNPPC	Deamidation Q /	3 / 10 / 2	De-novo
1273,0137	2	2544,0118	2544,0349	-0,0231	-9,0895	38,7755	217,5254	1	25	FHQESGENLGAPDTGGSTSSPNPPC	Deamidation Q /	3 / 8 / 25	De-novo
1273,0137	2	2544,0118	2544,071	-0,0592	-23,2792	37,415	216,8923	1	25	FHQESGTNGLVPDTGGSTSSPNPPC	Deamidation Q /	3 / 8 / 25	De-novo
883,8964	2	1765,7772	1765,7943	-0,0171	-9,6976	47,4747	175,9045	1	17	ELMTNGSGDLSGWGNTK			De-novo
883,8964	2	1765,7772	1765,7943	-0,0171	-9,6976	47,4747	175,4103	1	17	LEMTNGSGDLSGWGNTK			De-novo
883,8964	2	1765,7772	1765,7943	-0,0171	-9,6976	47,4747	174,2811	1	17	QLMTNGSGDLSGWGNTK	Deamidation Q	1	De-novo
413,2132	2	824,4108	824,4102	0,0006	0,6987	38,4615	51,8308	1	7	GNFVMLK	Deamidation N / Oxidation	2 / 5	De-novo
413,2132	2	824,4108	824,4102	0,0006	0,6987	38,4615	50,825	1	7	FGNVMLK	Deamidation N / Oxidation	3 / 5	De-novo
737,7045	3	2210,0901	2210,0525	0,0376	16,9969	41,4634	97,2354	1	21	RDGFSQETLGAGLVDLVSCDK	Deamidation Q	6	De-novo
737,7045	3	2210,0901	2210,0676	0,0225	10,1644	34,9594	97,1948	1	21	LFTSQHAYSGAGLVDLVCSNK	Deamidation N	20	De-novo
737,7045	3	2210,0901	2210,0525	0,0376	16,9969	44,7154	97,0884	1	21	DRGFSQETLGAGLVDLVSCDK	Deamidation Q	6	De-novo

NCBI gi|108880629|gb|EAT44854.1| initiation factor 5a [Aedes aegypti] Total Score: 115

```
                      0       40       80       120      160
                      |       |        |        |        |160
gi|108880629|gb|EAT44 | ________________________________________
Local hits (HSPs)     |                     __     _____
```

Score = 77 (41.0 bits)
Identities = 13/20 (65%), Positives = 16/20 (80%)

```
Query:    70 NGNLGTQLRLPNGDLGTQLR 89
             NG+L   L +P+GDLGTQLR
Sbjct:   107 NGDLREDLKIPDGDLGTQLR 126   MatchSeq
```

Score = 38 (21.1 bits)
Identities = 5/5 (100%), Positives = 5/5 (100%)

```
Query:     4 VPHVK 8
             VPHVK
Sbjct:    83 VPHVK 87   MatchSeq
```

ButterflyBase SEP00040_1 Total Score: 115

```
                      0       40       80       120      160
                      |       |        |        |        |160
db|SEP00040_1|SEP0004 | ________________________________________
Local hits (HSPs)     |                     _      _____
```

Score = 77 (41.0 bits)
Identities = 13/20 (65%), Positives = 16/20 (80%)

```
Query:    70 NGNLGTQLRLPNGDLGTQLR 89
             NG+L   L +P+GDLGTQLR
Sbjct:   108 NGDLREDLKIPDGDLGTQLR 127
```

Score = 38 (21.1 bits)
Identities = 5/5 (100%), Positives = 5/5 (100%)

```
Query:     4 VPHVK 8
             VPHVK
Sbjct:    83 VPHVK 87
```

Ha SG EST hargln|HA-GLN Contig 1601 FWD frame 1 Total Score: 115

```
                      0       50       100      150      200
                      |       |        |        |        |     |234
hargln|HA-GLN_Contig_ | ________________________________________
Local hits (HSPs)     |                    _    _____
```

Score = 77 (41.0 bits)
Identities = 13/20 (65%), Positives = 16/20 (80%)

```
Query:    70 NGNLGTQLRLPNGDLGTQLR 89
             NG+L   L +P+GDLGTQLR
Sbjct:   136 NGDLREDLKIPDGDLGTQLR 155
```

Score = 38 (21.1 bits)
Identities = 5/5 (100%), Positives = 5/5 (100%)

```
Query:     4 VPHVK 8
             VPHVK
Sbjct:   111 VPHVK 115
```

Spot 29

m/z	Charge	Peak mW	Peptide mW	Delta (Da)	Delta (ppm)	Ladder Score	Log Likelihood	Start	End	Sequence	Modifications	Subs	Query Tool
423,2399	2	844,4642	844,4655	-0,0013	-1,5679	45,098	126,8586	1	9	GQAVALTGK	Deamidation Q	2	De-novo
423,2399	2	844,4642	844,4655	-0,0013	-1,5679	45,098	126,5853	1	9	QGAVALTGK	Deamidation Q	1	De-novo
423,2399	2	844,4642	844,4655	-0,0013	-1,5679	45,098	128,3442	1	9	NAAVALTGK	Deamidation N	1	De-novo
613,3261	2	1224,6366	1224,6462	-0,0096	-7,8586	71,0145	216,0125	1	12	NLVSETAHGAVK			De-novo
613,3261	2	1224,6366	1224,6462	-0,0096	-7,8586	74,6032	213,9837	1	11	NLVSETAHQVK			De-novo
437,7481	2	873,4806	873,4782	0,0024	2,7202	61,5385	97,6843	1	7	WLMSPLK			De-novo
437,7481	2	873,4806	873,4266	0,0540	61,7980	66,6667	98,7714	1	8	SPMDSPLK			De-novo
437,7481	2	873,4806	873,4266	0,0540	61,7980	60	97,5111	1	8	SPDMSPLK			De-novo
616,8043	2	1231,5930	1231,6383	-0,0453	-36,7998	40,5797	36,9889	1	12	CLRGGLSYLGPP			De-novo

NCBI gi|194151917|gb|EDW67351.1| GJ23129 [Drosophila virilis] Total Score: 68

```
                        0       210      420     630      840
                        |        |        |       |        |       |1016
gi|194151917|gb|EDW67   | ________________________________________
Local hits (HSPs)       |                                  __
```

Score = 68 (36.3 bits)
Identities = 12/18 (66%), Positives = 12/18 (66%)

```
Query:    54 SPLKSPMDSPLKSPDMSP 71
             SP KSP  SP KSP  SP
Sbjct:   837 SPAKSPVKSPVKSPAKSP 854   MatchSeq
```

ButterflyBase None

Ha SG EST ^ = hargln|HA-GLN Contig 3403 REV frame 0 Total Score: 108

```
                        0        40       80       120      160
                        |         |        |         |        |  |173
hargln|HA-GLN_Contig_   | ________________________________________
Local hits (HSPs)       |              __
```

Score = 56 (30.2 bits)
Identities = 7/9 (77%), Positives = 8/9 (88%)

```
Query:    50 KWLMSPLKS 58
             KWLMS +KS
Sbjct:    60 KWLMSTIKS 68
```

Score = 52 (28.2 bits)
Identities = 7/11 (63%), Positives = 8/11 (72%)

```
Query:   115 GAVKWLMSPLK 125
             G  KWLMS +K
Sbjct:    57 GTTKWLMSTIK 67
```

Spot 30

m/z	Charge	Peak mW	Peptide mW	Delta (Da)	Delta (ppm)	Ladder Score	Log Likelihood	Start	End	Sequence	Modifications	Subs	Query Tool
577,8114	2	1153,6072	1153,6091	-0,0019	-1,6678	63,7681	214,8044	1	12	NLVSGTVGAHAK	Deamidation N	1	De-novo
577,8114	2	1153,6072	1153,6091	-0,0019	-1,6678	61,9048	211,978	1	11	NLVSGTVQHAK	Deamidation N	1	De-novo
746,3951	2	1490,7746	1490,8093	-0,0347	-23,2920	50,6667	68,9748	1	13	KYTLGLEQGDVLR			De-novo
746,3951	2	1490,7746	1490,8093	-0,0347	-23,2920	50,6667	69,4592	1	13	KYTLGLEQGNVLR	Deamidation N	10	De-novo
746,3951	2	1490,7746	1490,8093	-0,0347	-23,2920	49,3333	68,8448	1	13	YKTLGLEQGDVLR			De-novo
562,3109	2	1122,6062	1122,6073	-0,0011	-1,0012	66,6667	163,3145	1	9	LFTPLTQFR	Deamidation Q	7	De-novo
562,3109	2	1122,6062	1122,6073	-0,0011	-1,0012	66,6667	163,5619	1	9	LFTPLTEFR			De-novo
562,3109	2	1122,6062	1122,6073	-0,0011	-1,0012	64,7059	156,5331	1	9	LFTPLETFR			De-novo
457,7912	2	913,5668	913,567	-0,0002	-0,2452	57,7778	103,3218	1	8	MSLLLPLK			De-novo
457,7912	2	913,5668	913,567	-0,0002	-0,2452	57,7778	102,8406	1	8	SMLLLPLK			De-novo
457,7912	2	913,5668	913,567	-0,0002	-0,2452	55,5556	90,448	1	8	AMLLLPLK	Oxidation M	2	De-novo
530,29	2	1058,5644	1058,5608	0,0036	3,3782	64,7059	156,0862	1	9	ELELTQGLR	Deamidation Q	6	De-novo
530,29	2	1058,5644	1058,5608	0,0036	3,3782	64,7059	154,702	1	9	ELELTEGLR			De-novo
530,29	2	1058,5644	1058,5608	0,0036	3,3782	64,7059	154,5152	1	9	QLELTQGLR	Deamidation Q	1 / 6	De-novo

NCBI gi|156968287|gb|ABU98615.1| fructosidase [Helicoverpa armigera] Total Score: 68

```
                        0       100      200     300      400
                        |        |        |       |        |       |479
gi|156968287|gb|ABU98   | ________________________________________
Local hits (HSPs)       |                           _
```

Score = 68 (35.9 bits)
Identities = 10/10 (100%), Positives = 10/10 (100%)

```
Query:   122 RELELTEGLR 131
             RELELTEGLR
Sbjct:   320 RELELTEGLR 329   MatchSeq
```

ButterflyBase None

Ha SG EST ^ = hargln|HA-GLN Contig 1323 FWD frame 0 Total Score: 156

```
                        0       150      300     450      600
                        |        |        |       |        |       |747
hargln|HA-GLN_Contig_   | ________________________________________
Local hits (HSPs)       |      _        _
```

Score = 69 (36.4 bits)
Identities = 9/12 (75%), Positives = 11/12 (91%)

```
Query:    12 KNLVSGTVQHAK 23
             K++VSGTVQH K
Sbjct:   264 KDIVSGTVQHVK 275
```

Score = 45 (24.3 bits)
Identities = 5/9 (55%), Positives = 8/9 (88%)

```
Query:   139 LRNLVSGTV 147
             + ++VSGTV
Sbjct:   263 IKDIVSGTV 271
```

Score = 42 (22.8 bits)
Identities = 5/8 (62%), Positives = 8/8 (100%)

```
Query:   108 LLLPLKEL 115
             LLLP+K++
Sbjct:   117 LLLPIKDI 124
```

Spot 31

m/z	Charge	Peak mW	Peptide mW	Delta (Da)	Delta (ppm)	Ladder Score	Log Likelihood	Start	End	Sequence	Modifications	Subs	Query Tool
470,2336	2	938,4516	938,4532	-0,0016	-1,7305	53,3333	57,7281	1	8	PALSYTCK	Carbamidomethyl C	7	De-novo
470,2336	2	938,4516	938,4532	-0,0016	-1,7305	53,3333	55,4898	1	8	APLSYTCK	Carbamidomethyl C	7	De-novo
470,2336	2	938,4516	938,4532	-0,0016	-1,7305	44,4444	53,575	1	8	PLASYTCK	Carbamidomethyl C	7	De-novo
598,3458	2	1194,6760	1194,672	0,0040	3,3281	46,6667	69,8862	1	13	GAGVPLTVPAGTR			De-novo
598,3458	2	1194,6760	1194,672	0,0040	3,3281	46,6667	68,711	1	13	AGGVPLTVPAGTR			De-novo
598,3458	2	1194,6760	1194,672	0,0040	3,3281	38,6667	67,053	1	13	GAGVPLSLPAGTR			De-novo
684,3764	2	1366,7372	1366,7456	-0,0084	-6,1635	77,3333	287,7646	1	13	VVPATDSLVVPNR	Deamidation N	12	De-novo
684,3764	2	1366,7372	1366,7456	-0,0084	-6,1635	77,3333	286,7009	1	13	VVPATNSLVVPNR	Deamidation N	6 / 12	De-novo
684,3764	2	1366,7372	1366,7456	-0,0084	-6,1635	77,3333	283,9026	1	13	VVPATDSLVVPDR			De-novo
635,8633	2	1269,7110	1269,6829	0,0281	22,1126	30,1587	22,8321	1	11	EFAGEPVLRPR			De-novo
830,903	2	1659,7904	1659,8025	-0,0121	-7,3045	37,931	77,2989	1	15	TLSCLELEESAQLQP			De-novo
830,903	2	1659,7904	1659,8025	-0,0121	-7,3045	37,931	78,531	1	15	TLSCLELQESAQLQP	Deamidation Q	8	De-novo
731,3917	2	1460,7678	1460,7622	0,0056	3,8172	66,6667	214,1243	1	14	TLQGGNAYPSVVVR	Deamidation N	6	De-novo
731,3917	2	1460,7678	1460,7622	0,0056	3,8172	68	210,7415	1	13	TLQNDAYPSVVVR			De-novo
731,3917	2	1460,7678	1460,7622	0,0056	3,8172	68	211,2868	1	13	TLQNNAYPSVVVR	Deamidation N	5	De-novo

NCBI gi|74778861|sp|P82171.1|CU095_LOCMI RecName: Full=Cuticle protein 9.5; AltName: Full=LmNCP9.5 Total Score: 187

```
                        0        20       40       60       80
                        |         |        |        |        |    |90
gi|74778861|sp|P82171   | ________________________________________
Local hits (HSPs)       | ___            _____    ____   ___
```

Score = 45 (24.4 bits)
Identities = 7/9 (77%), Positives = 7/9 (77%)

```
Query:    72 VVPNRVVPA 80
             VV  RVVPA
Sbjct:    34 VVAGRVVPA 42   MatchSeq
```

Score = 41 (22.4 bits)
Identities = 6/6 (100%), Positives = 6/6 (100%)

```
Query:    31 TVPAGT 36
             TVPAGT
Sbjct:     2 TVPAGT 7   MatchSeq
```

Score = 35 (19.4 bits)
Identities = 5/5 (100%), Positives = 5/5 (100%)

```
Query:    64 VVPAT 68
             VVPAT
Sbjct:    57 VVPAT 61   MatchSeq
```

Score = 33 (18.4 bits)
Identities = 5/6 (83%), Positives = 5/6 (83%)

```
Query:   151 YPSVVV 156
             YPS VV
Sbjct:    53 YPSGVV 58   MatchSeq
```

Score = 33 (18.4 bits)
Identities = 5/8 (62%), Positives = 5/8 (62%)

```
Query:   175 NAYPSVVV 182
             N YP  VV
Sbjct:    70 NVYPAGVV 77   MatchSeq
```

ButterflyBase TBP00299_1 Total Score: 160

```
                        0          30         60         90
                        |           |          |          |       |112
db|TBP00299_1|TBP0029   | ________________________________________
Local hits (HSPs)       |                ______________
```

Score = 85 (44.5 bits)
Identities = 16/37 (43%), Positives = 20/37 (54%)

```
Query:    64 VVPATDSLVVPNRVVPATNSLVVPNRVVPATDSLVVP 100
             VVP   S+V P  VVP   S++ P  V P   S+V P
Sbjct:    45 VVPEVPSIVLPTPVVPEIPSIILPTPVLPENPSIVLP 81
```

Score = 75 (39.5 bits)
Identities = 14/31 (45%), Positives = 17/31 (54%)

```
Query:    62 TRVVPATDSLVVPNRVVPATNSLVVPNRVVP 92
             T VVP   S++ P  V P   S+V P  VVP
Sbjct:    56 TPVVPEIPSIILPTPVLPENPSIVLPTPVVP 86
```

Ha SG EST None

Ha EST other harmi_fn|HA-FN-ALL Contig 610 FWD frame 0 Total Score: 251

```
                        0         40         80        120       160
                        |          |          |         |         |162
harmi_fn|HA-FN-ALL_Co   | ________________________________________
Local hits (HSPs)       |       ____  ______
```

Score = 101 (52.6 bits)
Identities = 14/15 (93%), Positives = 15/15 (100%)

```
Query:   196 RTLQGGNAYPSVVVR 210
             RTLQGG+AYPSVVVR
Sbjct:    56 RTLQGGDAYPSVVVR 70
```

Score = 91 (47.6 bits)
Identities = 12/14 (85%), Positives = 13/14 (92%)

```
Query:    89 RVVPATDSLVVPDR 102
             RVVPATDS+V PDR
Sbjct:    27 RVVPATDSIVAPDR 40
```

Score = 59 (31.5 bits)
Identities = 8/8 (100%), Positives = 8/8 (100%)

```
Query:     9 APLSYTCK 16
             APLSYTCK
Sbjct:    47 APLSYTCK 54
```

Spot 32

m/z	Charge	Peak mW	Peptide mW	Delta (Da)	Delta (ppm)	Ladder Score	Log Likelihood	Start	End	Sequence	Modifications	Subs	Query Tool
590,3074	2	1178,5992	1178,5931	0,0061	5,1553	70,5882	97,1396	1	9	YEELQLTQR			De-novo
590,3074	2	1178,5992	1178,5931	0,0061	5,1553	70,5882	96,179	1	9	YEELLQTQR			De-novo
590,3074	2	1178,5992	1178,5931	0,0061	5,1553	70,5882	96,3214	1	9	YQELQLTQR	Deamidation Q	2	De-novo
666,3531	2	1330,6906	1330,7244	-0,0338	-25,4177	62,3188	343,6449	1	12	NANLVNALVQFK	Deamidation N	3	De-novo
529,7229	2	1057,4302	1057,4321	-0,0019	-1,8195	73,3333	182,8366	1	8	LMNAYCNR	Oxidation M /	2 / 6 / 7	De-novo
529,7229	2	1057,4302	1057,4386	-0,0084	-7,9664	74,5098	187,2683	1	9	LMNAYTNSQ	Oxidation M / Deamidation	2 / 7	De-novo
529,7229	2	1057,4302	1057,4386	-0,0084	-7,9664	72,549	188,3332	1	9	LMNAYTSNQ	Oxidation M / Deamidation	2 / 8	De-novo

NCBI gi|77862436|gb|ABB04499.1| SUMO [Bombyx mori] Total Score: 122

```
                        0        20       40       60       80
                        |         |        |        |        |   |91
gi|77862436|gb|ABB044   | ________________________________________
Local hits (HSPs)       |           ___    ____
```

Score = 67 (34.4 bits)
Identities = 8/9 (88%), Positives = 9/9 (100%)

```
Query:    39 KLMNAYCNR 47
             KLMNAYC+R
Sbjct:    36 KLMNAYCDR 44   MatchSeq
```

Score = 55 (28.6 bits)
Identities = 6/8 (75%), Positives = 8/8 (100%)

```
Query:    33 NALVQFKL 40
             NA+VQFK+
Sbjct:    21 NAIVQFKI 28   MatchSeq
```

Ha SG EST hargln|HA-GLN Contig 1326 FWD frame 0 Total Score: 122

```
                        0        60      120      180      240
                        |         |        |        |        |   |286
hargln|HA-GLN_Contig_   | ________________________________________
Local hits (HSPs)       |          _ _
```

Score = 67 (34.4 bits)
Identities = 8/9 (88%), Positives = 9/9 (100%)

```
Query:    39 KLMNAYCNR 47
             KLMNAYC+R
Sbjct:    78 KLMNAYCDR 86
```

Score = 55 (28.6 bits)
Identities = 6/8 (75%), Positives = 8/8 (100%)

```
Query:    33 NALVQFKL 40
             NA+VQFK+
Sbjct:    63 NAIVQFKI 70
```

Spot 33

m/z	Charge	Peak mW	Peptide mW	Delta (Da)	Delta (ppm)	Ladder Score	Log Likelihood	Start	End	Sequence	Modifications	Subs	Query Tool
534,7527	2	1067,4898	1067,4917	-0,0019	-1,8024	63,1579	79,0997	1	10	MVTGSTSVNR	Oxidation M / Deamidation	1 / 9	De-novo
534,7527	2	1067,4898	1067,4917	-0,0019	-1,8024	57,8947	77,0157	1	10	MVTGSTVSNR	Oxidation M / Deamidation	1 / 9	De-novo
534,7527	2	1067,4898	1067,4917	-0,0019	-1,8024	56,1404	75,3689	1	10	MVTGSLSSNR	Oxidation M / Deamidation	1 / 9	De-novo
526,7569	2	1051,4982	1051,4968	0,0014	1,3086	70,1754	130,3248	1	10	VMTGSLSSNR	Deamidation N	9	De-novo
526,7569	2	1051,4982	1051,4968	0,0014	1,3086	70,1754	125,8716	1	10	VMGTSLSSNR	Deamidation N	9	De-novo
526,7569	2	1051,4982	1051,4968	0,0014	1,3086	70,1754	123,037	1	10	VMTGSLSNSR	Deamidation N	8	De-novo
468,2362	2	934,4568	934,4582	-0,0014	-1,5239	71,1111	138,4296	1	8	WLVMDAGK	Oxidation M	4	De-novo
540,291	2	1078,5664	1078,5658	0,0006	0,5340	69,8413	212,8752	1	11	QTLAGFASGVK	Deamidation Q	1	De-novo
540,291	2	1078,5664	1078,5659	0,0005	0,4413	66,6667	213,0535	1	10	QTLQFASGVK	Deamidation Q	1	De-novo
540,291	2	1078,5664	1078,5658	0,0006	0,5340	60,3175	213,7563	1	11	QTLGAFASGVK	Deamidation Q	1	De-novo
613,8234	2	1225,6312	1225,6489	-0,0177	-14,4609	69,8413	113,689	1	11	LPNLLANSPCK	Carbamidomethyl C	10	De-novo
613,8323	2	1225,6490	1225,6489	0,0001	0,0620	84,127	377,259	1	11	LPNLLANSPCK	Carbamidomethyl C	10	De-novo
613,8323	2	1225,6490	1225,6489	0,0001	0,0620	84,127	377,259	1	11	LPNLLANSPCK			De-novo
613,8234	2	1225,6312	1225,6488	-0,0176	-14,3793	63,4921	111,5332	1	11	PNLLLANSPCK	Carbamidomethyl C	10	De-novo
613,8234	2	1225,6312	1225,6488	-0,0176	-14,3793	57,1429	111,2558	1	11	NPLLLANSPCK	Carbamidomethyl C	10	De-novo
530,2894	2	1058,5632	1058,5648	-0,0016	-1,5342	68,6274	88,1441	1	9	QLELAPKYP	Deamidation Q	1	De-novo
883,8987	2	1765,7818	1765,7943	-0,0125	-7,0926	45,4545	105,0198	1	17	PQMSGGKSDLSGWGNTK	Deamidation Q / Oxidation	2 / 3	De-novo
883,8987	2	1765,7818	1765,7943	-0,0125	-7,0926	46,2366	102,4382	1	16	DLCQGKSDLSGWGNTK	Carbamidomethyl C /	3 / 4	De-novo
883,8987	2	1765,7818	1765,7942	-0,0124	-7,0359	43,4343	107,4052	1	17	SGTCQPKSDLSGWGNTK	Deamidation Q	5	De-novo
513,7725	2	1025,5294	1025,5004	0,0290	28,2555	60	177,5908	1	8	MLFPYPSR	Oxidation M	1	De-novo
513,7725	2	1025,5294	1025,5005	0,0289	28,1580	64,4444	176,8465	1	8	LMFPYPSR	Oxidation M	2	De-novo
513,7725	2	1025,5294	1025,4971	0,0323	31,4735	60	175,941	1	8	YPFPYPSR			De-novo
562,3138	2	1122,6120	1122,6073	0,0047	4,1653	62,7451	111,4369	1	9	LFTPLTEFR			De-novo
562,3138	2	1122,6120	1122,6073	0,0047	4,1653	62,7451	109,7237	1	9	LFTPLTQFR	Deamidation Q	7	De-novo
562,3138	2	1122,6120	1122,6073	0,0047	4,1653	50,9804	109,0695	1	9	LFTPLQTFR	Deamidation Q	6	De-novo
583,7645	2	1165,5134	1165,5033	0,0101	8,6452	82,5397	154,1527	1	11	TSQSAAQAMNR	Deamidation Q /	7 / 10	De-novo
583,7645	2	1165,5134	1165,5033	0,0101	8,6452	82,5397	154,0635	1	11	TSQSAAEAMNR	Deamidation N	10	De-novo
494,7638	2	987,5120	987,5138	-0,0018	-1,8471	70,5882	147,899	1	9	ALSPYQPGR			De-novo
494,7638	2	987,5120	987,5139	-0,0019	-1,9483	70,5882	145,8335	1	9	LASPYQPGR			De-novo
494,7638	2	987,5120	987,5138	-0,0018	-1,8471	61,4035	143,2425	1	10	ALSPYGAPGR			De-novo
604,3211	2	1206,6266	1206,6244	0,0022	1,8034	49,2063	168,6454	1	11	NLATGANLLYR	Deamidation N	1 / 7	De-novo
613,8323	2	1225,6490	1225,6489	0,0001	0,0620	72,4638	366,9989	1	12	LPNLLAGGSPCK	Carbamidomethyl C	11	De-novo
613,8323	2	1225,6490	1225,6488	0,0002	0,1436	74,6032	365,9993	1	11	LNPLLANSPCK	Carbamidomethyl C	10	De-novo

NCBI gi|86450717|gb|ABC96694.1| yellow-d [Bombyx mori] Total Score: 503

```
                       0       90      180     270     360
                       |        |       |       |       |      |446
gi|86450717|gb|ABC966  | _________________________________________
Local hits (HSPs)      |              _        _ _     _  ___ ___
```

```
 Score = 82 (43.2 bits)
 Identities = 11/12 (91%), Positives = 12/12 (100%)

Query:   276 RTSQSAAEAMNR 287
             RTSQSAAEAM+R
Sbjct:   307 RTSQSAAEAMDR 318   MatchSeq
```

```
 Score = 74 (39.2 bits)
 Identities = 9/12 (75%), Positives = 12/12 (100%)

Query:    30 RVMTGSLSSNRV 41
             RVMTGS+SS+R+
Sbjct:   383 RVMTGSISSDRI 394   MatchSeq
```

```
 Score = 62 (33.1 bits)
 Identities = 9/11 (81%), Positives = 10/11 (90%)

Query:    79 KQTLQFASGVK 89
             K+TLQFASG K
Sbjct:   353 KETLQFASGLK 363   MatchSeq
```

```
 Score = 60 (32.1 bits)
 Identities = 7/11 (63%), Positives = 10/11 (90%)

Query:   136 LLLANSPCKNP 146
             ++LA+SPC NP
Sbjct:   405 IILADSPCTNP 415   MatchSeq
```

```
 Score = 57 (30.6 bits)
 Identities = 7/11 (63%), Positives = 10/11 (90%)

Query:   100 KLPNLLANSPC 110
             K+P +LA+SPC
Sbjct:   402 KIPIILADSPC 412   MatchSeq
```

```
 Score = 49 (26.6 bits)
 Identities = 6/6 (100%), Positives = 6/6 (100%)

Query:   217 FPYPSR 222
             FPYPSR
Sbjct:   229 FPYPSR 234   MatchSeq
```

```
 Score = 43 (23.6 bits)
 Identities = 7/9 (77%), Positives = 7/9 (77%)

Query:   288 ALSPYQPGR 296
             ALSPY  GR
Sbjct:   254 ALSPYRTGR 262   MatchSeq
```

```
 Score = 40 (22.0 bits)
 Identities = 5/6 (83%), Positives = 6/6 (100%)

Query:    63 VMDAGK 68
             +MDAGK
Sbjct:   137 IMDAGK 142   MatchSeq
```

```
 Score = 36 (20.0 bits)
 Identities = 6/9 (66%), Positives = 6/9 (66%)

Query:    95 FASGVKLPN 103
             FASG K  N
Sbjct:   358 FASGLKVVN 366   MatchSeq
```

Ha SG EST hargln|HA-GLN Contig 285 FWD frame 1 Total Score: 481

```
                       0       70      140      210      280
                       |        |       |        |        |      |333
hargln|HA-GLN_Contig_  | _________________________________________
Local hits (HSPs)      |   _  _       _      _  __ ___
```

```
 Score = 82 (43.2 bits)
 Identities = 11/12 (91%), Positives = 12/12 (100%)

Query:   276 RTSQSAAEAMNR 287
             RTSQSAAEAM+R
Sbjct:   111 RTSQSAAEAMDR 122
```

```
 Score = 75 (39.7 bits)
 Identities = 9/12 (75%), Positives = 11/12 (91%)

Query:   100 KLPNLLANSPCK 111
             K+PN+LA SPCK
Sbjct:   206 KIPNILAXSPCK 217
```

```
 Score = 71 (37.7 bits)
 Identities = 10/12 (83%), Positives = 11/12 (91%)

Query:    30 RVMTGSLSSNRV 41
             RVMTGSLSS+ V
Sbjct:   187 RVMTGSLSSDXV 198
```

```
 Score = 58 (31.1 bits)
 Identities = 8/9 (88%), Positives = 8/9 (88%)

Query:   222 RLMFPYPSR 230
             RL FPYPSR
Sbjct:    30 RLFFPYPSR 38
```

```
 Score = 56 (30.1 bits)
 Identities = 8/11 (72%), Positives = 9/11 (81%)

Query:    79 KQTLQFASGVK 89
             K+TLQFAS  K
Sbjct:   157 KETLQFASXXK 167
```

```
 Score = 53 (28.6 bits)
 Identities = 8/9 (88%), Positives = 8/9 (88%)

Query:   288 ALSPYQPGR 296
             ALSPYQ GR
Sbjct:    58 ALSPYQHGR 66
```

```
 Score = 49 (26.6 bits)
 Identities = 6/9 (66%), Positives = 8/9 (88%)

Query:   137 LLANSPCKN 145
             +LA SPCK+
Sbjct:   210 ILAXSPCKD 218
```

```
 Score = 37 (20.5 bits)
 Identities = 6/7 (85%), Positives = 6/7 (85%)

Query:   304 GRALSPY 310
             G ALSPY
Sbjct:    56 GMALSPY 62
```

Spot 34

m/z	Charge	Peak mW	Peptide mW	Delta (Da)	Delta (ppm)	Ladder Score	Log Likelihood	Start	End	Sequence	Modifications	Subs	Query Tool
595,319	2	1188,6224	1188,6251	-0,0027	-2,2917	63,1579	230,2309	1	10	QAQKPDVFTR			De-novo
595,319	2	1188,6224	1188,6251	-0,0027	-2,2917	57,1429	228,3984	1	11	QAGAKPDVFTR			De-novo
515,7728	2	1029,5300	1029,5283	0,0017	1,6279	60	173,8158	1	8	FLPHPEYK			De-novo
515,7728	2	1029,5300	1029,5283	0,0017	1,6279	62,2222	173,8321	1	8	LFPHPEYK			De-novo
515,7728	2	1029,5300	1029,5283	0,0017	1,6279	60	171,4549	1	8	FLPHPQYK	Deamidation Q	6	De-novo
500,2916	2	998,5676	998,5661	0,0015	1,4781	68,6274	121,7116	1	9	FLLTAAHAR			De-novo
500,2916	2	998,5676	998,5661	0,0015	1,4781	66,6667	116,7126	1	9	FLLTAAAHR			De-novo
500,2916	2	998,5676	998,5662	0,0014	1,3780	60,7843	115,6924	1	9	LFLTAAHAR			De-novo
514,2432	2	1026,4708	1026,4692	0,0016	1,5354	57,7778	120,9867	1	8	SDCLEYLK	Carbamidomethyl C	3	De-novo
514,2432	2	1026,4708	1026,4692	0,0016	1,5354	57,7778	120,261	1	8	SNCLEYLK	Deamidation N /	2 / 3	De-novo
717,3876	2	1432,7596	1432,735	0,0246	17,1532	34,6667	67,6407	1	13	GSEPLLHFGYSVK			De-novo
696,8577	2	1391,6998	1391,7296	-0,0298	-21,4295	73,913	297,3557	1	12	FADTLQTLLNEK			De-novo
696,8577	2	1391,6998	1391,7296	-0,0298	-21,4295	73,913	297,2601	1	12	FADTLQTLNLEK			De-novo
737,7049	3	2210,0913	2210,0969	-0,0056	-2,5501	46,3415	270,4977	1	21	NANHLLDVLEGNGAEFLNAAK	Deamidation N	3	De-novo
832,0638	3	2493,1680	2493,177	-0,0090	-3,6243	51,0204	399,993	1	25	TNAPEAYDGAASGEPLLVSNAGFSR			De-novo
832,0638	3	2493,1680	2493,177	-0,0090	-3,6243	51,0204	398,6352	1	25	TNAPQAYDGAASGEPLLVSNAGFSR	Deamidation Q	5	De-novo
832,0638	3	2493,1680	2493,177	-0,0090	-3,6243	51,0204	398,8272	1	25	TNAPEAYNGAASGEPLLVSNAGFSR	Deamidation N	8	De-novo
669,3417	3	2005,0017	2005,0045	-0,0028	-1,4145	54,5455	276,9122	1	17	LNHYALLETETPLTFNK	Deamidation N	2 / 16	De-novo
745,8591	2	1489,7026	1489,7269	-0,0243	-16,3278	62,069	301,1988	1	15	NVVMSPTAGPATAMK	Oxidation M	4	De-novo
745,8591	2	1489,7026	1489,7269	-0,0243	-16,3278	63,2184	295,5517	1	15	NVVMSPTAPATGAMK	Oxidation M	4	De-novo
638,8317	2	1275,6478	1275,6433	0,0045	3,5088	56,1404	73,938	1	10	VLELPYHCFR			De-novo
638,8317	2	1275,6478	1275,6433	0,0045	3,5088	56,1404	73,8285	1	10	VLELPYCHFR			De-novo
638,8317	2	1275,6478	1275,6433	0,0045	3,5088	56,1404	74,3397	1	10	VLQLPYHCFR	Deamidation Q	3	De-novo
691,8452	2	1381,6748	1381,6627	0,0121	8,7402	60	206,3654	1	13	SLNPPSSASAWHK	Deamidation N	3	De-novo
691,8452	2	1381,6748	1381,6627	0,0121	8,7402	58,6667	206,204	1	13	LSNPPSSASAWHK	Deamidation N	3	De-novo
1003,5118	2	2005,0080	2005,0197	-0,0117	-5,8473	52,5253	187,1678	1	17	HYDLALLDPFTPLTFDK			De-novo
1003,5118	2	2005,0080	2005,0197	-0,0117	-5,8473	50,5051	187,0147	1	17	YHDLALLDPFTPLTFDK			De-novo
1003,5118	2	2005,0080	2005,0197	-0,0117	-5,8473	52,5253	185,6044	1	17	HYDLALLPDFTPLTFDK			De-novo

NCBI gi|157106438|ref|XP_001649322.1| proacrosin, putative [Aedes aegypti] Total Score: 212

```
                       0       80      160      240      320
                       |        |       |        |        |      |385
gi|157106438|ref|XP_0  | _________________________________________
Local hits (HSPs)      |                   _  _ __
```

```
 Score = 70 (36.8 bits)
 Identities = 10/17 (58%), Positives = 14/17 (82%)

Query:   341 KYHDLALLDPFTPLTFD 357
             KYHD+AL++  TP T+D
Sbjct:   217 KYHDIALIELVTPFTYD 233   MatchSeq
```

```
 Score = 54 (28.8 bits)
 Identities = 8/8 (100%), Positives = 8/8 (100%)

Query:    45 KFLLTAAH 52
             KFLLTAAH
Sbjct:   165 KFLLTAAH 172   MatchSeq
```

```
 Score = 48 (25.8 bits)
 Identities = 8/15 (53%), Positives = 11/15 (73%)

Query:   226 ALLETETPLTFNKNV 240
             AL+E  TP T++ NV
Sbjct:   222 ALIELVTPFTYDQNV 236   MatchSeq
```

```
 Score = 40 (21.8 bits)
 Identities = 6/8 (75%), Positives = 6/8 (75%)

Query:    37 KFLPHPQY 44
             KF PHP Y
Sbjct:   205 KFYPHPAY 212   MatchSeq
```

ButterflyBase SFP11092_1 Total Score: 462

```
                       0      60      120      180      240      300
                       |       |       |        |        |       |301
db|SFP11092_1|SFP1109  | _________________________________________
Local hits (HSPs)      |        _ _          _  _ ____   _
```

```
 Score = 76 (39.8 bits)
 Identities = 10/14 (71%), Positives = 12/14 (85%)

Query:   227 LLETETPLTFNKNV 240
             L+ETE  +TFNKNV
Sbjct:   223 LIETEDQITFNKNV 236
```

```
 Score = 69 (36.3 bits)
 Identities = 8/11 (72%), Positives = 10/11 (90%)

Query:   114 FADTLQTLNLE 124
             FAD +QT+NLE
Sbjct:   266 FADSIQTINLE 276
```

```
 Score = 53 (28.3 bits)
 Identities = 8/18 (44%), Positives = 13/18 (72%)

Query:   341 KYHDLALLDPFTPLTFDK 358
             KYHD+ L++    +TF+K
Sbjct:   217 KYHDIGLIETEDQITFNK 234
```

```
 Score = 52 (27.8 bits)
 Identities = 6/9 (66%), Positives = 7/9 (77%)

Query:    21 RFLPHPEYK 29
             RF+ HP YK
Sbjct:   205 RFIAHPNYK 213
```

```
 Score = 48 (25.8 bits)
 Identities = 7/8 (87%), Positives = 8/8 (100%)

Query:   311 KLSNPPSS 318
             KLS+PPSS
Sbjct:   190 KLSDPPSS 197
```

```
 Score = 47 (25.3 bits)
 Identities = 6/6 (100%), Positives = 6/6 (100%)

Query:   239 NVVMSP 244
             NVVMSP
Sbjct:    64 NVVMSP 69
```

```
 Score = 40 (21.8 bits)
 Identities = 5/7 (71%), Positives = 7/7 (100%)

Query:    46 FLLTAAH 52
             ++LTAAH
Sbjct:   165 YILTAAH 171
```

```
 Score = 39 (21.3 bits)
 Identities = 5/7 (71%), Positives = 7/7 (100%)

Query:    54 RFLLTAA 60
             R++LTAA
Sbjct:   164 RYILTAA 170
```

```
 Score = 38 (20.8 bits)
 Identities = 6/9 (66%), Positives = 7/9 (77%)

Query:    71 ARSDCLEYL 79
             AR  CL+YL
Sbjct:    81 ARDACLQYL 89
```

Ha SG EST hargln|HA-GLN Contig 3250 REV frame 1 Total Score: 68

```
                       0        30        60        90        120      |135
                       |         |         |         |         |
hargln|HA-GLN_Contig_  | _________________________________________
Local hits (HSPs)      |     ___
```

```
 Score = 68 (35.8 bits)
 Identities = 8/9 (88%), Positives = 9/9 (100%)

Query:   337 LTFDKYHDL 345
             LTFDKYHD+
Sbjct:    16 LTFDKYHDI 24
```

Spot 35

m/z	Charge	Peak mW	Peptide mW	Delta (Da)	Delta (ppm)	Ladder Score	Log Likelihood	Start	End	Sequence	Modifications	Subs	Query Tool
525,7633	2	1049,5110	1049,5077	0,0033	3,1215	60,7843	162,6995	1	9	FLQAANACR	Carbamidomethyl C	8	De-novo
525,7633	2	1049,5110	1049,5077	0,0033	3,1215	60,7843	154,8652	1	9	LFQAANACR	Carbamidomethyl C	8	De-novo
525,7633	2	1049,5110	1049,5077	0,0033	3,1215	61,4035	155,0618	1	10	FLGAAANACR	Carbamidomethyl C	9	De-novo
551,3038	2	1100,5920	1100,6077	-0,0157	-14,2867	68,4211	188,4944	1	10	LVTAVNNLEK	Deamidation N	7	De-novo
551,3038	2	1100,5920	1100,6077	-0,0157	-14,2867	68,4211	187,3056	1	10	LVTAVNDLEK			De-novo
551,3038	2	1100,5920	1100,6077	-0,0157	-14,2867	64,9123	185,0965	1	10	VLTAVNNLEK	Deamidation N	7	De-novo
604,8068	2	1207,5980	1207,5941	0,0039	3,2097	66,6667	124,5528	1	11	LLSMQMGGDLK	Oxidation M	6	De-novo
604,8068	2	1207,5980	1207,5941	0,0039	3,2097	66,6667	123,9166	1	11	LLSMQMGGNLK	Oxidation M / Deamidation	6 / 9	De-novo
563,7775	2	1125,5394	1125,5376	0,0018	1,5779	80,7018	206,4214	1	10	MGLTEYNAVK	Deamidation N	7	De-novo
563,7775	2	1125,5394	1125,5376	0,0018	1,5779	70,1754	199,1338	1	10	MGLTEYNVAK	Deamidation N	7	De-novo
493,7385	2	985,4614	985,4617	-0,0003	-0,3288	62,2222	91,7849	1	8	LPFSHNNR	Deamidation N	6 / 7	De-novo
493,7385	2	985,4614	985,4617	-0,0003	-0,3288	62,2222	90,7419	1	8	LPFSHDNR	Deamidation N	7	De-novo
493,7385	2	985,4614	985,4617	-0,0003	-0,3288	62,2222	90,3344	1	8	LPFSHNDR	Deamidation N	6	De-novo
411,2251	2	820,4346	820,433	0,0016	1,9209	64,1026	102,6948	1	7	EVFDALK			De-novo
411,2251	2	820,4346	820,433	0,0016	1,9209	66,6667	101,9175	1	7	VEFDALK			De-novo
411,2251	2	820,4346	820,433	0,0016	1,9209	64,1026	102,0452	1	7	QVFDALK	Deamidation Q	1	De-novo
601,2975	2	1200,5794	1200,5848	-0,0054	-4,5178	46,0317	169,4003	1	11	SAFYPLTGMSK			De-novo
601,2975	2	1200,5794	1200,5848	-0,0054	-4,5178	46,0317	168,7778	1	11	ASFYPLTGMSK			De-novo
601,2975	2	1200,5794	1200,5848	-0,0054	-4,5178	46,0317	168,2454	1	11	TGFYPLTGMSK			De-novo
853,3904	2	1704,7652	1704,7592	0,0060	3,5055	72,043	247,1664	1	16	QHGTEAEGGVYDLSNK	Deamidation Q	1	De-novo
853,3904	2	1704,7652	1704,7592	0,0060	3,5055	69,8925	246,4735	1	16	HQGTEAEGGVYDLSNK	Deamidation Q	2	De-novo
853,3904	2	1704,7652	1704,7592	0,0060	3,5055	72,043	246,1049	1	16	QHGTQAEGGVYDLSNK	Deamidation Q	1 / 5	De-novo
551,8077	2	1101,5998	1101,5665	0,0333	30,2079	52,6316	102,3231	1	10	NVTAVENNLK	Deamidation N	8	De-novo
402,2194	2	802,4232	802,4589	-0,0357	-44,5182	48,7179	73,7394	1	7	PLFDALK			De-novo
561,3301	1	560,3223	560,317	0,0053	9,4375	62,963	81,687	1	5	ATLEK			De-novo
561,3301	1	560,3223	560,317	0,0053	9,4375	62,963	80,0725	1	5	ATLQK	Deamidation Q	4	De-novo
660,3558	2	1318,6960	1318,698	-0,0020	-1,5348	66,6667	290,7882	1	13	VSSTLPTTSGELK			De-novo
660,3558	2	1318,6960	1318,698	-0,0020	-1,5348	74,6667	296,8872	1	13	VSSTLGSLEGELK			De-novo
660,3558	2	1318,6960	1318,698	-0,0020	-1,5348	74,6667	295,3223	1	13	VSSTLGSLEGQLK	Deamidation Q	11	De-novo

NCBI gi|161088178|gb|ABX57148.1| arginine kinase [Archiearis parthenias] Total Score: 822

```
                      0        40       80       120      160
                      |        |        |        |        |        |199
gi|161088178|gb|ABX57  | ______________________________________________
Local hits (HSPs)      | ______    __      __ _______        ______
```

```
Score = 92 (47.8 bits)
Identities = 12/13 (92%), Positives = 13/13 (100%)

Query:  182 TEAEGGVYDLSNK 194
            TEAEGGVYD+SNK
Sbjct:  170 TEAEGGVYDISNK 182   MatchSeq

Score = 79 (41.3 bits)
Identities = 11/14 (78%), Positives = 12/14 (85%)

Query:  298 LKSAFYPLTGMSKQ 311
            LK  FYPLTGMSK+
Sbjct:   17 LKGTFYPLTGMSKE 30   MatchSeq

Score = 78 (40.8 bits)
Identities = 10/11 (90%), Positives = 11/11 (100%)

Query:   59 LLSMQMGGDLK 69
            L+SMQMGGDLK
Sbjct:   84 LISMQMGGDLK 94   MatchSeq

Score = 75 (39.3 bits)
Identities = 12/15 (80%), Positives = 13/15 (86%)

Query:  347 EKVSSTLGSLEGELK 361
            +KVSSTL  LEGELK
Sbjct:    4 DKVSSTLSGLEGELK 18   MatchSeq

Score = 73 (38.3 bits)
Identities = 10/12 (83%), Positives = 11/12 (91%)

Query:  327 LVTAVNDLEKQV 338
            LVTAVND+EK V
Sbjct:  100 LVTAVNDIEKRV 111   MatchSeq

Score = 70 (36.8 bits)
Identities = 9/11 (81%), Positives = 11/11 (100%)

Query:   28 RLVTAVNNLEK 38
            RLVTAVN++EK
Sbjct:   99 RLVTAVNDIEK 109   MatchSeq

Score = 69 (36.3 bits)
Identities = 9/10 (90%), Positives = 10/10 (100%)

Query:   81 MGLTEYNAVK 90
            MGLTEY+AVK
Sbjct:  185 MGLTEYDAVK 194   MatchSeq

Score = 68 (35.8 bits)
Identities = 12/17 (70%), Positives = 13/17 (76%)

Query:  279 KVSSTLGSLEGQLKEVF 295
            KVSSTL  LEG+LK  F
Sbjct:    5 KVSSTLSGLEGELKGTF 21   MatchSeq

Score = 66 (34.8 bits)
Identities = 9/9 (100%), Positives = 9/9 (100%)

Query:    1 FLQAANACR 9
            FLQAANACR
Sbjct:   48 FLQAANACR 56   MatchSeq

Score = 57 (30.3 bits)
Identities = 7/11 (63%), Positives = 8/11 (72%)

Query:  114 DNRLPFSHNDR 124
            + R PFSH DR
Sbjct:  108 EKRVPFSHHDR 118   MatchSeq

Score = 54 (28.8 bits)
Identities = 8/12 (66%), Positives = 9/12 (75%)

Query:  108 RLPFSHDNRLPF 119
            R PFSH +RL F
Sbjct:  110 RVPFSHHDRLGF 121   MatchSeq

Score = 41 (22.3 bits)
Identities = 5/10 (50%), Positives = 8/10 (80%)

Query:  133 EFDALKQVFD 142
            E+DA K+ +D
Sbjct:  189 EYDAVKEMYD 198   MatchSeq
```

Ha SG EST hargln|HA-GLN Contig 1125 FWD frame 1 Total Score: 763

```
                      0        90       180      270      360
                      |        |        |        |        |    |406
hargln|HA-GLN_Contig_  | ______________________________________________
Local hits (HSPs)      | _              ___  __   _____     _ _
```

```
Score = 92 (47.8 bits)
Identities = 12/13 (92%), Positives = 13/13 (100%)

Query:  182 TEAEGGVYDLSNK 194
            TEAEGGVYD+SNK
Sbjct:  302 TEAEGGVYDISNK 314

Score = 81 (42.3 bits)
Identities = 13/15 (86%), Positives = 13/15 (86%)

Query:  347 EKVSSTLGSLEGELK 361
            EKVSSTL  LEGELK
Sbjct:  136 EKVSSTLSGLEGELK 150

Score = 79 (41.3 bits)
Identities = 11/14 (78%), Positives = 12/14 (85%)

Query:  298 LKSAFYPLTGMSKQ 311
            LK  FYPLTGMSK+
Sbjct:  149 LKGTFYPLTGMSKE 162

Score = 78 (40.8 bits)
Identities = 10/11 (90%), Positives = 11/11 (100%)

Query:   59 LLSMQMGGDLK 69
            L+SMQMGGDLK
Sbjct:  216 LISMQMGGDLK 226

Score = 71 (37.3 bits)
Identities = 9/12 (75%), Positives = 11/12 (91%)

Query:  108 RLPFSHDNRLPF 119
            R+PFSHD+RL F
Sbjct:  242 RIPFSHDDRLGF 253

Score = 70 (36.8 bits)
Identities = 9/11 (81%), Positives = 11/11 (100%)

Query:   28 RLVTAVNNLEK 38
            RLVTAVN++EK
Sbjct:  231 RLVTAVNDIEK 241

Score = 69 (36.3 bits)
Identities = 9/10 (90%), Positives = 10/10 (100%)

Query:   81 MGLTEYNAVK 90
            MGLTEY+AVK
Sbjct:  317 MGLTEYDAVK 326

Score = 66 (34.8 bits)
Identities = 9/9 (100%), Positives = 9/9 (100%)

Query:    1 FLQAANACR 9
            FLQAANACR
Sbjct:  180 FLQAANACR 188

Score = 64 (33.8 bits)
Identities = 7/11 (63%), Positives = 10/11 (90%)

Query:  114 DNRLPFSHNDR 124
            + R+PFSH+DR
Sbjct:  240 EKRIPFSHDDR 250

Score = 57 (30.3 bits)
Identities = 8/8 (100%), Positives = 8/8 (100%)

Query:  292 KEVFDALK 299
            KEVFDALK
Sbjct:   19 KEVFDALK 26

Score = 36 (19.8 bits)
Identities = 5/12 (41%), Positives = 8/12 (66%)

Query:  133 EFDALKQVFDAL 144
            E+DA K+  D +
Sbjct:  321 EYDAVKEMHDGI 332
```

Spot 36

m/z	Charge	Peak mW	Peptide mW	Delta (Da)	Delta (ppm)	Ladder Score	Log Likelihood	Start	End	Sequence	Modifications	Subs	Query Tool
494,7851	2	987,5546	987,5601	-0,0055	-5,5936	68,6274	135,1926	1	9	AKLEELASK			De-novo
494,7851	2	987,5546	987,5601	-0,0055	-5,5936	72,549	135,0583	1	9	KALEELASK			De-novo
494,7851	2	987,5546	987,5601	-0,0055	-5,5936	68,6274	133,3513	1	9	AKLEQLASK	Deamidation Q	5	De-novo
493,7379	2	985,4602	985,4617	-0,0015	-1,5465	62,2222	112,7418	1	8	LPFSHDNR	Deamidation N	7	De-novo
493,7379	2	985,4602	985,4617	-0,0015	-1,5465	62,2222	113,3629	1	8	LPFSHNNR	Deamidation N	6 / 7	De-novo
493,7379	2	985,4602	985,4617	-0,0015	-1,5465	62,2222	112,3936	1	8	LPFSHNDR	Deamidation N	6	De-novo
551,3044	2	1100,5932	1100,6077	-0,0145	-13,1963	58,7302	194,7595	1	11	LVTAVGGNLEK	Deamidation N	8	De-novo
551,3044	2	1100,5932	1100,6077	-0,0145	-13,1963	68,4211	192,1036	1	10	LVTAVNNLEK	Deamidation N	7	De-novo
551,3044	2	1100,5932	1100,6077	-0,0145	-13,1963	58,7302	192,2319	1	11	LVTAVGGNLQK	Deamidation N /	8 / 10	De-novo
660,3596	2	1318,7036	1318,698	0,0056	4,2284	58,6667	169,5047	1	13	VSSTLSQLGGELK	Deamidation Q	7	De-novo
660,3596	2	1318,7036	1318,698	0,0056	4,2284	58,6667	168,788	1	13	VSSTLSELGGELK			De-novo
660,3596	2	1318,7036	1318,698	0,0056	4,2284	58,6667	168,1476	1	13	VSSTLSQLGGQLK	Deamidation Q	7 / 11	De-novo
525,7601	2	1049,5046	1049,5077	-0,0031	-2,9766	60,7843	115,665	1	9	FLQAANACR	Carbamidomethyl C	8	De-novo
525,7601	2	1049,5046	1049,5077	-0,0031	-2,9766	61,4035	113,911	1	10	FLGAAANACR	Carbamidomethyl C	9	De-novo
525,7601	2	1049,5046	1049,5077	-0,0031	-2,9766	60,7843	112,1628	1	9	LFQAANACR	Carbamidomethyl C	8	De-novo

NCBI gi|161088190|gb|ABX57154.1| arginine kinase [Macrosoma tipulata] Total Score: 445

```
                      0        40       80       120      160
                      |        |        |        |        |        |199
gi|161088190|gb|ABX57  | ______________________________________________
Local hits (HSPs)      | ___       __        _______     __
```

```
Score = 75 (39.8 bits)
Identities = 10/10 (100%), Positives = 10/10 (100%)

Query:  123 FLQAANACRF 132
            FLQAANACRF
Sbjct:   48 FLQAANACRF 57   MatchSeq

Score = 74 (39.3 bits)
Identities = 9/10 (90%), Positives = 10/10 (100%)

Query:   43 RLPFSHNDRL 52
            R+PFSHNDRL
Sbjct:  110 RIPFSHNDRL 119   MatchSeq

Score = 68 (36.3 bits)
Identities = 13/16 (81%), Positives = 13/16 (81%)

Query:   94 ELKVSSTLSELGGELK 109
            E KVSSTLS L GELK
Sbjct:    3 EEKVSSTLSGLEGELK 18   MatchSeq

Score = 62 (33.2 bits)
Identities = 8/10 (80%), Positives = 10/10 (100%)

Query:   63 LVTAVNNLEK 72
            LVTAVN++EK
Sbjct:  100 LVTAVNDIEK 109   MatchSeq

Score = 57 (30.7 bits)
Identities = 7/11 (63%), Positives = 10/11 (90%)

Query:   28 LPFSHDNRLPF 38
            +PFSH++RL F
Sbjct:  111 IPFSHNDRLGF 121   MatchSeq

Score = 55 (29.7 bits)
Identities = 9/12 (75%), Positives = 10/12 (83%)

Query:   16 ASKAKLEQLASK 27
            A KAKLE+ ASK
Sbjct:  146 ADKAKLEEVASK 157   MatchSeq

Score = 54 (29.2 bits)
Identities = 8/10 (80%), Positives = 8/10 (80%)

Query:  141 RLFQAANACR 150
            R  QAANACR
Sbjct:   47 RFLQAANACR 56   MatchSeq
```

Ha SG EST hargln|HA-GLN Contig 1125 FWD frame 1 Total Score: 450

```
                      0        90       180      270      360
                      |        |        |        |        |    |406
hargln|HA-GLN_Contig_  | ______________________________________________
Local hits (HSPs)      |               __   __    ___   __
```

```
Score = 75 (39.8 bits)
Identities = 10/10 (100%), Positives = 10/10 (100%)

Query:  123 FLQAANACRF 132
            FLQAANACRF
Sbjct:  180 FLQAANACRF 189

Score = 68 (36.3 bits)
Identities = 13/16 (81%), Positives = 13/16 (81%)

Query:   94 ELKVSSTLSELGGELK 109
            E KVSSTLS L GELK
Sbjct:  135 EEKVSSTLSGLEGELK 150

Score = 68 (36.3 bits)
Identities = 8/10 (80%), Positives = 10/10 (100%)

Query:   43 RLPFSHNDRL 52
            R+PFSH+DRL
Sbjct:  242 RIPFSHDDRL 251

Score = 63 (33.7 bits)
Identities = 8/11 (72%), Positives = 10/11 (90%)

Query:   28 LPFSHDNRLPF 38
            +PFSHD+RL F
Sbjct:  243 IPFSHDDRLGF 253

Score = 62 (33.2 bits)
Identities = 8/10 (80%), Positives = 10/10 (100%)

Query:   63 LVTAVNNLEK 72
            LVTAVN++EK
Sbjct:  232 LVTAVNDIEK 241

Score = 60 (32.2 bits)
Identities = 9/12 (75%), Positives = 11/12 (91%)

Query:   16 ASKAKLEQLASK 27
            A KAKLE++ASK
Sbjct:  278 ADKAKLEEIASK 289

Score = 54 (29.2 bits)
Identities = 8/10 (80%), Positives = 8/10 (80%)

Query:  141 RLFQAANACR 150
            R  QAANACR
Sbjct:  179 RFLQAANACR 188
```

Spot 37

m/z	Charge	Peak mW	Peptide mW	Delta (Da)	Delta (ppm)	Ladder Score	Log Likelihood	Start	End	Sequence	Modifications	Subs	Query Tool
727,8416	2	1453,6676	1453,6686	-0,0010	-0,7044	58,6667	335,7334	1	13	ELPVNENDPNGQK	Deamidation N	10	De-novo
727,8416	2	1453,6676	1453,6686	-0,0010	-0,7044	58,6667	335,2361	1	13	LEPVNENDPNGQK	Deamidation N	10	De-novo
727,8416	2	1453,6676	1453,6686	-0,0010	-0,7044	58,6667	334,008	1	13	ELPVNENDPDGQK			De-novo
533,2997	2	1064,5838	1064,5801	0,0037	3,4530	68,4211	184,96	1	10	VGVACLPPPR	Carbamidomethyl C	5	De-novo
533,2997	2	1064,5838	1064,5801	0,0037	3,4530	61,4035	180,9413	1	10	GVVACLPPPR	Carbamidomethyl C	5	De-novo
533,2997	2	1064,5838	1064,5801	0,0037	3,4530	57,8947	179,8466	1	10	VGAVCLPPPR	Carbamidomethyl C	5	De-novo
694,7906	2	1387,5656	1387,5939	-0,0283	-20,4123	65,2174	267,5891	1	12	CGGWANPDQNLR	Carbamidomethyl C /	1 / 10	De-novo
694,7906	2	1387,5656	1387,5939	-0,0283	-20,4123	65,2174	265,9873	1	12	CGGWANPDQDLR	Carbamidomethyl C	1	De-novo
694,7906	2	1387,5656	1387,5939	-0,0283	-20,4123	65,2174	266,8007	1	12	CGGWANPNQDLR	Carbamidomethyl C /	1 / 8	De-novo
534,2623	2	1066,5090	1066,5083	0,0007	0,6338	57,7778	137,9827	1	8	ELYPFQNR	Deamidation N	7	De-novo
534,2623	2	1066,5090	1066,5083	0,0007	0,6338	66,6667	137,4846	1	8	LEYPFQNR	Deamidation N	7	De-novo
534,2623	2	1066,5090	1066,5083	0,0007	0,6338	57,7778	137,2628	1	8	QLYPFQNR	Deamidation Q /	1 / 7	De-novo
562,3112	2	1122,6068	1122,6073	-0,0005	-0,4668	58,8235	92,4469	1	9	LFTPLQTFR	Deamidation Q	6	De-novo
562,3112	2	1122,6068	1122,6185	-0,0117	-10,4434	47,0588	93,257	1	9	LFTPKNTFR			De-novo
562,3112	2	1122,6068	1122,6073	-0,0005	-0,4668	58,8235	92,1811	1	9	LFTPQTLFR	Deamidation Q	5	De-novo
727,3815	2	1452,7474	1452,7476	-0,0002	-0,1542	52,1739	142,8551	1	12	FGEFPWMVALLK	Oxidation M	7	De-novo
727,3815	2	1452,7474	1452,7476	-0,0002	-0,1542	52,1739	138,7204	1	12	FGQFPWMVALLK	Deamidation Q / Oxidation	3 / 7	De-novo
727,3815	2	1452,7474	1452,7476	-0,0002	-0,1542	46,3768	136,4656	1	12	GFEFPWMVALLK	Oxidation M	7	De-novo
804,857	2	1607,6984	1607,7139	-0,0155	-9,6559	68,9655	404,9265	1	15	DGGGSPLSCPLEYEK	Carbamidomethyl C	9	De-novo
804,857	2	1607,6984	1607,7139	-0,0155	-9,6559	68,9655	403,9823	1	15	DGGGSPLSCPLEYQK	Carbamidomethyl C /	9 / 14	De-novo
804,857	2	1607,6984	1607,7139	-0,0155	-9,6559	68,9655	404,2138	1	15	DGGGSPLSCPLQYEK	Carbamidomethyl C /	9 / 12	De-novo
737,3718	2	1472,7280	1472,726	0,0020	1,3417	69,3333	125,7533	1	13	NVASVLTHQDFNK	Deamidation N	1	De-novo
737,3718	2	1472,7280	1472,726	0,0020	1,3417	69,3333	125,5502	1	13	NVASVLTHQNFNK	Deamidation N	1 / 10	De-novo
737,3718	2	1472,7280	1472,726	0,0020	1,3417	66,6667	125,8207	1	13	VDASVLTHQNFNK	Deamidation N	10	De-novo
746,394	2	1490,7724	1490,7729	-0,0005	-0,3515	45,3333	88,4326	1	13	TLQYGLEGLNDLR			De-novo
746,394	2	1490,7724	1490,7729	-0,0005	-0,3515	42,6667	87,7955	1	13	TLQYGLEGLDNLR			De-novo
702,8017	2	1403,5878	1403,5922	-0,0044	-3,1519	68	252,1553	1	13	CGGCVANPDQNLR	Carbamidomethyl C /	1 / 11	De-novo
702,8017	2	1403,5878	1403,5922	-0,0044	-3,1519	68	250,1644	1	13	CGGMAANPDQNLR	Carbamidomethyl C /	1 / 11	De-novo
702,8017	2	1403,5878	1403,5922	-0,0044	-3,1519	68	248,9869	1	13	CGGMAANPDQDLR	Carbamidomethyl C	1	De-novo

NCBI gi|208972549|gb|ACI32835.1| serine proteinase-like protein 1 [Helicoverpa armigera] Total Score: 725

```
                       0        90       180       270       360
                       |         |         |         |         |      |428
gi|208972549|gb|ACI32  | _____________________________________________
Local hits (HSPs)      |              _ _____    __    __        _
```

```
 Score = 102 (52.7 bits)
 Identities = 14/17 (82%), Positives = 15/17 (88%)

Query:   192 KDGGGSPLSCPLEYEKD 208
             K  GGSPLSCP+EYEKD
Sbjct:   363 KGDGGSPLSCPIEYEKD 379   MatchSeq

 Score = 98 (50.7 bits)
 Identities = 12/14 (85%), Positives = 14/14 (100%)

Query:    13 KLEPVNENDPNGQK 26
             K+EPVNENDP+GQK
Sbjct:   182 KIEPVNENDPDGQK 195   MatchSeq

 Score = 94 (48.7 bits)
 Identities = 11/13 (84%), Positives = 13/13 (100%)

Query:   168 KFGQFPWMVALLK 180
             KFG+FPWMVA+LK
Sbjct:   170 KFGEFPWMVAILK 182   MatchSeq

 Score = 91 (47.2 bits)
 Identities = 11/15 (73%), Positives = 14/15 (93%)

Query:   236 EKNVASVLTHQDFNK 250
             + +VASV+THQDFNK
Sbjct:   244 DRDVASVITHQDFNK 258   MatchSeq

 Score = 88 (45.7 bits)
 Identities = 12/14 (85%), Positives = 12/14 (85%)

Query:    29 PVNENDPDGQKVGV 42
             PVNENDPDGQK  V
Sbjct:   185 PVNENDPDGQKLNV 198   MatchSeq

 Score = 73 (38.2 bits)
 Identities = 10/10 (100%), Positives = 10/10 (100%)

Query:    40 VGVACLPPPR 49
             VGVACLPPPR
Sbjct:   280 VGVACLPPPR 289   MatchSeq

 Score = 67 (35.2 bits)
 Identities = 9/9 (100%), Positives = 9/9 (100%)

Query:    52 VACLPPPRV 60
             VACLPPPRV
Sbjct:   282 VACLPPPRV 290   MatchSeq

 Score = 63 (33.2 bits)
 Identities = 7/12 (58%), Positives = 10/12 (83%)

Query:   102 QDLRELYPFQNR 113
             Q+  E+YPFQ+R
Sbjct:   234 QNTKEIYPFQDR 245   MatchSeq

 Score = 49 (26.2 bits)
 Identities = 6/6 (100%), Positives = 6/6 (100%)

Query:    72 GWANPD 77
             GWANPD
Sbjct:   150 GWANPD 155   MatchSeq
```

HaEST hargln|HA-GLN_Contig_4443_FWD_frame_0 Total Score: 143

```
                       0       40       80      120      160
                       |         |         |         |         |      |192
hargln|HA-GLN_Contig_  | _____________________________________________
Local hits (HSPs)      |                                      __   ___
```

```
 Score = 94 (48.7 bits)
 Identities = 11/13 (84%), Positives = 13/13 (100%)

Query:   168 KFGQFPWMVALLK 180
             KFG+FPWMVA+LK
Sbjct:   179 KFGEFPWMVAILK 191

 Score = 49 (26.2 bits)
 Identities = 6/6 (100%), Positives = 6/6 (100%)

Query:    72 GWANPD 77
             GWANPD
Sbjct:   159 GWANPD 164
```

Spot 38

m/z	Charge	Peak mW	Peptide mW	Delta (Da)	Delta (ppm)	Ladder Score	Log Likelihood	Start	End	Sequence	Modifications	Subs	Query Tool
464,2164	2	926,4172	926,4168	0,0004	0,4059	76,9231	158,4535	1	7	MYLAEQR	Oxidation M / Deamidation	1 / 6	De-novo
464,2164	2	926,4172	926,4168	0,0004	0,4059	76,9231	158,496	1	7	MYLAEER	Oxidation M	1	De-novo
464,2164	2	926,4172	926,4168	0,0004	0,4059	76,9231	157,9142	1	7	MYLAQER	Oxidation M / Deamidation	1 / 5	De-novo
514,7397	2	1027,4638	1027,4611	0,0027	2,6045	70,5882	113,4382	1	9	TSNGFPDYK			De-novo
514,7397	2	1027,4638	1027,4611	0,0027	2,6045	70,5882	112,3705	1	9	TSNGFPNYK	Deamidation N	7	De-novo
514,7397	2	1027,4638	1027,4611	0,0027	2,6045	82,3529	110,0901	1	9	TNSGFPDYK			De-novo
874,9247	2	1747,8338	1747,9834	-0,1496	-85,5981	37,3737	129,4092	1	17	NLPVDGLAPNLNLTGLK			De-novo
874,9247	2	1747,8338	1747,9834	-0,1496	-85,5981	35,3535	129,3008	1	17	LNPVDGLAPNLNLTGLK			De-novo
874,9247	2	1747,8338	1747,9834	-0,1496	-85,5981	37,3737	129,4815	1	17	NLPVNGLAPNLNLTGLK	Deamidation N	5	De-novo
631,861	2	1261,7064	1261,703	0,0034	2,6757	65,2174	257,577	1	12	LGPQPLPQAVDK			De-novo
631,861	2	1261,7064	1261,703	0,0034	2,6757	65,2174	257,1309	1	12	GLPQPLPQAVDK			De-novo
631,861	2	1261,7064	1261,703	0,0034	2,6757	65,2174	257,5929	1	12	AVPQPLPQAVDK			De-novo
731,3761	2	1460,7366	1460,7358	0,0008	0,5312	70,1149	288,7957	1	15	SLVAGDVADATDLSK			De-novo
731,3761	2	1460,7366	1460,7358	0,0008	0,5312	70,1149	287,2219	1	15	LSVAGDVADATDLSK			De-novo
731,3761	2	1460,7366	1460,7358	0,0008	0,5312	70,1149	289,7033	1	15	SLVAGNVADATDLSK	Deamidation N	6	De-novo
641,3572	2	1280,6988	1280,6976	0,0012	0,9182	62,3188	96,2369	1	12	LDDQPSLPVGLK			De-novo
641,3572	2	1280,6988	1280,6976	0,0012	0,9182	62,3188	95,6134	1	12	LNDQPSLPVGLK	Deamidation N	2	De-novo
641,3572	2	1280,6988	1280,6976	0,0012	0,9182	62,3188	95,1621	1	12	LDNQPSLPVGLK	Deamidation N	3	De-novo
454,7361	2	907,4566	907,4553	0,0013	1,4061	43,5897	106,1501	1	7	TVAEWFR			De-novo
728,892	2	1455,7684	1455,7643	0,0041	2,7999	78,6667	224,3826	1	13	MELSLGEPLNPLK	Oxidation M	1	De-novo
728,892	2	1455,7684	1455,7643	0,0041	2,7999	78,6667	222,72	1	13	MQLSLGEPLNPLK	Oxidation M / Deamidation	1 / 2	De-novo
728,892	2	1455,7684	1455,7643	0,0041	2,7999	70,6667	221,1584	1	13	QMLSLGEPLNPLK	Deamidation Q / Oxidation	1 / 2	De-novo
883,8945	2	1765,7734	1765,816	-0,0426	-24,1384	61,2903	97,5465	1	16	LFNESPSELSGWGNTK	Deamidation N	3	De-novo
883,8945	2	1765,7734	1765,8159	-0,0425	-24,0818	51,6129	98,2206	1	16	LFNESPDTLSGWGNTK	Deamidation N	3	De-novo
883,8945	2	1765,7734	1765,816	-0,0426	-24,1384	59,1398	97,0146	1	16	FLNESPSELSGWGNTK	Deamidation N	3	De-novo
771,3765	2	1540,7374	1540,7368	0,0006	0,3738	67,8161	373,4292	1	15	GNGGNPLDVLELGQR	Deamidation N /	2 / 5 / 14	De-novo
771,3765	2	1540,7374	1540,7368	0,0006	0,3738	67,8161	372,589	1	15	GNGGDPLDVLELGQR	Deamidation N /	2 / 14	De-novo
771,3765	2	1540,7374	1540,7368	0,0006	0,3738	67,8161	372,5818	1	15	NGGGNPLDVLELGQR	Deamidation N /	1 / 5 / 14	De-novo
897,9104	2	1793,8052	1793,8254	-0,0202	-11,2742	31,0345	57,9744	1	15	SCNEQTALLSKDNWK	Carbamidomethyl C /	2 / 3	De-novo
897,9104	2	1793,8052	1793,8253	-0,0201	-11,2185	34,4086	57,486	1	16	SCNEQTALLSVSGNWK	Carbamidomethyl C /	2 / 3	De-novo
611,3775	2	1220,7394	1220,738	0,0014	1,1272	44,4444	103,0691	1	11	LPDALLLPELK			De-novo
611,3775	2	1220,7394	1220,7379	0,0015	1,2091	41,2698	100,8141	1	11	PLDALLLPELK			De-novo
491,2783	2	980,5410	980,5079	0,0331	33,7335	54,902	38,1248	1	9	VPNWAQGLP			De-novo
491,2783	2	980,5410	980,5079	0,0331	33,7335	60,7843	35,1611	1	9	VPNWAQGLP			De-novo
421,7582	2	841,5008	841,5021	-0,0013	-1,5734	64,4444	94,4876	1	8	VATVSLPR			De-novo
538,772	2	1075,5284	1075,5259	0,0025	2,3021	77,7778	236,9174	1	11	TNVSVEGSAGR			De-novo
538,772	2	1075,5284	1075,5259	0,0025	2,3021	77,193	233,4116	1	10	TNVSVEGSQR			De-novo
586,329	2	1170,6424	1170,6431	-0,0007	-0,6185	59,6491	64,3672	1	10	LVCTVVEVPR	Carbamidomethyl C	3	De-novo
586,329	2	1170,6424	1170,6431	-0,0007	-0,6185	61,4035	60,7963	1	10	LVCTVVEPVR	Carbamidomethyl C	3	De-novo

NCBI gi|15862142|emb|CAC88582.1| unnamed protein product [Heliothis virescens] Total Score: 562

```
                       0       60      120      180      240
                       |         |         |         |         |      |288
gi|15862142|emb|CAC88  | _____________________________________________
Local hits (HSPs)      | _   __ ____     ____       _     _____   _
```

```
 Score = 100 (51.5 bits)
 Identities = 15/18 (83%), Positives = 16/18 (88%)

Query:   136 SLVAGDVADATDLSKLSV 153
             SLVAGDVADATD+SK  V
Sbjct:   228 SLVAGDVADATDISKTNV 245   MatchSeq

 Score = 98 (50.5 bits)
 Identities = 12/15 (80%), Positives = 15/15 (100%)

Query:   236 KMQLSLGEPLNPLKQ 250
             KM++SLGEPLNP+KQ
Sbjct:    56 KMEISLGEPLNPIKQ 70   MatchSeq

 Score = 78 (40.6 bits)
 Identities = 10/12 (83%), Positives = 11/12 (91%)

Query:   112 GLPQPLPQAVDK 123
             GLPQP+PQ VDK
Sbjct:   270 GLPQPIPQSVDK 281   MatchSeq

 Score = 70 (36.6 bits)
 Identities = 10/14 (71%), Positives = 11/14 (78%)

Query:   447 LPRTNVSVEGSAGR 460
             +  TNVSVEGS GR
Sbjct:   240 ISKTNVSVEGSVGR 253   MatchSeq

 Score = 69 (36.1 bits)
 Identities = 7/11 (63%), Positives = 11/11 (100%)

Query:   330 DPLDVLELGQR 340
             DP+DV+E+G+R
Sbjct:   117 DPIDVIEIGER 127   MatchSeq

 Score = 56 (29.6 bits)
 Identities = 6/7 (85%), Positives = 7/7 (100%)

Query:     8 MYLAEER 14
             MY+AEER
Sbjct:     1 MYIAEER 7   MatchSeq

 Score = 53 (28.1 bits)
 Identities = 10/13 (76%), Positives = 10/13 (76%)

Query:   468 SQRLVCTVVEVPR 480
             S RLV  VVEVPR
Sbjct:    39 SKRLVNMVVEVPR 51   MatchSeq

 Score = 38 (20.7 bits)
 Identities = 4/4 (100%), Positives = 4/4 (100%)

Query:   220 EWFR 223
             EWFR
Sbjct:   187 EWFR 190   MatchSeq
```

Ha SG EST None

Ha EST other harmi_rn|HA-RN-ALL_Contig_2487_FWD_frame_0 Total Score: 221

```
                       0       60      120      180      240
                       |         |         |         |         |  |259
harmi_rn|HA-RN-ALL_Co  | ___________________________________________
Local hits (HSPs)      |       ______  __
```

```
 Score = 71 (37.1 bits)
 Identities = 8/14 (57%), Positives = 12/14 (85%)

Query:   411 LKPLDALLLPELKV 424
             +K +D L++PELKV
Sbjct:    65 IKAIDPLIIPELKV 78

 Score = 69 (36.1 bits)
 Identities = 9/11 (81%), Positives = 9/11 (81%)

Query:    20 ERTSNGFPDYK 30
             E TSNGF DYK
Sbjct:    54 EKTSNGFXDYK 64

 Score = 43 (23.2 bits)
 Identities = 5/8 (62%), Positives = 7/8 (87%)

Query:    58 NLNLTGLK 65
             N+ +TGLK
Sbjct:    92 NIXITGLK 99

 Score = 38 (20.7 bits)
 Identities = 5/10 (50%), Positives = 8/10 (80%)

Query:    46 DYKNLPVDGL 55
             D+KN+ + GL
Sbjct:    89 DFKNIXITGL 98
```

Spot 39

m/z	Charge	Peak mW	Peptide mW	Delta (Da)	Delta (ppm)	Ladder Score	Log Likelihood	Start	End	Sequence	Modifications	Subs	Query Tool
645,862	2	1289,7084	1289,7092	-0,0008	-0,6389	52,1739	95,5397	1	12	SPAVPPKRDLNP			De-novo
645,862	2	1289,7084	1289,7092	-0,0008	-0,6389	52,1739	95,7628	1	12	SPAVPPKRNLNP	Deamidation N	9	De-novo
645,862	2	1289,7084	1289,7092	-0,0008	-0,6389	52,1739	93,3199	1	12	SPAVPPKRDNLP			De-novo
972,401	2	1942,7864	1942,7964	-0,0100	-5,1596	60,6838	387,6316	1	20	FNSACGNGDSGSGLVNETGR	Deamidation N	2 / 16	De-novo
972,401	2	1942,7864	1942,7964	-0,0100	-5,1596	55,2846	386,0894	1	21	FNSACGGGGDSGSGLVNETGR	Deamidation N	2 / 17	De-novo
972,401	2	1942,7864	1942,8141	-0,0277	-14,2700	59,8291	382,3551	1	20	FNSSSGNGDSGSGLVNETGR	Deamidation N	2 / 16	De-novo
590,3063	2	1178,5970	1178,5931	0,0039	3,2887	54,386	64,4892	1	10	YEELVSLGNR			De-novo
648,8251	2	1295,6346	1295,6292	0,0054	4,1493	60,3175	111,2468	1	11	CSSNQLVVFSR	Carbamidomethyl C	1	De-novo
648,8251	2	1295,6346	1295,6292	0,0054	4,1493	60,3175	111,3025	1	11	SCSNQLVVFSR	Carbamidomethyl C	2	De-novo
648,8251	2	1295,6346	1295,6292	0,0054	4,1493	53,6232	112,1746	1	12	GSCSNQLVVFSR			De-novo
883,8961	2	1765,7766	1765,7756	0,0010	0,5527	50,4762	126,3722	1	18	SGTDWSAQALSGGEGNTK	Deamidation Q	8	De-novo
883,8961	2	1765,7766	1765,7756	0,0010	0,5527	48,5714	126,0791	1	18	GSTDWSAQALSGGEGNTK	Deamidation Q	8	De-novo
883,8961	2	1765,7766	1765,7756	0,0010	0,5527	50,4762	124,5674	1	18	SGTDWSAEALSGGEGNTK			De-novo
625,8057	2	1249,5958	1249,5979	-0,0021	-1,6997	71,9298	114,8985	1	10	WLNADEFNLK	Deamidation N	3	De-novo
611,3742	2	1220,7328	1220,738	-0,0052	-4,2794	49,2063	66,975	1	11	LADPLLLPELK			De-novo
611,3742	2	1220,7328	1220,738	-0,0052	-4,2794	57,1429	65,3346	1	11	LPEGLLLEPLK			De-novo
611,3742	2	1220,7328	1220,7379	-0,0051	-4,1975	47,619	65,9418	1	11	ALDPLLLPELK			De-novo
737,9227	2	1473,8298	1473,8303	-0,0005	-0,3555	49,3827	86,1177	1	14	APSLAVREPVNPPK			De-novo
737,9227	2	1473,8298	1473,8303	-0,0005	-0,3555	51,7241	86,6635	1	15	APSLAVREPVGGPPK			De-novo
1366,1223	2	2730,2290	2730,2117	0,0173	6,3277	37,1069	196,8403	1	27	PSNDGAGTGGYLSHPVDNTENESGVTR			De-novo
1366,1223	2	2730,2290	2730,2117	0,0173	6,3277	37,1069	196,6239	1	27	PSNDGAGTGGYLHSPVDNTENESGVTR			De-novo
1366,1223	2	2730,2290	2730,2117	0,0173	6,3277	37,1069	197,4086	1	27	PSNDGAGTGGYLSHPVDNTENQSGVTR	Deamidation Q	22	De-novo
492,2836	3	1473,8274	1473,8302	-0,0028	-1,9242	46,9136	182,0672	1	14	APSLAVPPKPVNER			De-novo
492,2836	3	1473,8274	1473,7939	0,0335	22,7060	54,321	183,6546	1	14	APSSPVPPKPVNER			De-novo
492,2836	3	1473,8274	1473,8302	-0,0028	-1,9242	46,9136	181,637	1	14	APSLAVPPKPVNQR	Deamidation Q	13	De-novo
698,8475	2	1395,6794	1395,6783	0,0011	0,7710	56	127,4177	1	13	LVGVASNLGENWH	Deamidation N	7	De-novo
698,8475	2	1395,6794	1395,6783	0,0011	0,7710	56	126,2632	1	13	VLGVASNLGENWH	Deamidation N	7	De-novo
698,8475	2	1395,6794	1395,6783	0,0011	0,7710	56	125,8384	1	13	LVGVASNLGQNWH	Deamidation N /	7 / 10	De-novo

NCBI gi|261277877|sp|C1JE15.1|SCLXB_HELVI RecName: Full=Scolexin B; Flags: [Heliothis virescens] Total Score: 306

```
                     0     60     120    180    240
                     |      |      |      |      |     |288
gi|261277877|sp|C1JE1 |_______________________________________
Local hits (HSPs)     |   ____        _           ____ _
```

```
Score = 89 (46.4 bits)
Identities = 13/14 (92%), Positives = 14/14 (100%)

Query:   43 NGDSGSGLVNETGR 56
            NGDSGSGLV+ETGR
Sbjct:  236 NGDSGSGLVDETGR 249   MatchSeq
```

```
Score = 73 (38.4 bits)
Identities = 11/15 (73%), Positives = 12/15 (80%)

Query:  490 KAPSLAVPPKPVNER 504
            KA   A+PPKPVNER
Sbjct:   47 KASAPAIPPKPVNER 61   MatchSeq
```

```
Score = 65 (34.4 bits)
Identities = 7/10 (70%), Positives = 10/10 (100%)

Query:  196 WLNADEFNLK 205
            WL+AD+FN+K
Sbjct:  129 WLDADDFNIK 138   MatchSeq
```

```
Score = 42 (22.9 bits)
Identities = 6/6 (100%), Positives = 6/6 (100%)

Query:  113 LVVFSR 118
            LVVFSR
Sbjct:  269 LVVFSR 274   MatchSeq
```

```
Score = 37 (20.4 bits)
Identities = 5/6 (83%), Positives = 6/6 (100%)

Query:  404 VLGVAS 409
            V+GVAS
Sbjct:  250 VIGVAS 255   MatchSeq
```

Ha SG EST None

Ha EST other harmi_rn|HA-RN-ALL_Contig 5254 FWD frame 2 Total Score: 417

```
                     0      60     120    180    240
                     |      |      |      |      |  |260
harmi_rn|HA-RN-ALL_Co |_______________________________________
Local hits (HSPs)     |     ___  ___  __          ____
```

```
Score = 89 (46.4 bits)
Identities = 13/14 (92%), Positives = 14/14 (100%)

Query:   43 NGDSGSGLVNETGR 56
            NGDSGSGLV+ETGR
Sbjct:  239 NGDSGSGLVDETGR 252
```

```
Score = 67 (35.4 bits)
Identities = 9/10 (90%), Positives = 9/10 (90%)

Query:  353 AVPPKPVNER 362
            AVPPK VNER
Sbjct:   55 AVPPKXVNER 64
```

```
Score = 63 (33.4 bits)
Identities = 8/10 (80%), Positives = 9/10 (90%)

Query:  196 WLNADEFNLK 205
            WL+A EFNLK
Sbjct:  132 WLDAXEFNLK 141
```

```
Score = 63 (33.4 bits)
Identities = 9/17 (52%), Positives = 10/17 (58%)

Query:  278 YLSHPVDNTENESGVTR 294
            Y+     N ENESGV R
Sbjct:   99 YILXXTXNXENESGVXR 115
```

```
Score = 50 (26.9 bits)
Identities = 7/7 (100%), Positives = 7/7 (100%)

Query:    1 SPAVPPK 7
            SPAVPPK
Sbjct:   53 SPAVPPK 59
```

```
Score = 48 (25.9 bits)
Identities = 8/13 (61%), Positives = 9/13 (69%)

Query:   56 RFNSACGGGGDSG 68
            RF+SAC G   SG
Sbjct:  233 RFDSACNGDSGSG 245
```

```
Score = 37 (20.4 bits)
Identities = 5/6 (83%), Positives = 6/6 (100%)

Query:  404 VLGVAS 409
            V+GVAS
Sbjct:  253 VIGVAS 258
```

Spot 40

m/z	Charge	Peak mW	Peptide mW	Delta (Da)	Delta (ppm)	Ladder Score	Log Likelihood	Start	End	Sequence	Modifications	Subs	Query Tool
665,8038	2	1329,5920	1329,5909	0,0011	0,8093	39,1304	72,1755	1	12	QNSATGQPNEER			De-novo
665,8038	2	1329,5920	1329,5908	0,0012	0,8845	42,6667	74,8916	1	13	GGQSATGPNEEQR			De-novo
665,8038	2	1329,5920	1329,5909	0,0011	0,8093	52,1739	73,14	1	12	QNSATGPNQEER			De-novo
508,2263	2	1014,4370	1014,4692	-0,0322	-31,7644	62,7451	85,655	1	9	SVEAAYMTK	Oxidation M	7	De-novo
508,2263	2	1014,4370	1014,4692	-0,0322	-31,7644	62,7451	85,941	1	9	SVQAAYMTK	Deamidation Q / Oxidation	3 / 7	De-novo
618,268	2	1234,5204	1234,5215	-0,0011	-0,9105	48	124,7256	1	13	FSSSSGNATGSHP			De-novo
618,268	2	1234,5204	1234,5215	-0,0011	-0,9105	50,6667	123,4797	1	13	FSSSSGNAASSHP			De-novo
618,268	2	1234,5204	1234,5215	-0,0011	-0,9105	42,6667	123,4525	1	13	FSSSSGNATSGHP			De-novo
515,2193	2	1028,4230	1028,4451	-0,0221	-21,5121	72,549	225,0313	1	9	TSNGFPDYK	Deamidation N	3	De-novo
515,2193	2	1028,4230	1028,4451	-0,0221	-21,5121	72,549	222,0406	1	9	TSDGFPDYK			De-novo
874,9144	2	1747,8132	1748,0311	-0,2179	-124,6683	43,4343	227,3372	1	17	LNTVAGGLKHLNLTGLK			De-novo
884,9122	2	1767,8088	1767,8064	0,0024	1,3440	56,1905	268,3069	1	18	GYSPGALGSSQPSGNNFK	Deamidation N	16	De-novo
884,9122	2	1767,8088	1767,8064	0,0024	1,3440	56,1905	267,1617	1	18	GYSPGALGSSQPSGNDFK			De-novo
884,9122	2	1767,8088	1767,8064	0,0024	1,3440	56,7568	268,3836	1	19	GYSPGALGSSAGPSGNDFK			De-novo
679,3305	2	1356,6454	1356,6885	-0,0431	-31,7862	81,4815	219,0026	1	14	LNDLENALGAAGAK	Deamidation N	6	De-novo
679,3305	2	1356,6454	1356,6885	-0,0431	-31,7862	81,3333	217,385	1	13	LNDLENALGAQAK	Deamidation N	6	De-novo
514,3051	2	1026,5946	1026,6073	-0,0127	-12,3942	68,4211	172,1905	1	10	VALNLVGNVK	Deamidation N	8	De-novo
437,7486	2	873,4816	873,4959	-0,0143	-16,3985	48,8889	121,4371	1	8	LANALFPK	Deamidation N	3	De-novo
437,7486	2	873,4816	873,496	-0,0144	-16,5130	48,8889	120,9105	1	8	LADALFPK			De-novo
437,7486	2	873,4816	873,4959	-0,0143	-16,3985	62,2222	114,4737	1	8	LAANLFPK	Deamidation N	4	De-novo
611,3599	2	1220,7042	1220,7379	-0,0337	-27,6259	49,2063	258,4876	1	11	NPLLALLPELK	Deamidation N	1	De-novo
651,8642	2	1301,7128	1301,7078	0,0050	3,8227	65,2174	174,3211	1	12	SLDLDLSLAEVK			De-novo
651,8642	2	1301,7128	1301,7078	0,0050	3,8227	73,913	173,4953	1	12	SLDLDSLLAEVK			De-novo
651,8642	2	1301,7128	1301,7078	0,0050	3,8227	65,2174	172,1709	1	12	SLDLDLSLAQVK	Deamidation Q	10	De-novo
433,2187	2	864,4218	864,4341	-0,0123	-14,2567	48,8889	115,4407	1	8	NAALSDFK			De-novo
665,3233	2	1328,6310	1328,6321	-0,0011	-0,8460	53,6232	88,6857	1	12	NVQDALANQAQR	Deamidation N /	8 / 9	De-novo
665,3233	2	1328,6310	1328,6321	-0,0011	-0,8460	60,8696	88,5399	1	12	VNQDALANQAQR	Deamidation N /	8 / 9	De-novo
665,3233	2	1328,6310	1328,6321	-0,0011	-0,8460	53,6232	87,8553	1	12	NVQDALADQAQR	Deamidation Q	9	De-novo
801,8792	2	1601,7428	1601,8812	-0,1384	-86,4134	44,086	277,5359	1	16	LNTGLGGLTMNLTGLK			De-novo
605,2825	2	1208,5494	1208,5496	-0,0002	-0,1853	63,4921	210,1965	1	11	TGGQLFNPCSK	Deamidation Q /	4 / 9	De-novo
605,2825	2	1208,5494	1208,5496	-0,0002	-0,1853	63,4921	208,5071	1	11	TGGELFNPCSK	Carbamidomethyl C	9	De-novo
605,2825	2	1208,5494	1208,5496	-0,0002	-0,1853	55,0725	209,6682	1	12	TGGQLFNPGCSK	Deamidation Q	4	De-novo
662,8101	2	1323,6046	1323,6017	0,0029	2,1729	65,2174	271,746	1	12	DPMGATESFLEK			De-novo
662,8101	2	1323,6046	1323,6017	0,0029	2,1729	73,913	269,1202	1	12	MPDGATESFLEK			De-novo
662,8101	2	1323,6046	1323,6017	0,0029	2,1729	65,2174	270,7243	1	12	DPMGATQSFLEK	Deamidation Q	7	De-novo
583,2938	2	1164,5720	1164,5775	-0,0055	-4,7434	74,5098	86,8739	1	9	LENELQTYR			De-novo
583,2938	2	1164,5720	1164,5775	-0,0055	-4,7434	71,9298	85,5816	1	10	LENELGATYR			De-novo
897,9142	2	1793,8128	1793,8256	-0,0128	-7,1490	40,9524	160,1921	1	18	AASNECAGSLLSGWGNKK	Deamidation N	4 / 16	De-novo
897,9142	2	1793,8128	1793,8256	-0,0128	-7,1490	40	160,3173	1	18	GANTECAGSLLSGWGNKK	Deamidation N	3 / 16	De-novo
897,9142	2	1793,8128	1793,8256	-0,0128	-7,1490	42,4242	153,466	1	17	AASECADSLLSGWGNKK	Carbamidomethyl C /	5 / 15	De-novo

NCBI gi|194176442|gb|EDW90053.1| GE13058 [Drosophila yakuba] Total Score: 113

```
                     0     120    240    360    480
                     |      |      |      |      |     |576
gi|194176442|gb|EDW90 |_______________________________________
Local hits (HSPs)     |                    ____   _
```

```
Score = 80 (42.2 bits)
Identities = 20/41 (48%), Positives = 21/41 (51%)

Query:   55 KFSSSSGNATGSHPFSSSSGNAASSHPFSSSSGNATSGHPT 95
            K SSSS      S    SSSS     SS     SSSSG     S +PT
Sbjct:  345 KSSSSSHSSNSSNSSSSSSHSSSSSNSISSSSGSGSSDQPT 385   MatchSeq
```

```
Score = 33 (18.5 bits)
Identities = 6/9 (66%), Positives = 6/9 (66%)

Query:  510 SSSGNATGS 518
            SSS N  GS
Sbjct:  435 SSSSNTNGS 443   MatchSeq
```

ButterflyBase SFP04769_1 Total Score: 273

```
                     0      50     100    150    200
                     |      |      |      |      |     |244
butterfly|SFP04769_1  |_______________________________________
Local hits (HSPs)     |   ____ _  __   __
```

```
Score = 56 (30.0 bits)
Identities = 8/8 (100%), Positives = 8/8 (100%)

Query:  391 ATESFLEK 398
            ATESFLEK
Sbjct:   38 ATESFLEK 45
```

```
Score = 55 (29.5 bits)
Identities = 7/12 (58%), Positives = 10/12 (83%)

Query:  211 KVALNLVGNVKL 222
            K  LN+VG+VK+
Sbjct:  110 KAVLNIVGDVKI 121
```

```
Score = 45 (24.5 bits)
Identities = 6/9 (66%), Positives = 7/9 (77%)

Query:  121 KHLNLTGLK 129
            K +N TGLK
Sbjct:   81 KNINVTGLK 89
```

```
Score = 44 (24.0 bits)
Identities = 6/11 (54%), Positives = 8/11 (72%)

Query:  103 KTSDGFPDYKL 113
            KT +G PDY +
Sbjct:   45 KTCNGIPDYDI 55
```

```
Score = 38 (21.0 bits)
Identities = 4/6 (66%), Positives = 6/6 (100%)

Query:  251 LLPELK 256
            ++PELK
Sbjct:   62 IIPELK 67
```

```
Score = 35 (19.5 bits)
Identities = 5/8 (62%), Positives = 6/8 (75%)

Query:  292 KNAALSDF 299
            KN  +SDF
Sbjct:   89 KNQQISDF 96
```

Ha SG EST None

Ha EST other harmi_rn|HA-RN-ALL_Contig 2487 FWD frame 0 Total Score: 397

```
                     0      60     120    180    240
                     |      |      |      |      |  |259
harmi_rn|HA-RN-ALL_Co |_______________________________________
Local hits (HSPs)     |   __ ____ __  __  ___   _
```

```
Score = 67 (35.6 bits)
Identities = 8/11 (72%), Positives = 10/11 (90%)

Query:  103 KTSDGFPDYKL 113
            KTS+GF DYK+
Sbjct:   55 KTSNGFXDYKI 65
```

```
Score = 64 (34.1 bits)
Identities = 10/13 (76%), Positives = 11/13 (84%)

Query:  390 GATESFLEKMPDG 402
            GATESFLEK  +G
Sbjct:   47 GATESFLEKTSNG 59
```

```
Score = 60 (32.1 bits)
Identities = 9/10 (90%), Positives = 9/10 (90%)

Query:  354 GGQLFNPCSK 363
            GG LFNPCSK
Sbjct:   30 GGTLFNPCSK 39
```

```
Score = 55 (29.6 bits)
Identities = 7/12 (58%), Positives = 10/12 (83%)

Query:  211 KVALNLVGNVKL 222
            K  LN+VG+VK+
Sbjct:  120 KAVLNIVGDVKI 131
```

```
Score = 42 (23.0 bits)
Identities = 5/9 (55%), Positives = 7/9 (77%)

Query:  121 KHLNLTGLK 129
            K + +TGLK
Sbjct:   91 KNIXITGLK 99
```

```
Score = 38 (21.0 bits)
Identities = 4/6 (66%), Positives = 6/6 (100%)

Query:  251 LLPELK 256
            ++PELK
Sbjct:   72 IIPELK 77
```

```
Score = 37 (20.5 bits)
Identities = 5/6 (83%), Positives = 6/6 (100%)

Query:  135 ALGSSQ 140
            A+GSSQ
Sbjct:  150 AIGSSQ 155
```

```
Score = 34 (19.0 bits)
Identities = 4/5 (80%), Positives = 5/5 (100%)

Query:  298 DFKNV 302
            DFKN+
Sbjct:   89 DFKNI 93
```

Spot 41

m/z	Charge	Peak mW	Peptide mW	Delta (Da)	Delta (ppm)	Ladder Score	Log Likelihood	Start	End	Sequence	Modifications	Subs	Query Tool
564,7482	2	1127,4808	1127,4918	-0,0110	-9,7775	40,3509	77,2838	1	10	STNCTYTPGK	Carbamidomethyl C	4	De-novo
564,7482	2	1127,4808	1127,4918	-0,0110	-9,7775	40,3509	77,2332	1	10	TSNCTYTPGK	Carbamidomethyl C	4	De-novo
564,7482	2	1127,4808	1127,4918	-0,0110	-9,7775	49,1228	76,2329	1	10	NSTCTYTPGK	Carbamidomethyl C	4	De-novo
473,7394	2	945,4632	945,488	-0,0248	-26,2552	75,5556	213,7926	1	8	TNLQVTDR			De-novo
543,241	2	1084,4664	1084,4683	-0,0019	-1,7741	57,8947	120,9244	1	10	VCSACYTPGK	Carbamidomethyl C	2	De-novo
543,241	2	1084,4664	1084,4681	-0,0017	-1,5897	43,1373	118,3279	1	9	VCMNYTPGK	Carbamidomethyl C /	2 / 3	De-novo
543,241	2	1084,4664	1084,4683	-0,0019	-1,7741	57,8947	119,5724	1	10	CVSACYTPGK	Carbamidomethyl C	1	De-novo
660,8152	2	1319,6148	1319,614	0,0008	0,5881	66,6667	217,0034	1	12	NVADVLSQSACR	Deamidation N /	1 / 11	De-novo
660,8152	2	1319,6148	1319,614	0,0008	0,5881	66,6667	216,6714	1	12	VNADVLSQSACR	Deamidation N /	2 / 11	De-novo
660,8152	2	1319,6148	1319,6139	0,0009	0,6638	66,6667	216,7681	1	12	NVANVLSQSACR	Deamidation N /	1 / 4 / 11	De-novo
464,2169	2	926,4182	926,4246	-0,0064	-6,9342	62,2222	142,8214	1	8	FNTNFAGR	Deamidation N	4	De-novo
464,2169	2	926,4182	926,4247	-0,0065	-7,0421	62,2222	141,624	1	8	FNTDFAGR			De-novo
464,2169	2	926,4182	926,4246	-0,0064	-6,9342	48,8889	138,9159	1	8	FSQNFAGR	Deamidation N	4	De-novo
480,235	2	958,4544	958,4906	-0,0362	-37,7928	49,0196	103,2884	1	9	CPGVLLSNR	Deamidation N	8	De-novo
480,235	2	958,4544	958,4907	-0,0363	-37,8971	66,6667	103,269	1	8	PCVLLSNR	Carbamidomethyl C /	2 / 7	De-novo
480,235	2	958,4544	958,4906	-0,0362	-37,7928	54,902	104,7224	1	9	PCGVLLSNR	Deamidation N	8	De-novo
487,7863	2	973,5570	973,5596	-0,0026	-2,6953	62,7451	138,6766	1	9	VTLLGWGTK			De-novo
487,7863	2	973,5570	973,5596	-0,0026	-2,6953	58,8235	133,2762	1	9	TVLLGWGTK			De-novo
487,7863	2	973,5570	973,5596	-0,0026	-2,6953	50,9804	130,054	1	9	VTLLGWSAK			De-novo

NCBI gi|239835736|ref|NP_001155191.1| silk gland derived serine protease [Bombyx mori] Total Score: 276

```
                          0        80      160      240      320
                          |         |        |        |        |        |392
gi|239835736|ref|NP_0  |  _____________________________________________
Local hits (HSPs)      |    _                _         _  _
```

```
Score = 60 (32.5 bits)
Identities = 8/12 (66%), Positives = 10/12 (83%)

Query:    80 VNADVLSQSACR 91
             V+ DV+SQS CR
Sbjct:   301 VDLDVISQSTCR 312   MatchSeq
```

```
Score = 56 (30.4 bits)
Identities = 7/8 (87%), Positives = 8/8 (100%)

Query:   154 VTLLGWGT 161
             VT+LGWGT
Sbjct:   280 VTILGWGT 287   MatchSeq
```

```
Score = 51 (27.9 bits)
Identities = 7/9 (77%), Positives = 8/9 (88%)

Query:   169 GTKVTLLGW 177
             G KVT+LGW
Sbjct:   277 GLKVTILGW 285   MatchSeq
```

```
Score = 40 (22.2 bits)
Identities = 6/9 (66%), Positives = 7/9 (77%)

Query:   128 CPGVLLSNR 136
             C G L+SNR
Sbjct:   181 CGGALISNR 189   MatchSeq
```

```
Score = 37 (20.7 bits)
Identities = 5/6 (83%), Positives = 5/6 (83%)

Query:   105 NTNFAG 110
             NTNF G
Sbjct:   272 NTNFTG 277   MatchSeq
```

```
Score = 32 (18.2 bits)
Identities = 4/5 (80%), Positives = 4/5 (80%)

Query:    52 NYTPG 56
             NY PG
Sbjct:    44 NYSPG 48   MatchSeq
```

Ha SG EST hargln|HA-GLN_Contig_1255_FWD_frame_2 Total Score: 297

```
                          0       110      220      330      440
                          |         |        |        |        |        |516
hargln|HA-GLN_Contig_  |  _____________________________________________
Local hits (HSPs)      |                  _        _ ___ _
```

```
Score = 71 (38.1 bits)
Identities = 9/10 (90%), Positives = 10/10 (100%)

Query:   153 RVTLLGWGTK 162
             RVT+LGWGTK
Sbjct:   302 RVTILGWGTK 311
```

```
Score = 64 (34.5 bits)
Identities = 8/9 (88%), Positives = 9/9 (100%)

Query:    71 DVLSQSACR 79
             DV+SQSACR
Sbjct:   327 DVISQSACR 335
```

```
Score = 62 (33.5 bits)
Identities = 8/9 (88%), Positives = 9/9 (100%)

Query:   200 KFNTNFAGR 208
             KFNT+FAGR
Sbjct:   293 KFNTDFAGR 301
```

```
Score = 57 (30.9 bits)
Identities = 7/8 (87%), Positives = 8/8 (100%)

Query:    14 CTYTPGKN 21
             CTYTPGK+
Sbjct:   346 CTYTPGKD 353
```

```
Score = 43 (23.8 bits)
Identities = 5/9 (55%), Positives = 8/9 (88%)

Query:   128 CPGVLLSNR 136
             C GV++S+R
Sbjct:   205 CGGVIISDR 213
```

Spot 42

m/z	Charge	Peak mW	Peptide mW	Delta (Da)	Delta (ppm)	Ladder Score	Log Likelihood	Start	End	Sequence	Modifications	Subs	Query Tool
459,2508	2	916,4860	916,4865	-0,0005	-0,5717	62,7451	148,5923	1	9	VASLTNSPK	Deamidation N	6	De-novo
459,2508	2	916,4860	916,4865	-0,0005	-0,5717	62,7451	144,976	1	9	VASLTDSPK			De-novo
591,2761	2	1180,5366	1180,5334	0,0032	2,6903	54,902	75,1948	1	9	WVVAFCNER	Carbamidomethyl C /	6 / 7	De-novo
591,2761	2	1180,5366	1180,5336	0,0030	2,5209	56,8627	76,1937	1	9	WVVANCFER	Deamidation N /	5 / 6	De-novo
591,2761	2	1180,5366	1180,5334	0,0032	2,6903	54,902	74,3429	1	9	WVVAFCNQR	Carbamidomethyl C /	6 / 7 / 8	De-novo
574,3413	2	1146,6670	1146,6648	0,0022	1,8977	64,9123	178,2348	1	10	LTLTFPTLNK			De-novo
574,3413	2	1146,6670	1146,6647	0,0023	1,9849	61,4035	178,1688	1	10	TLLTFPTLNK			De-novo
574,3413	2	1146,6670	1146,6648	0,0022	1,8977	59,6491	174,1516	1	10	LTTLFPTLNK			De-novo
808,9249	2	1615,8342	1615,8304	0,0038	2,3369	60,4938	246,978	1	14	ETLLVSNQEELVNK	Deamidation N	7	De-novo
808,9249	2	1615,8342	1615,8304	0,0038	2,3369	62,963	246,8042	1	14	TELLVSNQEELVNK	Deamidation N	7	De-novo
808,9249	2	1615,8342	1615,8304	0,0038	2,3369	65,4321	246,3331	1	14	ETLLVSNEQELVNK	Deamidation N	7	De-novo

NCBI gi|124108018|gb|ABM90638.1| 6-phosphogluconolactonase [Bombyx mori] Total Score: 182

```
                          0        50      100      150      200
                          |         |        |        |        |        |233
gi|124108018|gb|ABM90  |  _____________________________________________
Local hits (HSPs)      |      ___                  ___
```

```
Score = 68 (35.2 bits)
Identities = 9/14 (64%), Positives = 10/14 (71%)

Query:    14 TDSPKWVVAFCNER 27
             TD  KW  AFC+ER
Sbjct:    56 TDWSKWTLAFCDER 69   MatchSeq
```

```
Score = 61 (31.8 bits)
Identities = 8/10 (80%), Positives = 9/10 (90%)

Query:     9 KVASLTDSPK 18
             KVA +TDSPK
Sbjct:   158 KVAAITDSPK 167   MatchSeq
```

```
Score = 53 (27.8 bits)
Identities = 6/11 (54%), Positives = 10/11 (90%)

Query:    44 QRLTLTFPTLN 54
             +R+TLT+P +N
Sbjct:   171 ERITLTYPVIN 181   MatchSeq
```

Ha SG EST hargln|HA-GLN_Contig_1667_FWD_frame_0 Total Score: 311

```
                          0        50      100      150      200
                          |         |        |        |        |        |245
hargln|HA-GLN_Contig_  |  _____________________________________________
Local hits (HSPs)      |     __       ___                  _   __
```

```
Score = 88 (45.0 bits)
Identities = 11/14 (78%), Positives = 14/14 (100%)

Query:   105 TLLVSNEQELVNKL 118
             T++VS+EQELVNKL
Sbjct:    33 TIIVSDEQELVNKL 46
```

```
Score = 79 (40.6 bits)
Identities = 10/14 (71%), Positives = 12/14 (85%)

Query:    14 TDSPKWVVAFCNER 27
             T+  KWVVAFC+ER
Sbjct:    86 TEWSKWVVAFCDER 99
```

```
Score = 77 (39.6 bits)
Identities = 9/12 (75%), Positives = 12/12 (100%)

Query:    44 QRLTLTFPTLNK 55
             +R+TLTFPT+NK
Sbjct:   201 ERITLTFPTINK 212
```

```
Score = 67 (34.7 bits)
Identities = 10/10 (100%), Positives = 10/10 (100%)

Query:     9 KVASLTDSPK 18
             KVASLTDSPK
Sbjct:   188 KVASLTDSPK 197
```

Spot 43

m/z	Charge	Peak mW	Peptide mW	Delta (Da)	Delta (ppm)	Ladder Score	Log Likelihood	Start	End	Sequence	Modifications	Subs	Query Tool
444,7393	2	887,4630	887,46	0,0030	3,3534	53,3333	164,7986	1	8	LVSPENTK	Deamidation N	6	De-novo
444,7393	2	887,4630	887,46	0,0030	3,3534	53,3333	164,1954	1	8	LVSPEDTK			De-novo
444,7393	2	887,4630	887,46	0,0030	3,3534	51,1111	162,1601	1	8	VLSPENTK	Deamidation N	6	De-novo
625,3066	2	1248,5976	1248,606	-0,0084	-6,7467	42,8571	95,0873	1	11	SSMLNLPVQFP	Oxidation M / Deamidation	3 / 9	De-novo
625,3066	2	1248,5976	1248,606	-0,0084	-6,7467	42,8571	93,9477	1	11	SSMLNLPVEFP	Oxidation M	3	De-novo
625,3066	2	1248,5976	1248,606	-0,0084	-6,7467	46,0317	92,6949	1	11	SMSLNLPVQFP	Oxidation M / Deamidation	2 / 9	De-novo
647,3583	2	1292,7010	1292,6975	0,0035	2,6890	72	208,257	1	13	LGVGLVSGSNYVK	Deamidation N	10	De-novo
647,3583	2	1292,7010	1292,6975	0,0035	2,6890	77,3333	208,7637	1	13	VAVGLVSGSNYVK	Deamidation N	10	De-novo
647,3583	2	1292,7010	1292,6975	0,0035	2,6890	69,3333	206,6004	1	13	GLVGLVSGSNYVK	Deamidation N	10	De-novo
473,2657	2	944,5158	944,5179	-0,0021	-2,2488	57,7778	140,6344	1	8	LNLSEELK			De-novo
473,2657	2	944,5158	944,5179	-0,0021	-2,2488	60	140,5884	1	8	NLLSEELK			De-novo
434,7193	2	867,4230	867,424	-0,0010	-1,1805	64,1026	89,8329	1	7	GNFVEFR			De-novo
434,7193	2	867,4230	867,424	-0,0010	-1,1805	64,1026	89,5233	1	7	NGFVEFR			De-novo

NCBI gi|195126393|ref|XP_002007655.1| GI12260 [Drosophila mojavensis] Total Score: 148

```
                          0        60      120      180      240
                          |         |        |        |        |  |250
gi|195126393|ref|XP_0  |  _____________________________________________
Local hits (HSPs)      |       __            ___
```

```
Score = 58 (30.4 bits)
Identities = 6/11 (54%), Positives = 8/11 (72%)

Query:   122 FVEFRSSMLNL 132
             F+EFR  M N+
Sbjct:   121 FIEFRNGMMNI 131   MatchSeq
```

```
Score = 54 (28.4 bits)
Identities = 7/9 (77%), Positives = 8/9 (88%)

Query:   113 GNFVEFRNG 121
             G F+EFRNG
Sbjct:   119 GTFIEFRNG 127   MatchSeq
```

```
Score = 36 (19.5 bits)
Identities = 5/11 (45%), Positives = 8/11 (72%)

Query:    70 KVAVGLVSGSN 80
             K  +G+V GS+
Sbjct:    39 KATIGIVGGSD 49   MatchSeq
```

ButterflyBase LOP00661_1 Total Score: 328

```
                          0        40       80      120      160
                          |         |        |        |        |   |177
db|LOP00661_1|LOP0066  |  _____________________________________________
Local hits (HSPs)      |     ___ ___       _         ___
```

```
Score = 84 (43.2 bits)
Identities = 10/14 (71%), Positives = 12/14 (85%)

Query:   119 RNGFVEFRSSMLNL 132
             R  FVEFRSSM+N+
Sbjct:   105 RGNFVEFRSSMINI 118
```

```
Score = 77 (39.7 bits)
Identities = 11/13 (84%), Positives = 13/13 (100%)

Query:    71 VAVGLVSGSNYVK 83
             VA+GLVSGS+YVK
Sbjct:    27 VAIGLVSGSDYVK 39
```

```
Score = 72 (37.3 bits)
Identities = 10/13 (76%), Positives = 13/13 (100%)

Query:    60 VGLVSGSNYVKVA 72
             +GLVSGS+YVK+A
Sbjct:    29 IGLVSGSDYVKIA 41
```

```
Score = 58 (30.4 bits)
Identities = 9/14 (64%), Positives = 10/14 (71%)

Query:    99 LSEELKNLLSEELK 112
             + EELKN L EE K
Sbjct:    11 ITEELKNFLLEEVK 24
```

```
Score = 37 (20.0 bits)
Identities = 6/8 (75%), Positives = 6/8 (75%)

Query:     6 NTKLVSPE 13
             N KLVS E
Sbjct:    67 NNKLVSSE 74
```

Ha SG EST hargln|HA-GLN_Contig_1207_FWD_frame_1 Total Score: 279

```
                          0        50      100      150      200
                          |         |        |        |        |   |226
hargln|HA-GLN_Contig_  |  _____________________________________________
Local hits (HSPs)      |                 ___ ___            ____
```

```
Score = 84 (43.2 bits)
Identities = 10/14 (71%), Positives = 12/14 (85%)

Query:   119 RNGFVEFRSSMLNL 132
             R  FVEFRSSM+N+
Sbjct:   188 RGNFVEFRSSMINI 201
```

```
Score = 79 (40.7 bits)
Identities = 12/14 (85%), Positives = 13/14 (92%)

Query:    70 KVAVGLVSGSNYVK 83
             KVAVGLVSGS+ VK
Sbjct:   109 KVAVGLVSGSDXVK 122
```

```
Score = 67 (34.8 bits)
Identities = 10/13 (76%), Positives = 12/13 (92%)

Query:    60 VGLVSGSNYVKVA 72
             VGLVSGS+ VK+A
Sbjct:   112 VGLVSGSDXVKIA 124
```

```
Score = 49 (25.9 bits)
Identities = 7/9 (77%), Positives = 8/9 (88%)

Query:    99 LSEELKNLL 107
             +SEELKN L
Sbjct:    94 ISEELKNFL 102
```

Spot 44

m/z	Charge	Peak mW	Peptide mW	Delta (Da)	Delta (ppm)	Ladder Score	Log Likelihood	Start	End	Sequence	Modifications	Subs	Query Tool
496,2513	2	990,4870	990,4805	0,0065	6,5382	52,6316	91,1739	1	10	MAEAVASQGK			De-novo
496,2513	2	990,4870	990,4805	0,0065	6,5382	52,6316	88,6707	1	10	AMEAVASQGK			De-novo
496,2513	2	990,4870	990,4805	0,0065	6,5382	50,8772	88,8823	1	10	MAEAVTGQGK			De-novo
630,3046	2	1258,5936	1258,5869	0,0067	5,3044	41,2698	100,406	1	11	FSNYPPPPPDK	Deamidation N	3	De-novo
630,3046	2	1258,5936	1258,5869	0,0067	5,3044	41,2698	100,0423	1	11	FSNYPPPPPNK	Deamidation N	3 / 10	De-novo
630,3046	2	1258,5936	1258,5869	0,0067	5,3044	41,2698	99,1999	1	11	FSNYPPPPNPK	Deamidation N	3 / 9	De-novo
625,3224	2	1248,6292	1248,6292	0,0000	-0,0192	57,8947	37,8353	1	10	YWAKGPDVWK			De-novo

NCBI gi|195491779|ref|XP_002093710.1| GE20611 [Drosophila yakuba] Total Score: 265

```
                      0         90        180       270       360
                      |         |         |         |         |     |401
gi|195491779|ref|XP_0 | ______________________________________________
Local hits (HSPs)     |                 ___      _  _         _
```

Score = 59 (31.0 bits)
Identities = 11/16 (68%), Positives = 11/16 (68%)

```
Query:   35 PPPPPDKFSNYPPPPP 50
            PPPPP K   Y PPPP
Sbjct:  163 PPPPPKKVVVYTPPPP 178   MatchSeq
```

Score = 46 (24.6 bits)
Identities = 6/7 (85%), Positives = 6/7 (85%)

```
Query:   46 PPPPPNK 52
            PPPPP K
Sbjct:  178 PPPPPKK 184   MatchSeq
```

Score = 43 (23.1 bits)
Identities = 6/7 (85%), Positives = 6/7 (85%)

```
Query:   78 PPPPPDK 84
            PPPPP K
Sbjct:  273 PPPPPKK 279   MatchSeq
```

Score = 41 (22.1 bits)
Identities = 6/7 (85%), Positives = 6/7 (85%)

```
Query:   57 PPPPNPK 63
            PPPP PK
Sbjct:  272 PPPPPPK 278   MatchSeq
```

Score = 38 (20.6 bits)
Identities = 5/6 (83%), Positives = 5/6 (83%)

```
Query:   77 YPPPPP 82
            Y PPPP
Sbjct:  364 YTPPPP 369   MatchSeq
```

Score = 38 (20.6 bits)
Identities = 5/6 (83%), Positives = 5/6 (83%)

```
Query:   34 YPPPPP 39
            Y PPPP
Sbjct:  248 YTPPPP 253   MatchSeq
```

ButterflyBase SFP01905_2 Total Score: 114

```
                      0         60        120       180       240
                      |         |         |         |         |  |255
butterfly|SFP01905_2  | ______________________________________________
Local hits (HSPs)     |                                         __
```

Score = 66 (34.5 bits)
Identities = 9/11 (81%), Positives = 9/11 (81%)

```
Query:   10 KAMEAVASQGK 20
            KAME VA QGK
Sbjct:  243 KAMESVANQGK 253
```

Score = 48 (25.6 bits)
Identities = 7/9 (77%), Positives = 7/9 (77%)

```
Query:    3 EAVASQGKA 11
            E VA QGKA
Sbjct:  246 ESVANQGKA 254
```

Ha SG EST hargln|HA-GLN_Contig_620_FWD_frame_2 Total Score: 138

```
                      0         90        180       270       360
                      |         |         |         |         |     |400
hargln|HA-GLN_Contig_ | ______________________________________________
Local hits (HSPs)     |                               _
```

Score = 78 (40.4 bits)
Identities = 11/11 (100%), Positives = 11/11 (100%)

```
Query:   10 KAMEAVASQGK 20
            KAMEAVASQGK
Sbjct:  275 KAMEAVASQGK 285
```

Score = 60 (31.5 bits)
Identities = 9/9 (100%), Positives = 9/9 (100%)

```
Query:    3 EAVASQGKA 11
            EAVASQGKA
Sbjct:  278 EAVASQGKA 286
```

Spot 45

m/z	Charge	Peak mW	Peptide mW	Delta (Da)	Delta (ppm)	Ladder Score	Log Likelihood	Start	End	Sequence	Modifications	Subs	Query Tool
540,7523	2	1079,4890	1079,4924	-0,0034	-3,1719	72,549	162,7442	1	9	FSSNAYTYK			De-novo
570,2921	2	1138,5686	1138,5692	-0,0006	-0,5481	68,4211	108,9067	1	10	YTLTNPGVMK	Oxidation M	9	De-novo
570,2921	2	1138,5686	1138,5692	-0,0006	-0,5481	68,4211	108,8116	1	10	TYLTNPGVMK	Oxidation M	9	De-novo
562,2943	2	1122,5730	1122,5743	-0,0013	-1,1794	59,6491	143,9994	1	10	TVLTNPGYMK			De-novo
562,2943	2	1122,5730	1122,5743	-0,0013	-1,1794	59,6491	142,9108	1	10	VTLTNPGYMK			De-novo
562,2943	2	1122,5730	1122,5742	-0,0012	-1,0904	56,1404	140,0638	1	10	SLLTNPGYMK			De-novo
782,383	2	1562,7504	1562,7577	-0,0073	-4,6866	46,6667	73,0557	1	13	TNLVNEYVTQQPR	Deamidation N /	2 / 10	De-novo
782,383	2	1562,7504	1562,7577	-0,0073	-4,6866	46,6667	72,8662	1	13	TDLVNEYVTQQPR	Deamidation Q	10	De-novo
782,383	2	1562,7504	1562,7577	-0,0073	-4,6866	46,6667	76,6089	1	13	TNLVNEYVTQQRP	Deamidation N /	2 / 10	De-novo
464,7653	2	927,5150	927,5178	-0,0028	-3,0447	64,7059	116,9978	1	9	PGLVGATWK			De-novo
464,7653	2	927,5150	927,5178	-0,0028	-3,0447	64,7059	116,5811	1	9	GPLVGATWK			De-novo
464,7653	2	927,5150	927,5178	-0,0028	-3,0447	54,902	111,3302	1	9	LPGVGATWK			De-novo
651,8307	2	1301,6458	1301,6462	-0,0004	-0,3257	60	328,8337	1	13	NAGTGGLENEVLK	Deamidation N	1	De-novo
651,8307	2	1301,6458	1301,6462	-0,0004	-0,3257	60	328,0541	1	13	ANGTGGLENEVLK	Deamidation N	2	De-novo
651,8307	2	1301,6458	1301,6462	-0,0004	-0,3257	60	327,5308	1	13	NAGTGGLENQVLK	Deamidation N /	1 / 10	De-novo

NCBI gi|91087863|ref|XP_968982.1| PREDICTED: similar to AGAP001957-PA [Tribolium castaneum] Total Score: 311

```
                      0         60        120       180       240
                      |         |         |         |         |    |272
gi|91087863|ref|XP_96 | ______________________________________________
Local hits (HSPs)     |      _ ___     __           __
```

Score = 76 (39.6 bits)
Identities = 9/10 (90%), Positives = 10/10 (100%)

```
Query:   30 TVLTNPGYMK 39
            TV+TNPGYMK
Sbjct:  101 TVITNPGYMK 110   MatchSeq
```

Score = 68 (35.7 bits)
Identities = 9/12 (75%), Positives = 10/12 (83%)

```
Query:   52 LTNPGYMKTNLV 63
            +TNPGYMK N V
Sbjct:  103 ITNPGYMKENFV 114   MatchSeq
```

Score = 48 (25.7 bits)
Identities = 7/9 (77%), Positives = 7/9 (77%)

```
Query:  108 GPLVGATWK 116
            GPLVG  WK
Sbjct:  176 GPLVGPNWK 184   MatchSeq
```

Score = 42 (22.7 bits)
Identities = 6/8 (75%), Positives = 6/8 (75%)

```
Query:  186 LVGATWKN 193
            LVG  WKN
Sbjct:  178 LVGPNWKN 185   MatchSeq
```

Score = 42 (22.7 bits)
Identities = 6/10 (60%), Positives = 7/10 (70%)

```
Query:  164 KFSSNAYTYK 173
            K+ S  YTYK
Sbjct:   56 KYTSGQYTYK 65   MatchSeq
```

Score = 35 (19.3 bits)
Identities = 5/5 (100%), Positives = 5/5 (100%)

```
Query:  148 EVLKN 152
            EVLKN
Sbjct:   40 EVLKN 44   MatchSeq
```

ButterflyBase BMP020939_1 Total Score: 317

```
                      0         60        120       180       240
                      |         |         |         |         |    |262
db|BMP020939_1|BMP020 | ______________________________________________
Local hits (HSPs)     |        _  _      __         _
```

Score = 76 (39.6 bits)
Identities = 10/10 (100%), Positives = 10/10 (100%)

```
Query:   30 TVLTNPGYMK 39
            TVLTNPGYMK
Sbjct:  101 TVLTNPGYMK 110
```

Score = 68 (35.7 bits)
Identities = 10/12 (83%), Positives = 10/12 (83%)

```
Query:   52 LTNPGYMKTNLV 63
            LTNPGYMK N V
Sbjct:  103 LTNPGYMKENFV 114
```

Score = 48 (25.7 bits)
Identities = 7/9 (77%), Positives = 7/9 (77%)

```
Query:  108 GPLVGATWK 116
            GPLVG  WK
Sbjct:  176 GPLVGPNWK 184
```

Score = 48 (25.7 bits)
Identities = 7/10 (70%), Positives = 8/10 (80%)

```
Query:  164 KFSSNAYTYK 173
            K+SS  YTYK
Sbjct:   56 KYSSGQYTYK 65
```

Score = 42 (22.7 bits)
Identities = 6/8 (75%), Positives = 6/8 (75%)

```
Query:  186 LVGATWKN 193
            LVG  WKN
Sbjct:  178 LVGPNWKN 185
```

Score = 35 (19.3 bits)
Identities = 4/5 (80%), Positives = 5/5 (100%)

```
Query:  148 EVLKN 152
            EV+KN
Sbjct:   40 EVIKN 44
```

Ha SG other none

Ha EST other harmi_rn|HA-RN-ALL_Contig_4648_FWD_frame_0 Total Score: 153

```
                      0         100       200       300       400
                      |         |         |         |         |      |497
harmi_rn|HA-RN-ALL_Co | ______________________________________________
Local hits (HSPs)     |                                       _   _
```

Score = 60 (31.7 bits)
Identities = 9/11 (81%), Positives = 9/11 (81%)

```
Query:   62 LVNEYVTQQPR 72
            LVNEYVT Q R
Sbjct:  436 LVNEYVTPQER 446
```

Score = 58 (30.7 bits)
Identities = 9/12 (75%), Positives = 9/12 (75%)

```
Query:   85 RTNLVNEYVTQQ 96
            R  LVNEYVT Q
Sbjct:  433 RDTLVNEYVTPQ 444
```

Score = 35 (19.3 bits)
Identities = 5/7 (71%), Positives = 5/7 (71%)

```
Query:   79 YVTQQPR 85
            YVT  PR
Sbjct:  474 YVTPDPR 480
```

Spot 46

m/z	Charge	Peak mW	Peptide mW	Delta (Da)	Delta (ppm)	Ladder Score	Log Likelihood	Start	End	Sequence	Modifications	Subs	Query Tool
889,4558	2	1776,8960	1776,8895	0,0065	3,6446	66,6667	207,0847	1	16	ELNYPSTVQLTELGGR	Deamidation N	3	De-novo
889,4558	2	1776,8960	1776,8895	0,0065	3,6446	68,8172	206,5866	1	16	LENYPSTVQLTELGGR	Deamidation N	3	De-novo
889,4558	2	1776,8960	1776,8895	0,0065	3,6446	66,6667	206,3008	1	16	QLNYPSTVQLTELGGR	Deamidation Q /	1 / 3	De-novo
846,0727	3	2535,1947	2535,1511	0,0436	17,1840	43,2624	238,8504	1	24	NQPANPSEAHSFPSPLLQNEGDGK	Deamidation Q /	2 / 19	De-novo
846,0727	3	2535,1947	2535,137	0,0577	22,7459	43,9716	239,312	1	24	NQPANPMGVHSEAMPLLQNEGDGK	Deamidation Q /	2 / 19	De-novo
846,0727	3	2535,1947	2535,1182	0,0765	30,1619	46,0993	238,7617	1	24	NQPANPSEAHSEAMPLLQNEGDGK	Deamidation Q /	2 / 19	De-novo
665,8446	2	1329,6736	1329,6677	0,0059	4,4191	55,0725	78,933	1	12	GGNVWEVNSVLR	Deamidation N	3	De-novo
665,8446	2	1329,6736	1329,6677	0,0059	4,4191	55,0725	78,1563	1	12	GGDVWEVNSVLR			De-novo
665,8446	2	1329,6736	1329,6677	0,0059	4,4191	40,5797	74,2999	1	12	GGDVWEVNGKLR	Deamidation N	8	De-novo
627,3251	2	1252,6346	1252,5909	0,0437	34,8685	57,8947	71,0642	1	10	WLDTQVMFGR	Deamidation Q	5	De-novo
627,3251	2	1252,6346	1252,5909	0,0437	34,8685	66,6667	70,8434	1	10	WLDTQVMGFR	Deamidation Q	5	De-novo

NCBI gi|2463066|emb|CAA72953.1| diverged serine protease [Helicoverpa armigera] Total Score: 171

```
                      0         60        120       180       240
                      |         |         |         |         |    |256
gi|2463066|emb|CAA729 | ______________________________________________
Local hits (HSPs)     |    __         ___
```

Score = 97 (49.7 bits)
Identities = 12/13 (92%), Positives = 13/13 (100%)

```
Query:  132 RGGDVWEVNSVLR 144
            RGGDVWEVNSV+R
Sbjct:   87 RGGDVWEVNSVIR 99   MatchSeq
```

Score = 74 (38.3 bits)
Identities = 9/11 (81%), Positives = 11/11 (100%)

```
Query:   16 RLENYPSTVQL 26
            R+E+YPSTVQL
Sbjct:   27 RIEDYPSTVQL 37   MatchSeq
```

Ha SG EST hargln|HA-GLN_Contig_1408_FWD_frame_0 Total Score: 69

```
                      0         60        120       180       240
                      |         |         |         |         |    |263
hargln|HA-GLN_Contig_ | ______________________________________________
Local hits (HSPs)     |                              ___
```

Score = 69 (35.9 bits)
Identities = 9/11 (81%), Positives = 11/11 (100%)

```
Query:   62 SPLLQNEGDGK 72
            SPL+Q+EGDGK
Sbjct:  177 SPLIQDEGDGK 187
```

Spot 47

m/z	Charge	Peak mW	Peptide mW	Delta (Da)	Delta (ppm)	Ladder Score	Log Likelihood	Start	End	Sequence	Modifications	Subs	Query Tool
473,7449	2	945,4742	945,4767	-0,0025	-2,6696	57,7778	109,0847	1	8	LGETQLER	Deamidation Q	5	De-novo
473,7449	2	945,4742	945,4767	-0,0025	-2,6696	64,4444	107,9316	1	8	LGETLEQR	Deamidation Q	7	De-novo
473,7449	2	945,4742	945,4767	-0,0025	-2,6696	64,4444	107,4949	1	8	LGETLQER	Deamidation Q	6	De-novo
440,2306	2	878,4456	878,4498	-0,0042	-4,8085	61,5385	99,7415	1	7	TEQVVFR	Deamidation Q	3	De-novo
440,2306	2	878,4456	878,4498	-0,0042	-4,8085	61,5385	98,3694	1	7	TEEVVFR			De-novo
440,2306	2	878,4456	878,4498	-0,0042	-4,8085	61,5385	97,3014	1	7	TQEVVFR	Deamidation Q	2	De-novo
514,3121	2	1026,6086	1026,6073	0,0013	1,2429	57,8947	91,9836	1	10	VALNLEAAVK			De-novo
883,8966	2	1765,7776	1765,8749	-0,0973	-55,1138	38,0952	78,8296	1	18	SGPNPNAPGLLSGWGNTK			De-novo
897,9111	2	1793,8066	1793,818	-0,0114	-6,3685	33,3333	75,7923	1	17	SWSAEAADSLSGRSENK			De-novo
897,9111	2	1793,8066	1793,818	-0,0114	-6,3685	33,3333	75,0566	1	17	SSWAEAADSLSGRSENK			De-novo
897,9111	2	1793,8066	1793,818	-0,0114	-6,3685	33,3333	75,8583	1	17	SWSAEAADSLSGRSQNK	Deamidation Q	15	De-novo
695,3581	2	1388,7006	1388,7083	-0,0077	-5,5620	68,1159	78,8869	1	12	VLACLGTKNEER	Carbamidomethyl C	4	De-novo
695,3581	2	1388,7006	1388,7083	-0,0077	-5,5620	68,1159	79,0346	1	12	VLACLGTKNQER	Carbamidomethyl C /	4 / 10	De-novo
695,3581	2	1388,7006	1388,7083	-0,0077	-5,5620	68,1159	78,4525	1	12	VLACLGTKNEQR	Carbamidomethyl C /	4 / 11	De-novo
691,3283	2	1380,6410	1380,7289	-0,0879	-63,6794	45,3333	103,6449	1	13	GFTLTPATLFGEK			De-novo

NCBI gi|259016091|gb|ACV89326.1| triosephosphate isomerase [Nigronia sp. Total Score: 121

```
                 0         40        80        120
                 |         |         |         |         |158
gi|259016091|gb|ACV89  | ________________________________________
Local hits (HSPs)      |                          _________
```

Score = 65 (34.1 bits)
Identities = 9/13 (69%), Positives = 11/13 (84%)

```
Query:  124 KVLACLGTKNEER 136
            KV+AC+G K EER
Sbjct:  113 KVIACIGEKLEER 125   MatchSeq
```

Score = 56 (29.6 bits)
Identities = 10/18 (55%), Positives = 12/18 (66%)

```
Query:  173 KTEQVVFRVALNLEAAVK 190
            KTE+VVFR    + A VK
Sbjct:  129 KTEEVVFRQTKAIAAVVK 146   MatchSeq
```

ButterflyBase none

Ha SG EST none

Ha EST other harmi_rn|HA-RN-ALL_Contig_464_FWD_frame_1 Total Score: 157

```
                 0         60        120       180       240
                 |         |         |         |         |     |269
harmi_rn|HA-RN-ALL_Co  | ________________________________________
Local hits (HSPs)      |                     _  ___
```

Score = 58 (30.6 bits)
Identities = 8/13 (61%), Positives = 10/13 (76%)

```
Query:  124 KVLACLGTKNEER 136
            KV+AC+G   EER
Sbjct:  156 KVIACIGEXLEER 168
```

Score = 52 (27.6 bits)
Identities = 7/10 (70%), Positives = 8/10 (80%)

```
Query:  168 TLFGEKTEQV 177
            T+FGEK E V
Sbjct:  134 TIFGEKDEXV 143
```

Score = 47 (25.1 bits)
Identities = 6/8 (75%), Positives = 7/8 (87%)

```
Query:  173 KTEQVVFR 180
            K E+VVFR
Sbjct:  172 KXEEVVFR 179
```

Spot 48

m/z	Charge	Peak mW	Peptide mW	Delta (Da)	Delta (ppm)	Ladder Score	Log Likelihood	Start	End	Sequence	Modifications	Subs	Query Tool
417,2172	2	832,4188	832,4291	-0,0103	-12,4023	48,7179	122,7674	1	7	SVEETLR			De-novo
417,2172	2	832,4188	832,4291	-0,0103	-12,4023	58,9744	121,9677	1	7	VSEETLR			De-novo
796,8626	2	1591,7096	1591,7123	-0,0027	-1,7114	47,3118	168,1999	1	16	FGQVCPANMAAGPGSK	Deamidation Q /	3 / 5	De-novo
796,8626	2	1591,7096	1591,6936	0,0160	10,0371	51,6129	167,4653	1	16	FGQVCPANSDAGPGSK	Deamidation Q /	3 / 5	De-novo
796,8626	2	1591,7096	1591,7123	-0,0027	-1,7114	49,4624	168,2517	1	16	FGQVCPANAMAGPGSK	Deamidation Q /	3 / 5	De-novo
606,8094	2	1211,6032	1211,6187	-0,0155	-12,8126	71,4286	244,4472	1	11	LVQAFAGYTDK			De-novo
606,3229	2	1210,6302	1210,6669	-0,0367	-30,3337	65,0794	331,0247	1	11	QLTVNDLPVGR			De-novo
606,3229	2	1210,6302	1210,6669	-0,0367	-30,3337	65,0794	330,5244	1	11	QLTVNNLPVGR	Deamidation N	6	De-novo
561,9722	3	1682,8932	1682,9357	-0,0425	-25,2749	43,4343	208,9323	1	17	NKAAVGPGGFLPLLSNK	Deamidation N	16	De-novo
561,9722	3	1682,8932	1682,9357	-0,0425	-25,2749	45,4545	208,6324	1	17	KNAAVGPGGFLPLLSNK	Deamidation N	16	De-novo
561,9722	3	1682,8932	1682,9355	-0,0423	-25,1560	44,086	205,7307	1	16	NKAAVGPNFLPLLSNK	Deamidation N	15	De-novo
778,414	2	1554,8124	1554,8075	0,0049	3,1361	65,5172	376,6709	1	15	NAGLGPMNLPLLSDK	Oxidation M	7	De-novo
778,414	2	1554,8124	1554,8075	0,0049	3,1361	60,9195	376,1727	1	15	ANGLGPMNLPLLSDK	Oxidation M	7	De-novo
778,414	2	1554,8124	1554,8076	0,0048	3,0718	59,1398	374,0828	1	16	NAGLGPMGGLPLLSDK	Oxidation M	7	De-novo
798,3912	2	1594,7668	1594,7627	0,0041	2,5559	71,6049	266,0037	1	14	YNGVLNEESGLPFR	Deamidation N	2	De-novo
798,3912	2	1594,7668	1594,7627	0,0041	2,5559	71,6049	265,9058	1	14	NYGVLNEESGLPFR	Deamidation N	1	De-novo
798,3912	2	1594,7668	1594,7627	0,0041	2,5559	71,6049	264,6414	1	14	DYGVLNEESGLPFR			De-novo
769,8845	2	1537,7534	1537,781	-0,0276	-17,9635	59,1398	290,6104	1	16	PGSPGPMGGLPLLSDK	Oxidation M	7	De-novo
769,8845	2	1537,7534	1537,781	-0,0276	-17,9635	61,2903	290,1576	1	16	GPSPGPMGGLPLLSDK	Oxidation M	7	De-novo
769,8845	2	1537,7534	1537,781	-0,0276	-17,9635	59,1398	290,2623	1	16	PGSPGPMGGLPLLSNK	Oxidation M / Deamidation	7 / 15	De-novo
761,9016	2	1521,7876	1521,7972	-0,0096	-6,3241	41,3793	51,7938	1	15	PPSGGPMNLLQQGVK			De-novo
761,9016	2	1521,7876	1521,7972	-0,0096	-6,3241	38,7097	52,3233	1	16	PPSGGPMNLLQGAGVK			De-novo
761,9016	2	1521,7876	1521,7972	-0,0096	-6,3241	48,2759	50,2418	1	15	PPSGGPMNLLQQVGK			De-novo
692,8689	2	1383,7222	1383,7227	-0,0005	-0,3787	55,0725	60,9096	1	12	FFVAPFPEVFGK			De-novo
692,8689	2	1383,7222	1383,7227	-0,0005	-0,3787	55,0725	60,6101	1	12	FFVAPFPEVGFK			De-novo
692,8689	2	1383,7222	1383,7227	-0,0005	-0,3787	55,0725	60,3377	1	12	FFVAPFPQVFGK	Deamidation Q	8	De-novo
788,8636	2	1575,7116	1575,714	-0,0024	-1,5384	67,9012	292,8636	1	14	FGEVCPANWQPGSK	Carbamidomethyl C	5	De-novo
788,8636	2	1575,7116	1575,714	-0,0024	-1,5384	65,4321	292,5542	1	14	GFEVCPANWQPGSK	Carbamidomethyl C	5	De-novo
788,8636	2	1575,7116	1575,714	-0,0024	-1,5384	67,9012	287,414	1	14	FGQVCPANWQPGSK	Deamidation Q /	3 / 5	De-novo
485,7777	2	969,5398	969,5396	0,0002	0,1815	60	71,1646	1	8	LAWLTNPR			De-novo
883,8947	2	1765,7738	1765,7943	-0,0205	-11,6231	52,5253	163,1767	1	17	ENSMDGAALLSGWGNTK	Oxidation M	4	De-novo
883,8947	2	1765,7738	1765,7943	-0,0205	-11,6231	52,5253	162,8592	1	17	NESMDGAALLSGWGNTK	Oxidation M	4	De-novo
883,8947	2	1765,7738	1765,7943	-0,0205	-11,6231	52,5253	163,7868	1	17	ENSMNGAALLSGWGNTK	Oxidation M / Deamidation	4 / 5	De-novo
460,7582	2	919,5008	919,5014	-0,0006	-0,6786	53,3333	83,0587	1	8	FLGLLDDK			De-novo
460,7582	2	919,5008	919,5014	-0,0006	-0,6786	53,3333	81,5941	1	8	LFGLLDDK			De-novo
460,7582	2	919,5008	919,5014	-0,0006	-0,6786	53,3333	82,5403	1	8	FLGLLDNK	Deamidation N	7	De-novo

NCBI gi|159459926|gb|ABW96360.1| thioredoxin peroxidase [Helicoverpa armigera] Total Score: 521

```
                 0         40        80        120       160
                 |         |         |         |         |       |195
gi|159459926|gb|ABW96  | ________________________________________
Local hits (HSPs)      |                   _____ ____   _____  ___
```

Score = 117 (60.6 bits)
Identities = 15/15 (100%), Positives = 15/15 (100%)

```
Query:  363 KFGEVCPANWQPGSK 377
            KFGEVCPANWQPGSK
Sbjct:  164 KFGEVCPANWQPGSK 178   MatchSeq
```

Score = 110 (57.1 bits)
Identities = 14/15 (93%), Positives = 15/15 (100%)

```
Query:  219 RDYGVLNEESGLPFR 233
            RDYGVLNEESG+PFR
Sbjct:  110 RDYGVLNEESGIPFR 124   MatchSeq
```

Score = 90 (47.0 bits)
Identities = 13/16 (81%), Positives = 14/16 (87%)

```
Query:  145 KNAGLGPMNLPLLSDK 160
            K  GLGPMN+PLLSDK
Sbjct:   89 KQGGLGPMNIPLLSDK 104   MatchSeq
```

Score = 84 (44.0 bits)
Identities = 12/12 (100%), Positives = 12/12 (100%)

```
Query:  489 SVEETLRLVQAF 500
            SVEETLRLVQAF
Sbjct:  148 SVEETLRLVQAF 159   MatchSeq
```

Score = 79 (41.5 bits)
Identities = 10/12 (83%), Positives = 12/12 (100%)

```
Query:   84 RQLTVNNLPVGR 95
            RQ+TVN+LPVGR
Sbjct:  136 RQITVNDLPVGR 147   MatchSeq
```

Score = 41 (22.4 bits)
Identities = 5/8 (62%), Positives = 6/8 (75%)

```
Query:  406 LAWLTNPR 413
            LAW+  PR
Sbjct:   81 LAWINTPR 88   MatchSeq
```

Ha SG EST hargln|HA-GLN_Contig_604_FWD_frame_1 Total Score: 233

```
                 0         70        140       210       280
                 |         |         |         |         |  |302
hargln|HA-GLN_Contig_  | ________________________________________
Local hits (HSPs)      |                              ___     ___
```

Score = 71 (37.5 bits)
Identities = 9/12 (75%), Positives = 11/12 (91%)

```
Query:   84 RQLTVNNLPVGR 95
            RQ+T N+LPVGR
Sbjct:  281 RQITMNDLPVGR 292
```

Score = 63 (33.5 bits)
Identities = 9/10 (90%), Positives = 10/10 (100%)

```
Query:  489 SVEETLRLVQ 498
            SV+ETLRLVQ
Sbjct:  293 SVDETLRLVQ 302
```

Score = 58 (30.9 bits)
Identities = 10/15 (66%), Positives = 11/15 (73%)

```
Query:  145 KNAGLGPMNLPLLSD 159
            K  GLG  N+PLLSD
Sbjct:  234 KEGGLGKINIPLLSD 248
```

Score = 41 (22.4 bits)
Identities = 5/8 (62%), Positives = 6/8 (75%)

```
Query:  406 LAWLTNPR 413
            LAW+  PR
Sbjct:  226 LAWINTPR 233
```

Spot 49

m/z	Charge	Peak mW	Peptide mW	Delta (Da)	Delta (ppm)	Ladder Score	Log Likelihood	Start	End	Sequence	Modifications	Subs	Query Tool
573,2947	2	1144,5738	1144,5724	0,0014	1,2022	50,8772	138,2743	1	10	QLNVETVNAR	Deamidation N	3 / 8	De-novo
573,2947	2	1144,5738	1144,5724	0,0014	1,2022	50,8772	137,5746	1	10	QLNVETVANR	Deamidation N	3 / 9	De-novo
573,2947	2	1144,5738	1144,5724	0,0014	1,2022	50,8772	133,1458	1	10	QLNVETSPTR	Deamidation N	3	De-novo
631,3115	2	1260,6074	1260,6061	0,0013	1,0122	58,7302	156,1956	1	11	MLYSSSALFDK			De-novo
631,3115	2	1260,6074	1260,6061	0,0013	1,0122	58,7302	156,3526	1	11	MLYSSSAFDLK			De-novo
631,3115	2	1260,6074	1260,6061	0,0013	1,0122	58,7302	155,4507	1	11	MLYSSSALFNK	Deamidation N	10	De-novo
883,8934	2	1765,7712	1765,7943	-0,0231	-13,0955	47,619	213,6779	1	18	AASCDEGASLLSGWGNTK			De-novo
883,8934	2	1765,7712	1765,7943	-0,0231	-13,0955	49,5238	213,5547	1	18	AACDESGASLLSGWGNTK			De-novo
883,8934	2	1765,7712	1765,7943	-0,0231	-13,0955	44,7619	214,7104	1	18	AASENGCASLLSGWGNTK	Deamidation N	5	De-novo
897,911	2	1793,8064	1793,8254	-0,0190	-10,6053	43,8095	157,5475	1	18	AASNGMDGALLSGWGNKK	Deamidation N / Oxidation	4 / 6 / 16	De-novo
897,911	2	1793,8064	1793,8254	-0,0190	-10,6053	43,4343	154,9712	1	17	NETGMDGALLSGWGNKK	Oxidation M / Deamidation	5 / 15	De-novo
897,911	2	1793,8064	1793,8254	-0,0190	-10,6053	43,4343	154,7144	1	17	ENTGMDGALLSGWGNKK	Oxidation M / Deamidation	5 / 15	De-novo
742,8491	2	1483,6826	1483,6841	-0,0015	-1,0272	66,6667	126,2604	1	12	FLLMSWCPDTAK	Oxidation M /	4 / 7	De-novo
742,8491	2	1483,6826	1483,6841	-0,0015	-1,0272	66,6667	127,3645	1	12	FLLMSWCPNTAK	Oxidation M /	4 / 7 / 9	De-novo
734,8508	2	1467,6860	1467,6891	-0,0031	-2,1285	62,3188	142,0534	1	12	FLLMSWCPNTAK	Carbamidomethyl C /	7 / 9	De-novo
734,8508	2	1467,6860	1467,6891	-0,0031	-2,1285	62,3188	140,7558	1	12	FLLMSWCPDTAK	Carbamidomethyl C	7	De-novo
734,8508	2	1467,6860	1467,6892	-0,0032	-2,1967	68,1159	140,5446	1	12	LFLMSWCPNTAK	Carbamidomethyl C /	7 / 9	De-novo
480,2709	2	958,5262	958,5276	-0,0014	-1,4856	46,1538	58,2828	1	7	YVVFYLR			De-novo
480,2709	2	958,5262	958,5276	-0,0014	-1,4856	46,1538	56,1009	1	7	YVVFLYR			De-novo

NCBI gi|170062807|ref|XP_001866830.1| conserved hypothetical protein [Culex quinquefasciatus] Total Score: 279

```
                 0         40        80        120       160
                 |         |         |         |         | |164
gi|170062807|ref|XP_0  | ________________________________________
Local hits (HSPs)      |      __ ____        ___     ___
```

Score = 96 (49.8 bits)
Identities = 12/13 (92%), Positives = 13/13 (100%)

```
Query:  217 KLFLMSWCPNTAK 229
            KLFLMSWCP+TAK
Sbjct:   88 KLFLMSWCPDTAK 100   MatchSeq
```

Score = 59 (31.3 bits)
Identities = 6/8 (75%), Positives = 8/8 (100%)

```
Query:  300 RYVVFYLR 307
            RYV+FY+R
Sbjct:   25 RYVIFYIR 32   MatchSeq
```

Score = 51 (27.3 bits)
Identities = 5/7 (71%), Positives = 7/7 (100%)

```
Query:  230 YVVFYLR 236
            YV+FY+R
Sbjct:  125 YVIFYIR 131   MatchSeq
```

Score = 37 (20.3 bits)
Identities = 4/10 (40%), Positives = 8/10 (80%)

```
Query:   11 QLNVETVANR 20
            Q++VE + +R
Sbjct:   36 QIDVEVIGDR 45   MatchSeq
```

Score = 36 (19.8 bits)
Identities = 4/5 (80%), Positives = 5/5 (100%)

```
Query:  236 RYVVF 240
            RYV+F
Sbjct:  124 RYVIF 128   MatchSeq
```

ButterflyBase PIP01950_1 Total Score: 251

```
                 0         40        80        120       160
                 |         |         |         |         |161
butterfly|PIP01950_1   | ________________________________________
Local hits (HSPs)      |         ___ ___          ____ _
```

Score = 96 (49.8 bits)
Identities = 12/13 (92%), Positives = 13/13 (100%)

```
Query:  217 KLFLMSWCPNTAK 229
            KLFLMSWCP+TAK
Sbjct:  101 KLFLMSWCPDTAK 113
```

Score = 56 (29.8 bits)
Identities = 6/7 (85%), Positives = 7/7 (100%)

```
Query:  230 YVVFYLR 236
            YVVFY+R
Sbjct:   39 YVVFYIR 45
```

Score = 51 (27.3 bits)
Identities = 7/7 (100%), Positives = 7/7 (100%)

```
Query:   41 KMLYSSS 47
            KMLYSSS
Sbjct:  117 KMLYSSS 123
```

Score = 48 (25.8 bits)
Identities = 6/10 (60%), Positives = 8/10 (80%)

```
Query:   11 QLNVETVANR 20
            Q++VETV  R
Sbjct:   49 QIDVETVGER 58
```

Ha SG EST hargln|HA-GLN_Contig_1716_FWD_frame_2 Total Score: 252

```
                 0         70        140       210       280
                 |         |         |         |         |   |308
hargln|HA-GLN_Contig_  | ________________________________________
Local hits (HSPs)      |     ___      ___
```

Score = 89 (46.3 bits)
Identities = 11/13 (84%), Positives = 12/13 (92%)

```
Query:  217 KLFLMSWCPNTAK 229
            KLFLMSWCP+T K
Sbjct:  106 KLFLMSWCPDTTK 118
```

Score = 64 (33.8 bits)
Identities = 7/8 (87%), Positives = 8/8 (100%)

```
Query:  300 RYVVFYLR 307
            RYVVFY+R
Sbjct:   43 RYVVFYIR 50
```

Score = 51 (27.3 bits)
Identities = 7/7 (100%), Positives = 7/7 (100%)

```
Query:   41 KMLYSSS 47
            KMLYSSS
Sbjct:  122 KMLYSSS 128
```

Score = 48 (25.8 bits)
Identities = 6/10 (60%), Positives = 8/10 (80%)

```
Query:   11 QLNVETVANR 20
            Q++VETV  R
Sbjct:   54 QIDVETVGER 63
```

Spot 50

m/z	Charge	Peak mW	Peptide mW	Delta (Da)	Delta (ppm)	Ladder Score	Log Likelihood	Start	End	Sequence	Modifications	Subs	Query Tool
568,7653	2	1135,5150	1135,5179	-0,0029	-2,5750	58,7302	160,7977	1	11	QAGVTVSDACK	Deamidation Q /	1 / 10	De-novo
568,7653	2	1135,5150	1135,5179	-0,0029	-2,5750	58,7302	163,0078	1	11	EAGVTVSDACK	Carbamidomethyl C	10	De-novo
568,7653	2	1135,5150	1135,5179	-0,0029	-2,5750	58,7302	158,349	1	11	AQGVTVSDACK	Deamidation Q /	2 / 10	De-novo
573,2927	2	1144,5698	1144,5724	-0,0026	-2,2926	66,6667	255,6222	1	10	QLNVETVGQR	Deamidation N /	3 / 9	De-novo
573,2927	2	1144,5698	1144,5724	-0,0026	-2,2926	66,6667	255,1168	1	10	QLNVETVGER	Deamidation N	3	De-novo
703,3562	2	1404,6968	1404,6959	0,0009	0,6236	69,5652	152,2152	1	12	MLYSSSFDALKK	Oxidation M	1	De-novo
703,3562	2	1404,6968	1404,6959	0,0009	0,6236	69,5652	149,9863	1	12	MLYSSSFNALKK	Oxidation M / Deamidation	1 / 8	De-novo
703,3562	2	1404,6968	1404,7072	-0,0104	-7,4208	60,8696	149,4775	1	12	MLYSSPHDALKK	Oxidation M	1	De-novo
469,2365	3	1404,6861	1404,7039	-0,0178	-12,6973	56,5217	156,8583	1	12	YAEPSHFDALKK			De-novo
469,2365	3	1404,6861	1404,7039	-0,0178	-12,6973	56,5217	156,0265	1	12	SFEPSHFDALKK			De-novo
806,8815	2	1611,7474	1611,7417	0,0057	3,5217	62,963	267,2747	1	14	NAEYDQFLENLGAK	Deamidation N	10	De-novo
806,8815	2	1611,7474	1611,7417	0,0057	3,5217	62,963	268,0742	1	14	NAEYNQFLENLGAK	Deamidation N	5 / 10	De-novo
806,8815	2	1611,7474	1611,7417	0,0057	3,5217	56,7901	265,2941	1	14	NAEYDQFLELNGAK	Deamidation N	11	De-novo
666,3542	2	1330,6928	1330,7034	-0,0106	-7,9837	34,9206	64,0682	1	11	VYVFKAENHPK			De-novo
666,3542	2	1330,6928	1330,7034	-0,0106	-7,9837	34,9206	63,3607	1	11	VYVFKAENPHK			De-novo
666,3542	2	1330,6928	1330,7034	-0,0106	-7,9837	41,2698	63,5882	1	11	YVVFKAENHPK			De-novo

NCBI gi|38047865|gb|AAR09835.1| similar to Drosophila melanogaster tsr — Total Score: 443

```
                 0         30        60        90        120
                 |          |         |         |         |         |148
gi|38047865|gb|AAR098   | ________________________________________________
Local hits (HSPs)       | ___   __    ______              ____

 Score = 94 (48.4 bits)
 Identities = 13/14 (92%), Positives = 14/14 (100%)

Query:    64 KKMLYSSSFNALKK 77
             KKMLYSSSF+ALKK
Sbjct:   103 KKMLYSSSFDALKK 116   MatchSeq

 Score = 77 (40.0 bits)
 Identities = 9/11 (81%), Positives = 11/11 (100%)

Query:   114 NAEYDQFLENL 124
             NAEYDQFLE++
Sbjct:    46 NAEYDQFLEDI 56   MatchSeq

 Score = 71 (37.0 bits)
 Identities = 10/15 (66%), Positives = 10/15 (66%)

Query:   136 ENLGAKNAEYDQFLE 150
             E     NAEYDQFLE
Sbjct:    40 ETVADRNAEYDQFLE 54   MatchSeq

 Score = 59 (31.1 bits)
 Identities = 7/11 (63%), Positives = 10/11 (90%)

Query:   188 KQLNVETVGER 198
             KQ++VETV +R
Sbjct:    35 KQIDVETVADR 45   MatchSeq

 Score = 57 (30.1 bits)
 Identities = 9/11 (81%), Positives = 9/11 (81%)

Query:    23 AQGVTVSDACK 33
             A GVTVSD CK
Sbjct:     2 ASGVTVSDVCK 12   MatchSeq

 Score = 48 (25.6 bits)
 Identities = 7/7 (100%), Positives = 7/7 (100%)

Query:    96 FDALKKS 102
             FDALKKS
Sbjct:   111 FDALKKS 117   MatchSeq

 Score = 37 (20.2 bits)
 Identities = 4/6 (66%), Positives = 5/6 (83%)

Query:   176 HKYVVF 181
             H YV+F
Sbjct:    24 HRYVIF 29   MatchSeq
```

ButterflyBase db|HMP01674_3|HMP01674_3 — Total Score: 403

```
                 0        30        60        90        120       150
                 |         |         |         |         |         |151
db|HMP01674_3|HMP0167   | ________________________________________________
Local hits (HSPs)       |  ___   _ ______          ____

 Score = 94 (48.4 bits)
 Identities = 13/14 (92%), Positives = 14/14 (100%)

Query:    64 KKMLYSSSFNALKK 77
             KKMLYSSSF+ALKK
Sbjct:   106 KKMLYSSSFDALKK 119

 Score = 81 (42.0 bits)
 Identities = 11/17 (64%), Positives = 13/17 (76%)

Query:   122 ENLGAKNAEYNQFLENL 138
             E  G  NAEY+QFLE+L
Sbjct:    43 ETVGERNAEYDQFLEDL 59

 Score = 73 (38.0 bits)
 Identities = 9/11 (81%), Positives = 11/11 (100%)

Query:   188 KQLNVETVGER 198
             KQ++VETVGER
Sbjct:    38 KQIDVETVGER 48

 Score = 65 (34.0 bits)
 Identities = 10/11 (90%), Positives = 10/11 (90%)

Query:    23 AQGVTVSDACK 33
             A GVTVSDACK
Sbjct:     5 ASGVTVSDACK 15

 Score = 48 (25.6 bits)
 Identities = 7/7 (100%), Positives = 7/7 (100%)

Query:    96 FDALKKS 102
             FDALKKS
Sbjct:   114 FDALKKS 120

 Score = 42 (22.6 bits)
 Identities = 5/6 (83%), Positives = 5/6 (83%)

Query:   176 HKYVVF 181
             H YVVF
Sbjct:    27 HRYVVF 32
```

Ha SG EST hargln|HA-GLN_Contig_1716_FWD_frame_2 — Total Score: 403

```
                 0          70         140        210        280
                 |           |           |          |          |    |308
hargln|HA-GLN_Contig_   | ________________________________________________
Local hits (HSPs)       |   __  ______       __

 Score = 94 (48.4 bits)
 Identities = 13/14 (92%), Positives = 14/14 (100%)

Query:    64 KKMLYSSSFNALKK 77
             KKMLYSSSF+ALKK
Sbjct:   121 KKMLYSSSFDALKK 134

 Score = 81 (42.0 bits)
 Identities = 11/17 (64%), Positives = 13/17 (76%)

Query:   122 ENLGAKNAEYNQFLENL 138
             E  G  NAEY+QFLE+L
Sbjct:    58 ETVGERNAEYDQFLEDL 74

 Score = 73 (38.0 bits)
 Identities = 9/11 (81%), Positives = 11/11 (100%)

Query:   188 KQLNVETVGER 198
             KQ++VETVGER
Sbjct:    53 KQIDVETVGER 63

 Score = 65 (34.0 bits)
 Identities = 10/11 (90%), Positives = 10/11 (90%)

Query:    23 AQGVTVSDACK 33
             A GVTVSDACK
Sbjct:    20 ASGVTVSDACK 30

 Score = 48 (25.6 bits)
 Identities = 7/7 (100%), Positives = 7/7 (100%)

Query:    96 FDALKKS 102
             FDALKKS
Sbjct:   129 FDALKKS 135

 Score = 42 (22.6 bits)
 Identities = 5/6 (83%), Positives = 5/6 (83%)

Query:   176 HKYVVF 181
             H YVVF
Sbjct:    42 HRYVVF 47
```

Spot 51

667,3138	2	1332,6120	1332,6157	-0,0037	-2,7945	56,7901	373,6277	1	14	NLGSGGVEETNAGK	Deamidation N	1	De-novo
734,3537	2	1466,6918	1466,6888	0,0030	2,0291	56,7901	157,2131	1	14	LGDGSNANDLTVYK	Deamidation N	8	De-novo
734,3537	2	1466,6918	1466,6888	0,0030	2,0291	56,7901	157,4584	1	14	LGDGSNANNLTVYK	Deamidation N	8 / 9	De-novo
734,3537	2	1466,6918	1466,6888	0,0030	2,0291	56,7901	156,517	1	14	LGDGSNADNLTVYK	Deamidation N	9	De-novo
478,7381	2	955,4606	955,4651	-0,0045	-4,7349	48,8889	132,8073	1	8	YQAADFLK	Deamidation Q	2	De-novo
620,8147	2	1239,6138	1239,6346	-0,0208	-16,7985	69,5652	304,7963	1	12	NAPASPLAELEK	Deamidation N	1	De-novo
620,8147	2	1239,6138	1239,6346	-0,0208	-16,7985	69,5652	303,5108	1	12	NAPASPLAQLEK	Deamidation N /	1 / 9	De-novo
620,8147	2	1239,6138	1239,6346	-0,0208	-16,7985	69,5652	303,7093	1	12	DAPASPLAELEK			De-novo
562,3132	2	1122,6108	1122,6073	0,0035	3,0964	70,5882	211,6156	1	9	LFTPLTEFR			De-novo
562,3132	2	1122,6108	1122,6073	0,0035	3,0964	70,5882	209,6179	1	9	LFTPLTQFR	Deamidation Q	7	De-novo
562,3132	2	1122,6108	1122,6073	0,0035	3,0964	74,5098	202,5893	1	9	LFTPLETFR			De-novo
779,9276	2	1557,8396	1557,8363	0,0033	2,1029	61,2903	219,1368	1	16	LQAALAGTTVAGETQK			De-novo
779,9276	2	1557,8396	1557,8363	0,0033	2,1029	61,2903	218,3248	1	16	LQAALAGTTVAGQTQK	Deamidation Q	13	De-novo
779,9276	2	1557,8396	1557,8363	0,0033	2,1029	62,069	215,9729	1	15	LQAALQTTVAGETQK			De-novo
844,3851	2	1686,7546	1686,9304	-0,1758	-104,2272	30,303	133,4387	1	17	ATPALASYASALVSLPR			De-novo
844,3851	2	1686,7546	1686,9304	-0,1758	-104,2272	30,303	133,3362	1	17	ATPALASYASLAVSLPR			De-novo
844,3851	2	1686,7546	1686,9304	-0,1758	-104,2272	30,303	133,1349	1	17	ATPALASFSSALVSLPR			De-novo
421,7583	2	841,5010	841,5021	-0,0011	-1,3357	68,8889	72,7063	1	8	VAVTSLPR			De-novo
421,7583	2	841,5010	841,5021	-0,0011	-1,3357	75,5556	72,929	1	8	VATVSLPR			De-novo
528,2985	2	1054,5814	1054,5771	0,0043	4,0547	47,3684	64,1061	1	10	GNPVLTEAVR			De-novo

NCBI gi|195022761|ref|XP_001985637.1| GH17180 [Drosophila grimshawi] — Total Score: 160

```
                 0        420       840       1260      1680
                 |         |         |         |         |         |2075
gi|195022761|ref|XP_0   | ________________________________________________
Local hits (HSPs)       |                 _   __

 Score = 58 (30.9 bits)
 Identities = 9/14 (64%), Positives = 11/14 (78%)

Query:   192 ATPALASYASLAVS 205
             A PA+ASYA  A+S
Sbjct:   938 AAPAIASYAAPAIS 951   MatchSeq

 Score = 52 (27.9 bits)
 Identities = 7/10 (70%), Positives = 8/10 (80%)

Query:   175 ATPALASYAS 184
             A PA+ASY S
Sbjct:   812 AAPAIASYSS 821   MatchSeq

 Score = 50 (26.9 bits)
 Identities = 7/10 (70%), Positives = 9/10 (90%)

Query:   209 ATPALASFSS 218
             A PA+AS+SS
Sbjct:   989 AAPAIASYSS 998   MatchSeq
```

ButterflyBase db|HEP01070_2|HEP01070_2 — Total Score: 158

```
                 0          50         100        150        200
                 |           |           |          |          |    |218
db|HEP01070_2|HEP0107   | ________________________________________________
Local hits (HSPs)       |                   __   _____           __

 Score = 57 (30.4 bits)
 Identities = 10/13 (76%), Positives = 10/13 (76%)

Query:   192 ATPALASYASLAV 204
             A PALASYA  AV
Sbjct:   180 AAPALASYAAPAV 192

 Score = 53 (28.4 bits)
 Identities = 8/11 (72%), Positives = 8/11 (72%)

Query:   175 ATPALASYASA 185
             A PA ASYA A
Sbjct:    81 AAPAVASYAAA 91

 Score = 48 (25.9 bits)
 Identities = 11/26 (42%), Positives = 12/26 (46%)

Query:   194 PALASYASLAVSLPRATPALASFSSA 219
             PA  SY S   S     PA AS+  A
Sbjct:   101 PASSSYSSYSSSVSHGAPAVASYAAA 126
```

Ha SG EST hargln|HA-GLN_Contig_478_FWD_frame_0 — Total Score: 158

```
                 0         90        180       270       360
                 |          |          |         |         |         |439
hargln|HA-GLN_Contig_   | ________________________________________________
Local hits (HSPs)       |                _                     ___

 Score = 85 (44.4 bits)
 Identities = 12/14 (85%), Positives = 13/14 (92%)

Query:    15 LGDGSNANDLTVYK 28
             LGD SNA+DLTVYK
Sbjct:   348 LGDXSNADDLTVYK 361

 Score = 73 (38.4 bits)
 Identities = 9/10 (90%), Positives = 10/10 (100%)

Query:   100 KLFTPLTEFR 109
             KLFTP+TEFR
Sbjct:   155 KLFTPITEFR 164
```

Spot 52

m/z	Charge	Peak mW	Peptide mW	Delta (Da)	Delta (ppm)	Ladder Score	Log Likelihood	Start	End	Sequence	Modifications	Subs	Query Tool
791,3429	2	1580,6702	1580,6704	-0,0002	-0,1417	55,914	232,6244	1	16	NLESYNANGGGGENGK	Deamidation N	1	De-novo
791,3429	2	1580,6702	1580,6704	-0,0002	-0,1417	55,914	232,204	1	16	NLESYNANGGGGQNGK	Deamidation N /	1 / 13	De-novo
791,3429	2	1580,6702	1580,6704	-0,0002	-0,1417	55,914	230,9746	1	16	NLQSYNANGGGGENGK	Deamidation N /	1 / 3	De-novo
590,3046	2	1178,5936	1178,6045	-0,0109	-9,2686	43,1373	72,6696	1	9	YEELQRSVR			De-novo
590,3046	2	1178,5936	1178,6044	-0,0108	-9,1837	54,902	70,6391	1	9	YEELKRNAR	Deamidation N	7	De-novo
883,8938	2	1765,7720	1765,812	-0,0400	-22,6661	38,0952	185,3124	1	18	SGTNETGGSLLSGWGNTK	Deamidation N	4	De-novo
883,8938	2	1765,7720	1765,812	-0,0400	-22,6661	37,1429	184,9572	1	18	GSTNETGGSLLSGWGNTK	Deamidation N	4	De-novo
883,8938	2	1765,7720	1765,812	-0,0400	-22,6661	40	184,5719	1	18	SGTNETGSGLLSGWGNTK	Deamidation N	4	De-novo
897,9103	2	1793,8050	1793,8077	-0,0027	-1,5186	52,381	152,7799	1	18	AACNAAGECLLSGWGNKK	Deamidation N	4 / 16	De-novo
897,9103	2	1793,8050	1793,8254	-0,0204	-11,3857	47,619	150,954	1	18	AACNAGDTSLLSGWGNKK	Deamidation N	4 / 16	De-novo
897,9103	2	1793,8050	1793,8077	-0,0027	-1,5186	46,4646	150,8123	1	17	AAMDNGECLLSGWGNKK	Deamidation N	15	De-novo

NCBI gi|151301198|ref|NP_001093087.1| mobility group protein 1B [Bombyx mori] — Total Score: 95

```
                 0          30          60          90
                 |           |           |          |          |119
gi|151301198|ref|NP_0   | ________________________________________________
Local hits (HSPs)       |                           _____

 Score = 95 (50.0 bits)
 Identities = 14/16 (87%), Positives = 15/16 (93%)

Query:   173 KNLESYNANGGGGENG 188
             K+LESYNANGGGGE G
Sbjct:    65 KDLESYNANGGGGEGG 80   MatchSeq
```

Ha SG EST hargln|HA-GLN_Contig_755_FWD_frame_1 — Total Score: 95

```
                 0          50          100         150         200
                 |           |           |           |           | |206
hargln|HA-GLN_Contig_   | ________________________________________________
Local hits (HSPs)       |                         ____

 Score = 95 (50.0 bits)
 Identities = 14/16 (87%), Positives = 15/16 (93%)

Query:   173 KNLESYNANGGGGENG 188
             K+LESYNANGGGGE G
Sbjct:    95 KDLESYNANGGGGEGG 110
```

Spot 53

m/z	Charge	Peak mW	Peptide mW	Delta (Da)	Delta (ppm)	Ladder Score	Log Likelihood	Start	End	Sequence	Modifications	Subs	Query Tool
576,2938	2	1150,5720	1150,573	-0,0010	-0,8900	69,8413	289,9803	1	11	LEHGAPNSAVR	Deamidation N	7	De-novo
576,2938	2	1150,5720	1150,573	-0,0010	-0,8900	69,8413	285,654	1	11	LQHGAPNSAVR	Deamidation Q /	2 / 7	De-novo
883,8928	2	1765,7700	1765,7942	-0,0242	-13,7185	49,5238	200,6673	1	18	AAENCGSSALLSGWGNTK	Deamidation N	4	De-novo
883,8928	2	1765,7700	1765,7942	-0,0242	-13,7185	49,5238	200,8419	1	18	AAENCGSASLLSGWGNTK	Deamidation N	4	De-novo
883,8928	2	1765,7700	1765,7942	-0,0242	-13,7185	46,6667	200,882	1	18	AAENCGSTGLLSGWGNTK	Deamidation N	4	De-novo
897,9108	2	1793,8060	1793,8079	-0,0019	-1,0726	50,5051	161,007	1	17	GCPETGCSLLSGWGKNK	Carbamidomethyl C /	2 / 16	De-novo
897,9108	2	1793,8060	1793,8079	-0,0019	-1,0726	48,4848	160,4595	1	17	GCPETCGSLLSGWGKNK	Carbamidomethyl C /	2 / 16	De-novo
897,9108	2	1793,8060	1793,8079	-0,0019	-1,0726	47,4747	159,6288	1	17	GPCEASCSLLSGWGKNK	Carbamidomethyl C /	3 / 16	De-novo
565,2794	2	1128,5432	1128,5411	0,0021	1,8395	58,7302	56,5004	1	11	LEDGAPNLGSR	Deamidation N	7	De-novo
565,2794	2	1128,5432	1128,541	0,0022	1,9282	56,1404	54,249	1	10	LEDGAEPNKR	Deamidation N	8	De-novo
565,2794	2	1128,5432	1128,541	0,0022	1,9282	56,1404	55,7402	1	10	LEDGAQPDKR	Deamidation Q	6	De-novo

NCBI gi|48095159|ref|XP 394370.1| PREDICTED: similar to CG5255-PA [Apis melifera] Total Score: 113

```
                 0         60        120       180       240
                 |         |         |         |         |    |263
gi|48095159|ref|XP_39  | ____________________________________________
Local hits (HSPs)      |                      ___
```

```
 Score = 59 (32.0 bits)
 Identities = 9/13 (69%), Positives = 10/13 (76%)

Query:    46 GSASLLSGWGNTK 58
             GS  +LSGWG TK
Sbjct:   142 GSDCILSGWGTTK 154   MatchSeq

 Score = 54 (29.5 bits)
 Identities = 9/16 (56%), Positives = 10/16 (62%)

Query:   119 LLSGWGKNKLEDGAPN 134
              +LSGWG  KL    PN
Sbjct:   146 ILSGWGTTKLNGNVPN 161   MatchSeq
```

ButterflyBase none

Ha SG EST hargln|HA-GLN Contig 722 FWD frame 0 Total Score: 71

```
             Length = 358
                 0         80        160       240       320
                 |         |         |         |         |    |358
hargln|HA-GLN_Contig_  | ____________________________________________
Local hits (HSPs)      |                 _
```

```
 Score = 71 (38.2 bits)
 Identities = 10/11 (90%), Positives = 11/11 (100%)

Query:     1 LEHGAPNSAVR 11
             LEHGAP+SAVR
Sbjct:   129 LEHGAPDSAVR 139
```

Spot 54

m/z	Charge	Peak mW	Peptide mW	Delta (Da)	Delta (ppm)	Ladder Score	Log Likelihood	Start	End	Sequence	Modifications	Subs	Query Tool
474,7247	2	947,4338	947,4461	-0,0123	-13,0076	71,1111	192,6002	1	8	HYSDLASR			De-novo
474,7247	2	947,4338	947,4461	-0,0123	-13,0076	71,1111	192,1008	1	8	HYSNLASR	Deamidation N	4	De-novo
474,7247	2	947,4338	947,4461	-0,0123	-13,0076	62,2222	180,465	1	8	YHSDLASR			De-novo
735,8385	2	1469,6614	1469,7111	-0,0497	-33,8325	92,5926	521,1668	1	14	NLLHGSNSVESANK	Deamidation N	7	De-novo
735,8385	2	1469,6614	1469,7111	-0,0497	-33,8325	92,5926	517,4958	1	14	NLLHGSDSVESANK			De-novo
735,8385	2	1469,6614	1469,7111	-0,0497	-33,8325	92,5926	517,6302	1	14	NLLHGSNSVQSANK	Deamidation N /	7 / 10	De-novo
513,7838	2	1025,5520	1025,5618	-0,0098	-9,5791	39,6825	106,0501	1	11	SGPAAALQVGR			De-novo
513,7838	2	1025,5520	1025,5618	-0,0098	-9,5791	39,6825	104,6394	1	11	SGAPAALQVGR			De-novo
513,7838	2	1025,5520	1025,5618	-0,0098	-9,5791	36,5079	104,233	1	11	GSPAAALQVGR			De-novo
710,8815	2	1419,7474	1419,7543	-0,0069	-4,8769	68,1159	274,2524	1	12	FTLMLKPDGVQR	Oxidation M	4	De-novo
710,8815	2	1419,7474	1419,7543	-0,0069	-4,8769	68,1159	273,5237	1	12	FTLMLKPNGVQR	Oxidation M / Deamidation	4 / 8	De-novo
710,8815	2	1419,7474	1419,7543	-0,0069	-4,8769	68,1159	267,9508	1	12	TFLMLKPDGVQR	Oxidation M	4	De-novo
871,4339	2	1740,8522	1740,8828	-0,0306	-17,5911	74,7475	398,597	1	17	QMLGATNPADSLPGTLR			De-novo
871,4339	2	1740,8522	1740,8829	-0,0307	-17,6485	76,1905	390,5514	1	18	QMLGATGGPADSLPGTLR			De-novo
871,4339	2	1740,8522	1740,8828	-0,0306	-17,5911	73,1183	379,6969	1	16	QMLQTNPADSLPGTLR			De-novo
870,9357	2	1739,8558	1739,8591	-0,0033	-1,9105	55,5556	141,4337	1	17	AYHGATNPADSLPGTLR			De-novo
870,9357	2	1739,8558	1739,8591	-0,0033	-1,9105	55,5556	141,283	1	17	YAHGATNPADSLPGTLR			De-novo
870,9357	2	1739,8558	1739,8591	-0,0033	-1,9105	55,5556	140,5654	1	17	FSHGATNPADSLPGTLR			De-novo
452,7577	2	903,4998	903,5219	-0,0221	-24,4864	64,4444	165,3505	1	8	FPFPGLVK			De-novo
452,7577	2	903,4998	903,5252	-0,0254	-28,1387	64,4444	163,9124	1	8	MLFPGLVK			De-novo
452,7577	2	903,4998	903,5252	-0,0254	-28,1387	68,8889	162,7161	1	8	LMFPGLVK			De-novo
695,3704	2	1388,7252	1388,7275	-0,0023	-1,6735	68,1159	116,3549	1	12	FVWPSKPGCLQK			De-novo
695,3704	2	1388,7252	1388,7275	-0,0023	-1,6735	65,0794	112,522	1	11	FVWPSKPCLQK	Carbamidomethyl C	8	De-novo
695,3704	2	1388,7252	1388,7275	-0,0023	-1,6735	68,1159	114,6929	1	12	VFWPSKPGCLQK			De-novo
537,6784	2	1073,3412	1073,4923	-0,1511	-140,7779	35,0877	98,599	1	10	SAMHSDLASR			De-novo
474,2565	3	1419,7461	1419,7543	-0,0082	-5,8010	56,5217	244,5499	1	12	MLFTLKPNGVQR	Oxidation M / Deamidation	1 / 8	De-novo
474,2565	3	1419,7461	1419,7543	-0,0082	-5,8010	56,5217	243,3123	1	12	MLFTLKPDGVQR	Oxidation M	1	De-novo
474,2565	3	1419,7461	1419,7832	-0,0371	-26,1561	48	247,7525	1	13	SPQPVLKPNGVQR	Deamidation N	9	De-novo
490,9109	3	1469,7093	1469,7111	-0,0018	-1,2492	59,2593	319,8299	1	14	NLLHGNSSVESANK	Deamidation N	6	De-novo
490,9109	3	1469,7093	1469,7111	-0,0018	-1,2492	59,2593	319,3146	1	14	NLLHGNSSVQSANK	Deamidation N /	6 / 10	De-novo
490,9109	3	1469,7093	1469,7111	-0,0018	-1,2492	59,2593	316,5755	1	14	NLLHGDSSVESANK			De-novo
521,3085	2	1040,6014	1040,5978	0,0036	3,4365	60,7843	80,5489	1	9	NLVGQLLER			De-novo
521,3085	2	1040,6014	1040,5978	0,0036	3,4365	60,7843	79,3485	1	9	NLVGQLLQR	Deamidation Q	8	De-novo
521,3085	2	1040,6014	1040,5978	0,0036	3,4365	54,386	81,3627	1	10	GGLVGQLLER			De-novo

NCBI gi|21435082|gb|AAM53644.1| abnormal wing disc-like protein [Choristoneura Total Score: 574

```
                 0         40        80        120
                 |         |         |         |         |153
gi|21435082|gb|AAM536  | ______________________________________
Local hits (HSPs)      |  _____   ______ __     ___  _____
```

```
 Score = 126 (65.7 bits)
 Identities = 17/18 (94%), Positives = 18/18 (100%)

Query:   455 RQMLGATNPADSLPGTLR 472
             RQMLGATNPADSLPGT+R
Sbjct:    89 RQMLGATNPADSLPGTIR 106   MatchSeq

 Score = 100 (52.5 bits)
 Identities = 12/17 (70%), Positives = 16/17 (94%)

Query:    24 RNLLHGSNSVESANKNL 40
             RN++HGS+SVESANK +
Sbjct:   115 RNIIHGSDSVESANKEI 131   MatchSeq

 Score = 98 (51.5 bits)
 Identities = 12/14 (85%), Positives = 14/14 (100%)

Query:   122 QRTFLMLKPDGVQR 135
             +RTFLM+KPDGVQR
Sbjct:     6 ERTFLMIKPDGVQR 19   MatchSeq

 Score = 64 (34.2 bits)
 Identities = 9/13 (69%), Positives = 11/13 (84%)

Query:   409 QLLERHYSDLASR 421
             +LL+ HY DLASR
Sbjct:    47 ELLQKHYADLASR 59   MatchSeq

 Score = 53 (28.7 bits)
 Identities = 6/10 (60%), Positives = 9/10 (90%)

Query:   336 LKPNGVQRNL 345
             +KP+GVQR +
Sbjct:    12 IKPDGVQRGI 21   MatchSeq

 Score = 46 (25.1 bits)
 Identities = 7/8 (87%), Positives = 7/8 (87%)

Query:   248 FPGLVKLM 255
             FPGLVK M
Sbjct:    62 FPGLVKYM 69   MatchSeq

 Score = 45 (24.6 bits)
 Identities = 5/9 (55%), Positives = 8/9 (88%)

Query:   405 GLVGQLLER 413
             G+VG ++ER
Sbjct:    20 GIVGTIIER 28   MatchSeq

 Score = 42 (23.1 bits)
 Identities = 5/6 (83%), Positives = 5/6 (83%)

Query:   261 KFVWPS 266
             KF WPS
Sbjct:    40 KFMWPS 45   MatchSeq
```

Ha SG EST hargln|HA-GLN Contig 418 FWD frame 0 Total Score: 601

```
                 0         50        100       150       200
                 |         |         |         |         |    |218
hargln|HA-GLN_Contig_  | ______________________________________
Local hits (HSPs)      |     _____  _____   ___  ___
```

```
 Score = 126 (65.7 bits)
 Identities = 17/18 (94%), Positives = 18/18 (100%)

Query:   455 RQMLGATNPADSLPGTLR 472
             RQMLGATNPADSLPGT+R
Sbjct:   119 RQMLGATNPADSLPGTIR 136

 Score = 100 (52.5 bits)
 Identities = 12/17 (70%), Positives = 16/17 (94%)

Query:    24 RNLLHGSNSVESANKNL 40
             RN++HGS+SVESANK +
Sbjct:   145 RNIIHGSDSVESANKEI 161

 Score = 98 (51.5 bits)
 Identities = 11/14 (78%), Positives = 14/14 (100%)

Query:   122 QRTFLMLKPDGVQR 135
             +RTF+M+KPDGVQR
Sbjct:    36 ERTFIMIKPDGVQR 49

 Score = 70 (37.3 bits)
 Identities = 10/13 (76%), Positives = 12/13 (92%)

Query:   409 QLLERHYSDLASR 421
             +LL+ HYSDLASR
Sbjct:    77 ELLQKHYSDLASR 89

 Score = 58 (31.2 bits)
 Identities = 7/9 (77%), Positives = 9/9 (100%)

Query:   405 GLVGQLLER 413
             GLVGQ++ER
Sbjct:    50 GLVGQIIER 58

 Score = 53 (28.7 bits)
 Identities = 7/10 (70%), Positives = 9/10 (90%)

Query:   336 LKPNGVQRNL 345
             +KP+GVQR L
Sbjct:    42 IKPDGVQRGL 51

 Score = 50 (27.1 bits)
 Identities = 6/6 (100%), Positives = 6/6 (100%)

Query:   261 KFVWPS 266
             KFVWPS
Sbjct:    70 KFVWPS 75

 Score = 46 (25.1 bits)
 Identities = 7/8 (87%), Positives = 7/8 (87%)

Query:   248 FPGLVKLM 255
             FPGLVK M
Sbjct:    92 FPGLVKYM 99
```

Spot 55

m/z	Charge	Peak mW	Peptide mW	Delta (Da)	Delta (ppm)	Ladder Score	Log Likelihood	Start	End	Sequence	Modifications	Subs	Query Tool
674,8025	2	1347,5894	1347,5878	0,0016	1,1695	68,254	102,0512	1	11	YTQSNSVCYAR	Carbamidomethyl C	8	De-novo
674,8025	2	1347,5894	1347,5878	0,0016	1,1695	60,8696	101,7416	1	12	YTAGSNSVCYAR	Carbamidomethyl C	9	De-novo
674,8025	2	1347,5894	1347,5878	0,0016	1,1695	55,5556	99,6914	1	11	YTQSNSVFSCR	Carbamidomethyl C	10	De-novo
523,7819	2	1045,5482	1045,5153	0,0329	31,4448	63,1579	104,6527	1	10	NSSPATNLSR			De-novo
535,7808	2	1069,5460	1069,5444	0,0016	1,4735	62,7451	154,1055	1	9	QTPAYSYLK			De-novo
535,7808	2	1069,5460	1069,5444	0,0016	1,4735	66,6667	153,7304	1	9	TQPAYSYLK			De-novo
650,8492	2	1299,6828	1299,6857	-0,0029	-2,2498	58,6667	218,504	1	13	NPAAGAVLMLAEK	Oxidation M	9	De-novo
883,8944	2	1765,7732	1765,7732	0,0000	-0,0136	52,6882	94,4814	1	16	EQTWECGALSGWGNTK			De-novo
861,3818	2	1720,7480	1720,7476	0,0004	0,2185	65,4321	174,5223	1	14	TPSCNQWQTEVTNR	Carbamidomethyl C /	4 / 5	De-novo
861,3818	2	1720,7480	1720,7476	0,0004	0,2185	65,4321	172,3755	1	14	TPSCDQWQTEVTNR	Carbamidomethyl C	4	De-novo
861,3818	2	1720,7480	1720,7476	0,0004	0,2185	64,1975	173,1481	1	14	GLDCNQWQTEVTNR	Carbamidomethyl C /	4 / 5	De-novo

NCBI gi|190656549|gb|EDV53781.1| GG11332 [Drosophila erecta] Total Score: 86

```
                 0        120       240       360       480       540
                          |         |         |         |         |       |590
gi|190656549|gb|EDV53  | ______________________________________________
Local hits (HSPs)      |                             _
```

```
 Score = 86 (44.7 bits)
 Identities = 11/11 (100%), Positives = 11/11 (100%)

Query:     1 YTQSNSVCYAR 11
             YTQSNSVCYAR
Sbjct:   425 YTQSNSVCYAR 435   MatchSeq
```

ButterflyBase PIP04233 1 Total Score: 78

```
                 0         40        80        120       160
                 |         |         |         |         |      |189
butterfly|PIP04233_1   | ______________________________________
Local hits (HSPs)      |       _____
```

```
 Score = 78 (40.7 bits)
 Identities = 10/10 (100%), Positives = 10/10 (100%)

Query:     1 YTQSNSVCYA 10
             YTQSNSVCYA
Sbjct:    36 YTQSNSVCYA 45
```

Ha SG EST hargln|HA-GLN Contig 5386 FWD frame 2 Total Score: 92

```
                 0         50        100       150       200      217
                 |         |         |         |         |
hargln|HA-GLN_Contig_  | ______________________________________________
Local hits (HSPs)      |                  ___
```

```
 Score = 92 (47.7 bits)
 Identities = 11/11 (100%), Positives = 11/11 (100%)

Query:   111 CDQWQTEVTNR 121
             CDQWQTEVTNR
Sbjct:    98 CDQWQTEVTNR 108
```

Spot 56

m/z	Charge	Peak mW	Peptide mW	Delta (Da)	Delta (ppm)	Ladder Score	Log Likelihood	Start	End	Sequence	Modifications	Subs	Query Tool
836,8768	2	1671,7380	1671,7378	0,0002	0,1053	64,3678	324,163	1	15	YNGGVEANLDNYTNK	Deamidation N	2	De-novo
836,8768	2	1671,7380	1671,7378	0,0002	0,1053	61,7284	322,6485	1	14	YNNVEANLDNYTNK	Deamidation N	2	De-novo
836,8768	2	1671,7380	1671,7378	0,0002	0,1053	60,2151	325,1017	1	16	YNGGVEANLDNYTGGK	Deamidation N	2	De-novo
818,3792	2	1634,7428	1634,7433	-0,0005	-0,3205	62,963	177,6951	1	14	YPNVMSVVDPMNPR	Deamidation N / Oxidation	3 / 5	De-novo
818,3792	2	1634,7428	1634,7433	-0,0005	-0,3205	62,963	177,6736	1	14	YPNVMSVVNPMNPR	Deamidation N / Oxidation	3 / 5 / 9	De-novo
818,3792	2	1634,7428	1634,7433	-0,0005	-0,3205	55,5556	175,3746	1	14	YPNVMSVVDPNMPR	Deamidation N / Oxidation	3 / 5	De-novo
520,282	2	1038,5484	1038,5498	-0,0014	-1,3711	54,902	138,0669	1	9	YSPTGFVLR			De-novo
520,282	2	1038,5484	1038,5498	-0,0014	-1,3711	56,8627	137,8478	1	9	SYPTGFVLR			De-novo
520,282	2	1038,5484	1038,5498	-0,0014	-1,3711	43,1373	123,8971	1	9	YSPTGFLVR			De-novo
542,3091	2	1082,6026	1082,6012	0,0014	1,2710	54,902	142,7049	1	9	NYLVLVAYK	Deamidation N	1	De-novo
542,3091	2	1082,6026	1082,6012	0,0014	1,2710	54,902	142,7637	1	9	YNLVLVAYK	Deamidation N	2	De-novo
542,3091	2	1082,6026	1082,6012	0,0014	1,2710	74,5098	140,0249	1	9	LVDYLVAYK			De-novo
650,8193	2	1299,6230	1299,6248	-0,0018	-1,4035	63,1579	108,7269	1	10	NPAFYQLYQR	Deamidation N	1	De-novo
650,8193	2	1299,6230	1299,6248	-0,0018	-1,4035	63,1579	109,4027	1	10	PNAFYQLYQR	Deamidation N	2	De-novo
549,3157	2	1096,6158	1096,6168	-0,0010	-0,9338	49,0196	127,7944	1	9	NYLVLLAYK	Deamidation N	1	De-novo
549,3157	2	1096,6158	1096,6168	-0,0010	-0,9338	49,0196	127,8776	1	9	YNLVLLAYK	Deamidation N	2	De-novo
549,3157	2	1096,6158	1096,6168	-0,0010	-0,9338	49,0196	123,2416	1	9	DYLVLLAYK			De-novo
481,2416	2	960,4676	960,4706	-0,0030	-3,1485	64,1026	111,9437	1	7	FSLFYER			De-novo
481,2416	2	960,4676	960,4706	-0,0030	-3,1485	64,1026	111,0846	1	7	FSLFYQR	Deamidation Q	6	De-novo
481,2416	2	960,4676	960,4706	-0,0030	-3,1485	48,7179	106,4233	1	7	FLSFYER			De-novo
697,8742	2	1393,7328	1393,7355	-0,0027	-1,9545	79,3651	174,0322	1	11	QAVQQFLELYR			De-novo
563,8145	2	1125,6134	1125,6182	-0,0048	-4,2856	50,8772	59,883	1	10	LAPGENWVLK			De-novo
563,8145	2	1125,6134	1125,6029	0,0105	9,3070	42,8571	60,9871	1	11	LAPGEANNVLK	Deamidation N	8	De-novo
563,8145	2	1125,6134	1125,6029	0,0105	9,3070	49,2063	60,8735	1	11	LAPGEANNLVK	Deamidation N	8	De-novo
504,3002	1	503,2924	503,3108	-0,0184	-36,5818	61,9048	83,5798	1	4	FLPK			De-novo
548,2968	2	1094,5780	1094,576	0,0020	1,8053	60,7843	108,9527	1	9	AYPTEFLVR			De-novo
548,2968	2	1094,5780	1094,576	0,0020	1,8053	60,7843	108,6009	1	9	YAPTEFLVR			De-novo

NCBI gi|5869989|emb|CAB55605.1| arylphorin subunit [Spodoptera litura] Total Score: 355

```
                        0       140      280      420      560
                        |        |        |        |        |       |698
gi|5869989|emb|CAB556   | ______________________________________________
Local hits (HSPs)       |   ___                   ___   _
```

```
 Score = 74 (38.0 bits)
 Identities = 9/10 (90%), Positives = 10/10 (100%)

Query:   142 NPAFYQLYQR 151
             +PAFYQLYQR
Sbjct:   417 DPAFYQLYQR 426   MatchSeq
```

```
 Score = 72 (37.0 bits)
 Identities = 8/9 (88%), Positives = 9/9 (100%)

Query:   187 YKFSLFYER 195
             YKFS+FYER
Sbjct:    95 YKFSIFYER 103   MatchSeq
```

```
 Score = 62 (32.1 bits)
 Identities = 7/12 (58%), Positives = 10/12 (83%)

Query:    18 NVEANLDNYTNK 29
             ++EAN+ NY NK
Sbjct:    65 DIEANIQNYSNK 76   MatchSeq
```

```
 Score = 53 (27.7 bits)
 Identities = 8/11 (72%), Positives = 10/11 (90%)

Query:   210 QAVQQFLELYR 220
             QAV++FL LYR
Sbjct:    77 QAVEEFLLLYR 87   MatchSeq
```

```
 Score = 48 (25.3 bits)
 Identities = 6/10 (60%), Positives = 8/10 (80%)

Query:   256 KAYPTEFLVR 265
             KAYP  ++VR
Sbjct:   484 KAYPVNYIVR 493   MatchSeq
```

```
 Score = 46 (24.3 bits)
 Identities = 5/9 (55%), Positives = 8/9 (88%)

Query:   133 LVDYLVAYK 141
             ++DYL+ YK
Sbjct:   427 IIDYLIDYK 435   MatchSeq
```

Ha SG EST none

Ha EST other harmi_fn|HA-FN-ALL_Contig 630 FWD frame 0 Total Score: 335

```
                        0       140      280      420      560
                        |        |        |        |        |       |666
harmi_fn|HA-FN-ALL_Co   | ______________________________________________
Local hits (HSPs)       |                           _   _   __
```

```
 Score = 75 (38.5 bits)
 Identities = 9/10 (90%), Positives = 10/10 (100%)

Query:   256 KAYPTEFLVR 265
             KAYPTEF+VR
Sbjct:   491 KAYPTEFIVR 500
```

```
 Score = 74 (38.0 bits)
 Identities = 9/10 (90%), Positives = 10/10 (100%)

Query:   142 NPAFYQLYQR 151
             +PAFYQLYQR
Sbjct:   424 DPAFYQLYQR 433
```

```
 Score = 65 (33.6 bits)
 Identities = 8/9 (88%), Positives = 9/9 (100%)

Query:   133 LVDYLVAYK 141
             +VDYLVAYK
Sbjct:   434 IVDYLVAYK 442
```

```
 Score = 41 (21.9 bits)
 Identities = 6/6 (100%), Positives = 6/6 (100%)

Query:   221 LAPGEN 226
             LAPGEN
Sbjct:   559 LAPGEN 564
```

```
 Score = 40 (21.4 bits)
 Identities = 5/7 (71%), Positives = 6/7 (85%)

Query:   175 LLAYKDY 181
             L AYK+Y
Sbjct:   438 LVAYKEY 444
```

```
 Score = 40 (21.4 bits)
 Identities = 6/6 (100%), Positives = 6/6 (100%)

Query:   230 KLAPGE 235
             KLAPGE
Sbjct:   558 KLAPGE 563
```

Spot 57

m/z	Charge	Peak mW	Peptide mW	Delta (Da)	Delta (ppm)	Ladder Score	Log Likelihood	Start	End	Sequence	Modifications	Subs	Query Tool
505,7432	2	1009,4708	1009,4717	-0,0009	-0,9153	52,6316	119,1841	1	10	SADVSNAAFK	Deamidation N	6	De-novo
505,7432	2	1009,4708	1009,4716	-0,0008	-0,8163	52,6316	119,2252	1	10	SANVSDAAFK	Deamidation N	3	De-novo
599,797	2	1197,5784	1197,5779	0,0005	0,3975	47,619	105,5619	1	11	AGSPAYSYLNR			De-novo
599,797	2	1197,5784	1197,5779	0,0005	0,3975	46,0317	103,9197	1	11	GASPAYSYLNR			De-novo
599,797	2	1197,5784	1197,5779	0,0005	0,3975	50,8772	100,6407	1	10	QSPAYSYLNR			De-novo
744,3548	2	1486,6940	1486,6576	0,0364	24,4683	53,0864	82,7372	1	14	SNPSYVASNSFGQK	Deamidation N /	2 / 13	De-novo
744,3548	2	1486,6940	1486,694	0,0000	-0,0161	55,5556	82,076	1	14	TTPSYVASNSFGQK	Deamidation Q	13	De-novo
744,3548	2	1486,6940	1486,6576	0,0364	24,4683	48,1481	81,6447	1	14	SNPSYVASNSGFQK	Deamidation N /	2 / 13	De-novo
635,7918	2	1269,5680	1269,5699	-0,0019	-1,5155	64,9123	154,166	1	10	QPNMYFENVK	Deamidation N	8	De-novo
635,7918	2	1269,5680	1269,5699	-0,0019	-1,5155	64,9123	153,1894	1	10	QPNMYFEDVK			De-novo
520,2843	2	1038,5530	1038,5498	0,0032	3,0581	54,902	162,8875	1	9	YSPTGFVLR			De-novo
520,2843	2	1038,5530	1038,5498	0,0032	3,0581	54,902	162,7185	1	9	SYPTGFVLR			De-novo
650,8215	2	1299,6274	1299,6248	0,0026	1,9821	64,9123	131,5572	1	10	NPAFYQLYQR	Deamidation N	1	De-novo
650,8215	2	1299,6274	1299,6248	0,0026	1,9821	68,4211	129,9423	1	10	PNAFYQLYQR	Deamidation N	2	De-novo
650,8215	2	1299,6274	1299,6248	0,0026	1,9821	52,6316	128,7343	1	10	NPAFYQLQYR	Deamidation N	1	De-novo
549,3179	2	1096,6202	1096,6168	0,0034	3,0786	50,9804	123,5658	1	9	NYVLLLAYK	Deamidation N	1	De-novo
549,3179	2	1096,6202	1096,6168	0,0034	3,0786	54,902	123,6038	1	9	YNVLLLAYK	Deamidation N	2	De-novo
549,3179	2	1096,6202	1096,6168	0,0034	3,0786	50,9804	123,2555	1	9	NYLVLLAYK	Deamidation N	1	De-novo
481,2441	2	960,4726	960,4706	0,0020	2,0573	79,4872	113,1159	1	7	FSLFYER			De-novo
481,2441	2	960,4726	960,4706	0,0020	2,0573	79,4872	111,7339	1	7	FSLFYQR	Deamidation Q	6	De-novo
548,2967	2	1094,5778	1094,576	0,0018	1,6225	56,8627	166,2813	1	9	AYPTEFLVR			De-novo
548,2967	2	1094,5778	1094,576	0,0018	1,6225	60,7843	165,9212	1	9	YAPTEFLVR			De-novo
548,2967	2	1094,5778	1094,576	0,0018	1,6225	56,8627	165,1016	1	9	AYPTQFLVR	Deamidation Q	5	De-novo
486,7293	2	971,4430	971,4388	0,0042	4,2988	48,7179	85,2188	1	7	FNETFWK	Deamidation N	2	De-novo
486,7293	2	971,4430	971,4388	0,0042	4,2988	48,7179	83,8632	1	7	NFETFWK	Deamidation N	1	De-novo
486,7293	2	971,4430	971,4388	0,0042	4,2988	48,7179	83,9223	1	7	FDETFWK			De-novo
563,8129	2	1125,6102	1125,6143	-0,0041	-3,6638	43,8596	48,4172	1	10	LAPGENKQLR	Deamidation Q	8	De-novo
563,8129	2	1125,6102	1125,6143	-0,0041	-3,6638	43,8596	47,4776	1	10	LAPGENKLER			De-novo
563,8129	2	1125,6102	1125,6143	-0,0041	-3,6638	43,8596	46,7849	1	10	LAPGENKELR			De-novo

NCBI gi|5869989|emb|CAB55605.1| arylphorin subunit [Spodoptera litura] Total Score: 416

```
                        0       140      280      420      560
                        |        |        |        |        |       |698
gi|5869989|emb|CAB556   | ______________________________________________
Local hits (HSPs)       |     __                    ___   _   ___
```

```
 Score = 87 (44.7 bits)
 Identities = 11/12 (91%), Positives = 12/12 (100%)

Query:   131 LRNPAFYQLYQR 142
             LR+PAFYQLYQR
Sbjct:   415 LRDPAFYQLYQR 426   MatchSeq
```

```
 Score = 72 (37.3 bits)
 Identities = 8/9 (88%), Positives = 9/9 (100%)

Query:   188 YKFSLFYER 196
             YKFS+FYER
Sbjct:    95 YKFSIFYER 103   MatchSeq
```

```
 Score = 64 (33.3 bits)
 Identities = 10/14 (71%), Positives = 11/14 (78%)

Query:   146 FYQLYQRNPAFYQL 159
             FYQ   R+PAFYQL
Sbjct:   410 FYQTSLRDPAFYQL 423   MatchSeq
```

```
 Score = 61 (31.9 bits)
 Identities = 8/10 (80%), Positives = 9/10 (90%)

Query:   262 LAPGENKLER 271
             L PGENK+ER
Sbjct:   552 LVPGENKIER 561   MatchSeq
```

```
 Score = 47 (25.0 bits)
 Identities = 7/8 (87%), Positives = 7/8 (87%)

Query:   251 KLAPGENK 258
             KL PGENK
Sbjct:   551 KLVPGENK 558   MatchSeq
```

```
 Score = 44 (23.5 bits)
 Identities = 6/8 (75%), Positives = 7/8 (87%)

Query:   237 KNFETFWK 244
             K+FETF K
Sbjct:   119 KDFETFYK 126   MatchSeq
```

```
 Score = 41 (22.0 bits)
 Identities = 5/9 (55%), Positives = 7/9 (77%)

Query:   204 AYPTEFLVR 212
             AYP  ++VR
Sbjct:   485 AYPVNYIVR 493   MatchSeq
```

Ha SG EST hargln|Contig 1773 FWD frame 2 Total Score: 83

```
                        0       150      300      450      600
                        |        |        |        |        |      |701
hargln|Contig_1773_FW   | ______________________________________________
Local hits (HSPs)       |               _
```

```
 Score = 83 (42.7 bits)
 Identities = 11/11 (100%), Positives = 11/11 (100%)

Query:    42 RQSPAYSYLNR 52
             RQSPAYSYLNR
Sbjct:   257 RQSPAYSYLNR 267
```

Spot 58

m/z	Charge	Peak mW	Peptide mW	Delta (Da)	Delta (ppm)	Ladder Score	Log Likelihood	Start	End	Sequence	Modifications	Subs	Query Tool
556,7626	2	1111,5096	1111,5081	0,0015	1,3279	56,8627	99,5773	1	9	YASLCQTNR	Carbamidomethyl C	5	De-novo
556,7626	2	1111,5096	1111,5081	0,0015	1,3279	58,8235	100,8696	1	9	YASLCNTQR	Carbamidomethyl C	5	De-novo
556,7626	2	1111,5096	1111,5081	0,0015	1,3279	56,8627	98,4055	1	9	YASLQTNCR	Carbamidomethyl C	8	De-novo
511,2571	2	1020,4986	1020,4988	-0,0002	-0,2195	56,8627	71,6685	1	9	YASLSSTHR			De-novo
511,2571	2	1020,4986	1020,4988	-0,0002	-0,2195	49,0196	71,3232	1	9	YASLSNNPR			De-novo
511,2571	2	1020,4986	1020,4988	-0,0002	-0,2195	49,0196	70,8777	1	9	YASLSPNNR			De-novo
535,2535	2	1068,4914	1068,491	0,0004	0,3519	62,7451	158,9872	1	9	TYTPMDLGR	Oxidation M	5	De-novo
683,8201	2	1365,6246	1365,6447	-0,0201	-14,7359	67,9012	230,9902	1	14	VALAANESTGTMGK	Deamidation N / Oxidation	6 / 12	De-novo
683,8201	2	1365,6246	1365,6447	-0,0201	-14,7359	65,4321	229,1506	1	14	LGLAANESTGTMGK	Deamidation N / Oxidation	6 / 12	De-novo
683,8201	2	1365,6246	1365,6447	-0,0201	-14,7359	67,9012	229,7372	1	14	VALAADESTGTMGK	Oxidation M	12	
675,8348	2	1349,6540	1349,6498	0,0042	3,0941	64,1975	193,7115	1	14	GLLAANESTGTMGK	Deamidation N	6	De-novo
675,8348	2	1349,6540	1349,6498	0,0042	3,0941	64,1975	193,411	1	14	LGLAANESTGTMGK	Deamidation N	6	De-novo
675,8348	2	1349,6540	1349,6498	0,0042	3,0941	64,1975	193,5447	1	14	AVLAANESTGTMGK	Deamidation N	6	De-novo
527,2564	2	1052,4972	1052,4961	0,0011	1,0223	66,6667	109,4291	1	9	TYTPMDLGR			De-novo
527,2564	2	1052,4972	1052,4961	0,0011	1,0223	66,6667	110,5254	1	9	TYTPMNLGR	Deamidation N	6	De-novo
527,2564	2	1052,4972	1052,4961	0,0011	1,0223	62,7451	105,5191	1	9	YTTPMDLGR			De-novo
661,3247	2	1320,6338	1320,6561	-0,0223	-16,9037	54,6667	250,651	1	13	GSAEYLPSLAADK			De-novo
661,3247	2	1320,6338	1320,6561	-0,0223	-16,9037	54,6667	250,2162	1	13	SGAEYLPSLAADK			De-novo
422,7653	2	843,5150	843,5178	-0,0028	-3,3479	62,2222	129,4667	1	8	ATVTALLR			De-novo
422,7653	2	843,5150	843,5178	-0,0028	-3,3479	55,5556	123,1505	1	8	TAVTALLR			De-novo
897,9103	2	1793,8050	1793,8542	-0,0492	-27,4404	43,0108	48,9474	1	16	LPTYAEDDLSGSRSQR			De-novo
897,9103	2	1793,8050	1793,8542	-0,0492	-27,4404	40,8602	48,798	1	16	YTLPAEDDLSGSRSQR			De-novo
1086,0356	2	2170,0556	2170,0583	-0,0027	-1,2553	54,2857	212,2836	1	18	TEYFGYAPTPELQEKELR			De-novo
1086,0356	2	2170,0556	2170,0583	-0,0027	-1,2553	54,2857	212,96	1	18	TEYFGYAPTPELQEKQLR	Deamidation Q	16	De-novo
1086,0356	2	2170,0556	2170,0583	-0,0027	-1,2553	54,2857	211,6525	1	18	TYEFGYAPTPELQEKELR			De-novo
671,3675	2	1340,7194	1340,7188	0,0006	0,4296	70,6667	271,2942	1	13	DADGTPLVALLEK			De-novo
671,3675	2	1340,7194	1340,7188	0,0006	0,4296	70,6667	270,6537	1	13	ADDGTPLVALLEK			De-novo
671,3675	2	1340,7194	1340,7188	0,0006	0,4296	70,6667	270,2068	1	13	GEDGTPLVALLEK			De-novo
758,869	2	1515,7224	1515,7164	0,0060	3,9427	68	96,6582	1	13	LQNLGVENTEENR	Deamidation N	3	De-novo
758,869	2	1515,7224	1515,71	0,0124	8,1652	60,4938	100,849	1	14	LQNLGVENCGQANR	Deamidation N	3	De-novo
758,869	2	1515,7224	1515,7166	0,0058	3,8107	65,3333	95,2358	1	13	LQNLGVESQEENR	Deamidation N	3	De-novo
673,3489	2	1344,6822	1344,7435	-0,0613	-45,6027	37,6812	113,147	1	12	TVSLCLSLSLPR	Carbamidomethyl C	5	De-novo
673,3489	2	1344,6822	1344,7401	-0,0579	-43,0745	37,6812	112,077	1	12	TVSLWSSLSLPR			De-novo
673,3489	2	1344,6822	1344,7435	-0,0613	-45,6027	37,6812	111,5744	1	12	SLSLCLTVSLPR	Carbamidomethyl C	5	De-novo

NCBI gi|45330818|dbj|BAD12426.1| fructose 1,6-bisphosphate aldolase [Antheraea yamamai] Total Score: 660

```
                  0        80       160       240       320
                  |         |         |         |         |      |364
gi|45330818|dbj|BAD12  | ____________________________________________
Local hits (HSPs)      | _  ___     __           _       __          _
```

```
 Score = 101 (52.1 bits)
 Identities = 14/16 (87%), Positives = 16/16 (100%)

Query:   104 GKGLLAANESTGTMGK 119
             GKG+LAA+ESTGTMGK
Sbjct:    27 GKGILAADESTGTMGK 42   MatchSeq

 Score = 88 (45.6 bits)
 Identities = 11/13 (84%), Positives = 13/13 (100%)

Query:   342 LQNLGVENTEENR 354
             LQ++GVENTEENR
Sbjct:    44 LQDIGVENTEENR 56   MatchSeq

 Score = 81 (42.1 bits)
 Identities = 12/15 (80%), Positives = 13/15 (86%)

Query:   314 EKADDGTPLVALLEK 328
             +K DDGTPLV LLEK
Sbjct:    86 QKTDDGTPLVSLLEK 100   MatchSeq

 Score = 69 (36.2 bits)
 Identities = 8/10 (80%), Positives = 9/10 (90%)

Query:   147 KTYTPMDLGR 156
             KTYTPMD+ R
Sbjct:   242 KTYTPMDIAR 251   MatchSeq

 Score = 64 (33.7 bits)
 Identities = 8/14 (57%), Positives = 9/14 (64%)

Query:    55 TYTPMDLGRVALAA 68
             TYTPMD+ R    A
Sbjct:   243 TYTPMDIARATVTA 256   MatchSeq

 Score = 58 (30.7 bits)
 Identities = 9/9 (100%), Positives = 9/9 (100%)

Query:   201 ATVTALLRT 209
             ATVTALLRT
Sbjct:   252 ATVTALLRT 260   MatchSeq

 Score = 58 (30.7 bits)
 Identities = 7/10 (70%), Positives = 8/10 (80%)

Query:     9 RYASLCNTQR 18
             RYAS+C  QR
Sbjct:   173 RYASICQSQR 182   MatchSeq

 Score = 52 (27.7 bits)
 Identities = 7/7 (100%), Positives = 7/7 (100%)

Query:   256 PTPELQE 262
             PTPELQE
Sbjct:     8 PTPELQE 14   MatchSeq

 Score = 45 (24.2 bits)
 Identities = 7/9 (77%), Positives = 8/9 (88%)

Query:   180 LPSLAADKS 188
             +PSLAA KS
Sbjct:   348 IPSLAASKS 356   MatchSeq

 Score = 44 (23.7 bits)
 Identities = 7/9 (77%), Positives = 7/9 (77%)

Query:   208 RTAVTALLR 216
             R  VTALLR
Sbjct:   251 RATVTALLR 259   MatchSeq
```

Ha SG EST none

Ha EST other harmi_rn|HA-RN-ALL_Contig_24 FWD frame 2 Total Score: 687

```
                  0        100       200       300       400
                  |         |         |         |         |      |452
harmi_rn|HA-RN-ALL_Co  | ____________________________________________
Local hits (HSPs)      |  _ ___    __       __     __
```

```
 Score = 94 (48.6 bits)
 Identities = 14/15 (93%), Positives = 15/15 (100%)

Query:   314 EKADDGTPLVALLEK 328
             +KADDGTPLVALLEK
Sbjct:   107 QKADDGTPLVALLEK 121

 Score = 90 (46.6 bits)
 Identities = 13/16 (81%), Positives = 15/16 (93%)

Query:   104 GKGLLAANESTGTMGK 119
             GKG+LAA+ESTGT GK
Sbjct:    48 GKGILAADESTGTXGK 63

 Score = 80 (41.6 bits)
 Identities = 10/13 (76%), Positives = 12/13 (92%)

Query:   342 LQNLGVENTEENR 354
             LQ++GVENTE NR
Sbjct:    65 LQDIGVENTEXNR 77

 Score = 77 (40.1 bits)
 Identities = 9/10 (90%), Positives = 10/10 (100%)

Query:   147 KTYTPMDLGR 156
             KTYTPMD+GR
Sbjct:   263 KTYTPMDIGR 272

 Score = 72 (37.7 bits)
 Identities = 9/14 (64%), Positives = 10/14 (71%)

Query:    55 TYTPMDLGRVALAA 68
             TYTPMD+GR    A
Sbjct:   264 TYTPMDIGRATVTA 277

 Score = 59 (31.2 bits)
 Identities = 8/13 (61%), Positives = 10/13 (76%)

Query:   350 TEENRLQNLGVEN 362
             T   RLQ++GVEN
Sbjct:    60 TXGKRLQDIGVEN 72

 Score = 58 (30.7 bits)
 Identities = 9/9 (100%), Positives = 9/9 (100%)

Query:   201 ATVTALLRT 209
             ATVTALLRT
Sbjct:   273 ATVTALLRT 281

 Score = 57 (30.2 bits)
 Identities = 9/10 (90%), Positives = 9/10 (90%)

Query:   457 GKATVTALLR 466
             G ATVTALLR
Sbjct:   271 GRATVTALLR 280

 Score = 52 (27.7 bits)
 Identities = 7/7 (100%), Positives = 7/7 (100%)

Query:   256 PTPELQE 262
             PTPELQE
Sbjct:    29 PTPELQE 35

 Score = 48 (25.7 bits)
 Identities = 6/10 (60%), Positives = 7/10 (70%)

Query:     9 RYASLCNTQR 18
             R AS+C  QR
Sbjct:   194 RXASICQSQR 203
```

Spot 59

m/z	Charge	Peak mW	Peptide mW	Delta (Da)	Delta (ppm)	Ladder Score	Log Likelihood	Start	End	Sequence	Modifications	Subs	Query Tool
734,3528	2	1466,6900	1466,6888	0,0012	0,8018	77,7778	206,8026	1	14	LGDGSNANDLTVYK	Deamidation N	8	De-novo
734,3528	2	1466,6900	1466,6888	0,0012	0,8018	77,7778	207,5018	1	14	LGDGSNANNLTVYK	Deamidation N	8 / 9	De-novo
734,3528	2	1466,6900	1466,6888	0,0012	0,8018	77,7778	206,1086	1	14	LGDGSNADNLTVYK	Deamidation N	9	De-novo
530,2881	2	1058,5606	1058,5607	-0,0001	-0,1171	56,8627	83,6237	1	9	ELELTNLAR	Deamidation N	6	De-novo
530,2881	2	1058,5606	1058,5608	-0,0002	-0,2116	56,8627	82,8579	1	9	ELELTQLGR	Deamidation Q	6	De-novo
530,2881	2	1058,5606	1058,5608	-0,0002	-0,2116	56,8627	82,6066	1	9	ELELTELGR			De-novo
672,3406	2	1342,6656	1342,663	0,0026	1,9186	46,3768	54,5016	1	12	NNGWSGQLTLPR	Deamidation N	2	De-novo
672,3406	2	1342,6656	1342,663	0,0026	1,9186	46,3768	53,7988	1	12	NDGWSGQLTLPR			De-novo
672,3406	2	1342,6656	1342,663	0,0026	1,9186	46,3768	53,9623	1	12	NNGWSGQLTLPR	Deamidation N	1	De-novo
562,3108	2	1122,6060	1122,6073	-0,0013	-1,1794	56,8627	94,2558	1	9	LFTPQTLFR	Deamidation Q	5	De-novo
562,3108	2	1122,6060	1122,6073	-0,0013	-1,1794	49,0196	92,6269	1	9	LFTPLTEFR			De-novo
562,3108	2	1122,6060	1122,6073	-0,0013	-1,1794	49,0196	92,2113	1	9	LFTPLQTFR	Deamidation Q	6	De-novo

NCBI gi|156968287|gb|ABU98615.1| fructosidase [Helicoverpa armigera] Total Score: 270

```
                  0       100       200       300       400
                  |         |         |         |         |      |479
gi|156968287|gb|ABU98  | ____________________________________________
Local hits (HSPs)      |                     __           _
```

```
 Score = 96 (49.5 bits)
 Identities = 11/13 (84%), Positives = 13/13 (100%)

Query:    81 RNDGWSGQLTLPR 93
             RNDGWSGQ+T+PR
Sbjct:   308 RNDGWSGQITIPR 320   MatchSeq

 Score = 91 (47.0 bits)
 Identities = 13/15 (86%), Positives = 14/15 (93%)

Query:   132 RLGDGSNANDLTVYK 146
             RLGD SNA+DLTVYK
Sbjct:   454 RLGDSSNADDLTVYK 468   MatchSeq

 Score = 49 (26.1 bits)
 Identities = 7/7 (100%), Positives = 7/7 (100%)

Query:    60 RELELTE 66
             RELELTE
Sbjct:   320 RELELTE 326   MatchSeq

 Score = 34 (18.7 bits)
 Identities = 6/10 (60%), Positives = 8/10 (80%)

Query:    38 LTVYKELELT 47
             +T+  ELELT
Sbjct:   316 ITIPRELELT 325   MatchSeq
```

Ha SG EST hargln|HA-GLN_Contig_477 FWD frame 1 Total Score: 336

```
                  0        90       180       270       360
                  |         |         |         |         |      |442
hargln|HA-GLN_Contig_  | ____________________________________________
Local hits (HSPs)      |                _    _              _
```

```
 Score = 96 (49.5 bits)
 Identities = 11/13 (84%), Positives = 13/13 (100%)

Query:    81 RNDGWSGQLTLPR 93
             RNDGWSGQ+T+PR
Sbjct:   215 RNDGWSGQITIPR 227

 Score = 91 (47.0 bits)
 Identities = 13/15 (86%), Positives = 14/15 (93%)

Query:   132 RLGDGSNANDLTVYK 146
             RLGD SNA+DLTVYK
Sbjct:   361 RLGDSSNADDLTVYK 375

 Score = 66 (34.6 bits)
 Identities = 8/9 (88%), Positives = 9/9 (100%)

Query:   115 LFTPLTEFR 123
             LFTP+TEFR
Sbjct:   170 LFTPITEFR 178

 Score = 49 (26.1 bits)
 Identities = 7/7 (100%), Positives = 7/7 (100%)

Query:    60 RELELTE 66
             RELELTE
Sbjct:   227 RELELTE 233

 Score = 34 (18.7 bits)
 Identities = 6/10 (60%), Positives = 8/10 (80%)

Query:    38 LTVYKELELT 47
             +T+  ELELT
Sbjct:   223 ITIPRELELT 232
```

Spot 60

m/z	Charge	Peak mW	Peptide mW	Delta (Da)	Delta (ppm)	Ladder Score	Log Likelihood	Start	End	Sequence	Modifications	Subs	Query Tool
407,2262	2	812,4368	812,4392	-0,0024	-2,9836	35,5556	89,7919	1	8	EAAQGPLK			De-novo
407,2262	2	812,4368	812,4391	-0,0023	-2,8605	41,1765	91,288	1	9	EAAGAGPLK			De-novo
407,2262	2	812,4368	812,4392	-0,0024	-2,9836	35,5556	90,3327	1	8	AQAQGPLK	Deamidation Q	2	De-novo
464,7786	2	927,5416	927,5389	0,0027	2,8851	57,8947	132,2633	1	10	LLPASTGAAK			De-novo
464,7786	2	927,5416	927,5389	0,0027	2,8851	56,8627	130,1499	1	9	LLPASTQAK			De-novo
487,2708	2	972,5260	972,5181	0,0079	8,0986	54,902	108,9924	1	9	GPGKFPWGK			De-novo
487,2708	2	972,5260	972,5103	0,0157	16,1191	55,5556	103,8114	1	8	LLEMPWGK			De-novo
487,2708	2	972,5260	972,4851	0,0409	42,0325	48,8889	105,8877	1	8	MPKNPWGK	Oxidation M	1	De-novo
411,2266	2	820,4376	820,4443	-0,0067	-8,1956	64,1026	105,833	1	7	LAVFSQR	Deamidation Q	6	De-novo
411,2266	2	820,4376	820,4443	-0,0067	-8,1956	64,1026	105,6296	1	7	LAVFSER			De-novo
417,2326	2	832,4496	832,4556	-0,0060	-7,2364	66,6667	136,6369	1	8	LGLNGFGR			De-novo
417,2326	2	832,4496	832,4192	0,0304	36,4912	71,1111	130,9789	1	8	VSNPGFGR			De-novo
417,2326	2	832,4496	832,4192	0,0304	36,4912	66,6667	131,1647	1	8	SVNPGFGR			De-novo
512,7785	2	1023,5414	1023,5389	0,0025	2,4191	57,7778	58,4913	1	8	VYFLETRP			De-novo
579,6084	3	1735,8018	1735,8167	-0,0149	-8,6046	45,1613	201,6429	1	16	TANYAPHYLATNATAR	Deamidation N	3 / 12	De-novo
579,6084	3	1735,8018	1735,8167	-0,0149	-8,6046	41,9355	199,2366	1	16	TANSFPHYLATNATAR	Deamidation N	3 / 12	De-novo
579,6084	3	1735,8018	1735,8167	-0,0149	-8,6046	45,1613	199,8572	1	16	TADYAPHYLATNATAR	Deamidation N	12	De-novo
883,895	2	1765,7744	1765,7756	-0,0012	-0,6932	39,3939	112,649	1	17	GTSEQADTDLSGWGNTK			De-novo
883,895	2	1765,7744	1765,7756	-0,0012	-0,6932	39,3939	112,3033	1	17	ASSEQADTDLSGWGNTK			De-novo
883,895	2	1765,7744	1765,7756	-0,0012	-0,6932	41,4141	112,6239	1	17	TGSEQADTDLSGWGNTK			De-novo
747,4403	2	1492,8650	1492,8615	0,0035	2,3284	41,9753	76,9954	1	14	VPVPNVSKQGDLLK			De-novo
747,4403	2	1492,8650	1492,8615	0,0035	2,3284	45,679	75,9378	1	14	VPVPNVSKGQDLLK			De-novo
747,4403	2	1492,8650	1492,8615	0,0035	2,3284	41,9753	76,7374	1	14	VPVPNVSKQGNLLK	Deamidation N	11	De-novo
406,7476	2	811,4796	811,4803	-0,0007	-0,8922	48,8889	120,7263	1	8	LVPALDGK			De-novo
910,95	2	1819,8844	1819,8632	0,0212	11,6360	40,9524	222,0254	1	18	NSAMTMAASPACLAPLAK	Oxidation M /	4 / 12	De-novo
910,95	2	1819,8844	1819,8622	0,0222	12,1855	39,0476	213,2476	1	18	NSATTDDASVSCLAPLAK	Carbamidomethyl C	12	De-novo
910,95	2	1819,8844	1819,8622	0,0222	12,1855	39,0476	213,6792	1	18	NSATTNDASVSCLAPLAK	Deamidation N /	6 / 12	De-novo
913,9348	2	1825,8540	1825,9244	-0,0704	-38,5690	51,4286	182,6666	1	18	NSASVLPPSSDCLAPLAK	Carbamidomethyl C	12	De-novo
913,9348	2	1825,8540	1825,9244	-0,0704	-38,5690	51,4286	179,9481	1	18	NSASVLPAAEDCLAPLAK	Carbamidomethyl C	12	De-novo
913,9348	2	1825,8540	1825,8993	-0,0453	-24,8228	45,7143	186,208	1	18	NSASDANAPLNCLAPLAK	Carbamidomethyl C	12	De-novo
897,9077	2	1793,7998	1793,8254	-0,0256	-14,2846	38,3838	93,3597	1	17	CTTGEADGLLSGWGNKK	Carbamidomethyl C /	1 / 15	De-novo
897,9077	2	1793,7998	1793,8254	-0,0256	-14,2846	31,4286	95,9916	1	18	AASCSEADGLLSGWGNKK	Deamidation N	16	De-novo
897,9077	2	1793,7998	1793,8254	-0,0256	-14,2846	40,404	92,8952	1	17	TCTGEADGLLSGWGNKK	Carbamidomethyl C /	2 / 15	De-novo
713,8864	2	1425,7572	1425,7576	-0,0004	-0,2974	66,6667	324,3921	1	15	AANQNLLPASTGAAK			De-novo
713,8864	2	1425,7572	1425,7576	-0,0004	-0,2974	46,4646	328,7789	1	17	GANGALAGLPASTGAAK			De-novo
713,8864	2	1425,7572	1425,7576	-0,0004	-0,2974	50,5051	327,3543	1	17	GANGAGALLPASTGAAK			De-novo
905,9362	2	1809,8568	1809,9659	-0,1091	-60,2906	37,1429	216,0902	1	18	SANSVLPAEGLCLAPLAK	Carbamidomethyl C	12	De-novo
905,9362	2	1809,8568	1809,8679	-0,0111	-6,1463	46,6667	228,6932	1	18	SANSEGPPANNCLAPLAK	Carbamidomethyl C	12	De-novo
905,9362	2	1809,8568	1809,9659	-0,1091	-60,2906	37,1429	215,6231	1	18	ASNSVLPAEGLCLAPLAK	Carbamidomethyl C	12	De-novo

NCBI gi|53830712|gb|AAU95199.1| putative glyceraldehyde-3-phosphate dehydrogenase Total Score: 399

```
                  0        70       140       210       280
                  |         |         |         |         |      |333
gi|53830712|gb|AAU951  | ____________________________________________
Local hits (HSPs)      | _      ___ _        _     _____ _
```

```
 Score = 75 (39.9 bits)
 Identities = 10/14 (71%), Positives = 12/14 (85%)

Query:   426 ANQNLLPASTGAAK 439
             A QN++PA TGAAK
Sbjct:   199 AQQNIIPAATGAAK 212   MatchSeq

 Score = 73 (38.9 bits)
 Identities = 10/18 (55%), Positives = 13/18 (72%)

Query:   464 LLPASTGAAKSANSVLPA 481
             ++PA TGAAK    V+PA
Sbjct:   203 IIPAATGAAKAVGKVIPA 220   MatchSeq

 Score = 62 (33.3 bits)
 Identities = 7/10 (70%), Positives = 10/10 (100%)

Query:   546 KLGLNGFGRV 555
             K+G+NGFGR+
Sbjct:     3 KIGINGFGRI 12   MatchSeq

 Score = 55 (29.8 bits)
 Identities = 8/8 (100%), Positives = 8/8 (100%)

Query:   502 NCLAPLAK 509
             NCLAPLAK
Sbjct:   152 NCLAPLAK 159   MatchSeq

 Score = 51 (27.7 bits)
 Identities = 7/7 (100%), Positives = 7/7 (100%)

Query:   215 VPVPNVS 221
             VPVPNVS
Sbjct:   232 VPVPNVS 238   MatchSeq

 Score = 49 (26.7 bits)
 Identities = 6/8 (75%), Positives = 8/8 (100%)

Query:    69 KLAVFSQR 76
             K+AVFS+R
Sbjct:    70 KIAVFSER 77   MatchSeq

 Score = 34 (19.1 bits)
 Identities = 4/4 (100%), Positives = 4/4 (100%)

Query:    50 PWGK 53
             PWGK
Sbjct:    83 PWGK 86   MatchSeq
```

ButterflyBase HAP00799_1 Total Score: 511

```
                 0        60       120       180       240
                 |         |         |         |         |      |297
db|HAP00799_1|HAP0079  | ____________________________________________
Local hits (HSPs)      | __       __ __      __     _______   __
```

```
 Score = 81 (43.0 bits)
 Identities = 11/14 (78%), Positives = 13/14 (92%)

Query:   426 ANQNLLPASTGAAK 439
             A QN++PASTGAAK
Sbjct:   199 AQQNIIPASTGAAK 212

 Score = 79 (42.0 bits)
 Identities = 11/18 (61%), Positives = 14/18 (77%)

Query:   464 LLPASTGAAKSANSVLPA 481
             ++PASTGAAK    V+PA
Sbjct:   203 IIPASTGAAKAVGKVIPA 220

 Score = 62 (33.3 bits)
 Identities = 7/10 (70%), Positives = 10/10 (100%)

Query:   546 KLGLNGFGRV 555
             K+G+NGFGR+
Sbjct:     3 KIGINGFGRI 12

 Score = 55 (29.8 bits)
 Identities = 8/8 (100%), Positives = 8/8 (100%)

Query:   502 NCLAPLAK 509
             NCLAPLAK
Sbjct:   152 NCLAPLAK 159

 Score = 54 (29.3 bits)
 Identities = 8/8 (100%), Positives = 8/8 (100%)

Query:     1 EAAQGPLK 8
             EAAQGPLK
Sbjct:   261 EAAQGPLK 268

 Score = 51 (27.7 bits)
 Identities = 7/7 (100%), Positives = 7/7 (100%)

Query:   215 VPVPNVS 221
             VPVPNVS
Sbjct:   232 VPVPNVS 238

 Score = 49 (26.7 bits)
 Identities = 6/8 (75%), Positives = 8/8 (100%)

Query:    69 KLAVFSQR 76
             K+AVFS+R
Sbjct:    70 KIAVFSER 77

 Score = 46 (25.2 bits)
 Identities = 7/13 (53%), Positives = 9/13 (69%)

Query:   256 KLVPALDGKNSAM 268
             K +PAL+GK   M
Sbjct:   216 KVIPALNGKLTGM 228

 Score = 34 (19.1 bits)
 Identities = 4/4 (100%), Positives = 4/4 (100%)

Query:    50 PWGK 53
             PWGK
Sbjct:    83 PWGK 86
```

Ha SG EST hargln|HA-GLN_Contig_592 FWD frame 2 Total Score: 506

```
                  0        90       180       270       360
                  |         |         |         |         |      |435
hargln|HA-GLN_Contig_  | ____________________________________________
Local hits (HSPs)      |  _       __      ___    ____   _
```

```
 Score = 81 (43.0 bits)
 Identities = 11/14 (78%), Positives = 13/14 (92%)

Query:   426 ANQNLLPASTGAAK 439
             A QN++PASTGAAK
Sbjct:   224 AQQNIIPASTGAAK 237

 Score = 79 (42.0 bits)
 Identities = 11/18 (61%), Positives = 14/18 (77%)

Query:   464 LLPASTGAAKSANSVLPA 481
             ++PASTGAAK    V+PA
Sbjct:   228 IIPASTGAAKAVGKVIPA 245

 Score = 62 (33.3 bits)
 Identities = 7/10 (70%), Positives = 10/10 (100%)

Query:   546 KLGLNGFGRV 555
             K+G+NGFGR+
Sbjct:    28 KIGINGFGRI 37

 Score = 54 (29.3 bits)
 Identities = 8/8 (100%), Positives = 8/8 (100%)

Query:     1 EAAQGPLK 8
             EAAQGPLK
Sbjct:   286 EAAQGPLK 293

 Score = 51 (27.7 bits)
 Identities = 7/7 (100%), Positives = 7/7 (100%)

Query:   215 VPVPNVS 221
             VPVPNVS
Sbjct:   257 VPVPNVS 263

 Score = 50 (27.2 bits)
 Identities = 7/8 (87%), Positives = 7/8 (87%)

Query:   502 NCLAPLAK 509
             NCLAP AK
Sbjct:   177 NCLAPXAK 184

 Score = 49 (26.7 bits)
 Identities = 6/8 (75%), Positives = 8/8 (100%)

Query:    69 KLAVFSQR 76
             K+AVFS+R
Sbjct:    95 KIAVFSER 102

 Score = 46 (25.2 bits)
 Identities = 7/13 (53%), Positives = 9/13 (69%)

Query:   256 KLVPALDGKNSAM 268
             K +PAL+GK   M
Sbjct:   241 KVIPALNGKLTGM 253

 Score = 34 (19.1 bits)
 Identities = 4/4 (100%), Positives = 4/4 (100%)

Query:    50 PWGK 53
             PWGK
Sbjct:   108 PWGK 111
```

Spot 61

m/z	Charge	Peak mW	Peptide mW	Delta (Da)	Delta (ppm)	Ladder Score	Log Likelihood	Start	End	Sequence	Modifications	Substitu	Query Tool
407,2267	2	812,4378	812,4756	-0,0378	-46,5540	51,1111	104,8652	1	8	TAVQGPLK			De-novo
407,2267	2	812,4378	812,4392	-0,0014	-1,7527	57,7778	103,2334	1	8	EAAQGPLK			De-novo
407,2267	2	812,4378	812,4392	-0,0014	-1,7527	60	102,8027	1	8	AEAQGPLK			De-novo
464,7776	2	927,5396	927,5389	0,0007	0,7288	59,6491	147,2576	1	10	LLPASTGAAK			De-novo
464,7776	2	927,5396	927,5389	0,0007	0,7288	58,8235	144,2234	1	9	LLPASTQAK			De-novo
464,7776	2	927,5396	927,4662	0,0734	79,1145	57,8947	141,5483	1	10	QPPASTGAAK	Deamidation Q	1	De-novo
406,2058	2	810,3960	810,4058	-0,0098	-12,1223	56,4103	133,1164	1	7	LMTGAFR	Oxidation M	2	De-novo
406,2058	2	810,3960	810,4058	-0,0098	-12,1223	51,2821	132,9404	1	7	MLTGAFR	Oxidation M	1	De-novo
406,2058	2	810,3960	810,4058	-0,0098	-12,1223	76,9231	132,9593	1	7	TLMGAFR	Oxidation M	3	De-novo
713,889	2	1425,7624	1425,7576	0,0048	3,3498	64,3678	228,6062	1	15	AANQNLLPASTGAAK			De-novo
713,889	2	1425,7624	1425,7576	0,0048	3,3498	66,6667	228,0085	1	15	GAQQNLLPASTGAAK			De-novo
713,889	2	1425,7624	1425,7576	0,0048	3,3498	66,6667	226,0143	1	15	AGQQNLLPASTGAAK			De-novo
411,2296	2	820,4436	820,4443	-0,0007	-0,8824	58,9744	137,9853	1	7	LAVFSQR	Deamidation Q	6	De-novo
411,2296	2	820,4436	820,4443	-0,0007	-0,8824	58,9744	138,1662	1	7	LAVFSER			De-novo
411,2296	2	820,4436	820,4483	-0,0047	-5,7578	58,9744	138,7466	1	7	LAVFFQP			De-novo
417,7271	2	833,4386	833,4395	-0,0009	-1,1087	64,4444	133,9496	1	8	LGLNGFGR	Deamidation N	4	De-novo
417,7271	2	833,4386	833,4396	-0,0010	-1,2286	64,4444	131,6758	1	8	LGLDGFGR			De-novo
417,7271	2	833,4386	833,4144	0,0242	29,0084	57,7778	124,2691	1	8	LGNNGFGR			De-novo
747,4397	2	1492,8638	1492,8613	0,0025	1,6586	54,321	46,8238	1	14	VPVPNVSVVDSLLR			De-novo
747,4397	2	1492,8638	1492,8613	0,0025	1,6586	54,321	46,2623	1	14	PVVPNVSVVDSLLR			De-novo
747,4397	2	1492,8638	1492,8613	0,0025	1,6586	49,3827	45,6315	1	14	VPVPNVSVVDVTLR			De-novo

NCBI gi|157674455|gb|ABV60323.1| putative glyceraldehyde-3-phosphate dehydrogenase [Lutzomyia longipalpis] Total Score: 485

```
                 0        70       140       210       280
                 |         |         |         |        |         |332
gi|157674455|gb|ABV60  | ______________________________________________
Local hits (HSPs)      | _   ___               ___  __  _
```

```
Score = 98 (51.5 bits)
Identities = 13/16 (81%), Positives = 15/16 (93%)

Query:   90 GAQQNLLPASTGAAKA 105
            GAQQN++PA TGAAKA
Sbjct:  198 GAQQNIIPAATGAAKA 213   MatchSeq

Score = 96 (50.5 bits)
Identities = 13/15 (86%), Positives = 13/15 (86%)

Query:  192 RVPVPNVSVVDVTLR 206
            RVPVPNVSVVD T R
Sbjct:  231 RVPVPNVSVVDLTVR 245   MatchSeq

Score = 81 (42.9 bits)
Identities = 11/16 (68%), Positives = 13/16 (81%)

Query:   74 RAANQNLLPASTGAAK 89
            R A QN++PA TGAAK
Sbjct:  197 RGAQQNIIPAATGAAK 212   MatchSeq

Score = 64 (34.3 bits)
Identities = 7/10 (70%), Positives = 10/10 (100%)

Query:  141 LGLNGFGRLG 150
            +G+NGFGR+G
Sbjct:    4 IGINGFGRIG 13   MatchSeq

Score = 62 (33.3 bits)
Identities = 7/10 (70%), Positives = 10/10 (100%)

Query:  246 KLGLNGFGRV 255
            K+G+NGFGR+
Sbjct:    3 KIGINGFGRI 12   MatchSeq

Score = 44 (24.1 bits)
Identities = 7/8 (87%), Positives = 7/8 (87%)

Query:  206 REAAQGPL 213
            REAA GPL
Sbjct:  260 REAANGPL 267   MatchSeq

Score = 40 (22.1 bits)
Identities = 8/15 (53%), Positives = 9/15 (60%)

Query:  119 KLAVFSQRLAVFSER 133
            KL V  Q + VF ER
Sbjct:   63 KLVVNGQAITVFCER 77   MatchSeq
```

ButterflyBase HAP00799_1 Total Score: 461

```
             0        60       120       180       240
             |         |         |         |         |         |297
butterfly|HAP00799_1   | ______________________________________________
Local hits (HSPs)      | _          _                   __  ____  _
```

```
Score = 104 (54.6 bits)
Identities = 14/16 (87%), Positives = 16/16 (100%)

Query:   90 GAQQNLLPASTGAAKA 105
            GAQQN++PASTGAAKA
Sbjct:  198 GAQQNIIPASTGAAKA 213

Score = 96 (50.5 bits)
Identities = 13/15 (86%), Positives = 13/15 (86%)

Query:  192 RVPVPNVSVVDVTLR 206
            RVPVPNVSVVD T R
Sbjct:  231 RVPVPNVSVVDLTVR 245

Score = 87 (45.9 bits)
Identities = 12/16 (75%), Positives = 14/16 (87%)

Query:   74 RAANQNLLPASTGAAK 89
            R A QN++PASTGAAK
Sbjct:  197 RGAQQNIIPASTGAAK 212

Score = 64 (34.3 bits)
Identities = 7/10 (70%), Positives = 10/10 (100%)

Query:  141 LGLNGFGRLG 150
            +G+NGFGR+G
Sbjct:    4 IGINGFGRIG 13

Score = 61 (32.7 bits)
Identities = 9/9 (100%), Positives = 9/9 (100%)

Query:    8 KEAAQGPLK 16
            KEAAQGPLK
Sbjct:  260 KEAAQGPLK 268

Score = 49 (26.7 bits)
Identities = 6/8 (75%), Positives = 8/8 (100%)

Query:  119 KLAVFSQR 126
            K+AVFS+R
Sbjct:   70 KIAVFSER 77
```

Ha SG EST hargln|HA-GLN Contig 592 FWD frame 2 Total Score: 523

```
                 0        90       180       270       360
                 |         |         |         |        |         |435
hargln|HA-GLN_Contig_  | ______________________________________________
Local hits (HSPs)      |  _       _             __  ___ _
```

```
Score = 104 (54.6 bits)
Identities = 14/16 (87%), Positives = 16/16 (100%)

Query:   90 GAQQNLLPASTGAAKA 105
            GAQQN++PASTGAAKA
Sbjct:  223 GAQQNIIPASTGAAKA 238

Score = 96 (50.5 bits)
Identities = 13/15 (86%), Positives = 13/15 (86%)

Query:  192 RVPVPNVSVVDVTLR 206
            RVPVPNVSVVD T R
Sbjct:  256 RVPVPNVSVVDLTVR 270

Score = 87 (46.0 bits)
Identities = 12/16 (75%), Positives = 14/16 (87%)

Query:   74 RAANQNLLPASTGAAK 89
            R A QN++PASTGAAK
Sbjct:  222 RGAQQNIIPASTGAAK 237

Score = 64 (34.3 bits)
Identities = 7/10 (70%), Positives = 10/10 (100%)

Query:  141 LGLNGFGRLG 150
            +G+NGFGR+G
Sbjct:   29 IGINGFGRIG 38

Score = 62 (33.3 bits)
Identities = 7/10 (70%), Positives = 10/10 (100%)

Query:  246 KLGLNGFGRV 255
            K+G+NGFGR+
Sbjct:   28 KIGINGFGRI 37

Score = 61 (32.8 bits)
Identities = 9/9 (100%), Positives = 9/9 (100%)

Query:    8 KEAAQGPLK 16
            KEAAQGPLK
Sbjct:  285 KEAAQGPLK 293

Score = 49 (26.7 bits)
Identities = 6/8 (75%), Positives = 8/8 (100%)

Query:  119 KLAVFSQR 126
            K+AVFS+R
Sbjct:   95 KIAVFSER 102
```

Spot 62

m/z	Charge	Peak mW	Peptide mW	Delta (Da)	Delta (ppm)	Ladder Score	Log Likelihood	Start	End	Sequence	Modifications	Subs	Query Tool
796,3789	2	1590,7422	1590,7373	0,0049	3,0652	59,7701	160,0439	1	15	DLTETGVELGANSER	Deamidation N	12	De-novo
796,3789	2	1590,7422	1590,7373	0,0049	3,0652	60,9195	159,3096	1	15	LDTETGVELGANSER	Deamidation N	12	De-novo
796,3789	2	1590,7422	1590,7373	0,0049	3,0652	60,9195	159,8696	1	15	EVTETGVELGANSER	Deamidation N	12	De-novo
605,3115	2	1208,6074	1208,6036	0,0038	3,1243	55,5556	105,2879	1	11	YLAEVLNSSGR	Deamidation N	7	De-novo
605,3115	2	1208,6074	1208,6036	0,0038	3,1243	49,2063	104,2595	1	11	YLAEVLNSGSR	Deamidation N	7	De-novo
605,3115	2	1208,6074	1208,6036	0,0038	3,1243	52,381	103,9702	1	11	LYAEVLNSSGR	Deamidation N	7	De-novo
607,8088	2	1213,6020	1213,5979	0,0041	3,3586	73,6842	217,4318	1	10	NLQYYNLSAK	Deamidation N	6	De-novo
607,8088	2	1213,6020	1213,5979	0,0041	3,3586	73,6842	215,2698	1	10	NLQYYDLSAK			De-novo
607,8088	2	1213,6020	1213,5979	0,0041	3,3586	61,9048	211,702	1	11	NLGAYYNLSAK	Deamidation N	7	De-novo
647,8098	2	1293,6040	1293,599	0,0050	3,8466	66,6667	132,3271	1	11	FNVWNTAQGEK	Deamidation N	5	De-novo
647,8098	2	1293,6040	1293,599	0,0050	3,8466	66,6667	131,3655	1	11	FNVWNTAGQEK	Deamidation N	5	De-novo
647,8098	2	1293,6040	1293,599	0,0050	3,8466	66,6667	131,6906	1	11	FNVWNTAQGQK	Deamidation N /	5 / 10	De-novo
729,8787	2	1457,7418	1457,7372	0,0046	3,1391	70,6667	278,206	1	13	CVEGLPLVLCGNK	Carbamidomethyl C	1 / 10	De-novo
729,8787	2	1457,7418	1457,7371	0,0047	3,2077	70,6667	278,12	1	13	CVQGLPLVLCGNK	Carbamidomethyl C /	1 / 3 / 10	De-novo
729,8787	2	1457,7418	1457,7371	0,0047	3,2077	62,963	276,5912	1	14	CVEGLPLVLGCGNK	Carbamidomethyl C	1	De-novo
531,7684	2	1061,5212	1061,5176	0,0036	3,3688	80,9524	218,7529	1	11	CVLVGDGGTGK	Carbamidomethyl C	1	De-novo
531,7684	2	1061,5212	1061,5176	0,0036	3,3688	80,9524	216,9824	1	11	CVLVGNGGTGK	Carbamidomethyl C /	1 / 6	De-novo
531,7684	2	1061,5212	1061,5176	0,0036	3,3688	76,1905	209,2039	1	11	CVLVGDGTGGK	Carbamidomethyl C	1	De-novo
725,3613	2	1448,7070	1448,7157	-0,0087	-6,0219	62,3188	132,0689	1	12	CELFPLVLCGNK	Carbamidomethyl C	1 / 9	De-novo
725,3613	2	1448,7070	1448,7157	-0,0087	-6,0219	56,5217	131,9162	1	12	CEFLPLVLCGNK	Carbamidomethyl C	1 / 9	De-novo
725,3613	2	1448,7070	1448,6826	0,0244	16,8263	60,8696	132,304	1	12	CELMPLVLCGNK	Carbamidomethyl C /	1 / 4 / 9	De-novo
539,7662	2	1077,5168	1077,5818	-0,0650	-60,3425	49,1228	95,8656	1	10	KFLVGNNTGK	Deamidation N	6	De-novo

NCBI gi|242006141|ref|XP_002423913.1| GTP-binding nuclear protein RAN1, putative [Pediculus humanus corporis] Total Score: 351

```
                 0        90       180       270       360
                 |         |         |         |        |         |430
gi|242006141|ref|XP_0  | ______________________________________________
Local hits (HSPs)      | __   _      __ _           _
```

```
Score = 90 (47.3 bits)
Identities = 11/12 (91%), Positives = 12/12 (100%)

Query:  121 FNVWNTAGQEKF 132
            FNVW+TAGQEKF
Sbjct:   59 FNVWDTAGQEKF 70   MatchSeq

Score = 82 (43.2 bits)
Identities = 12/12 (100%), Positives = 12/12 (100%)

Query:  182 KCVLVGDGGTGK 193
            KCVLVGDGGTGK
Sbjct:   10 KCVLVGDGGTGK 21   MatchSeq

Score = 80 (42.2 bits)
Identities = 10/11 (90%), Positives = 11/11 (100%)

Query:   88 KNLQYYDLSAK 98
            KNLQYYD+SAK
Sbjct:  140 KNLQYYDISAK 150   MatchSeq

Score = 66 (35.1 bits)
Identities = 9/15 (60%), Positives = 12/15 (80%)

Query:  228 CEFLPLVLCGNKCEL 242
            CE +P VLCGNK ++
Sbjct:  110 CENIPMVLCGNKVDI 124   MatchSeq

Score = 33 (18.5 bits)
Identities = 4/4 (100%), Positives = 4/4 (100%)

Query:  226 NKCE 229
            NKCE
Sbjct:  238 NKCE 241   MatchSeq
```

ButterflyBase BMP006112_1 Total Score: 375

```
                 0        40        80        120       160
                 |         |          |         |         |   |176
db|BMP006112_1|BMP006  | ______________________________________________
Local hits (HSPs)      |     ___         ___         __     ___
```

```
Score = 90 (47.3 bits)
Identities = 11/12 (91%), Positives = 12/12 (100%)

Query:  121 FNVWNTAGQEKF 132
            FNVW+TAGQEKF
Sbjct:   65 FNVWDTAGQEKF 76

Score = 82 (43.2 bits)
Identities = 12/12 (100%), Positives = 12/12 (100%)

Query:  182 KCVLVGDGGTGK 193
            KCVLVGDGGTGK
Sbjct:   16 KCVLVGDGGTGK 27

Score = 80 (42.2 bits)
Identities = 10/11 (90%), Positives = 11/11 (100%)

Query:   88 KNLQYYDLSAK 98
            KNLQYYD+SAK
Sbjct:  146 KNLQYYDISAK 156

Score = 75 (39.7 bits)
Identities = 9/11 (81%), Positives = 11/11 (100%)

Query:  145 EGLPLVLCGNK 155
            EG+P+VLCGNK
Sbjct:  117 EGIPIVLCGNK 127

Score = 48 (26.0 bits)
Identities = 6/9 (66%), Positives = 8/9 (88%)

Query:  103 YYNLSAKFN 111
            YY++SAK N
Sbjct:  150 YYDISAKSN 158
```

Ha SG EST hargln|HA-GLN Contig 1236 FWD frame 1 Total Score: 327

```
                 0        90       180       270       360
                 |         |         |         |         |       |410
hargln|HA-GLN_Contig_  | ______________________________________________
Local hits (HSPs)      |    _      _    _  ___
```

```
Score = 90 (47.3 bits)
Identities = 11/12 (91%), Positives = 12/12 (100%)

Query:  121 FNVWNTAGQEKF 132
            FNVW+TAGQEKF
Sbjct:   93 FNVWDTAGQEKF 104

Score = 82 (43.2 bits)
Identities = 12/12 (100%), Positives = 12/12 (100%)

Query:  182 KCVLVGDGGTGK 193
            KCVLVGDGGTGK
Sbjct:   44 KCVLVGDGGTGK 55

Score = 80 (42.2 bits)
Identities = 10/11 (90%), Positives = 11/11 (100%)

Query:   88 KNLQYYDLSAK 98
            KNLQYYD+SAK
Sbjct:  174 KNLQYYDISAK 184

Score = 75 (39.7 bits)
Identities = 9/11 (81%), Positives = 11/11 (100%)

Query:  145 EGLPLVLCGNK 155
            EG+P+VLCGNK
Sbjct:  145 EGIPIVLCGNK 155
```

Spot 63

m/z	Charge	Peak mW	Peptide mW	Delta (Da)	Delta (ppm)	Ladder Score	Log Likelihood	Start	End	Sequence	Modifications	Subs	Query Tool
679,3521	2	1356,6886	1356,6885	0,0001	0,0560	79,7101	60,1981	1	12	LNDLENALQAQK	Deamidation N	6	De-novo
883,8971	2	1765,7786	1765,812	-0,0334	-18,9284	44,7619	146,7168	1	18	SGTDSNGATLLSGWGNTK	Deamidation N	6	De-novo
883,8971	2	1765,7786	1765,812	-0,0334	-18,9284	47,619	145,1399	1	18	SGTDNSGATLLSGWGNTK	Deamidation N	5	De-novo
883,8971	2	1765,7786	1765,812	-0,0334	-18,9284	43,4343	140,9536	1	17	SGTDNESTLLSGWGNTK			De-novo
797,8392	2	1593,6628	1593,665	-0,0022	-1,3955	62,6667	215,0721	1	13	LTPCNETNEEVCK	Carbamidomethyl C /	4 / 5 / 12	De-novo
797,8392	2	1593,6628	1593,665	-0,0022	-1,3955	62,6667	215,6837	1	13	LTPCNQTNEEVCK	Carbamidomethyl C /	/ 5 / 6 /	De-novo

NCBI gi|116833151|gb|ABK29488.1| promoting protein [Helicoverpa armigera] Total Score: 146

```
                 0         20        40        60
                 |          |          |          |          |77
gi|116833151|gb|ABK29  | ______________________________________________
Local hits (HSPs)      |             _____     ___
```

```
Score = 95 (49.6 bits)
Identities = 11/13 (84%), Positives = 13/13 (100%)

Query:   66 LTPCNETNEEVCK 78
            +TPC+ETNEEVCK
Sbjct:   23 ITPCDETNEEVCK 35   MatchSeq

Score = 51 (27.5 bits)
Identities = 6/8 (75%), Positives = 7/8 (87%)

Query:   79 LTPCNQTN 86
            +TPC QTN
Sbjct:   41 ITPCTQTN 48   MatchSeq
```

Ha SG EST none

Ha EST other harmi_rn|HA-RN-ALL Contig 1980 FWD frame 0 Total Score: 146

```
                 0        40        80        120       160
                 |         |          |          |          |   |173
harmi_rn|HA-RN-ALL_Co  | ______________________________________________
Local hits (HSPs)      |      _____  __
```

```
Score = 95 (49.6 bits)
Identities = 11/13 (84%), Positives = 13/13 (100%)

Query:   66 LTPCNETNEEVCK 78
            +TPC+ETNEEVCK
Sbjct:   28 ITPCDETNEEVCK 40

Score = 51 (27.5 bits)
Identities = 6/8 (75%), Positives = 7/8 (87%)

Query:   79 LTPCNQTN 86
            +TPC QTN
Sbjct:   46 ITPCTQTN 53
```

Spot 64

m/z	Charge	Peak mW	Peptide mW	Delta (Da)	Delta (ppm)	Ladder Score	Log Likelihood	Start	End	Sequence	Modifications	Subs	Query Tool
725,3633	2	1448,7110	1448,726	-0,0150	-10,3705	76,5432	370,2491	1	14	LNEAFGSQSGKPSK			De-novo
483,9197	3	1448,7357	1448,726	0,0097	6,6707	69,1358	242,2314	1	14	LNEAFGSQSGKPSK			De-novo
483,9197	3	1448,7357	1448,726	0,0097	6,6707	69,1358	242,2314	1	14	LNEAFGSQSGKPSK			De-novo
725,3633	2	1448,7110	1448,726	-0,0150	-10,3705	74,0741	368,6682	1	14	NLEAFGSQSGKPSK			De-novo
483,9197	3	1448,7357	1448,726	0,0097	6,6707	64,1975	242,9147	1	14	NLEAFGSQSGKPSK			De-novo
483,9197	3	1448,7357	1448,726	0,0097	6,6707	64,1975	242,9147	1	14	NLEAFGSQSGKPSK			De-novo
725,3633	2	1448,7110	1448,726	-0,0150	-10,3705	79,0123	361,8715	1	14	QVQAFGSQSGKPSK	Deamidation Q	3	De-novo
569,2791	2	1136,5426	1136,5463	-0,0037	-3,2766	80,9524	263,4294	1	11	NLEAFGSQSGK			De-novo
569,2791	2	1136,5426	1136,5463	-0,0037	-3,2766	74,6032	261,3531	1	11	LNEAFGSQSGK			De-novo
569,2791	2	1136,5426	1136,5463	-0,0037	-3,2766	79,7101	260,4201	1	12	NLEAFGSAGSGK			De-novo
716,8623	2	1431,7090	1431,7106	-0,0016	-1,1343	62,069	172,0202	1	15	THTAFGSAGGSKPSK			De-novo
417,7064	2	833,3972	833,4032	-0,0060	-7,2282	69,2308	116,7217	1	7	TSWLDGR			De-novo
741,0161	3	2220,0249	2219,957	0,0679	30,5700	48,2993	378,628	1	25	NPETGAGGAGFGATADPNTNGTGGK	Deamidation N	18 / 20	De-novo
741,0161	3	2220,0249	2219,957	0,0679	30,5700	48,2993	377,6711	1	25	NPETGAGGGAFGATADPNTNGTGGK	Deamidation N	18 / 20	De-novo
741,0161	3	2220,0249	2219,957	0,0679	30,5700	48,2993	378,1113	1	25	NPETGAGAGGFGATADPNTNGTGGK	Deamidation N	18 / 20	De-novo
735,6837	3	2204,0277	2203,9377	0,0900	40,8197	50,3704	325,8355	1	23	NFPMPNMSGTGAGGTNHNGTGGK	Deamidation N	18	De-novo
735,6837	3	2204,0277	2203,9377	0,0900	40,8197	50,3704	325,6814	1	23	FNPMPNMSGTGAGGTNHNGTGGK	Deamidation N	18	De-novo
728,8571	2	1455,6986	1455,6995	-0,0009	-0,6347	55,5556	129,6065	1	14	YVFNVSADGLGTGR	Deamidation N	4	De-novo
728,8571	2	1455,6986	1455,6995	-0,0009	-0,6347	58,0247	129,3999	1	14	VYFNVSADGLGTGR	Deamidation N	4	De-novo
728,8571	2	1455,6986	1455,6995	-0,0009	-0,6347	55,5556	129,0839	1	14	YVFDVSANGLGTGR	Deamidation N	8	De-novo

NCBI gi|110764221|ref|XP_001119897.1| PREDICTED: similar to Cyclophilin 1 CG9916-PA [Apis mellifera] Total Score: 305

```
                      0         40        80        120       160
                      |         |         |         |         | |164
gi|110764221|ref|XP_0 | ________________________________________________
Local hits (HSPs)     | ____              __       ___  _    ____
```

```
 Score = 70 (37.8 bits)
 Identities = 9/14 (64%), Positives = 11/14 (78%)

Query:   345 KNLEAFGSQSGKTH 358
             K +E +GSQ GKTH
Sbjct:   140 KKIESYGSQTGKTH 153   MatchSeq
```

```
 Score = 64 (34.7 bits)
 Identities = 9/9 (100%), Positives = 9/9 (100%)

Query:   244 TNHNGTGGK 252
             TNHNGTGGK
Sbjct:    68 TNHNGTGGK 76   MatchSeq
```

```
 Score = 58 (31.6 bits)
 Identities = 7/9 (77%), Positives = 9/9 (100%)

Query:   289 RVYFNVSAD 297
             RVYF++SAD
Sbjct:     5 RVYFDISAD 13   MatchSeq
```

```
 Score = 40 (22.4 bits)
 Identities = 5/6 (83%), Positives = 5/6 (83%)

Query:   147 KTSWLD 152
             KT WLD
Sbjct:   118 KTTWLD 123   MatchSeq
```

```
 Score = 38 (21.3 bits)
 Identities = 6/9 (66%), Positives = 6/9 (66%)

Query:   167 ATADPNTNG 175
             A   PNTNG
Sbjct:   101 ANSGPNTNG 109   MatchSeq
```

```
 Score = 35 (19.8 bits)
 Identities = 6/13 (46%), Positives = 9/13 (69%)

Query:   306 FDVSANGLGTGRL 318
             FD+SA+    GR+
Sbjct:     8 FDISADDKSLGRI 20   MatchSeq
```

ButterflyBase butterfly|TNP00255_1 Total Score: 287

```
                      0         40        80        120       160
                      |         |         |         |         |  |167
butterfly|TNP00255_1  | ________________________________________________
Local hits (HSPs)     | ___              __          ___    ___
```

```
 Score = 102 (54.3 bits)
 Identities = 14/17 (82%), Positives = 16/17 (94%)

Query:    84 KQVQAFGSQSGKPSKNL 100
             KQV+AFGSQSGKPSK +
Sbjct:   142 KQVEAFGSQSGKPSKKI 158
```

```
 Score = 64 (34.7 bits)
 Identities = 9/9 (100%), Positives = 9/9 (100%)

Query:   244 TNHNGTGGK 252
             TNHNGTGGK
Sbjct:    70 TNHNGTGGK 78
```

```
 Score = 61 (33.2 bits)
 Identities = 8/10 (80%), Positives = 10/10 (100%)

Query:   289 RVYFNVSADG 298
             RV+F+VSADG
Sbjct:     7 RVFFDVSADG 16
```

```
 Score = 60 (32.7 bits)
 Identities = 8/8 (100%), Positives = 8/8 (100%)

Query:   147 KTSWLDGR 154
             KTSWLDGR
Sbjct:   120 KTSWLDGR 127
```

Ha SG EST hargln|HA-GLN Contig 715 FWD frame 1 Total Score: 343

```
                      0         60        120       180       240
                      |         |         |         |         |   |264
hargln|HA-GLN_Contig_ | ________________________________________________
Local hits (HSPs)     |    ___         _         _  ___
```

```
 Score = 102 (54.2 bits)
 Identities = 14/17 (82%), Positives = 16/17 (94%)

Query:    84 KQVQAFGSQSGKPSKNL 100
             KQV+AFGSQSGKPSK +
Sbjct:   169 KQVEAFGSQSGKPSKKI 185
```

```
 Score = 69 (37.3 bits)
 Identities = 9/10 (90%), Positives = 10/10 (100%)

Query:   289 RVYFNVSADG 298
             RVYF+VSADG
Sbjct:    34 RVYFDVSADG 43
```

```
 Score = 64 (34.7 bits)
 Identities = 9/9 (100%), Positives = 9/9 (100%)

Query:   244 TNHNGTGGK 252
             TNHNGTGGK
Sbjct:    97 TNHNGTGGK 105
```

```
 Score = 60 (32.6 bits)
 Identities = 8/8 (100%), Positives = 8/8 (100%)

Query:   147 KTSWLDGR 154
             KTSWLDGR
Sbjct:   147 KTSWLDGR 154
```

```
 Score = 48 (26.5 bits)
 Identities = 8/13 (61%), Positives = 10/13 (76%)

Query:   306 FDVSANGLGTGRL 318
             FDVSA+G   GR+
Sbjct:    37 FDVSADGSALGRI 49
```

Spot 65

m/z	Charge	Peak mW	Peptide mW	Delta (Da)	Delta (ppm)	Ladder Score	Log Likelihood	Start	End	Sequence	Modifications	Subs	Query Tool
716,848	2	1431,6804	1431,7106	-0,0302	-21,1104	72,8395	255,3439	1	14	TTHAFGSQSGKPSK			De-novo
716,848	2	1431,6804	1431,7106	-0,0302	-21,1104	72,8395	253,8231	1	14	THTAFGSQSGKPSK			De-novo
716,848	2	1431,6804	1431,6993	-0,0189	-13,2179	69,1358	250,6455	1	14	TPPNFGSQSGKPSK	Deamidation N	4	De-novo
417,7082	2	833,4008	833,4032	-0,0024	-2,9086	64,1026	71,9717	1	7	TSWLNGR	Deamidation N	5	De-novo
417,7082	2	833,4008	833,4032	-0,0024	-2,9086	64,1026	69,1287	1	7	TSWLDGR			De-novo
897,9119	2	1793,8082	1793,8077	0,0005	0,2654	44,4444	129,9671	1	17	AADMNDACLLSGWGNKK	Deamidation N	15	De-novo
897,9119	2	1793,8082	1793,8077	0,0005	0,2654	45,4545	129,7677	1	17	AADMNDACLLSGWGKNK	Deamidation N	16	De-novo
897,9119	2	1793,8082	1793,8077	0,0005	0,2654	44,4444	129,5186	1	17	AADMNNACLLSGWGNKK	Deamidation N	6 / 15	De-novo
725,3722	2	1448,7288	1448,7258	0,0030	2,0542	49,3827	119,2169	1	14	LNEAFGSKNSAPSK			De-novo
725,3722	2	1448,7288	1448,7258	0,0030	2,0542	49,3827	118,5758	1	14	NLEAFGSKNSAPSK			De-novo
725,3722	2	1448,7288	1448,726	0,0028	1,9162	47,1264	118,7983	1	15	LNEAFGASGSGKPSK			De-novo

NCBI gi|189014680|gb|ACD69576.1| peptidyl-prolyl isomerase-1 [Gryllus pennsylvanicus] Total Score: 149

```
                      0         40        80        120       160
                      |         |         |         |         | |165
gi|189014680|gb|ACD69 | ________________________________________________
Local hits (HSPs)     |                              ___    ____
```

```
 Score = 63 (34.1 bits)
 Identities = 10/11 (90%), Positives = 10/11 (90%)

Query:     4 AFGSQSGKPSK 14
             AFGSQSGK SK
Sbjct:   144 AFGSQSGKTSK 154   MatchSeq
```

```
 Score = 45 (24.9 bits)
 Identities = 6/8 (75%), Positives = 7/8 (87%)

Query:    42 KTSWLNGR 49
             KTSWL+ R
Sbjct:   118 KTSWLDNR 125   MatchSeq
```

```
 Score = 41 (22.8 bits)
 Identities = 7/8 (87%), Positives = 7/8 (87%)

Query:   121 KNLEAFGS 128
             K LEAFGS
Sbjct:   140 KRLEAFGS 147   MatchSeq
```

ButterflyBase LOP00067_1 Total Score: 150

```
                      0         40        80        120       160
                      |         |         |         |         | |165
butterfly|LOP00067_1  | ________________________________________________
Local hits (HSPs)     |                              ___    ____
```

```
 Score = 57 (30.9 bits)
 Identities = 9/11 (81%), Positives = 9/11 (81%)

Query:     4 AFGSQSGKPSK 14
             AFGSQ GK SK
Sbjct:   144 AFGSQAGKTSK 154
```

```
 Score = 54 (29.4 bits)
 Identities = 7/8 (87%), Positives = 8/8 (100%)

Query:    42 KTSWLNGR 49
             KTSWL+GR
Sbjct:   118 KTSWLDGR 125
```

```
 Score = 39 (21.7 bits)
 Identities = 6/8 (75%), Positives = 6/8 (75%)

Query:   121 KNLEAFGS 128
             K  EAFGS
Sbjct:   140 KQVEAFGS 147
```

Ha SG EST hargln|HA-GLN Contig 715 FWD frame 1 Total Score: 129

```
                      0         60        120       180       240
                      |         |         |         |         |   |264
hargln|HA-GLN_Contig_ | ________________________________________________
Local hits (HSPs)     |                        _    __
```

```
 Score = 75 (40.2 bits)
 Identities = 11/11 (100%), Positives = 11/11 (100%)

Query:     4 AFGSQSGKPSK 14
             AFGSQSGKPSK
Sbjct:   173 AFGSQSGKPSK 183
```

```
 Score = 54 (29.5 bits)
 Identities = 7/8 (87%), Positives = 8/8 (100%)

Query:    42 KTSWLNGR 49
             KTSWL+GR
Sbjct:   147 KTSWLDGR 154
```
